# Metal- and Solvent-Free Synthesis of Phosphinothioates, Phosphonothioates, Phosphorothioates, and Related Selenium Derivatives: An Efficient Green Protocol

**DOI:** 10.3390/molecules30102097

**Published:** 2025-05-09

**Authors:** Yajie Fu, Hexia Ye, Xinyao Zhao, Yingle Liu, Junchen Li, Xiaojing Bi

**Affiliations:** 1 College of Chemistry and Environmental Engineering, Sichuan University of Science and Engineering, Zigong 643002, China; yajief2022@163.com; 2 State Key Laboratory Chemistry for NBC Hazards Protection, Beijing 102205, China; yehexia6688@yeah.net (H.Y.); xy01253@yeah.net (X.Z.)

**Keywords:** phosphinothioates, phosphonothioates, phosphorothioates, metal-free, solvent-free

## Abstract

Methodologies for the effective fabrication of phosphinothioates, phosphonothioates, and phosphorothioates have always been of great interest due to their widespread application in many research fields. We present here a solvent-free reaction system for the synthesis of above compounds without any catalysts or additives, directly using disulfides with diarylphosphine oxides, dialkylphosphites, and phosphinates as substrates. Most of the target compounds were obtained in nearly quantitative yields by heating at 50 °C for 5–10 min or stirring at room temperature for 3 h. The products were efficiently separated via a water extraction operation, simultaneously recovering the by-product thiols. This mild and practical coupling protocol was also employed to prepare phosphinoselenoates from the corresponding diselenides with good yields.

## 1. Introduction

**Figure 1:**
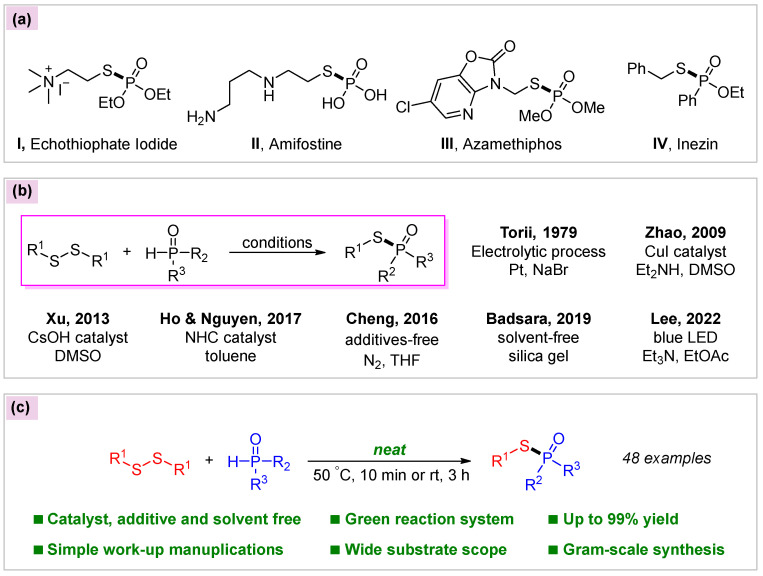
Significance and methods of synthesizing phosphinothioates, phosphonothioates, and phosphorothioates from disulfides. (**a**) Representative biologically thiophosphoryl compounds; (**b**) Reported typical approaches to thiophosphoryl compounds from disulfides [28,29,32,33,34,35,36]; (**c**) This work: Direct synthesis of thiophosphoryl compounds without any additives.

Phosphinothioates, phosphonothioates, and phosphorothioates, containing a typical P(O)-S group, are intriguing structural motifs that have great potential in the areas of pharmaceuticals [1,2,3,4,5], agrochemicals [6,7,8,9], and materials [10,11,12,13]. In the fields of medicinal and agricultural chemistry, some representative biologically active phosphonothioates and phosphorothioates are illustrated in Figure 1a, such as echothiophate iodide (I) [14,15], which is used for accommodative esotropia, amifostine (II) [16,17] for cancer adjuvant therapy, azamethiphos (III) [18,19] for insecticide, and inezin (IV) [20,21] for fungicide (Figure 1a). While the synthesis of these compounds can be achieved via relatively multifarious methodologies, traditional approaches generally rely on the incorporation of the toxic and moisture-sensitive halides R_2_P(O)Cl or RSO_2_Cl for nucleophilic substitution or Michaelis–Arbuzov reaction [22,23,24,25,26,27]. In particular, disulfides are attached to the HP(O) species (diarylphosphine oxides, dialkylphosphites, and phosphinates) to access phosphinothioates, phosphonothioates, and phosphorothioates. This has become a powerful method (Figure 1b). Pioneering work on direct coupling of dialkyl- or diarylphosphites and disulfides was achieved by Torii using an electrolytic technique with the assistance of NaBr [28]. In 2009, Zhao and co-workers developed a transition-metal catalysis protocol for the preparation of phosphorothioates from dialkylphosphites employing CuI as catalyst and Et_3_N as base [29]. Since then, a variety of relatively straightforward and effective methods based on disulfides have been reported, including base catalysis [30,31,32], N-heterocyclic carbene (NHC) catalysis [33], and photocatalysis [34]. Without any catalyst or oxidant and solely utilizing THF as the solvent under an N_2_ atmosphere, disulfides were coupled with diarylphosphine oxides [35]. Further, Badsara discovered that disulfides and diarylphosphine oxide could be efficiently converted in air at room temperature by employing inexpensive silica gel as the medium [36]. Most of these disulfide-based methods are characterized by simple conditions and convenient manipulations; however, the fly in the ointment is that the use of organic solvent or other types of media remains unavoidable.

As part of an ongoing research program in our group towards developing disulfides as viable feedstocks for organosulfur molecule synthesis [37,38], we are also very concerned about the development of disulfide-involved green synthetic methods, especially focusing on enhancing the simplicity of reaction systems, minimizing additive usage, and improving the convenience of work-up operations [39,40,41,42]. Herein, we report our efforts for the coupling of disulfides and HP(O) species without solvent or any additive under air, merely involving heating or stirring at room temperature. Broad substrate compatibility was demonstrated by successfully engaging substrates including diarylphosphine oxides, dialkylphosphites, and phosphinates with disulfides (Figure 1c).

## 2. Results

We commenced our investigation by examining the coupling of diphenyl disulfide **1a** and diphenylphosphine oxide **2a** (Table 1). Initially, we adopted Cheng’s conditions [35] as a reference; however, the temperature was adjusted from 80 °C to room temperature and N_2_ atmosphere to air atmosphere in this work. As expected, after stirring for 3 h at room temperature, a 75% yield of S-phenyl diphenylphosphinothioate **3a** was detected (entry 1). Other solvents (MeOH, DCM, MeCN, EtOAc, and 1,4-dioxane) were not advantageous for this transformation (entries 2–6), whereas the exciting aspect was that the yield of **3a** increased dramatically when H_2_O or DMF was utilized as the solvent (entries 7–8). Surprisingly, under solvent-free conditions, **1a** and **2a** were converted almost quantitatively into the corresponding product (entry 9). Shortening the reaction time from 3 h to 1 h only led to a slight decrease in yield (entry 10). Altering the substrate ratio had a great effect on the reaction. When the dosage of diphenylphosphine oxide **2a** was increased to 2 equivalents, the yield of **3a** significantly decreased to 42% (entry 11). Conversely, increasing the dosage of diphenyl disulfide **1a** to 2 equivalents did not result in a notable change in yield (entry 12). It is interesting that increasing the reaction temperature facilitated the conversion without any by-products (entries 13–16). When running the reaction at 50 °C, an impressive yield of 99% was achieved in just 10 min (entry 15). Practically, the by-product thiophenol generated in the reaction was easily removed by washing with saturated Na_2_CO_3_ aqueous solution, almost leaving the product only.

**Figure 2:**
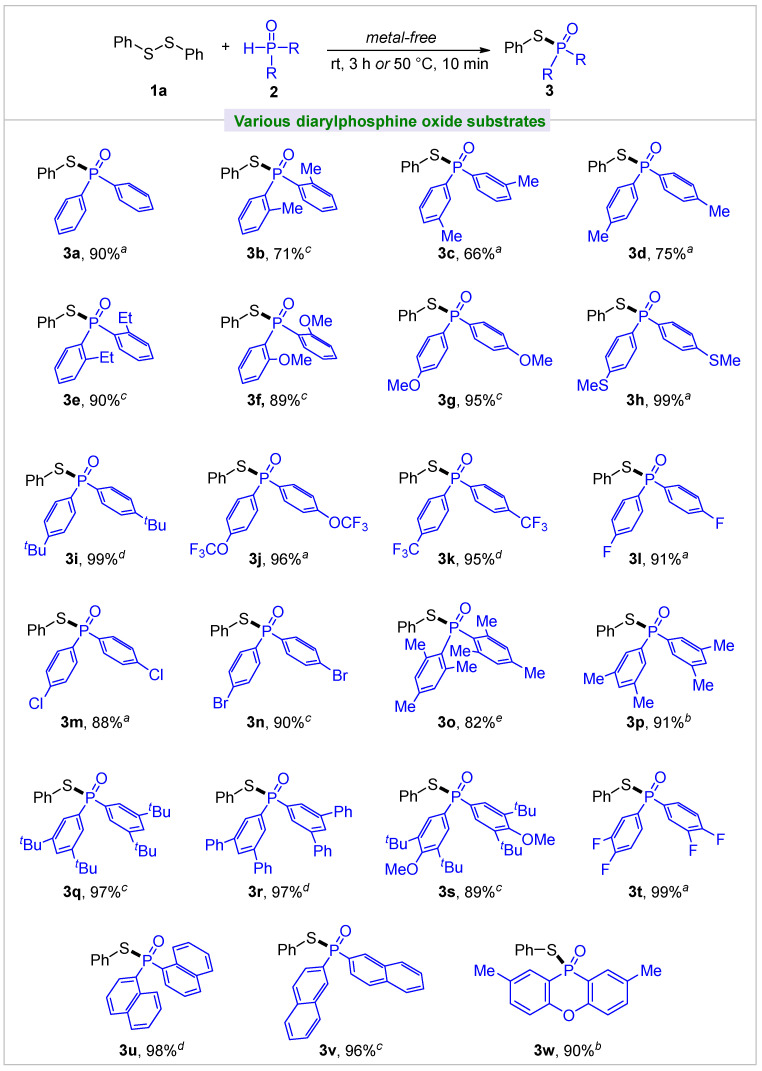
Substrate scope of diarylphosphine oxides. All yields are isolated. Standard conditions: diphenyl disulfide **1a** (0.3 mmol), diarylphosphine oxide. **2** (0.3 mmol). *^a^* Reaction was performed at rt for 3 h, or 50 °C for 10 min. *^b^* 50 °C for 1 h. *^c^* 70 °C for 1 h. *^d^* 70 °C for 3 h. *^e^* 70 °C for 7 h.

With a set of optimal conditions in hand, we evaluated the scope of the transformation with respect to other diarylphosphine oxides (Figure 2). A variety of functionalized diarylphosphine oxides were employed in the reaction with diphenyl disulfide **1a**, affording the phosphinothioates **3a**–**3w** with good to excellent yields. For example, diarylphosphine oxides with *ortho*-, *meta*-, or *para*-methyl substitution gave yields of 66–75% (**3b**–**3d**), whereas a higher yield of 90% was obtained with *ortho*-ethyl substitution (**3e**). Similar results were gained for MeO-substituted arenes (**3f** and **3g**). Notably, diarylphosphines with aryl groups bearing an electron-donating or electron-withdrawing substituent on the para-position proceeded smoothly to give the respective products in excellent yields (**3h**–**3n**), indicating that the electronic effect of the substituents might have hardly any impact on this transformation. It should be pointed out that a reactive group (Br, **3n**) was very compatible with the reaction. Moreover, aryl groups with two or more substituents were also successfully converted to the desired products with 82%–99% yields (**3o**–**3t**).

Other aromatic rings of diarylphosphine oxides, such as naphthalene (**3u** and **3v**) were well tolerated, giving high yields. Relatively rigid 2,8-dimethylphenoxaphosphinine 10-oxide was a suitable substrate that afforded a good yield of **3w**.

**Figure 3:**
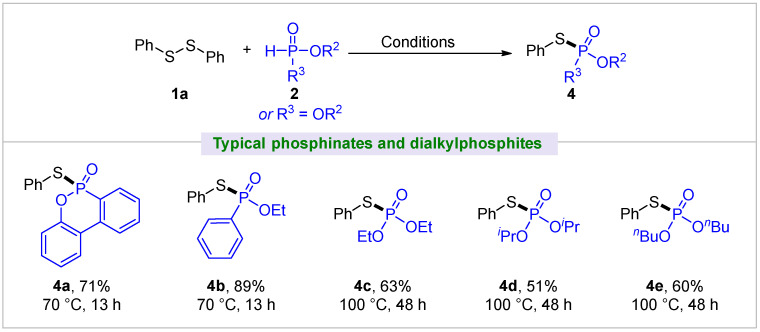
Substrate scope of phosphinates and dialkylphosphites. All yields are isolated. Standard conditions: diphenyl disulfide **1a** (0.3 mmol), phosphinates and dialkylphosphites **2** (0.3 mmol).

Next, we examined several HP(O) species that contain at least one P-O bond, specifically focusing on phosphinates and dialkylphosphites (Figure 3). Unlike diarylphosphine oxide substrates, these substrates exhibited comparatively lower reactivity with diphenyl disulfide **1a** and required higher reaction temperature as well as extended reaction time. For example, 6H-dibenzo[c,e][1,2]oxaphosphinine 6-oxide (DOPO), a flame retardant intermediate, showed good reactivity and generated the desired product **4a** with 73% yield at 70 °C for 13 h. (Ethoxy)phenylphosphinate gave a better yield of 89% for **4b** than DOPO under similar conditions. Though dialkylphosphites were also compatible with **1a**, relatively lower yields of **4c**–**4e** were obtained at an elevated temperature (100 °C) and longer time (48 h).

**Figure 4:**
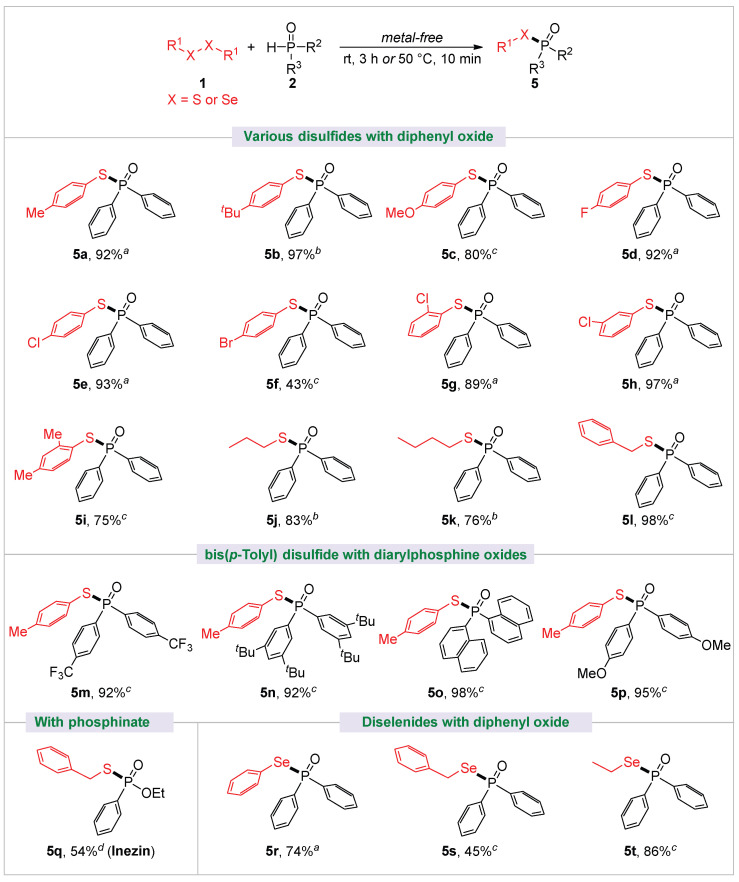
Substrate scope of disulfides and diselenides. All yields are isolated. Standard conditions: disulfide or diselenide **1** (0.3 mmol), diarylphosphine oxide **2** (0.3 mmol). *^a^* Reaction was performed at rt for 3 h, or 50 °C for 10 min. *^b^* 50 °C for 7 h. *^c^* 70 °C for 3 h. *^d^* 70 °C for 4 h.

The scope of the disulfide coupling partners was also identified (Figure 4). Employing diphenylphosphine oxide **2a** as the coupling substrate, most of the diaryl disulfides and dialkyl disulfides were transformed into the corresponding phosphinothioates with good to excellent yields (**5a**–**5e**, **5g**–**5l**). The effect of the substituted groups of diaryl disulfides for transformation was similar to the reaction of the above diarylphosphine oxides. It was apparent that both electron-donating and electron-withdrawing groups at the *para*-, *ortho*-, or *meta*-positions of diaryl disulfides were compatible, and the corresponding products (**5a**–**5e**, **5g**–**5i**) were obtained with good to high yields, whereas the competitive coupling of bis(4-bromophenyl) disulfide with **2a** resulted in a significant decrease in the observed yield of **5f**. When bis(*p*-tolyl) disulfide was used as the substrate, diarylphosphine oxides, bearing CF_3_, *^t^*Bu, MeO, or a naphthyl group, all proceeded well to give products with 92%–98% yields (**5m**–**5p**). As for phosphinate, for example, (ethoxy)phenylphosphinate reacted well to afford **5q** (inezin, a fungicide) in a synthetically useful yield of 54%. Excitingly, the reaction was found to be tolerant of diaryl or dialkyl diselenides, and moderate to good yields of **5r**–**5s** were obtained by reacting with diphenylphosphine oxide.

**Figure 5:**
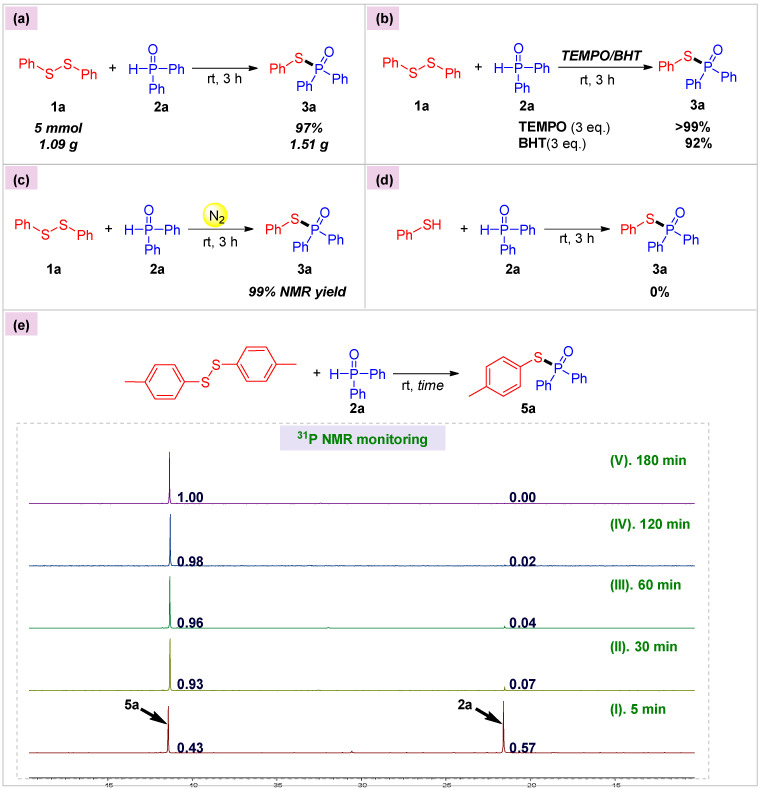
Gram-scale preparation and mechanistic study reactions. (**a**) Scalability experiment; (**b**) Radical inhibition experiments; (**c**) Air-excluding experiment; (**d**) Intermediate determination; (**e**) Reaction process monitoring experiments.

To demonstrate the synthetic utility of our protocol, we applied this method to the preparation of **3a** on a 5 mmol scale, and the reaction occurred smoothly at room temperature to give **3a** in a nearly quantitative yield (Figure 5a). Then, to gain further insights into this reaction, we conducted several control experiments (Figure 5b–e). Based on Han’s radical 1,2-bisphosphorylation of alkynes [43], we first considered whether this coupling proceeded via a radical process. When 3 equivalents of a radical scavenger, TEMPO or BHT, were added, the reaction still ran well to produce **3a** with 99% and 92% yields, respectively (Figure 5b). What is more, running the reaction under an N_2_ atmosphere brought about **3a** in a yield of 99% (Figure 5c). These results suggested that this transformation might not involve a radical mechanism. Thiophenol, the only by-product observed in the reaction process, did not couple with diphenylphosphine oxide **2a** (Figure 5d), implying that thiophenol was probably not a reaction intermediate. A series of ^31^P NMR monitoring experiments were then performed with model **2a** using bis(*p*-tolyl) disulfide as the coupling partner (Figure 5e). According to the integral area of **5a** and **2a**, we could clearly observe that no other phosphorus-containing intermediates were formed during the reaction.

**Figure 6:**
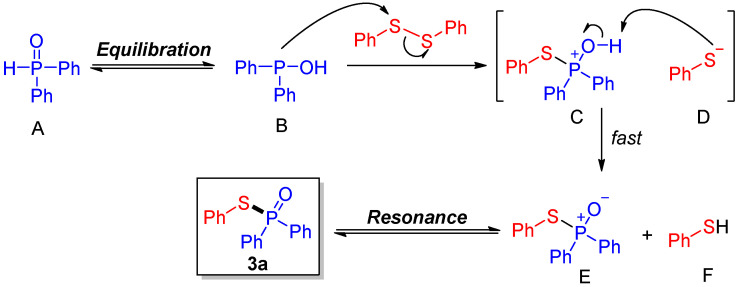
Proposed mechanism.

Based on our experimental findings, we hypothesize that the reaction proceeds through a classical ionic process, shown in Figure 6. The nucleophilic hydroxydiphenylphosphane **B**, the equilibration form of diphenphosphine oxide **A**, attacks diphenyl disulfide to give a possible ion pair that is composed of a phosphonium cation **C** and a thiophenol anion **D**. Fast intramolecular proton transfer of this ion pair occurred to generate the desired product **3a** (resonant structure **E**) and the by-product thiophenol **F**.

## 3. Materials and Methods

Unless otherwise specified, materials obtained from commercial suppliers were used directly without further purification. Reactions were monitored with an Agilent 8890-5977B Fisher Polaris Q GC-MS or Agilent 1260-LC/MSD (Santa Clara, CA, USA). High-resolution mass spectra (HRMS) were recorded on a Waters Xevo G2-XS QTOF (Waters, Medford, MA, USA) using the electrospray ionization (ESI) mode. Flash column chromatography was carried out on silica gel (200–300 mesh) from Qingdao Haiyang Huagong Co., Ltd. (Qingdao, China). Thin-layer chromatography (TLC) was performed using silica gel GF254 plates (Yantai Chemical Industry, Shandong, China). ^1^H NMR, ^13^C NMR, ^31^P NMR, and ^19^F NMR spectra were recorded on a Bruker Advance 300 spectrometer at ambient temperature in CDCl_3_. The residual solvent protons (^1^H NMR @ δ 0.00 ppm) or the solvent carbons (^13^C NMR @ δ 77.16 ppm) were used as internal standards. For ^19^F NMR, CF_3_COOC_2_H_5_ was used as the reference (^19^F NMR @ δ −75.80 ppm) with chemical shift at 0 ppm. ^31^P NMR spectra were referenced externally to phosphoric acid (^31^P NMR @ δ −0.00 ppm). ^1^H NMR data are presented as chemical shift in ppm (δ) downfield from tetramethylsilane. NMR data are reported as chemical shift, multiplicity (s = singlet, d = doublet, t = triplet, q = quartet, dd = doublet of doublets, td = triplet of doublets, qd = quartet of doublets, m = multiplet), coupling constants (Hz), and integration.

Experimental Procedure.

Disulfide **1** (0.3 mmol, 1.0 equiv.) and P(O)H compound **2** (0.3 mmol, 1.0 equiv.) were added to a 4 mL sealed tube and the mixture was then stirred at the corresponding temperature in air for several hours. The reaction was monitored by TLC. After completion of the reaction, DCM (10 mL) was added and the mixture was washed with saturated Na_2_CO_3_ solution (3 × 10 mL). During this weak alkali wash process, mercaptan was removed and recovered efficiently. The combined organic layer was dried over anhydrous Na_2_SO_4_ and concentrated by vacuum. In most cases, the products were obtained without any further purification. In some cases, the residues were purified by flash column chromatography on silica gel to provide the corresponding product with good to excellent yields.

General procedure for the gram-scale synthesis:

1,2-Diphenyldisulfane **1a** (5 mmol, 1.0 equiv.) and diphenylphosphine oxide **2a** (5 mmol, 1.0 equiv.) were added to a 10 mL sealed tube and the mixture was then stirred at room temperature under air for 3 h. The reaction was monitored by TLC. After completion of the reaction, DCM (20 mL) was added and the mixture was washed with saturated Na_2_CO_3_ solution (3 × 20 mL). During this weak alkali wash process, mercaptan was removed and recovered efficiently. The combined organic layer was dried over anhydrous Na_2_SO_4_ and concentrated by vacuum. The residues were purified by flash column chromatography on silica gel to provide the corresponding product in 97% yields.

*S*-Phenyl diphenylphosphinothioate (**3a**) [44]. 25 °C for 3h. White solid, 90% yield. ^1^H NMR (300 MHz, chloroform-d) δ 7.92–7.77 (m, 4H), 7.50–7.35 (m, 8H), 7.18 (t, *J* = 7.9 Hz, 3H). ^13^C NMR (75 MHz, chloroform-d) δ 135.33 (d, *J* = 3.9 Hz), 132.46 (d, *J* =105.1 Hz), 132.30 (d, *J* = 3.1 Hz), 131.55 (d, *J* = 10.2 Hz), 129.09 (d, *J* = 1.7 Hz), 128.90 (d, *J* = 2.2 Hz), 128.51 (d, *J* = 13.1 Hz), 126.12 (d, *J* = 5.2 Hz). ^31^P NMR (121 MHz, chloroform-d) δ 40.2.

*S*-Phenyl di-*o*-tolylphosphinothioate (**3b**) [45]. 70 °C for 1h. White solid, 71% yield. ^1^H NMR (300 MHz, chloroform-d) δ 7.79 (dd, *J* = 15.0, 7.6 Hz, 2H), 7.47 (d, *J* = 7.3 Hz, 2H), 7.39 (t, *J* = 7.4 Hz, 2H), 7.30–7.14 (m, 7H), 2.39 (s, 6H). ^13^C NMR (75 MHz, chloroform-d) δ 142.05 (d, *J* = 9.8 Hz), 135.81 (d, *J* = 3.8 Hz), 132.79 (d, *J* = 11.8 Hz), 132.36 (d, *J* = 3.0 Hz), 131.96 (d, *J* = 12.1 Hz), 131.54 (d, *J* = 102.8 Hz), 129.11 (d, *J* = 1.8 Hz), 128.94 (d, *J* = 2.2 Hz), 126.39 (d, *J* = 4.9 Hz), 125.64 (d, *J* = 13.4 Hz), 21.57 (d, *J* = 4.1 Hz). ^31^P NMR (121 MHz, chloroform-d) δ 42.67.

*S*-Phenyl di-*m*-tolylphosphinothioate (**3c**). 25 °C for 3h. Oily liquid, 66% yield. ^1^H NMR (300 MHz, chloroform-d) δ 7.72–7.56 (m, 4H), 7.46 (d, *J* = 7.8 Hz, 2H), 7.36–7.27 (m, 4H), 7.21 (q, *J* = 7.0, 6.2 Hz, 3H), 2.34 (s, 6H). ^13^C NMR (75 MHz, chloroform-d) δ 138.51 (d, *J* = 13.0 Hz), 135.43 (d, *J* = 3.8 Hz), 133.15 (d, *J* = 3.1 Hz), 132.35 (d, *J* = 106.4 Hz), 132.15 (d, *J* = 9.9 Hz), 129.13 (d, *J* = 1.8 Hz), 128.92 (d, *J* = 2.2 Hz), 128.70, 128.53 (d, *J* = 3.3 Hz), 128.32, 126.38 (d, *J* = 5.2 Hz), 21.43. ^31^P NMR (121 MHz, chloroform-d) δ 40.87. HRMS (ESI): m/z calculated for C_20_H_20_OPS^+^ [M+H] ^+^: 339.0974, found 339.0967.

*S*-Phenyl di-*p*-tolylphosphinothioate (**3d**) [36]. 25 °C for 3h. White solid, 75% yield. ^1^H NMR (300 MHz, chloroform-d) δ 7.72 (dd, *J* = 12.7, 8.0 Hz, 4H), 7.46 (d, *J* = 7.3 Hz, 2H), 7.21 (t, *J* = 7.6 Hz, 7H), 2.37 (s, 6H). ^13^C NMR (75 MHz, chloroform-d) δ 142.92 (d, *J* = 3.0 Hz), 135.34 (d, *J* = 3.9 Hz), 131.72 (d, *J* = 10.7 Hz), 130.31, 129.35 (d, *J* = 13.6 Hz), 129.16 (d, *J* = 1.6 Hz), 128.85, 126.77 (d, *J* = 5.1 Hz), 21.73 (d, *J* = 1.2 Hz). ^31^P NMR (121 MHz, chloroform-d) δ 40.71.

*S*-Phenyl bis(2-ethoxyphenyl)phosphinothioate (**3e**). 70 °C for 1h. Oily liquid, 90% yield. ^1^H NMR (300 MHz, chloroform-d) δ 7.79 (dd, *J* = 15.2, 7.7 Hz, 2H), 7.50 (d, *J* = 7.4 Hz, 2H), 7.42 (t, *J* = 7.5 Hz, 2H), 7.29–7.16 (m, 7H), 1.00 (t, *J* = 7.5 Hz, 6H). ^13^C NMR (75 MHz, chloroform-d) δ 148.52 (d, *J* = 10.1 Hz), 135.66 (d, *J* = 3.8 Hz), 132.60 (d, *J* = 12.3 Hz), 132.43 (d, *J* = 3.0 Hz), 131.65 (d, *J* = 102.6 Hz), 130.23 (d, *J* = 12.0 Hz), 129.07 (d, *J* = 1.8 Hz), 128.81 (d, *J* = 2.2 Hz), 126.75 (d, *J* = 4.9 Hz), 125.47 (d, *J* = 13.5 Hz), 27.12 (d, *J* = 4.3 Hz), 15.35. ^31^P NMR (121 MHz, chloroform-d) δ 43.49. HRMS (ESI): m/z calculated for C_22_H_24_OPS^+^ [M+H] ^+^: 367.1281, found 367.1280.

*S*-Phenyl bis(2-methoxyphenyl)phosphinothioate (**3f**). 70 °C for 1h. White solid, 89% yield, m.p: 134.5–136.2 °C. ^1^H NMR (300 MHz, chloroform-d) δ 7.81 (ddd, *J* = 15.2, 7.6, 1.5 Hz, 2H), 7.44 (t, *J* = 7.4 Hz, 4H), 7.18 (q, *J* = 6.7, 6.2 Hz, 3H), 6.98 (td, *J* = 7.4, 2.1 Hz, 2H), 6.90–6.79 (m, 2H), 3.66 (s, 6H). ^13^C NMR (75 MHz, chloroform-d) δ 160.42 (d, *J* = 3.6 Hz), 135.83 (d, *J* = 4.1 Hz), 133.91, 133.82 (d, *J* = 2.1 Hz), 128.73 (d, *J* = 1.9 Hz), 128.48 (d, *J* = 2.4 Hz), 127.82 (d, *J* = 5.7 Hz), 122.87, 121.40, 120.47 (d, *J* = 13.2 Hz), 111.30 (d, *J* = 7.3 Hz), 55.68. ^31^P NMR (121 MHz, chloroform-d) δ 35.56. HRMS (ESI): m/z calculated for C_20_H_20_O_3_PS^+^ [M+H] ^+^: 371.0865, found 371.0865.

*S*-Phenyl bis(4-methoxyphenyl)phosphinothioate (**3g**) [46]. 70 °C for 1h. White solid, 95% yield. ^1^H NMR (300 MHz, chloroform-d) δ 7.76 (dd, *J* = 12.2, 8.7 Hz, 4H), 7.45 (d, *J* = 7.6 Hz, 2H), 7.21 (q, *J* = 7.0, 6.2 Hz, 3H), 6.93 (dd, *J* = 8.8, 2.6 Hz, 4H), 3.83 (s, 6H). ^13^C NMR (75 MHz, chloroform-d) δ 162.78 (d, *J* = 3.1 Hz), 135.30 (d, *J* = 3.9 Hz), 133.65 (d, *J* = 11.8 Hz), 129.17 (d, *J* = 1.9 Hz), 128.84 (d, *J* = 2.2 Hz), 127.03 (d, *J* = 5.1 Hz), 124.03 (d, *J* = 115.0 Hz), 114.13 (d, *J* = 14.3 Hz), 55.49. ^31^P NMR (121 MHz, chloroform-d) δ 35.64.

*S*-Phenyl bis(4-(methylthio)phenyl)phosphinothioate (**3h**). 25 °C for 3h. Oily liquid, 99% yield. ^1^H NMR (300 MHz, chloroform-d) δ 7.70 (dd, *J* = 12.4, 8.3 Hz, 4H), 7.45 (d, *J* = 7.3 Hz, 2H), 7.23 (h, *J* = 7.1, 6.4 Hz, 7H), 2.46 (s, 6H). ^13^C NMR (75 MHz, chloroform-d) δ 145.08 (d, *J* = 3.3 Hz), 135.26 (d, *J* = 3.9 Hz), 131.77 (d, *J* = 11.0 Hz), 129.20 (d, *J* = 1.8 Hz), 128.96 (d, *J* = 2.3 Hz), 127.93 (d, *J* = 111.8 Hz), 126.29 (d, *J* = 5.2 Hz), 125.08 (d, *J* = 13.7 Hz), 14.70. ^31^P NMR (121 MHz, chloroform-d) δ 39.80; HRMS (ESI): m/z calculated for C_20_H_20_OPS_3_^+^ [M+H] ^+^: 403.0409, found 403.0407.

*S*-Phenyl bis(4-(tert-butyl)phenyl)phosphinothioate (**3i**). 70 °C for 3h. White solid, 99% yield, m.p: 137.1–138.3 °C. ^1^H NMR (300 MHz, chloroform-d) δ 7.83–7.70 (m, 1H), 7.50–7.39 (m, 2H), 7.27–7.12 (m, 1H), 1.31 (s, 6H). ^13^C NMR (75 MHz, chloroform-d) δ 155.85 (d, *J* = 3.1 Hz), 135.41 (d, *J* = 3.9 Hz), 131.61 (d, *J* = 10.6 Hz), 130.33, 129.10 (d, *J* = 1.7 Hz), 128.88, 128.79 (d, *J* = 2.2 Hz), 126.90 (d, *J* = 5.1 Hz), 125.61 (d, *J* = 13.4 Hz), 35.15, 31.20. ^31^P NMR (121 MHz, chloroform-d) δ 40.30. HRMS (ESI): m/z calculated for C_26_H_32_OPS^+^ [M+H] ^+^: 423.1904, found 423.1906.

*S*-Phenyl bis(4-(trifluoromethoxy)phenyl)phosphinothioate (**3j**). 25 °C for 3h. Yellow solid, 96% yield, m.p: 66.7–68.4 °C. ^1^H NMR (300 MHz, chloroform-d) δ 7.89 (dd, *J* = 12.3, 8.8 Hz, 4H), 7.46–7.39 (m, 2H), 7.26 (dd, *J* = 15.6, 8.5 Hz, 7H). ^13^C NMR (75 MHz, chloroform-d) δ 152.41 (dd, *J* = 3.5, 1.7 Hz), 135.55 (d, *J* = 4.1 Hz), 133.72 (d, *J* = 11.5 Hz), 130.69 (d, *J* = 109.5 Hz), 129.47 (d, *J* = 1.9 Hz), 125.25 (d, *J* = 5.4 Hz), 120.68 (d, *J* = 14.1 Hz), 120.35 (d, *J* = 259.2 Hz). ^31^P NMR (121 MHz, chloroform-d) δ 37.20. ^19^F NMR (282 MHz, chloroform-d) δ −56.84. HRMS (ESI): m/z calculated for C_20_H_14_F_6_O_3_PS^+^ [M+H] ^+^: 479.0301, found 479.0300.

*S*-Phenyl bis(4-(trifluoromethyl)phenyl)phosphinothioate (**3k**). Yellow solid, 95% yield, m.p: 100.5–102.2 °C. ^1^H NMR (300 MHz, chloroform-d) δ 7.98 (dd, *J* = 12.5, 8.0 Hz, 4H), 7.73 (d, *J* = 6.1 Hz, 4H), 7.45 (d, *J* = 7.5 Hz, 2H), 7.26 (p, *J* = 7.2 Hz, 3H). ^13^C NMR (75 MHz, chloroform-d) δ 137.00, 135.62 (d, *J* = 3.9 Hz), 134.47 (dd, *J* = 32.9, 3.2 Hz), 132.16 (d, *J* = 10.6 Hz), 129.68 (dd, *J* = 8.8, 2.2 Hz), 125.74 (dq, *J* = 13.5, 3.7 Hz), 125.29, 124.60 (d, *J* = 5.4 Hz), 121.66. ^31^P NMR (121 MHz, chloroform-d) δ 36.56. ^19^F NMR (282 MHz, chloroform-d) δ −62.39. HRMS (ESI): m/z calculated for C_20_H_14_F_6_OPS^+^ [M+H] ^+^: 447.0400, found 447.0402.

*S*-Phenyl bis(4-fluorophenyl)phosphinothioate (**3l**). 25 °C for 3h. Oily liquid, 91% yield, m.p: 78.3–79 °C. ^1^H NMR (300 MHz, chloroform-d) δ 7.84 (ddd, *J* = 12.3, 8.7, 5.5 Hz, 4H), 7.47–7.42 (m, 2H), 7.30–7.08 (m, 7H). ^13^C NMR (75 MHz, chloroform-d) δ 166.96 (d, *J* = 3.6 Hz), 163.59 (d, *J* = 3.6 Hz), 135.34 (d, *J* = 4.0 Hz), 134.13 (dd, *J* = 11.9, 8.9 Hz), 129.24 (dd, *J* = 5.7, 2.1 Hz), 127.52 (d, *J* = 3.4 Hz), 125.69 (d, *J* = 5.3 Hz), 116.05 (dd, *J* = 21.5, 14.5 Hz). ^31^P NMR (121 MHz, chloroform-d) δ 38.05. ^19^F NMR (282 MHz, chloroform-d) δ −105.57–−105.67 (m). HRMS (ESI): m/z calculated for C_18_H_14_F_2_OPS^+^[M+H] ^+^: 347.0464, found 347.0466.

*S*-Phenyl bis(4-chlorophenyl)phosphinothioate (**3m**) [45]. 25 °C for 3h. Oily liquid, 88% yield. ^1^H NMR (300 MHz, chloroform-d) δ 7.76 (dd, *J* = 12.4, 8.5 Hz, 4H), 7.46–7.38 (m, 6H), 7.28–7.19 (m, 3H). ^13^C NMR (75 MHz, methylene chloride-d2) δ 139.26 (d, *J* = 3.7 Hz), 135.43 (d, *J* = 4.0 Hz), 132.97 (d, *J* = 11.2 Hz), 130.75 (d, *J* = 109.4 Hz), 129.39 (d, *J* =2.3 Hz), 129.09 (d, *J* = 13.9 Hz), 125.39 (d, *J* = 5.3 Hz). ^31^P NMR (121 MHz, chloroform-d) δ 38.10.

*S*-Phenyl bis(4-bromophenyl)phosphinothioate (**3n**). 70 °C for 1h. White solid, 90% yield, m.p: 157.8–159.6 °C. ^1^H NMR (300 MHz, chloroform-d) δ 7.68 (dd, *J* = 12.2, 8.5 Hz, 4H), 7.59 (dd, *J* = 8.3, 3.0 Hz, 4H), 7.44 (d, *J* = 8.0 Hz, 2H), 7.25 (p, *J* = 6.3 Hz, 3H). ^13^C NMR (75 MHz, chloroform-d) δ 135.46 (d, *J* = 4.0 Hz), 133.06 (d, *J* = 11.1 Hz), 132.79 (d, *J* = 11.2 Hz), 132.06 (d, *J* = 13.7 Hz), 131.16 (d, *J* = 108.7 Hz)), 129.46 (d, *J* = 2.0 Hz), 127.97 (d, *J* = 3.9 Hz), 125.26 (d, *J* = 5.3 Hz). ^31^P NMR (121 MHz, chloroform-d) δ 38.22. HRMS (ESI): m/z calculated for C_18_H_14_Br_2_OPS^+^ [M+H] ^+^: 466.8862, found 466.8864.

*S*-Phenyl dimesitylphosphinothioate (**3o**). White solid, 82% yield, m.p: 167.8–169.5 °C. ^1^H NMR (300 MHz, chloroform-d) δ 7.29–7.18 (m, 3H), 7.12 (t, *J* = 7.6 Hz, 2H), 6.74 (d, *J* = 4.3 Hz, 4H), 2.25 (d, *J* = 5.7 Hz, 18H). ^13^C NMR (75 MHz, chloroform-d) δ 141.45 (d, *J* = 11.3 Hz), 141.13 (d, *J* = 3.0 Hz), 137.19 (d, *J* = 3.8 Hz), 130.89 (d, *J* = 12.7 Hz), 130.67 (d, *J* = 100.6 Hz), 129.03 (d, *J* = 2.8 Hz), 128.49 (d, *J* = 2.4 Hz), 127.54 (d, *J* = 5.6 Hz), 22.92 (d, *J* = 4.0 Hz), 21.08 (d, *J* = 1.5 Hz). ^31^P NMR (121 MHz, chloroform-d) δ 45.53. HRMS (ESI): m/z calculated for C_24_H_28_OPS^+^ [M+H] ^+^: 395.1590, found 395.1593.

*S*-Phenyl bis(3,5-dimethylphenyl)phosphinothioate (**3p**) [36]. White solid, 91% yield. ^1^H NMR (300 MHz, chloroform-d) δ 7.43 (d, *J* = 13.3 Hz, 6H), 7.19 (d, *J* = 6.9 Hz, 3H), 7.09 (s, 2H), 2.29 (s, 12H). ^13^C NMR (75 MHz, chloroform-d) δ 138.25 (d, *J* = 13.9 Hz), 135.39 (d, *J* = 3.8 Hz), 134.04 (d, *J* = 3.3 Hz), 132.33 (d, *J* = 105.7 Hz), 129.16 (d, *J* = 10.4 Hz), 128.81 (d, *J* = 2.2 Hz), 126.65 (d, *J* = 5.1 Hz), 21.32. ^31^P NMR (121 MHz, chloroform-d) δ 41.38.

*S*-Phenyl bis(3,5-di-tert-butylphenyl)phosphinothioate (**3q**). White solid, 97% yield, m.p: 127.6–128.6 °C. ^1^H NMR (300 MHz, chloroform-d) δ 7.68 (dd, *J* = 13.7, 1.4 Hz, 4H), 7.54 (s, 2H), 7.41 (d, *J* = 7.6 Hz, 2H), 7.16 (q, *J* = 8.0, 6.6 Hz, 3H), 1.30 (s, 36H). ^13^C NMR (75 MHz, chloroform-d) δ 150.98 (d, *J* = 12.8 Hz), 135.39 (d, *J* = 3.8 Hz), 131.61 (d, *J* = 105.8 Hz), 128.93 (d, *J* = 1.8 Hz), 128.63 (d, *J* = 2.2 Hz), 126.35 (d, *J* = 3.1 Hz), 125.93 (d, *J* = 10.9 Hz), 35.12, 31.40. ^31^P NMR (121 MHz, chloroform-d) δ 43.24. HRMS (ESI): m/z calculated for C_34_H_48_OPS^+^ [M+H] ^+^: 535.3155, found 535.3158.

*S*-Phenyl bis(3,5-di-phenyl)phosphinothioate (**3r**). Yellow solid, 97% yield, m.p: 148.3–149.9 °C. ^1^H NMR (300 MHz, chloroform-d) δ 8.13 (d, *J* = 13.2 Hz, 4H), 7.93 (s, 2H), 7.60 (d, *J* = 7.3 Hz, 10H), 7.40 (dt, *J* = 23.4, 7.2 Hz, 12H), 7.27–7.20 (m, 3H). ^13^C NMR (75 MHz, chloroform-d) δ 142.23 (d, *J* = 13.7 Hz), 139.70 (d, *J* = 1.0 Hz), 135.39 (d, *J* = 3.9 Hz), 133.69 (d, *J* = 105.7 Hz), 129.96 (d, *J* = 3.0 Hz), 129.24 (d, *J* = 1.7 Hz), 129.08 (d, *J* = 2.1 Hz), 128.93 (d, *J* = 5.7 Hz), 128.00, 127.21, 126.05 (d, *J* = 5.2 Hz). ^31^P NMR (121 MHz, chloroform-d) δ 40.46. HRMS (ESI): m/z calculated for C_42_H_32_OPS^+^ [M+H] ^+^: 615.1904, found 615.1906.

*S*-Phenyl bis(3,5-di-tert-butyl-4-methoxyphenyl)phosphinothioate (**3s**). White solid, 89% yield. m.p: 139.5–141.2 °C. ^1^H NMR (300 MHz, chloroform-d) δ 7.71 (s, 2H), 7.66 (s, 2H), 7.41 (d, *J* = 7.8 Hz, 2H), 7.23–7.11 (m, 3H), 3.67 (s, 6H), 1.38 (s, 36H). ^13^C NMR (75 MHz, chloroform-d) δ 163.17 (d, *J* = 3.6 Hz), 144.19 (d, *J* = 13.2 Hz), 135.37 (d, *J* = 3.8 Hz), 130.45 (d, *J* = 11.9 Hz), 128.95 (d, *J* = 1.7 Hz), 128.62 (d, *J* = 2.1 Hz), 127.69 (d, *J* = 5.0 Hz), 126.11 (d, *J* = 110.5 Hz), 64.59, 36.12, 32.00. ^31^P NMR (121 MHz, chloroform-d) δ 42.57. HRMS (ESI): m/z calculated for C_36_H_52_O_3_PS^+^ [M+H] ^+^: 595.3372, found 595.3369.

*S*-Phenyl bis(3,5-difluorophenyl)phosphinothioate (**3t**). White solid, 99% yield. m.p: 85.7–86.5 °C. ^1^H NMR (300 MHz, chloroform-d) δ 7.73–7.56 (m, 4H), 7.46 (d, *J* = 7.9 Hz, 2H), 7.34–7.22 (m, 5H). ^13^C NMR (75 MHz, chloroform-d) δ 154.98 (dd, *J* = 12.6, 3.2 Hz), 152.12 (dd, *J* = 19.7, 12.8 Hz), 151.57 (dd, *J* = 12.6, 3.2 Hz), 148.75 (dd, *J* = 19.7, 12.8 Hz), 135.46 (d, *J* = 4.1 Hz), 129.92 (t, *J* = 4.1 Hz), 129.65 (dd, *J* = 7.5, 2.2 Hz), 128.59 (ddt, *J* = 14.5, 7.4, 4.0 Hz), 124.77 (d, *J* = 5.4 Hz), 121.01 (ddd, *J* = 18.4, 12.4, 1.7 Hz), 118.35 (dd, *J* = 17.6, 16.0 Hz). ^31^P NMR (121 MHz, chloroform-d) δ 35.20. ^19^F NMR (282 MHz, chloroform-d) δ −128.76–−128.91 (m), −133.89–−134.04 (m). HRMS (ESI): m/z calculated for C_18_H_12_F_4_OPS^+^ [M+H] ^+^:383.0279, found 383.0278.

*S*-Phenyl di(naphthalen-1-yl)phosphinothioate (**3u**). White solid, 98% yield. m.p: 158.2–159.2 °C. ^1^H NMR (300 MHz, chloroform-d) δ 8.90–8.81 (m, 2H), 8.11–8.05 (m, 1H), 8.04–7.97 (m, 3H), 7.88 (dt, *J* = 6.8, 2.1 Hz, 2H), 7.58–7.37 (m, 8H), 7.24–7.09 (m, 3H). ^13^C NMR (75 MHz, chloroform-d) δ 135.36 (d, *J* = 4.1 Hz), 134.03 (d, *J* = 10.2 Hz), 133.88 (d, *J* = 3.3 Hz), 133.62 (d, *J* = 11.5 Hz), 133.32 (d, *J* = 9.5 Hz), 129.11 (d, *J* = 1.9 Hz), 129.02 (d, *J* = 103.0 Hz), 128.96 (d, *J* = 1.7 Hz), 128.88 (d, *J* = 2.2 Hz), 127.48, 127.27 (d, *J* = 5.0 Hz), 127.10 (d, *J* = 5.2 Hz), 126.63, 124.50 (d, *J* = 15.4 Hz). ^31^P NMR (121 MHz, chloroform-d) δ 44.24. HRMS (ESI): m/z calculated for C_26_H_20_OPS^+^ [M+H] ^+^: 411.0967, found 411.0967.

S-Phenyl di(naphthalen-2-yl)phosphinothioate (3v) [47]. White solid, 96% yield. ^1^H NMR (300 MHz, chloroform-d) δ 8.46 (d, *J* = 14.8 Hz, 2H), 7.88 (dd, *J* = 14.9, 7.2 Hz, 8H), 7.67–7.54 (m, 3H), 7.54–7.48 (m, 3H), 7.18 (t, *J* = 8.0 Hz, 3H). ^13^C NMR (75 MHz, chloroform-d) δ 135.51 (d, *J* = 3.9 Hz), 134.94 (d, *J* = 2.7 Hz), 134.09 (d, *J* = 9.4 Hz), 132.51 (d, *J* = 14.4 Hz), 130.42, 129.30 (d, *J* = 1.8 Hz), 129.10 (d, *J* = 2.4 Hz), 129.09 (d, *J* = 15.0 Hz), 128.60 (d, *J* = 3.7 Hz), 127.92, 127.79 (d, *J* = 100.6 Hz), 127.12, 126.40, 126.21 (d, *J* = 6.3 Hz). ^31^P NMR (121 MHz, chloroform-d) δ 40.30.

2,8-Dimethyl-10-(phenylthio)phenoxaphosphinine 10-oxide (**3w**). White solid, 90% yield, m.p: 124.7–125.5 °C. ^1^H NMR (300 MHz, chloroform-d) δ 7.74 (d, *J* = 14.5 Hz, 2H), 7.33 (d, *J* = 8.5 Hz, 2H), 7.22 (t, *J* = 7.3 Hz, 1H), 7.03 (t, *J* = 7.7 Hz, 2H), 6.94 (t, *J* = 8.0 Hz, 2H), 6.86 (d, *J* = 7.7 Hz, 2H), 2.39 (s, 6H). ^13^C NMR (75 MHz, chloroform-d) δ 153.92 (d, *J* = 4.1 Hz), 136.08 (d, *J* = 4.0 Hz), 135.31 (d, *J* = 2.3 Hz), 133.54 (d, *J* = 11.5 Hz), 129.93 (d, *J* = 4.6 Hz), 129.17 (d, *J* = 3.2 Hz), 128.87 (d, *J* = 2.7 Hz), 127.08 (d, *J* = 5.8 Hz), 117.62 (d, *J* = 7.4 Hz), 114.37 (d, *J* = 105.3 Hz), 20.71. ^31^P NMR (121 MHz, chloroform-d) δ 18.09. HRMS (ESI): m/z calculated for C_20_H_18_O_2_PS^+^[M+H] ^+^: 353.0760, found 353.0760.

6-(Phenylthio)dibenzo[c,e] [1,2] oxaphosphinine 6-oxide (**4a**). Oily liquid, 71% yield. ^1^H NMR (300 MHz, chloroform-d) δ 7.94–7.77 (m, 2H), 7.74–7.60 (m, 2H), 7.46 (td, *J* = 7.2, 3.6 Hz, 1H), 7.33 (t, *J* = 7.7 Hz, 1H), 7.25–7.11 (m, 5H), 7.05 (t, *J* = 7.6 Hz, 2H). ^13^C NMR (75 MHz, chloroform-d) δ 150.52 (d, *J* = 9.6 Hz), 136.41 (d, *J* = 7.4 Hz), 136.10 (d, *J* = 4.3 Hz), 133.88 (d, *J* = 2.7 Hz), 130.83 (d, *J* = 10.2 Hz), 130.57, 129.45 (d, *J* = 3.1 Hz), 129.08 (d, *J* = 2.6 Hz), 128.53 (d, *J* = 15.0 Hz), 125.66, 124.99, 124.75, 124.31 (d, *J* = 65.7 Hz), 124.23 (d, *J* = 6.3 Hz), 123.36 (d, *J* = 11.4 Hz), 121.81 (d, *J* = 11.6 Hz), 120.07 (d, *J* = 7.1 Hz). ^31^P NMR (121 MHz, chloroform-d) δ 33.11. HRMS (ESI): m/z calculated for C_18_H_14_O_2_PS^+^ [M+H] ^+^: 325.0447, found 325.0447.

*O*-Ethyl *S*-phenyl phenylphosphonothioate (**4b**). Oily liquid, 89% yield. ^1^H NMR (300 MHz, chloroform-d) δ 7.70–7.59 (m, 2H), 7.55–7.44 (m, 1H), 7.33 (ddd, *J* = 27.5, 9.6, 4.8 Hz, 5H), 7.24–7.17 (m, 2H), 4.55–4.24 (m, 2H), 1.40 (t, *J* = 7.1 Hz, 3H). ^13^C NMR (75 MHz, chloroform-d) δ 135.56 (d, *J* = 4.2 Hz), 132.57 (d, *J* = 3.2 Hz), 131.49 (d, *J* = 10.6 Hz), 130.51, 129.19 (d, *J* = 2.3 Hz), 129.05 (d, *J* = 2.8 Hz), 128.26 (d, *J* = 14.9 Hz), 126.65 (d, *J* = 5.6 Hz), 62.53 (d, *J* = 6.9 Hz), 16.41 (d, *J* = 6.8 Hz). ^31^P NMR (121 MHz, chloroform-d) δ 40.43. HRMS (ESI): m/z calculated for C_14_H_16_O_2_PS^+^[M+H] ^+^: 279.0605, found 279.0604.

*O*, *O*-Diethyl (*S*-phenyl)phosphorothioate (**4c**) [48]. Oily liquid, 63% yield. ^1^H NMR (300 MHz, chloroform-d) δ 7.57 (dd, *J* = 4.9, 2.4 Hz, 2H), 7.39–7.32 (m, 3H), 4.19 (dq, *J* = 14.2, 7.0 Hz, 4H), 1.31 (t, *J* = 7.1 Hz, 6H). ^13^C NMR (75 MHz, chloroform-d) δ 134.60 (d, *J* = 5.2 Hz), 129.41 (d, *J* = 2.2 Hz), 129.06 (d, *J* = 2.9 Hz), 126.62 (d, *J* = 7.2 Hz), 64.13 (d, *J* = 6.2 Hz), 16.07 (d, *J* = 7.2 Hz). ^31^P NMR (121 MHz, chloroform-d) δ 21.69.

*O*, *O*-Disopropyl (*S*-phenyl)phosphorothioate (**4d**) [48]. Oily liquid, 51% yield. ^1^H NMR (300 MHz, chloroform-d) δ 7.69–7.54 (m, 2H), 7.46–7.25 (m, 3H), 4.76 (ddd, *J* = 12.4, 8.4, 6.2 Hz, 2H), 1.33 (d, *J* = 6.2 Hz, 6H), 1.25 (d, *J* = 6.2 Hz, 6H). ^13^C NMR (75 MHz, chloroform-d) δ 134.28 (d, *J* = 5.5 Hz), 129.26 (d, *J* = 2.1 Hz), 128.74 (d, *J* = 2.7 Hz), 127.38 (d, *J* = 7.1 Hz), 73.39 (d, *J* = 6.7 Hz), 23.93 (d, *J* = 4.1 Hz), 23.57 (d, *J* = 5.8 Hz). ^31^P NMR (121 MHz, chloroform-d) δ 19.35.

*O*, *O*-dibutyl (*S*-phenyl) phosphorothioate (**4e**). Oily liquid, 60% yield. ^1^H NMR (300 MHz, chloroform-d) δ 7.63–7.51 (m, 2H), 7.41–7.29 (m, 3H), 4.11 (p, *J* = 7.2 Hz, 4H), 1.68–1.57 (m, 4H), 1.34 (dt, *J* = 14.6, 7.4 Hz, 4H), 0.90 (t, *J* = 7.4 Hz, 6H). ^13^C NMR (75 MHz, chloroform-d) δ 134.53 (d, *J* = 5.3 Hz), 129.35 (d, *J* = 2.2 Hz), 128.97 (d, *J* = 2.8 Hz), 126.68 (d, *J* = 7.1 Hz), 67.82 (d, *J* = 6.6 Hz), 32.17 (d, *J* = 7.2 Hz), 18.70, 13.61. ^31^P NMR (121 MHz, chloroform-d) δ 21.79. HRMS (ESI): m/z calculated for C_14_H_24_O_3_PS^+^ [M+H] ^+^: 303.1168, found 303.1178.

*S*-(*p*-Tolyl) diphenylphosphinothioate (**5a**) [45]. White solid, 92% yield. ^1^H NMR (300 MHz, chloroform-d) δ 7.85 (ddd, *J* = 12.9, 8.0, 1.4 Hz, 4H), 7.52–7.28 (m, 8H), 6.98 (d, *J* = 8.0 Hz, 2H), 2.20 (s, 3H). ^13^C NMR (75 MHz, chloroform-d) δ 139.17 (d, *J* = 2.5 Hz), 135.35 (d, *J* = 3.8 Hz), 132.65, (d, *J* =105.1), 132.27 (d, *J* = 3.0 Hz), 131.64 (d, *J* = 10.3 Hz), 129.98 (d, *J* = 1.9 Hz), 128.52 (d, *J* = 13.1 Hz), 122.26 (d, *J* = 5.2 Hz), 21.19.^31^P NMR (121 MHz, chloroform-d) δ 40.06.

*S*-(4-tert-Buty)phenyl)diphenylphosphinothioate (**5b**). White solid, 97% yield. m.p: 128.2–129 °C. ^1^H NMR (300 MHz, chloroform-d) δ 7.90–7.79 (m, 4H), 7.51–7.34 (m, 8H), 7.21 (d, *J* = 8.4 Hz, 2H), 1.22 (s, 9H). ^13^C NMR (75 MHz, chloroform-d) δ 152.22 (d, *J* = 2.5 Hz), 135.22 (d, *J* = 3.7 Hz), 132.67 (d, *J* = 106.6 Hz), 132.25 (d, *J* = 3.0 Hz), 131.62 (d, *J* = 10.3 Hz), 128.49 (d, *J* = 13.1 Hz), 126.31 (d, *J* = 1.9 Hz), 122.28 (d, *J* = 5.2 Hz), 34.59, 31.15. ^31^P NMR (121 MHz, chloroform-d) δ 40.31. HRMS (ESI): m/z calculated for C_22_H_24_OPS^+^ [M+H] ^+^: 367.1281, found 367.1280.

*S*-(4-Methoxyphenyl) diphenylphosphinothioate (**5c**) [45]. White solid, 80% yield. ^1^H NMR (300 MHz, chloroform-d) δ 7.90–7.78 (m, 4H), 7.50–7.38 (m, 6H), 7.33 (dd, *J* = 8.7, 1.5 Hz, 2H), 6.72 (d, *J* = 8.8 Hz, 2H), 3.69 (s, 3H). ^13^C NMR (75 MHz, chloroform-d) δ 160.52 (d, *J* = 2.3 Hz), 137.15 (d, *J* = 3.5 Hz), 132.69 (d, *J* = 106.2 Hz), 132.33 (d, *J* = 3.1 Hz), 131.72 (d, *J* = 10.2 Hz), 128.60 (d, *J* = 13.0 Hz), 116.06 (d, *J* = 5.3 Hz), 114.88 (d, *J* = 1.9 Hz), 55.37. ^31^P NMR (121 MHz, chloroform-d) δ 40.16.

*S*-(4-Fluorophenyl) diphenylphosphinothioate (**5d**) [49]. Oil liquid, 92% yield. ^1^H NMR (300 MHz, chloroform-d) δ 7.84 (dd, *J* = 12.8, 7.1 Hz, 4H), 7.57–7.36 (m, 8H), 6.89 (t, *J* = 8.6 Hz, 2H). ^13^C NMR (75 MHz, chloroform-d) δ 165.07 (d, *J* = 2.5 Hz), 161.76 (d, *J* = 2.5 Hz), 137.45 (dd, *J* = 8.5, 3.6 Hz), 133.00, 132.47 (d, *J* = 3.0 Hz), 131.61 (d, *J* = 10.3 Hz), 128.64 (d, *J* = 13.2 Hz), 121.22 (dd, *J* = 5.2, 3.4 Hz), 116.36 (dd, *J* = 22.0, 1.9 Hz).^31^P NMR (121 MHz, chloroform-d) δ 41.48 (d, *J* = 4.3 Hz). ^19^F NMR (282 MHz, chloroform-d) δ −111.75 (tq, *J* = 9.2, 5.0 Hz).

*S*-(4-Chlorophenyl) diphenylphosphinothioate (**5e**). White solid, 93% yield, m.p: 105–106.6 °C. ^1^H NMR (300 MHz, chloroform-d) δ 7.79–7.72 (m, 4H), 7.49–7.41 (m, 2H), 7.41–7.33 (m, 4H), 7.30 (dd, *J* = 8.4, 1.5 Hz, 2H), 7.13–7.05 (m, 2H). ^13^C NMR (75 MHz, chloroform-d) δ 136.62 (d, *J* = 3.8 Hz), 135.64 (d, *J* = 2.7 Hz), 132.98, 132.61 (d, *J* = 3.0 Hz), 131.76, 131.59 (d, *J* = 4.5 Hz), 129.42 (d, *J* = 1.9 Hz), 128.74 (d, *J* = 13.2 Hz), 124.78 (d, *J* = 5.3 Hz). ^31^P NMR (121 MHz, chloroform-d) δ 40.35.

*S*-(4-Bromophenyl) diphenylphosphinothioate (**5f**) [36]. Yellow solid, 65% yield. ^1^H NMR (300 MHz, chloroform-d) δ 7.84 (dd, *J* = 12.9, 7.0 Hz, 4H), 7.57–7.41 (m, 6H), 7.32 (s, 4H). ^13^C NMR (75 MHz, chloroform-d) δ 136.78 (d, *J* = 3.8 Hz), 132.58 (d, *J* = 3.1 Hz), 132.32 (d, *J* = 1.8 Hz), 132.19 (d, *J* = 107.1 Hz), 131.63 (d, *J* = 10.3 Hz), 128.70 (d, *J* = 13.2 Hz), 125.44 (d, *J* = 5.2 Hz), 123.84 (d, *J* = 2.8 Hz). ^31^P NMR (121 MHz, chloroform-d) δ 40.24.

*S*-(2-Chlorophenyl) diphenylphosphinothioate (**5g**) [45]. White solid, 97% yield. ^1^H NMR (300 MHz, chloroform-d) δ 7.88 (dd, *J* = 13.0, 7.1 Hz, 4H), 7.69 (t, *J* = 7.5 Hz, 1H), 7.54–7.39 (m, 6H), 7.22 (q, *J* = 7.2 Hz, 1H), 7.02 (t, *J* = 7.5 Hz, 1H), 6.92 (t, *J* = 8.6 Hz, 1H). ^13^C NMR (75 MHz, chloroform-d) δ 164.23 (d, *J* = 4.2 Hz), 160.93 (d, *J* = 4.0 Hz), 137.83 (d, *J* = 3.7 Hz), 133.02, 132.53 (d, *J* = 3.1 Hz), 131.61 (d, *J* = 10.4 Hz), 131.36 (d, *J* = 2.1 Hz), 131.25 (d, *J* = 2.1 Hz), 128.58 (d, *J* = 13.2 Hz), 124.70 (dd, *J* = 3.9, 1.8 Hz), 116.13 (d, *J* = 1.9 Hz), 115.82 (d, *J* = 1.9 Hz), 113.63 (d, *J* = 5.1 Hz), 113.39 (d, *J* = 5.0 Hz). ^31^P NMR (121 MHz, chloroform-d) δ 40.77(d, *J* = 1.9 Hz).

*S*-(3-Chlorophenyl) diphenylphosphinothioate (**5h**) [36]. Yellow solid, 89% yield. ^1^H NMR (300 MHz, chloroform-d) δ 7.85 (dd, *J* = 13.0, 7.1 Hz, 4H), 7.58–7.42 (m, 6H), 7.41–7.37 (m, 2H), 7.22 (d, *J* = 7.7 Hz, 1H), 7.13 (t, *J* = 8.1 Hz, 1H). ^13^C NMR (75 MHz, chloroform-d) δ 134.91 (d, *J* = 4.0 Hz), 134.55 (d, *J* = 2.0 Hz), 133.50 (d, *J* = 3.9 Hz), 132.64 (d, *J* = 3.1 Hz), 132.14 (d, *J* = 107.5 Hz), 131.66 (d, *J* = 10.3 Hz), 130.10 (d, *J* = 1.8 Hz), 129.27 (d, *J* = 2.2 Hz), 128.73 (d, *J* = 13.2 Hz), 128.19 (d, *J* = 5.0 Hz). ^31^P NMR (121 MHz, chloroform-d) δ 40.43.

*S*-(2,4-Dimethylphenyl) diphenylphosphinothioate (**5i**) [50]. Yellow solid, 75% yield. ^1^H NMR (300 MHz, chloroform-d) δ 7.82 (dd, *J* = 12.8, 7.2 Hz, 4H), 7.57–7.36 (m, 6H), 7.30 (d, *J* = 7.9 Hz, 1H), 6.95 (s, 1H), 6.81 (d, *J* = 7.8 Hz, 1H), 2.31 (s, 3H), 2.21 (s, 3H). ^13^C NMR (75 MHz, chloroform-d) δ 142.75 (d, *J* = 3.7 Hz), 139.48 (d, *J* = 2.5 Hz), 136.74 (d, *J* = 3.6 Hz),133.66, 132.24 (d, *J* = 2.8 Hz), 131.66 (d, *J* = 2.1 Hz), 131.52 (d, *J* = 10.2 Hz), 128.46 (d, *J* = 13.0 Hz), 127.35 (d, *J* = 2.1 Hz), 121.54 (d, *J* = 5.4 Hz), 21.26 (d, *J* = 20.9 Hz). ^31^P NMR (121 MHz, chloroform-d) δ 39.66.

*S*-*n*-Propyl diphenylphosphinothioate (**5j**) [45]. Oily liquid, 83% yield. ^1^H NMR (300 MHz, chloroform-d) δ 7.99–7.81 (m, 4H), 7.68–7.41 (m, 6H), 2.78 (dt, *J* = 10.3, 7.3 Hz, 2H), 1.66 (q, *J* = 7.3 Hz, 2H), 0.93 (t, *J* = 7.3 Hz, 3H). ^13^C NMR (75 MHz, chloroform-d) δ 133.55 (d, *J* = 107.0 Hz), 132.30 (d, *J* = 3.1 Hz), 131.53 (d, *J* = 10.4 Hz), 128.71 (d, *J* = 13.0 Hz), 31.30 (d, *J* = 2.3 Hz), 24.13 (d, *J* = 5.0 Hz), 13.35. ^31^P NMR (121 MHz, chloroform-d) δ 41.92.

*S*-*n*-Butyl diphenylphosphinothioate (**5k**) [45]. Oily liquid, 76% yield. ^1^H NMR (300 MHz, chloroform-d) δ 8.11–7.79 (m, 4H), 7.50 (dtt, *J* = 14.1, 6.7, 3.8 Hz, 6H), 2.80 (dt, *J* = 10.1, 7.4 Hz, 2H), 1.61 (p, *J* = 7.3 Hz, 2H), 1.35 (h, *J* = 7.3 Hz, 2H), 0.83 (t, *J* = 7.3 Hz, 3H). ^13^C NMR (75 MHz, chloroform-d) δ 133.53 (d, *J* = 107.0 Hz), 132.31 (d, *J* = 3.0 Hz), 131.54 (d, *J* = 10.4 Hz), 128.71 (d, *J* = 13.1 Hz), 32.66 (d, *J* = 4.9 Hz), 29.07 (d, *J* = 2.2 Hz), 21.81, 13.52. ^31^P NMR (121 MHz, chloroform-d) δ 41.90.

*S*-Benzyl diphenylphosphinothioate (**5l**) [51]. White solid, 98% yield. ^1^H NMR (300 MHz, chloroform-d) δ 7.86 (dd, *J* = 13.0, 7.1 Hz, 4H), 7.59–7.35 (m, 6H), 7.19 (s, 5H), 4.02 (d, *J* = 9.2 Hz, 2H). ^13^C NMR (75 MHz, chloroform-d) δ 136.81 (d, *J* = 5.5 Hz), 133.01 (d, *J* = 107.0 Hz), 132.41 (d, *J* = 3.0 Hz), 131.57 (d, *J* = 10.5 Hz), 128.94 (d, *J* = 19.0 Hz), 128.06 (d, *J* = 85.4 Hz), 33.23 (d, *J* = 2.1 Hz). ^31^P NMR (121 MHz, chloroform-d) δ 41.56.

*S*-(*p*-Tolyl) bis(4-(trifluoromethyl)phenyl)phosphinothioate (**5m**) [52]. White solid, 92% yield. ^1^H NMR (300 MHz, chloroform-d) δ 7.99 (dd, *J* = 12.5, 8.0 Hz, 4H), 7.72 (dd, *J* = 8.4, 2.9 Hz, 4H), 7.33 (dd, *J* = 8.2, 1.8 Hz, 2H), 7.05 (d, *J* = 7.8 Hz, 2H), 2.27 (s, 3H). ^13^C NMR (75 MHz, chloroform-d) δ 140.08 (d, *J* = 2.6 Hz), 136.44 (d, *J* = 105.0 Hz), 135.51 (d, *J* = 3.9 Hz), 134.54 (d, *J* = 3.2 Hz), 134.11 (d, *J* = 3.2 Hz), 132.14 (d, *J* = 10.6 Hz), 130.40 (d, *J* = 2.1 Hz), 125.66 (dq, *J* = 13.3, 3.7 Hz), 123.46 (d, *J* = 272.0 Hz), 21.23. ^31^P NMR (121 MHz, chloroform-d) δ 36.46. ^19^F NMR (282 MHz, chloroform-d) δ −62.42.

*S*-(*p*-Toyl)bis(3,5-di-tert-butylphenyl)phosphinothioate (**5n**) [50]. White solid, 90% yield. ^1^H NMR (300 MHz, chloroform-d) δ 7.70 (s, 2H), 7.66 (s, 2H), 7.55 (s, 2H), 7.28 (d, *J* = 7.3 Hz, 2H), 6.96 (d, *J* = 7.9 Hz, 2H), 2.22 (s, 3H), 1.30 (s, 36H). ^13^C NMR (75 MHz, chloroform-d) δ 150.80 (d, *J* = 12.8 Hz), 138.64 (d, *J* = 2.5 Hz), 135.36 (d, *J* = 3.7 Hz), 131.63 (d, *J* = 105.5 Hz), 129.66 (d, *J* = 1.9 Hz), 126.15 (d, *J* = 3.1 Hz), 125.84 (d, *J* = 10.8 Hz), 123.31 (d, *J* = 5.1 Hz), 35.01, 31.31, 21.09. ^31^P NMR (121 MHz, chloroform-d) δ 43.11.

*S*-(*p*-Tolyl)di(naphthalen-1-yl)phosphinothioate (**5o**) [50]. White solid, 98% yield. ^1^H NMR (300 MHz, chloroform-d) δ 8.84–8.72 (m, 2H), 8.00–7.81 (m, 4H), 7.76–7.68 (m, 2H), 7.41–7.32 (m, 4H), 7.28 (td, *J* = 8.1, 2.3 Hz, 4H), 6.83 (d, *J* = 7.9 Hz, 2H), 2.08 (s, 3H). ^13^C NMR (75 MHz, chloroform-d) δ 139.06 (d, *J* = 2.5 Hz), 135.33 (d, *J* = 3.9 Hz), 133.91 (d, *J* = 10.2 Hz), 133.73 (d, *J* = 3.3 Hz), 133.49 (d, *J* = 11.6 Hz), 133.25 (d, *J* = 9.3 Hz), 129.89 (d, *J* = 1.9 Hz), 129.02 (d, *J* = 102.5 Hz), 128.85 (d, *J* = 1.3 Hz), 127.33, 127.21 (d, *J* = 4.9 Hz), 126.49, 124.38 (d, *J* = 15.4 Hz), 123.06 (d, *J* = 5.2 Hz), 21.17. ^31^P NMR (121 MHz, chloroform-d) δ 44.22.

*S*-(*p*-Tolyl) bis(4-methoxyphenyl)phosphinothioate (**5p**) [47]. Oily liquid, 95% yield. ^1^H NMR (300 MHz, chloroform-d) δ 7.66 (dd, *J* = 12.3, 8.5 Hz, 4H), 7.23 (d, *J* = 7.4 Hz, 2H), 6.89 (d, *J* = 7.9 Hz, 2H), 6.82 (dd, *J* = 8.8, 2.8 Hz, 4H), 3.69 (s, 6H), 2.13 (s, 3H). ^13^C NMR (75 MHz, chloroform-d) δ 162.57 (d, *J* = 3.1 Hz), 138.84 (d, *J* = 2.4 Hz), 135.08 (d, *J* = 3.8 Hz), 133.48 (d, *J* = 11.8 Hz), 129.85 (d, *J* = 1.8 Hz), 124.80, 123.28, 123.04 (d, *J* = 5.2 Hz), 113.95 (d, *J* = 14.3 Hz), 55.30, 21.11. ^31^P NMR (121 MHz, chloroform-d) δ 40.20.

*S*-Benzyl *O*-ethyl phenylphosphonothioate (**5q**) [45]. Oily liquid, 54% yield. ^1^H NMR (300 MHz, chloroform-d) δ 7.83 (ddd, *J* = 13.9, 6.9, 1.5 Hz, 2H), 7.55–7.40 (m, 3H), 7.21 (d, *J* = 2.8 Hz, 5H), 4.34–4.08 (m, 2H), 4.04–3.85 (m, 2H), 1.33 (t, *J* = 7.0 Hz, 3H). ^13^C NMR (75 MHz, chloroform-d) δ 137.21 (d, *J* = 5.2 Hz), 132.52 (d, *J* = 3.3 Hz), 132.46 (d, *J* = 150.5 Hz), 131.18 (d, *J* = 10.9 Hz), 128.64 (d, *J* = 33.1 Hz), 128.59 (d, *J* = 3.7 Hz), 127.45, 62.22 (d, *J* = 6.8 Hz), 34.51 (d, *J* = 2.7 Hz), 16.30 (d, *J* = 6.9 Hz). ^31^P NMR (121 MHz, chloroform-d) δ 42.54.

*Se*-Phenyl diphenylphosphinoselenoate (**5r**) [53]. Yellow solid, 74% yield. ^1^H NMR (300 MHz, chloroform-d) δ 7.87–7.76 (m, 4H), 7.55–7.38 (m, 8H), 7.29–7.11 (m, 3H). ^13^C NMR (75 MHz, chloroform-d) δ 136.40 (d, *J* = 3.3 Hz), 134.19, 132.89, 132.37 (d, *J* = 3.1 Hz), 131.43 (d, *J* = 10.6 Hz), 129.35 (d, *J* = 1.8 Hz), 128.85 (d, *J* = 2.0 Hz), 128.61 (d, *J* = 13.2 Hz), 123.86 (d, *J* = 5.8 Hz). ^31^P NMR (121 MHz, chloroform-d) δ 30.15.

*Se*-Benzyl diphenylphosphinoselenoate (**5s**) [54]. Yellow solid, 45% yield. ^1^H NMR (300 MHz, chloroform-d) δ 7.85 (ddd, *J* = 13.3, 8.2, 1.6 Hz, 4H), 7.60–7.41 (m, 6H), 7.17 (s, 5H), 4.07 (d, *J* = 8.3 Hz, 2H). ^13^C NMR (75 MHz, chloroform-d) δ 137.68 (d, *J* = 4.3 Hz), 134.09 (d, *J* = 97.5 Hz), 132.42 (d, *J* = 3.1 Hz), 131.43 (d, *J* = 10.9 Hz), 129.03 (d, *J* = 25.0 Hz), 128.68 (d, *J* = 1.3 Hz), 127.29, 28.43 (d, *J* = 2.5 Hz). ^31^P NMR (121 MHz, chloroform-d) δ 38.94.

*Se*-Ethyl diphenylphosphinoselenoate (**5t**) [54]. Yellow solid, 86% yield. ^1^H NMR (300 MHz, chloroform-d) δ 7.89 (ddt, *J* = 13.2, 6.4, 1.7 Hz, 4H), 7.59–7.39 (m, 6H), 2.84 (dq, *J* = 10.1, 7.5 Hz, 2H), 1.39 (t, *J* = 7.5 Hz, 3H). ^13^C NMR (75 MHz, chloroform-d) δ 134.41 (d, *J* = 97.5 Hz), 132.26 (d, *J* = 3.2 Hz), 131.27 (d, *J* = 10.7 Hz), 128.66 (d, *J* = 13.1 Hz), 19.48 (d, *J* = 2.8 Hz), 16.64 (d, *J* = 4.0 Hz). ^31^P NMR (121 MHz, chloroform-d) δ 38.53.

## 4. Conclusions

In summary, we have developed an additive- and solvent-free direct coupling of disulfides and HP(O) species, providing access to phosphinothioates and thiophosphates with good to excellent yields. This method was found to be tolerant of various functional groups on HP(O) species, that is to say, almost no significant substituent effect was observed. In addition, the transformation is also suitable for diselenide substrates.

## Figures and Tables

**Table 1:** Optimization of the reaction conditions *^a^*.

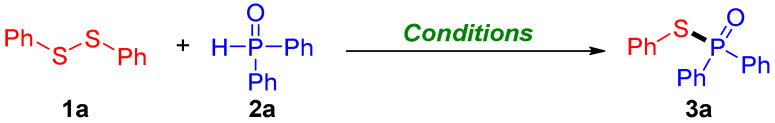					
Entry	1a:2a	Solvent	Time	Temp (°C)	Yield *^b^* (%)
1	1:1	THF	3 h	rt	75
2	1:1	MeOH	3 h	rt	25
3	1:1	DCM	3 h	rt	22
4	1:1	MeCN	3 h	rt	25
5	1:1	EtOAc	3 h	rt	61
6	1:1	1,4-dioxane	3 h	rt	54
7	1:1	H_2_O	3 h	rt	98
8	1:1	DMF	3 h	rt	97
9	1:1	-	3 h	rt	>99 (90) *^c^*
10	1:1	-	1 h	rt	96
11	1:2	-	3 h	rt	42
12	2:1	-	3 h	rt	97
13	1:1	-	3 h	50	>99
14	1:1	-	5 min	50	96
15	1:1	-	10 min	50	>99
16	1:1	-	10 min	40	97

*^a^* Reaction conditions: **1a** (0.3 mmol), **2a** (0.3 mmol), in the air. *^b^* NMR yield. *^c^* Isolated yield.

## Data Availability

The data supporting this article have been included as part of the Supplementary Information.

## Supplementary Materials

The following supporting information can be downloaded at https://www.mdpi.com/article/10.3390/molecules30102097/s1.
