# Supplementary material for: Metal- and Solvent-Free Synthesis of Phosphinothioates, Phosphonothioates, Phosphorothioates, and Related Selenium Derivatives: An Efficient Green Protocol
Source: Molecules. 2025 May 9;30(10):2097. doi: 10.3390/molecules30102097 (PMC12114534; doi:10.3390/molecules30102097)
Supplement: Supplementary file 1 [file molecules-30-02097-s001.zip › molecules-3592354-supplementary.pdf]

Copies of ^{1}H NMR, ^{13}C NMR, ^{31}P NMR and ^{19}F NMR spectra

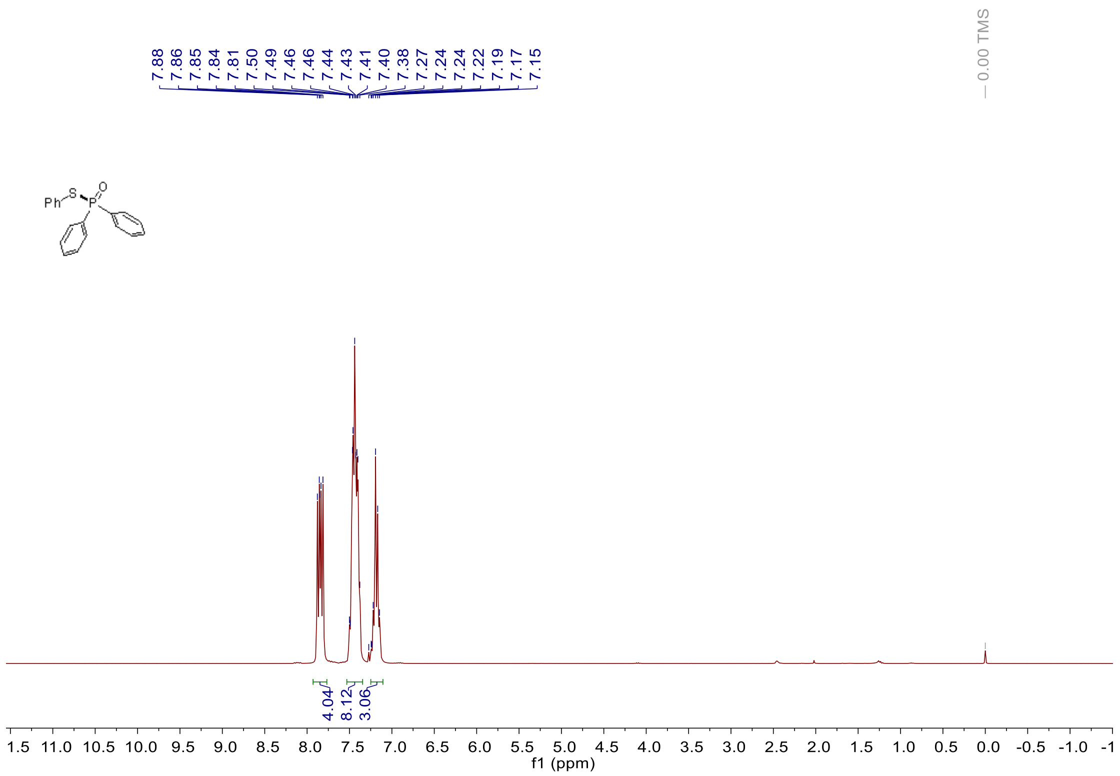

Figure S1. ^{1}H NMR spectra in $CDCl_3$ for Compound **3a**

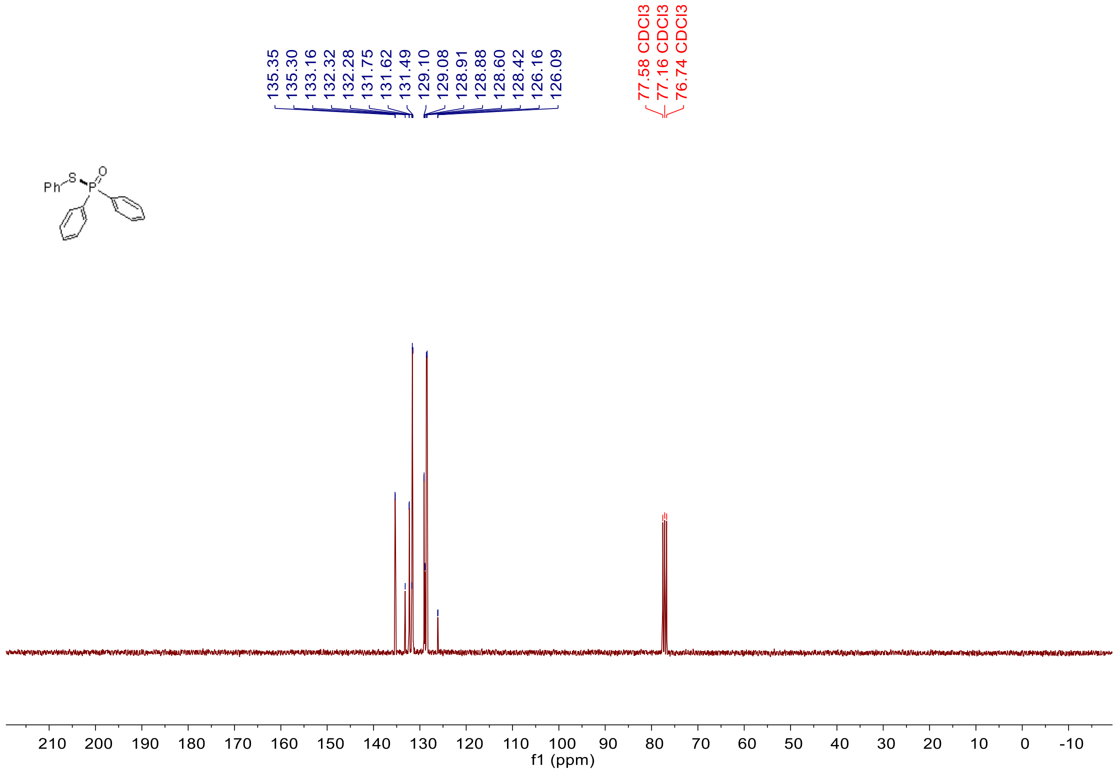

Figure S2. ^{13}C NMR spectra in $CDCl_3$ for Compound **3a**

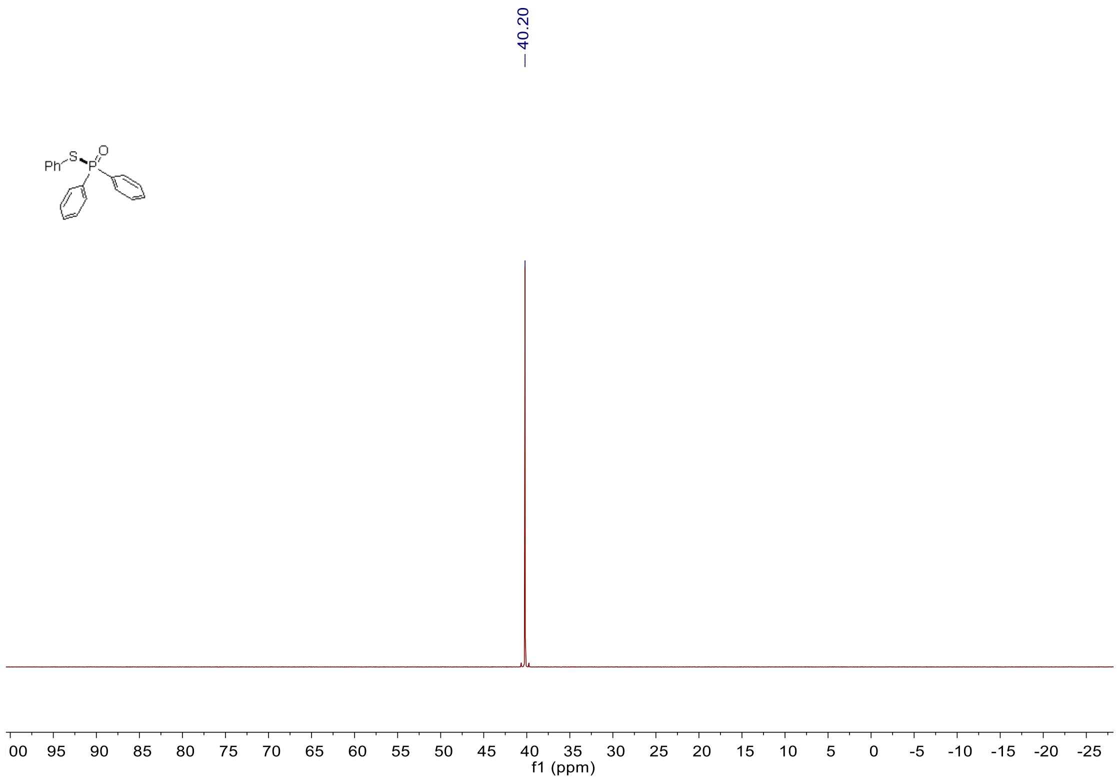

Figure S3. ^{31}P NMR spectra in $CDCl_3$ for compound **3a**

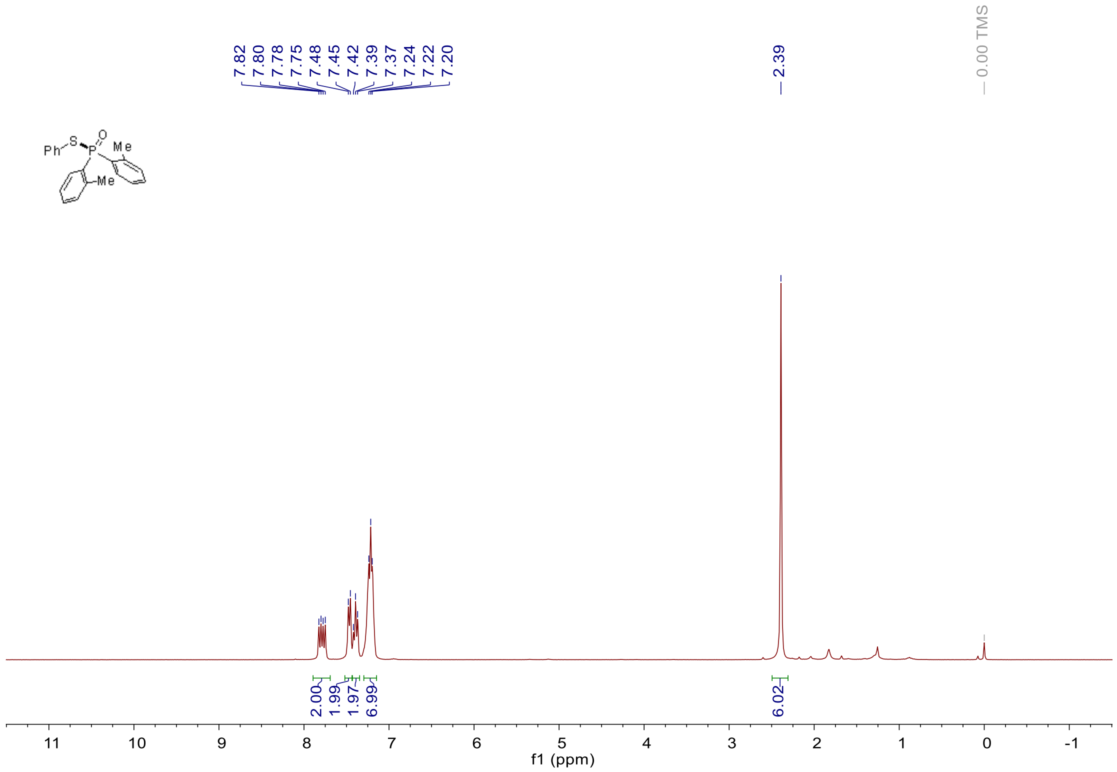

Figure S4. ^{1}H NMR spectra in $CDCl_3$ for Compound **3b**

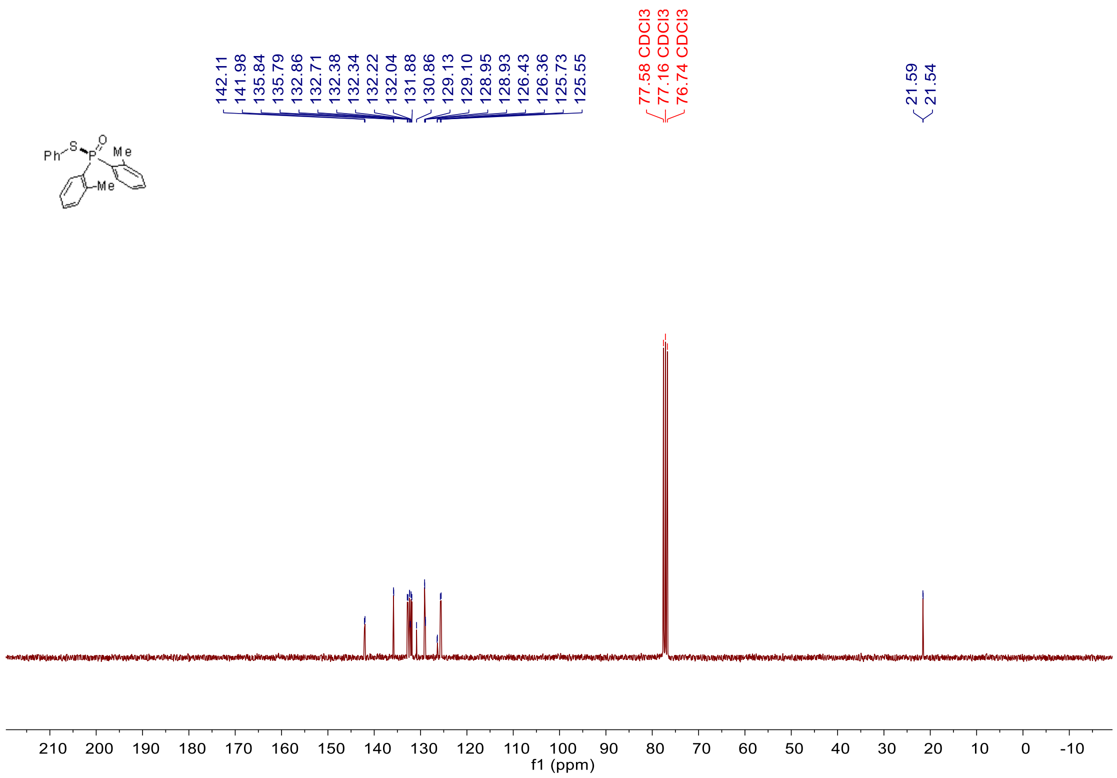

Figure S5. ^{13}C NMR spectra in $CDCl_3$ for Compound **3b**

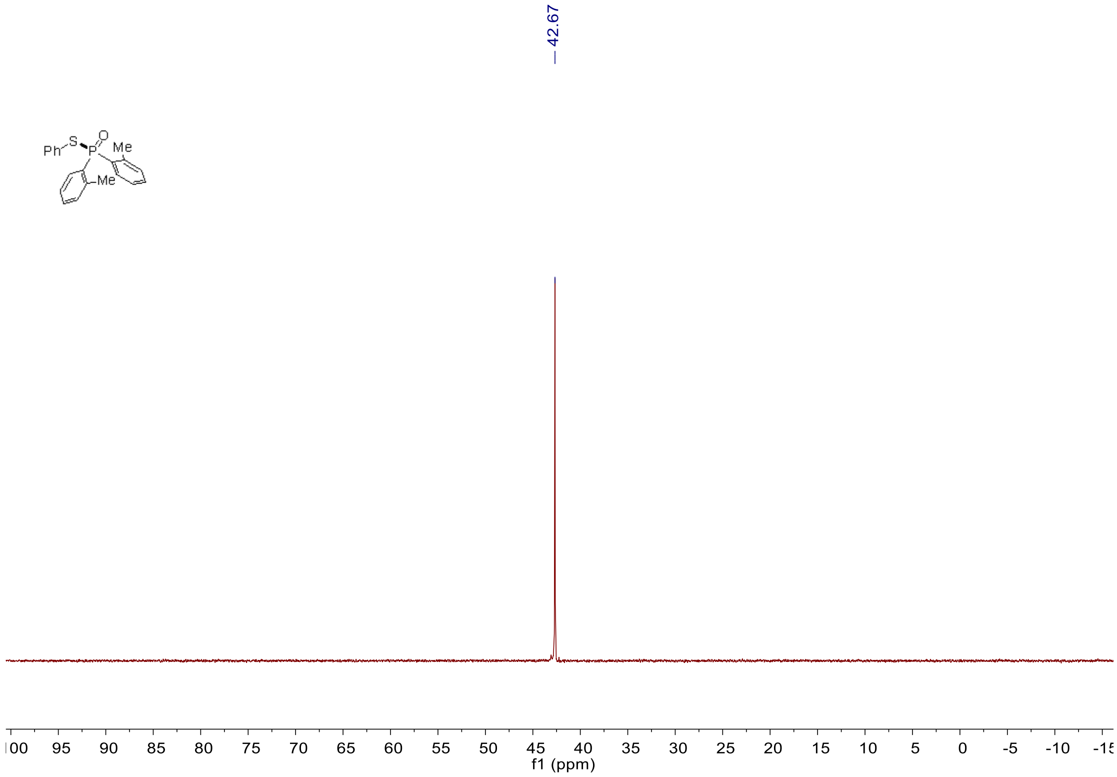

Figure S6. ^{31}P NMR spectra in $CDCl_3$ for Compound **3b**

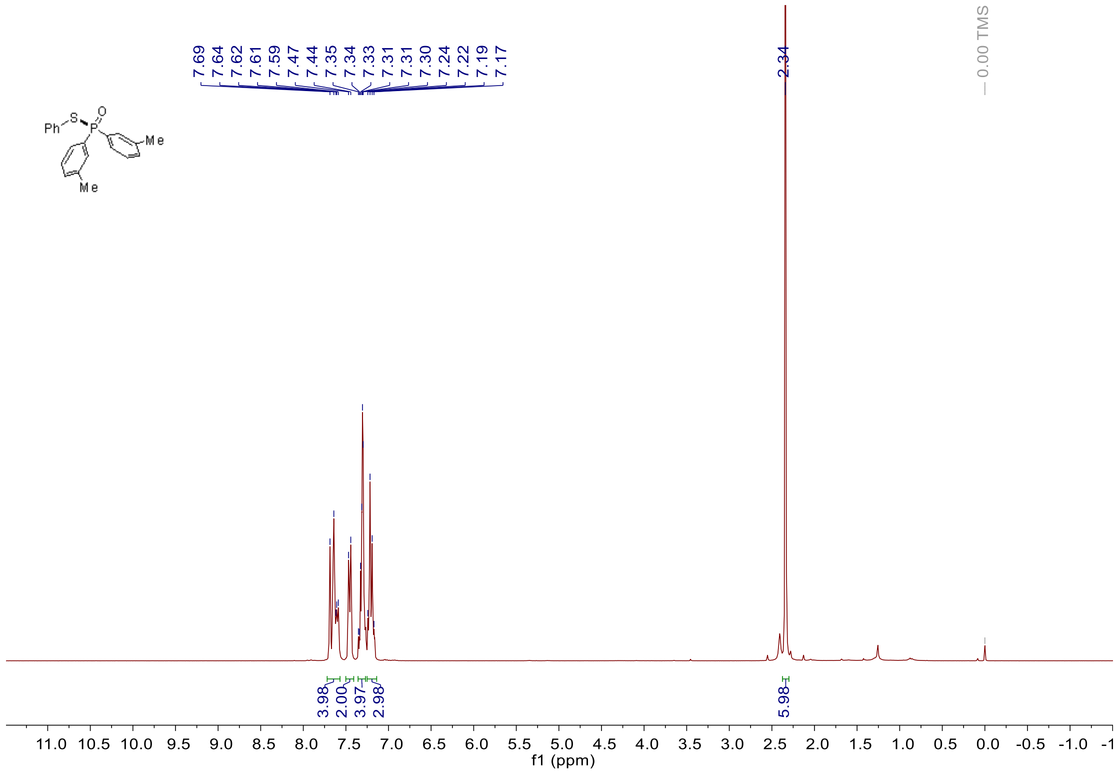

Figure S7. ^{1}H NMR spectra in $CDCl_3$ for Compound **3c**

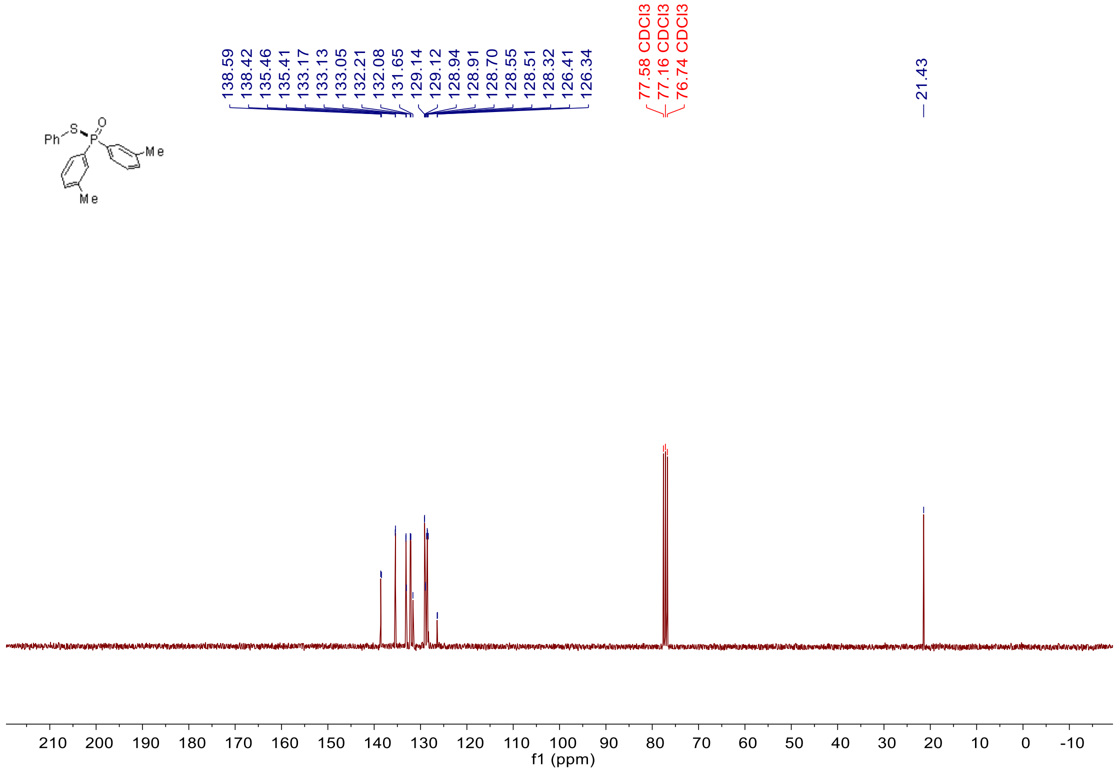

Figure S8. ^{13}C NMR spectra in $CDCl_3$ for Compound **3c**

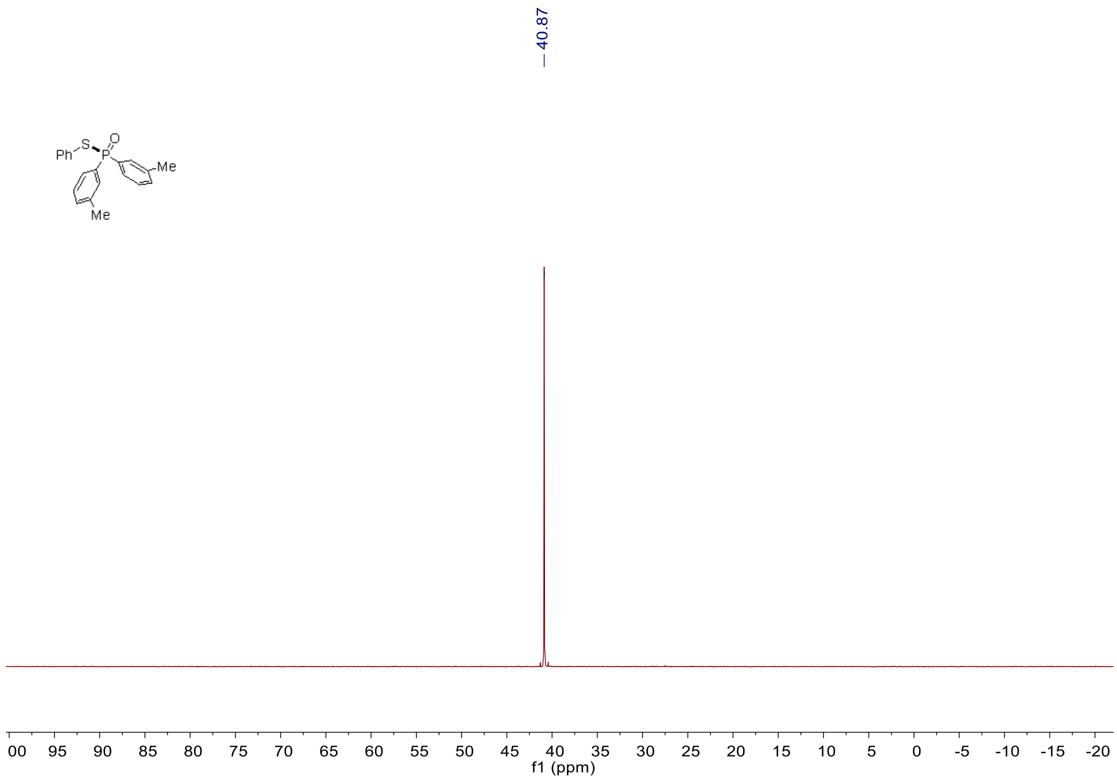

Figure S9. ^{31}P NMR spectra in $CDCl_3$ for Compound **3c**

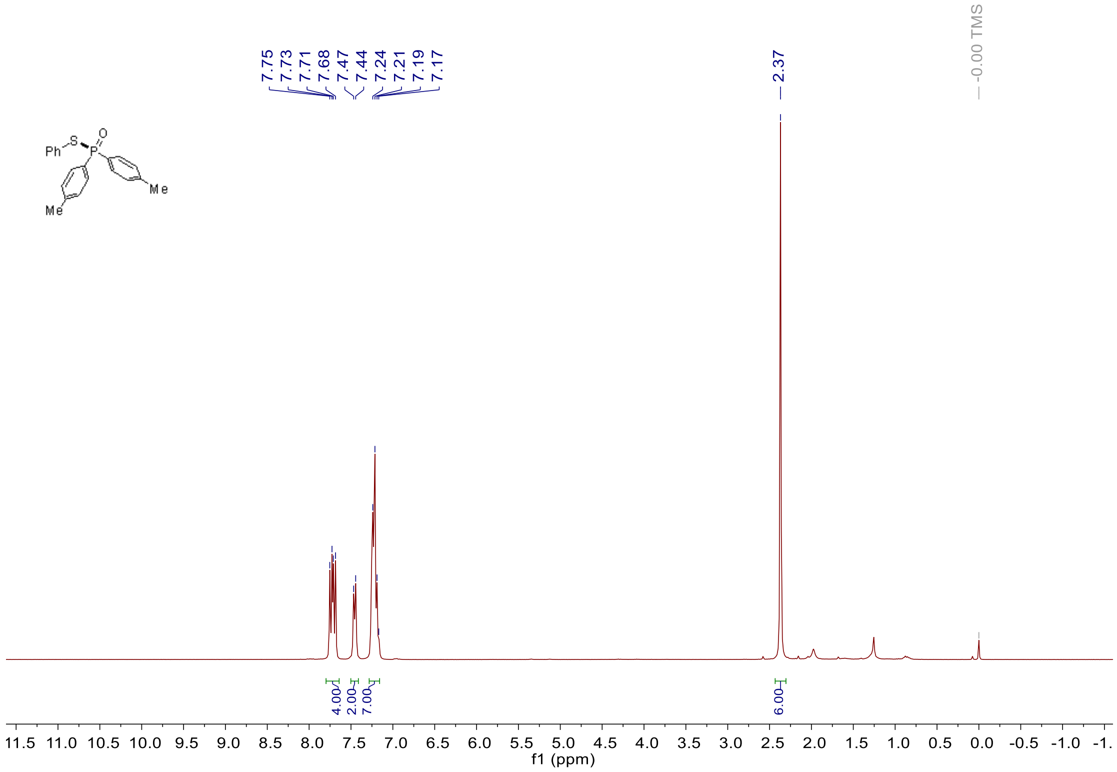

Figure S10. ^{1}H NMR spectra in $CDCl_3$ for Compound **3d**

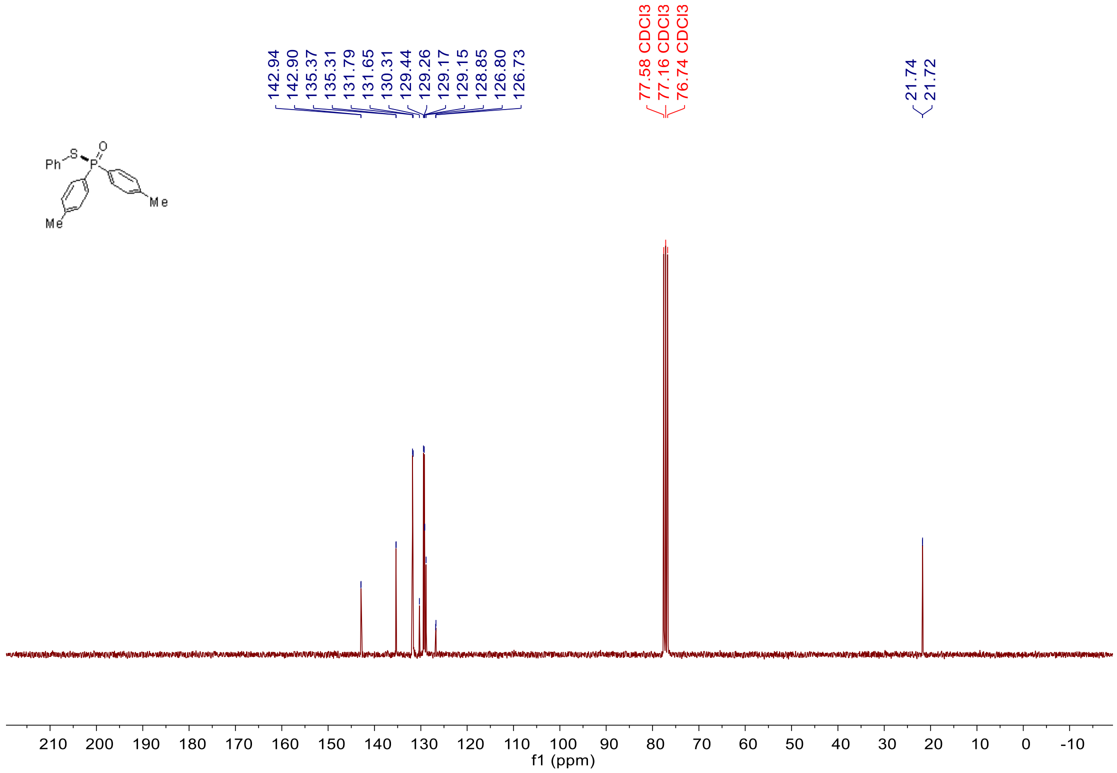

Figure S11. ^{13}C NMR spectra in $CDCl_3$ for Compound **3d**

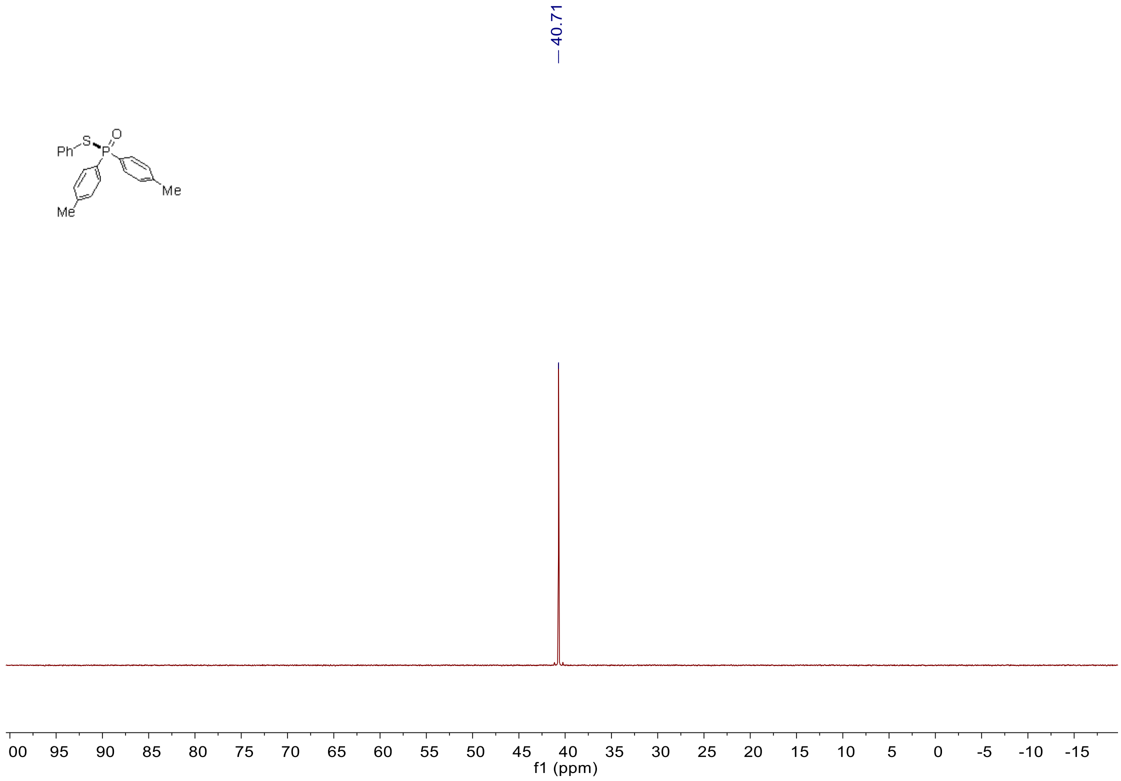

Figure S12. ^{31}P NMR spectra in $CDCl_3$ for Compound **3d**

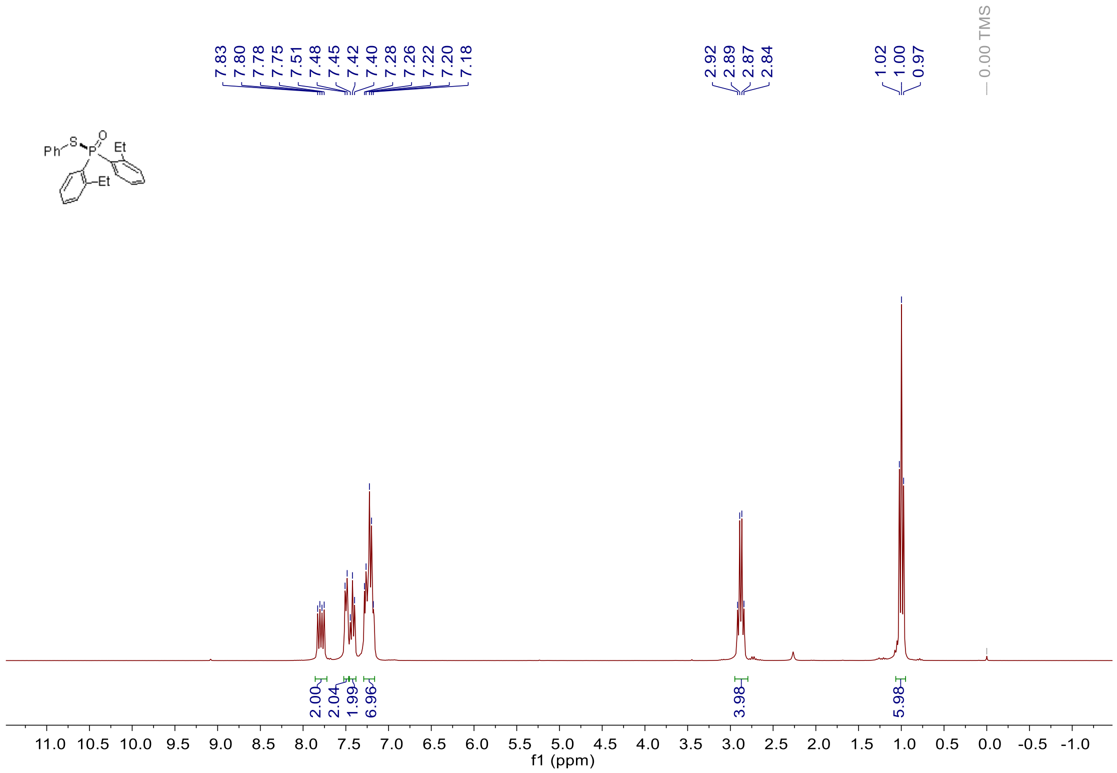

Figure S13. ^{1}H NMR spectra in $CDCl_3$ for Compound **3e**

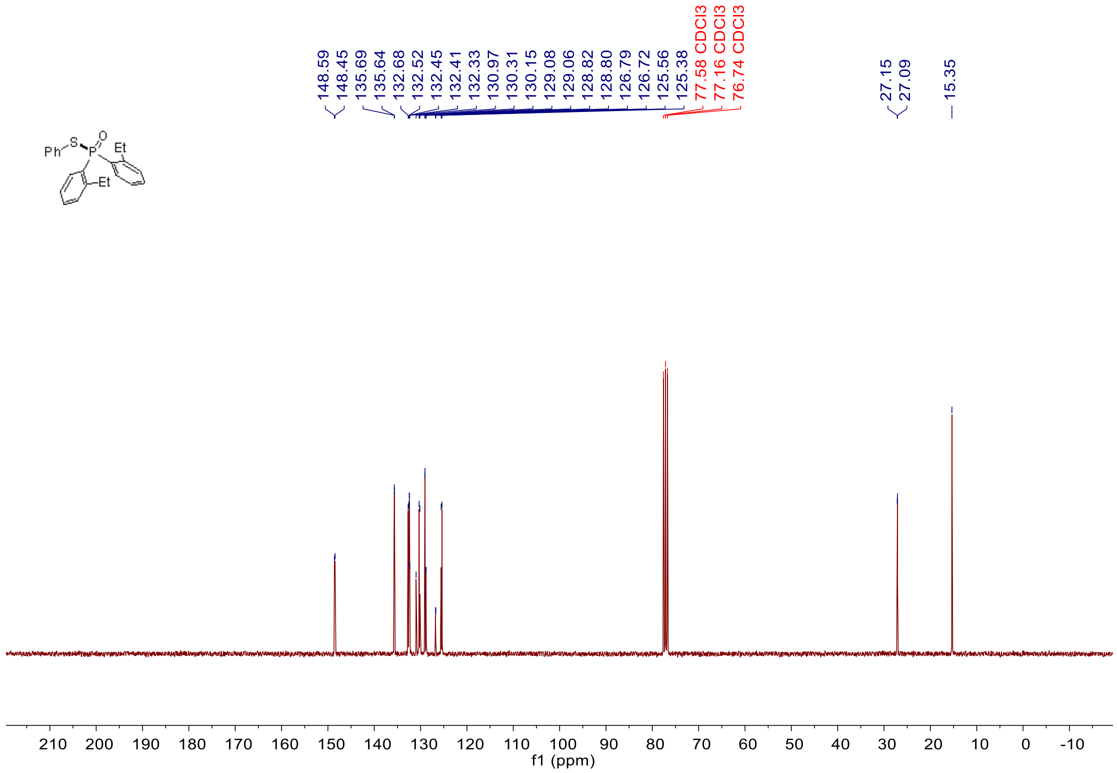

Figure S14. ^{13}C NMR spectra in $CDCl_3$ for Compound **3e**

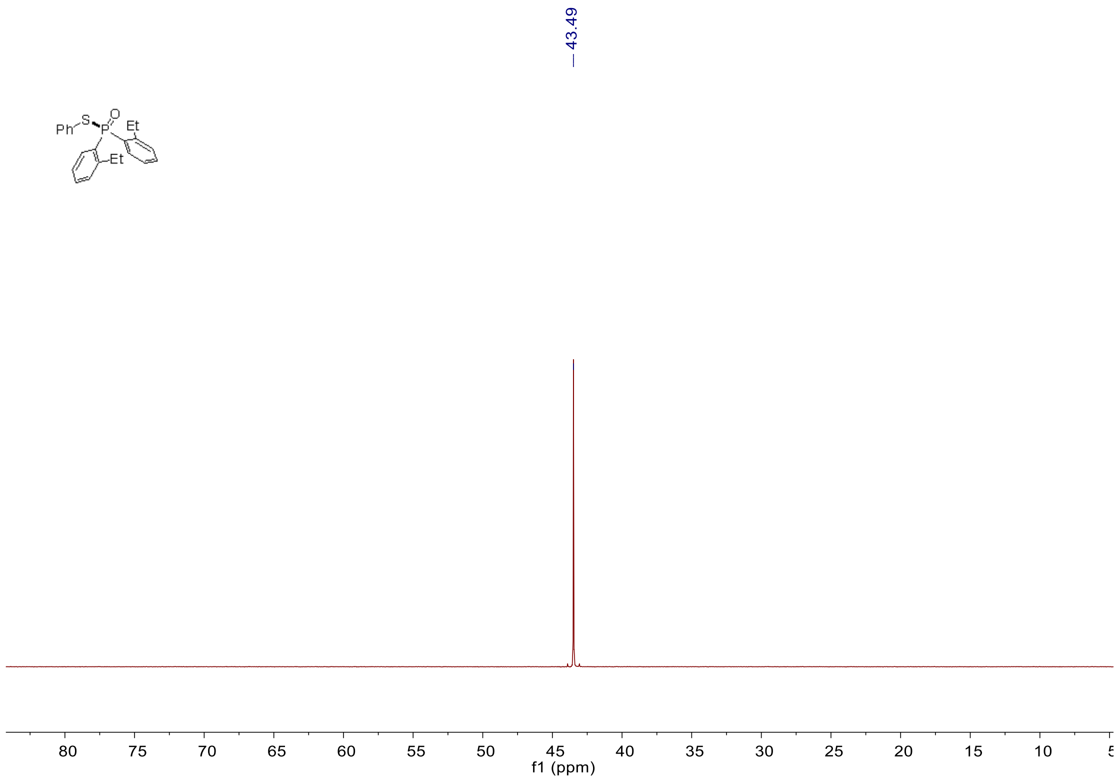

Figure S15. ^{31}P NMR spectra in $CDCl_3$ for Compound **3e**

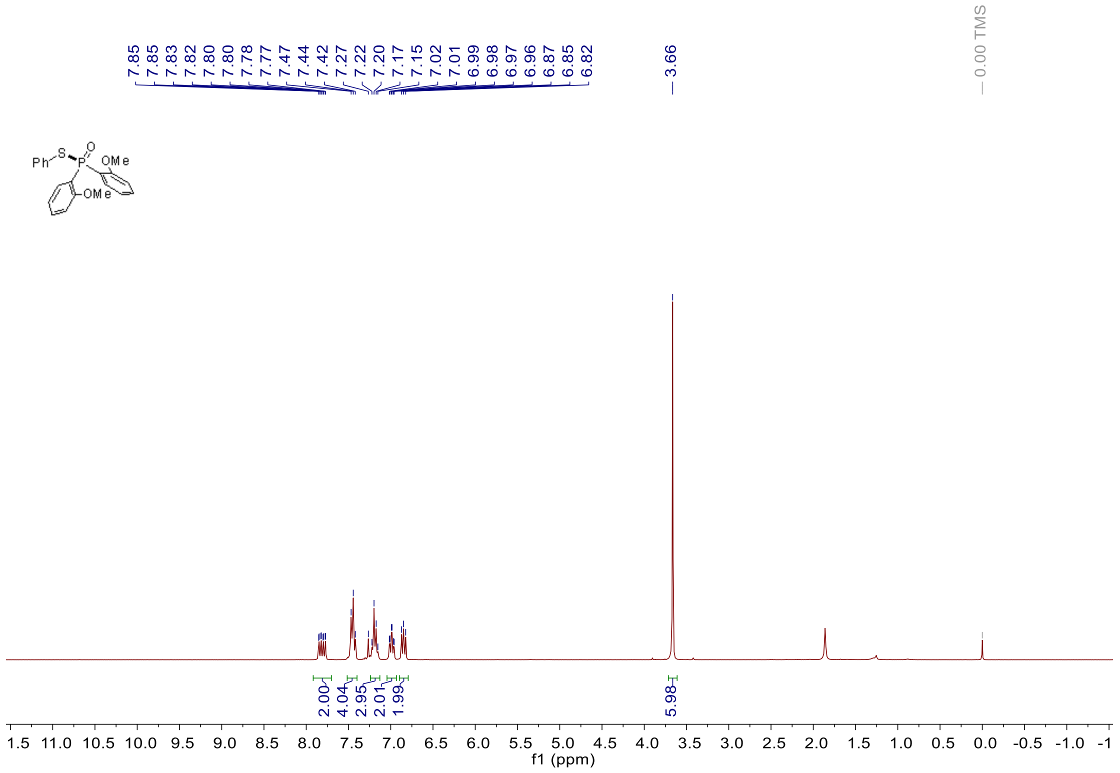

Figure S16. ^{1}H NMR spectra in $CDCl_3$ for Compound **3f**

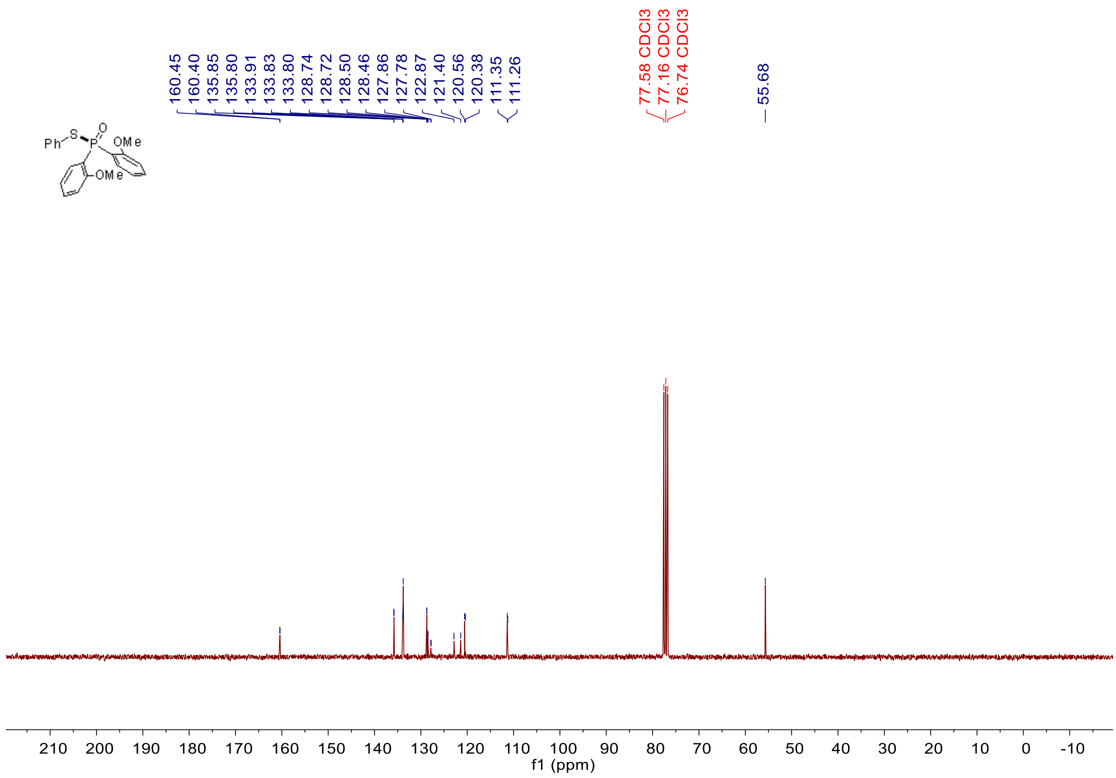

Figure S17. ^{13}C NMR spectra in $CDCl_3$ for Compound **3f**

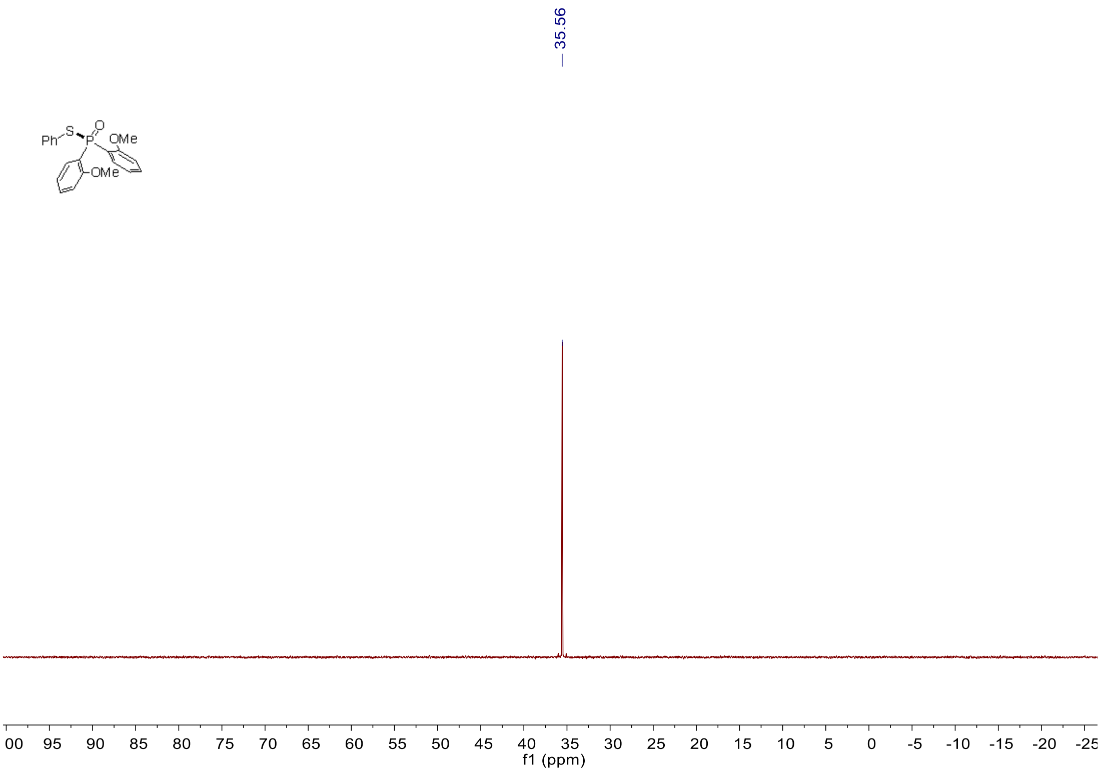

Figure S18. ^{31}P NMR spectra in $CDCl_3$ for Compound **3f**

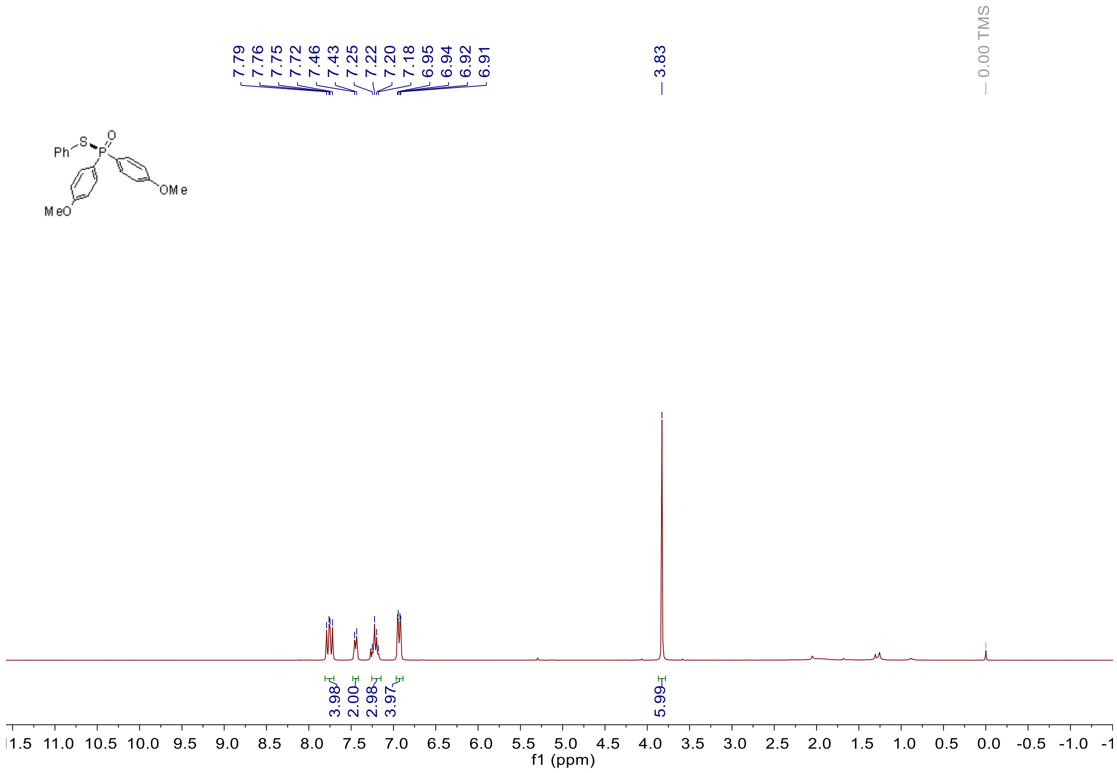

Figure S19. ^{1}H NMR spectra in $CDCl_3$ for Compound **3g**

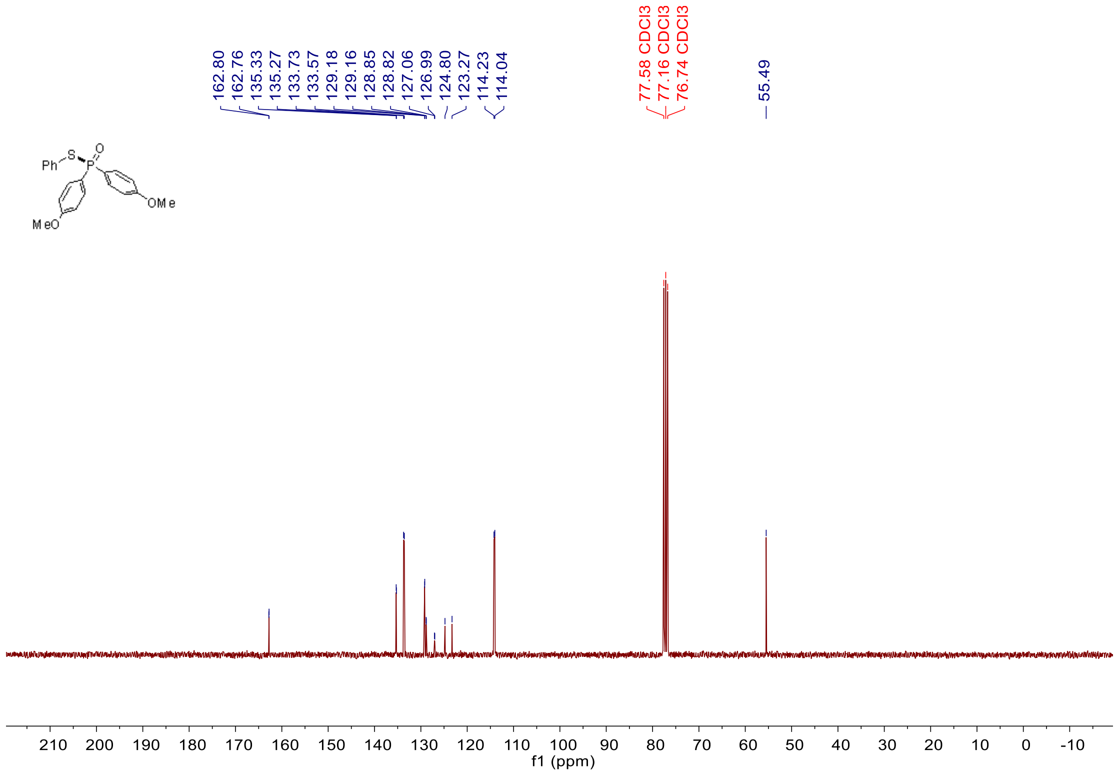

Figure S20. ^{13}C NMR spectra in $CDCl_3$ for Compound **3g**

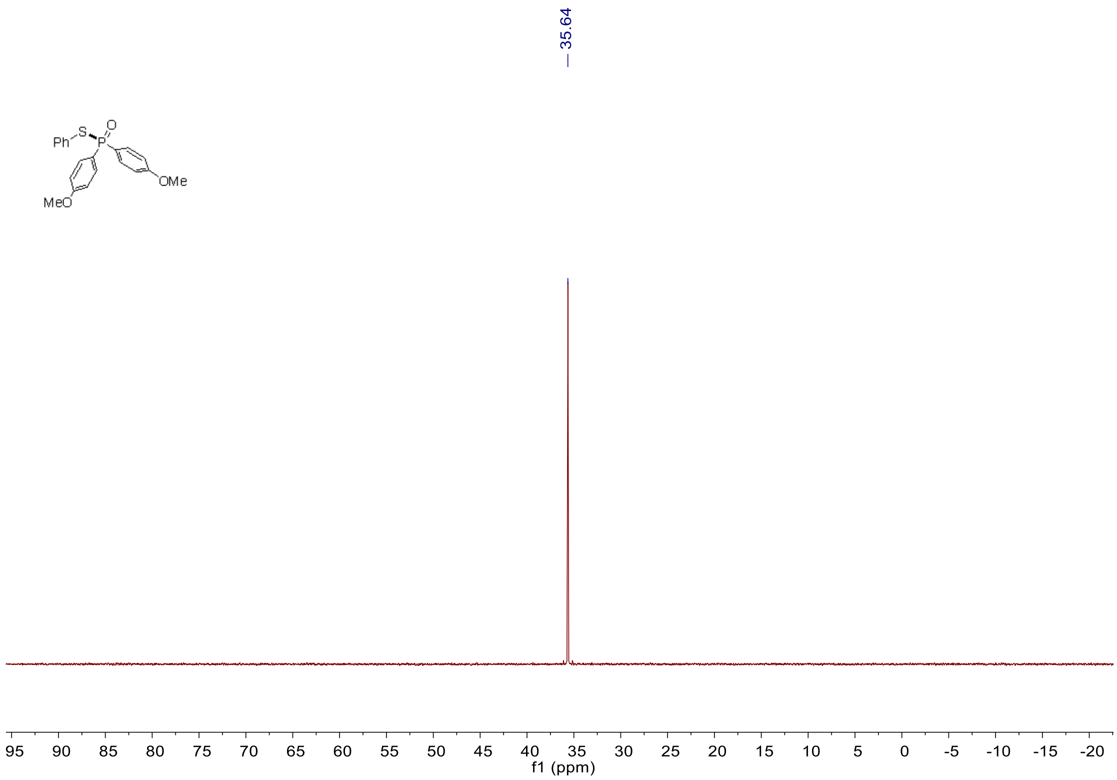

Figure S21. ^{31}P NMR spectra in $CDCl_3$ for Compound **3g**

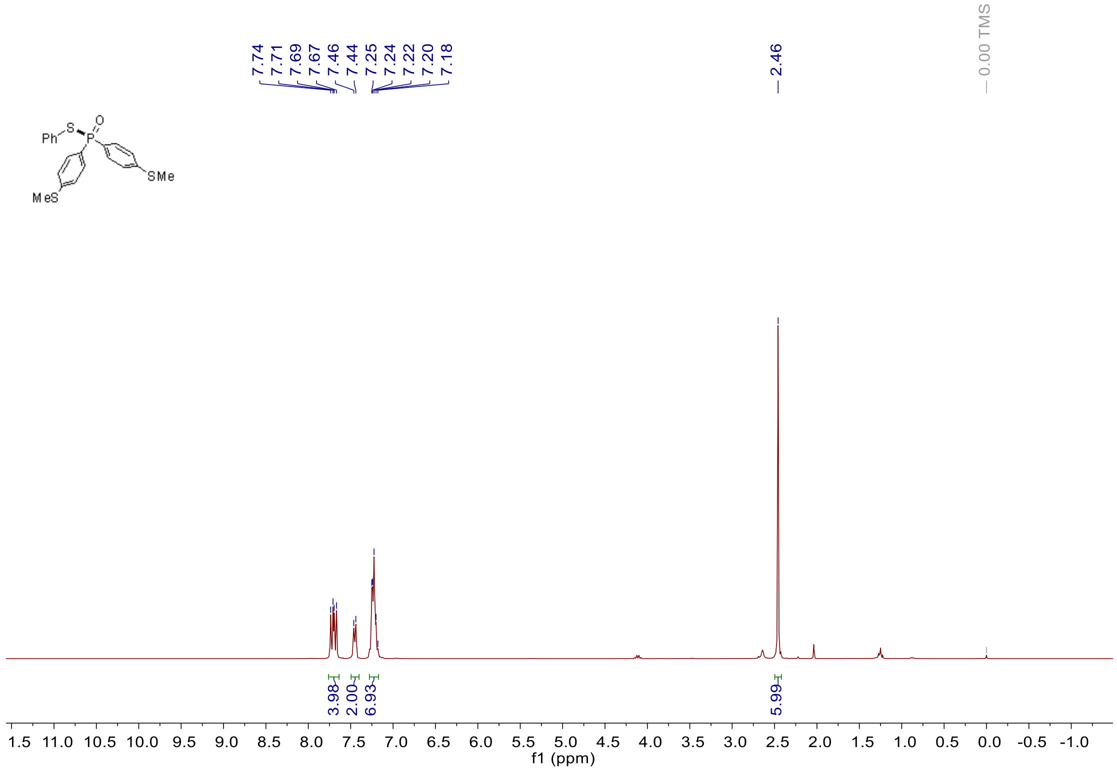

Figure S22. ^{1}H NMR spectra in $CDCl_3$ for Compound **3h**

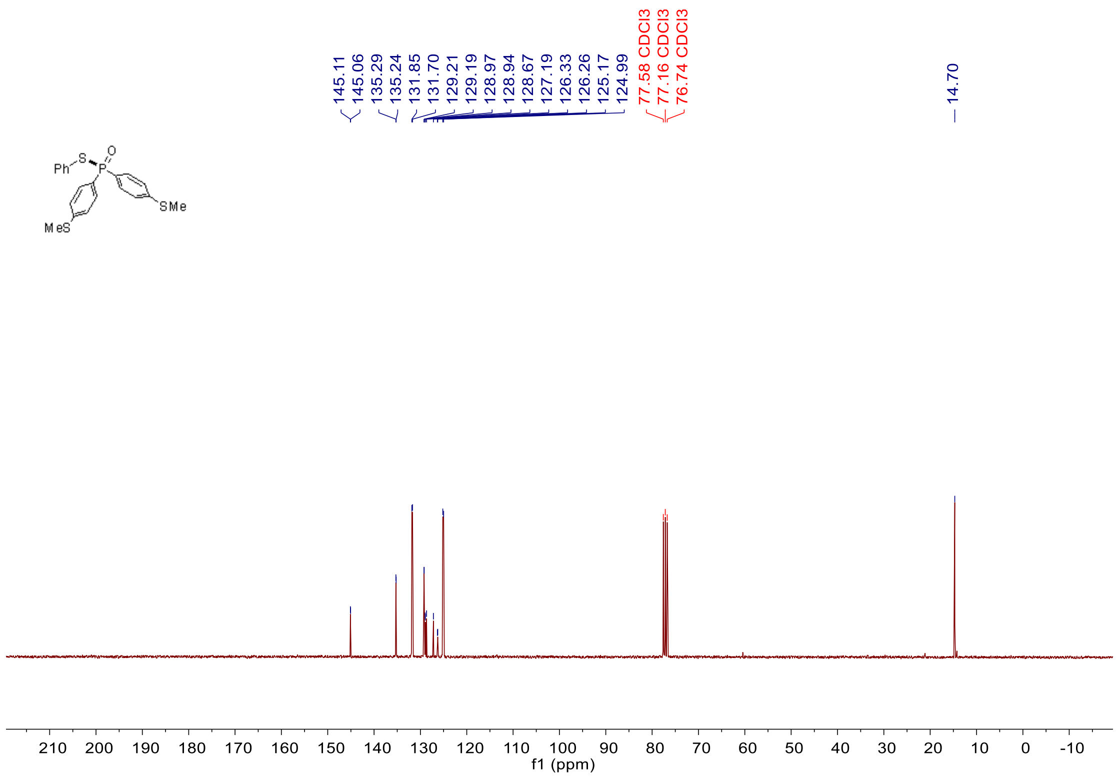

Figure S23. ^{13}C NMR spectra in $CDCl_3$ for Compound **3h**

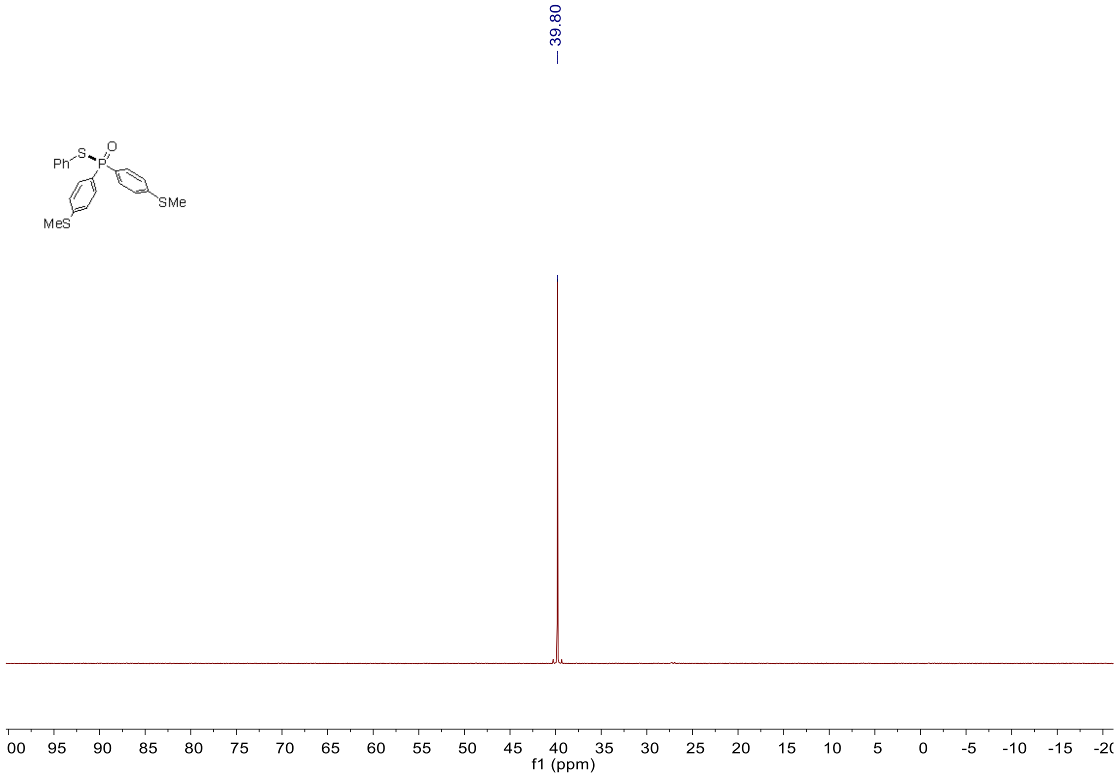

Figure S24. ^{31}P NMR spectra in $CDCl_3$ for Compound **3h**

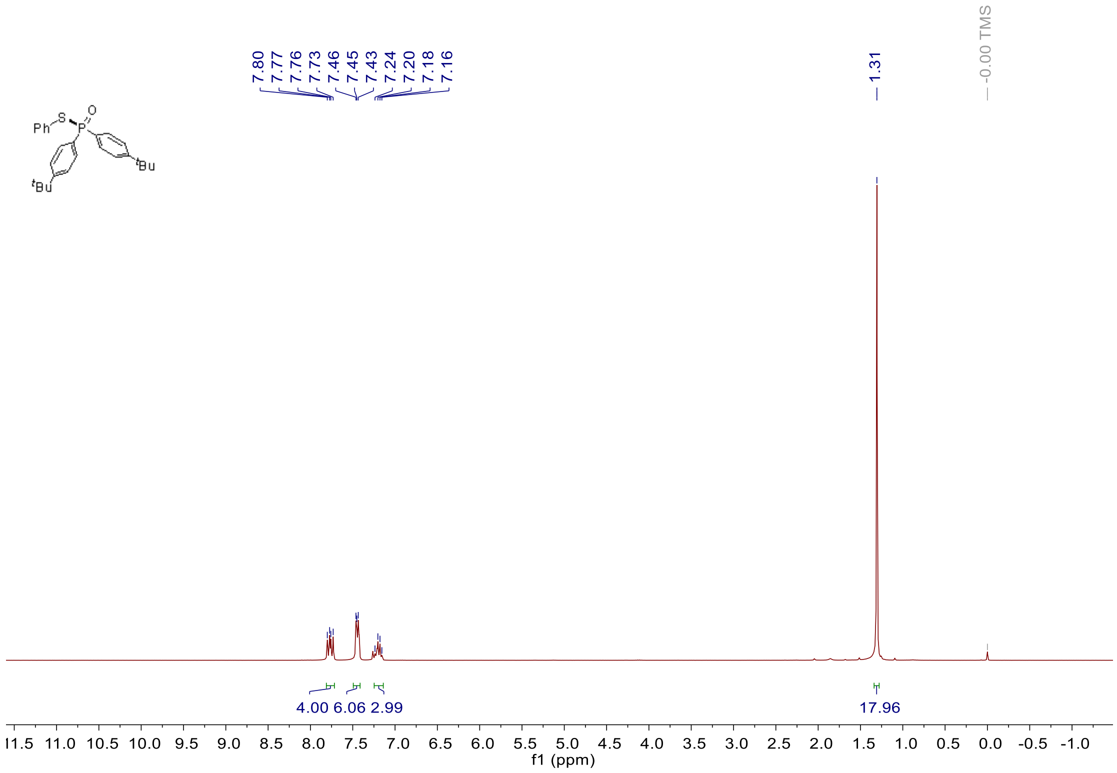

Figure S25. ^{1}H NMR spectra in $CDCl_3$ for Compound **3i**

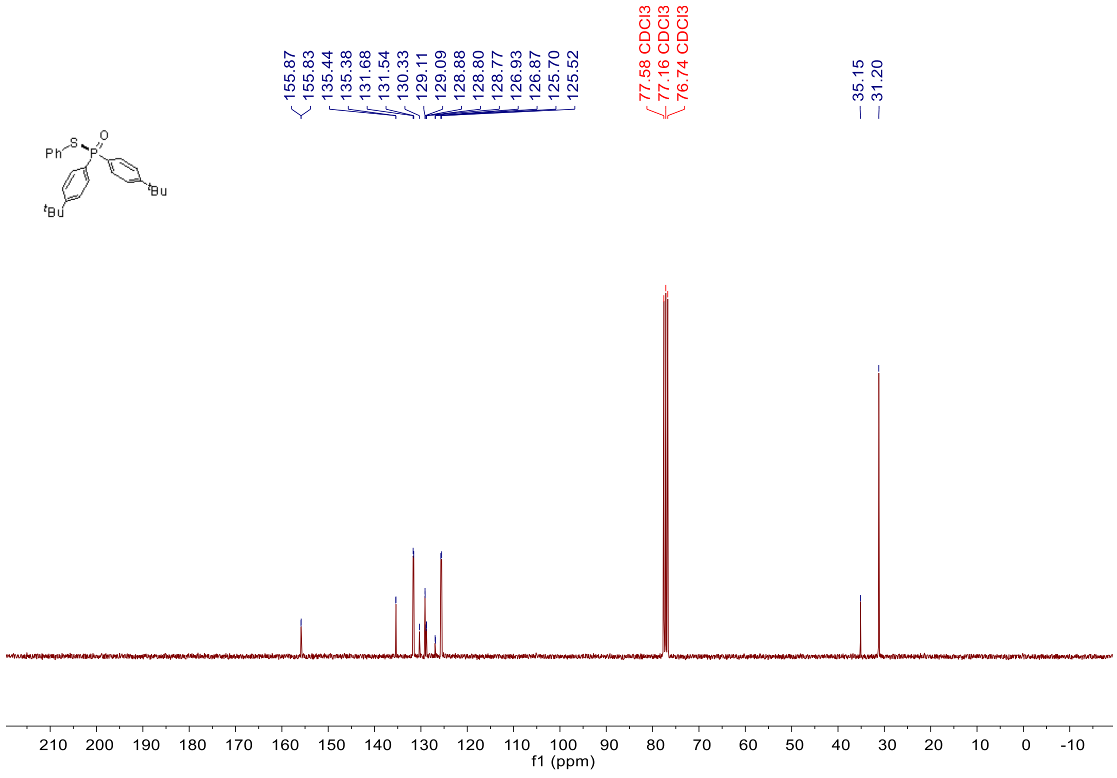

Figure S26. ^{13}C NMR spectra in $CDCl_3$ for Compound **3i**

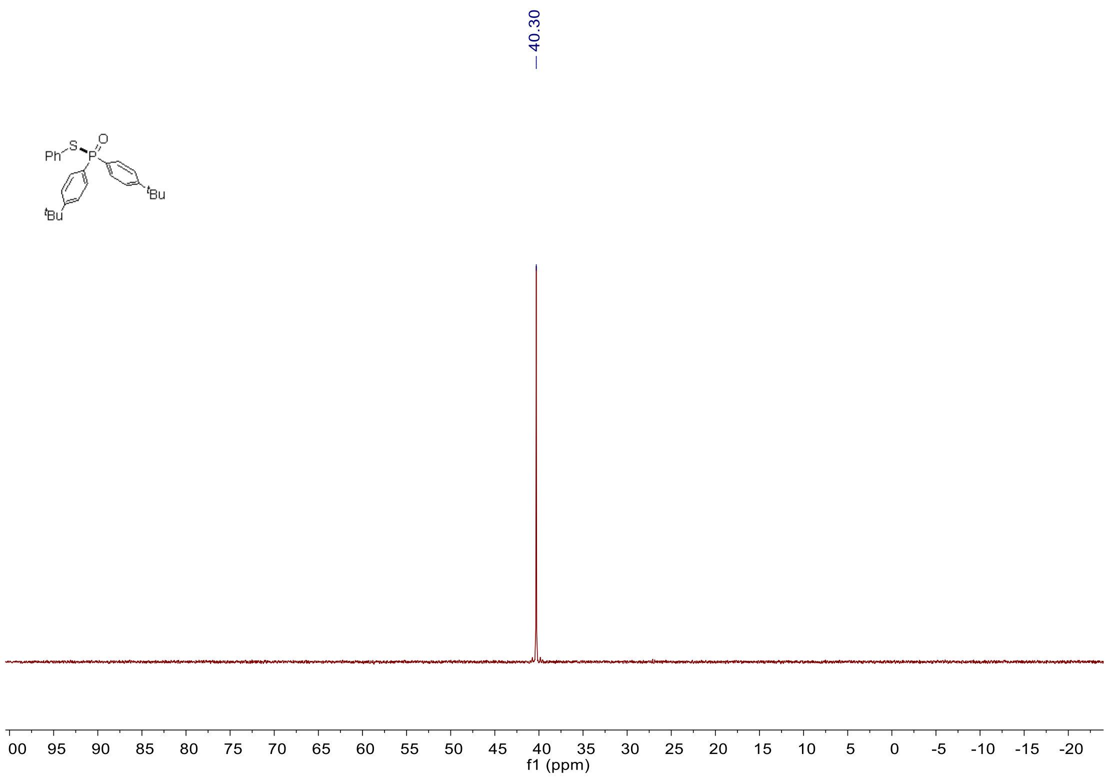

Figure S27. ^{31}P NMR spectra in $CDCl_3$ for Compound **3i**

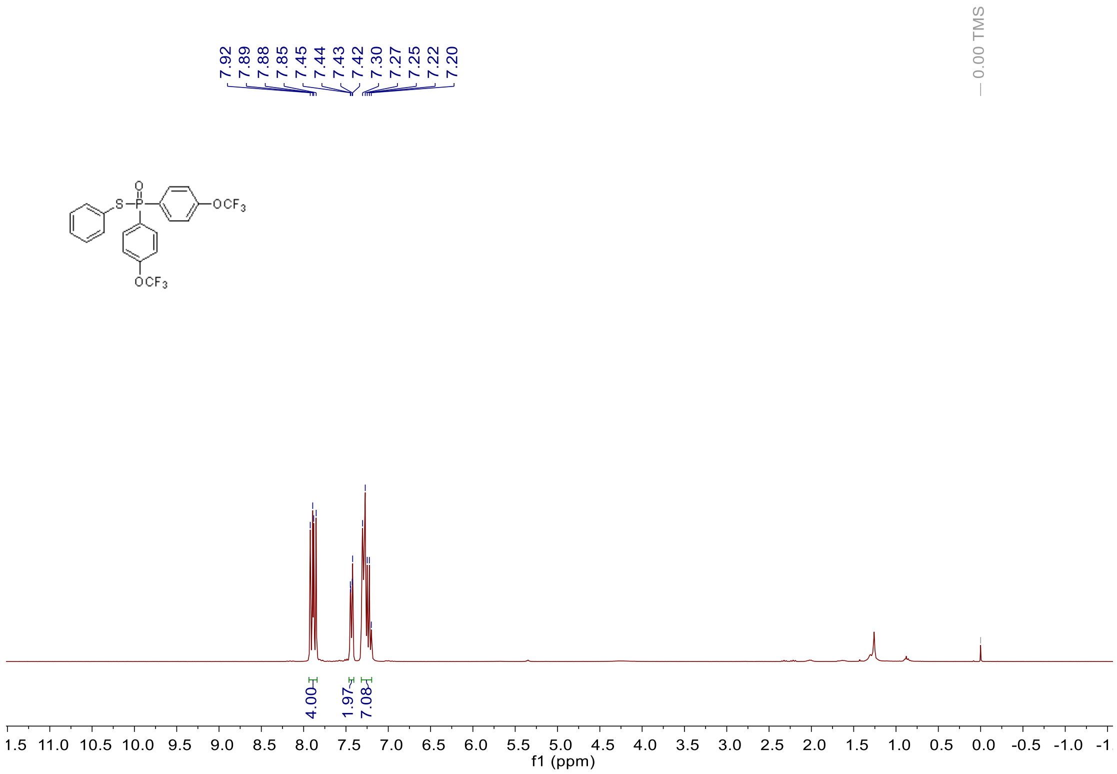

Figure S28. ^{1}H NMR spectra in $CDCl_3$ for Compound **3j**

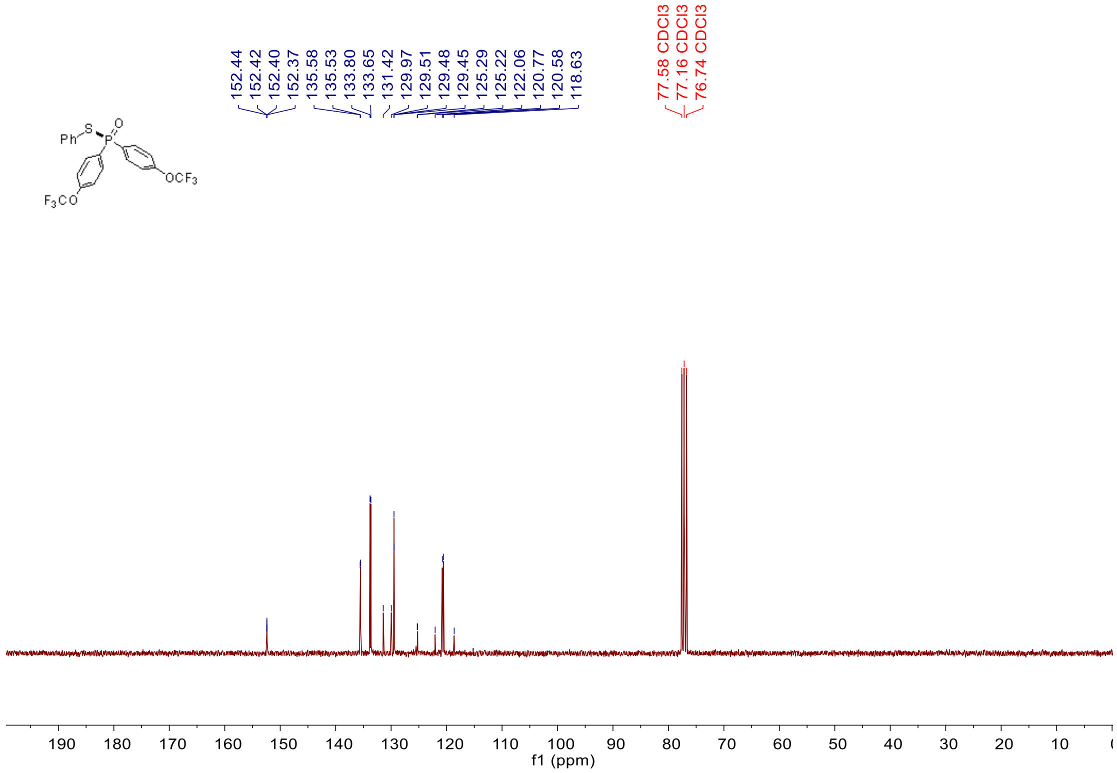

Figure S29. ^{13}C NMR spectra in $CDCl_3$ for Compound **3j**

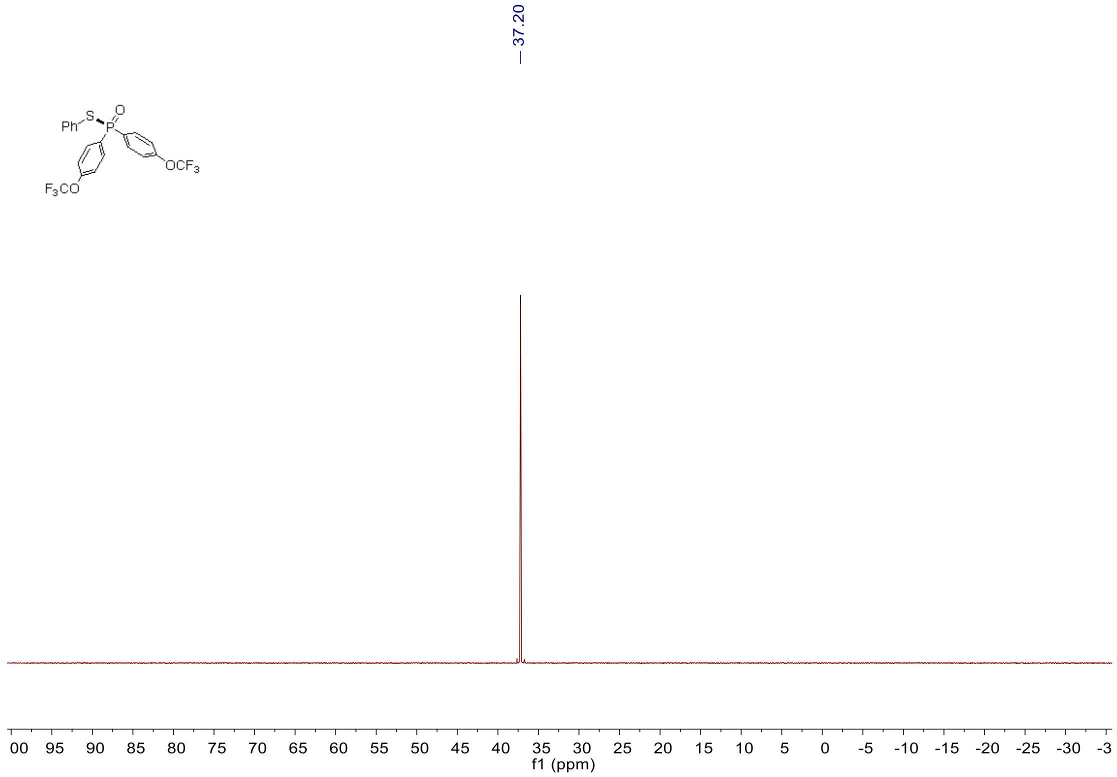

Figure S30. ^{31}P NMR spectra in $CDCl_3$ for Compound **3j**

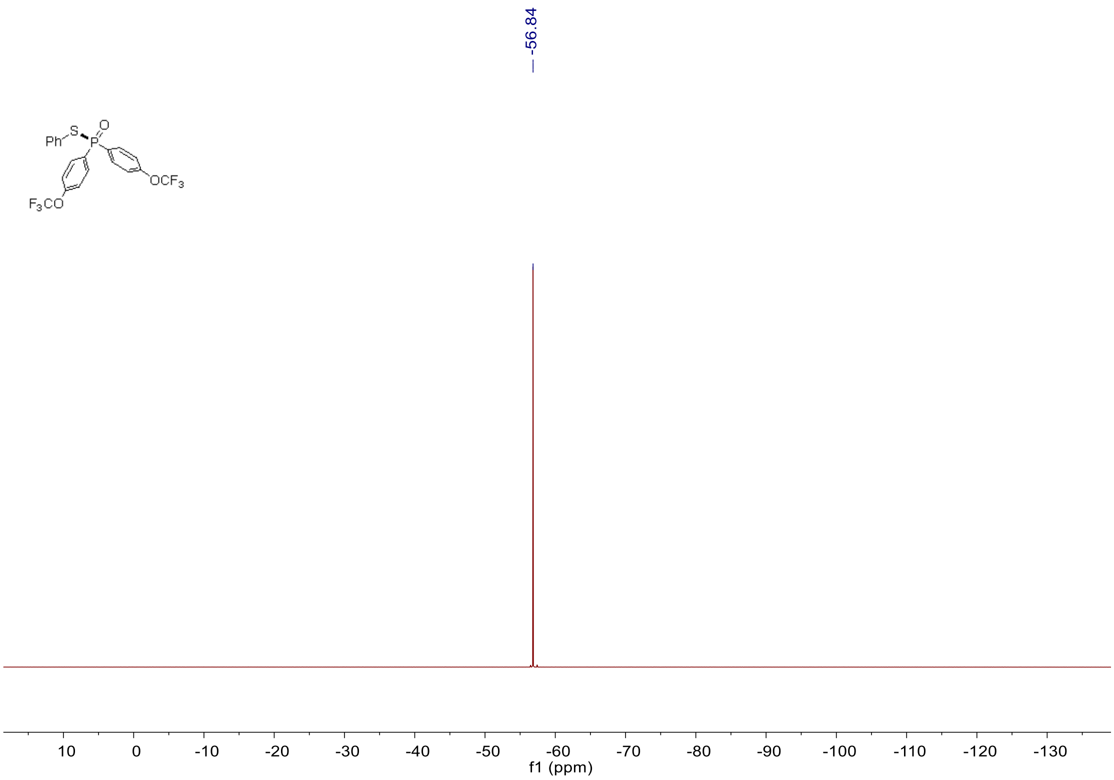

Figure S31. ^{19}F NMR spectra in $CDCl_3$ for Compound **3j**

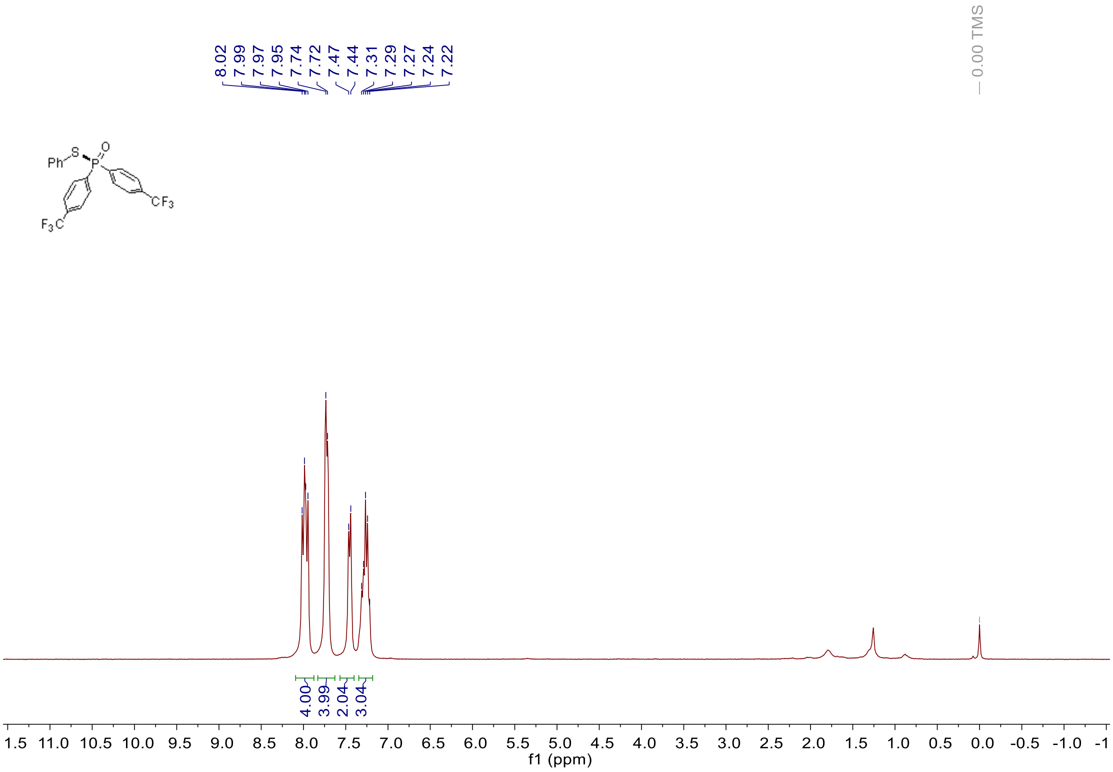

Figure S32. ^{1}H NMR spectra in $CDCl_3$ for Compound **3k**

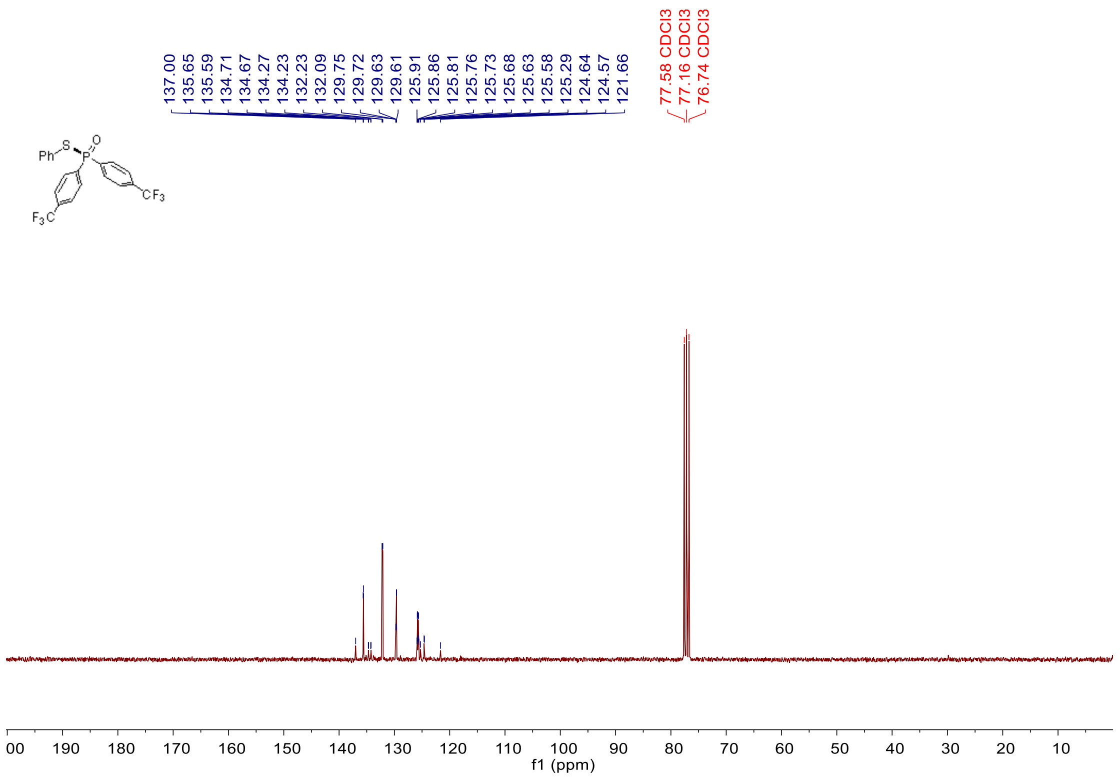

Figure S33. ^{13}C NMR spectra in $CDCl_3$ for Compound **3k**

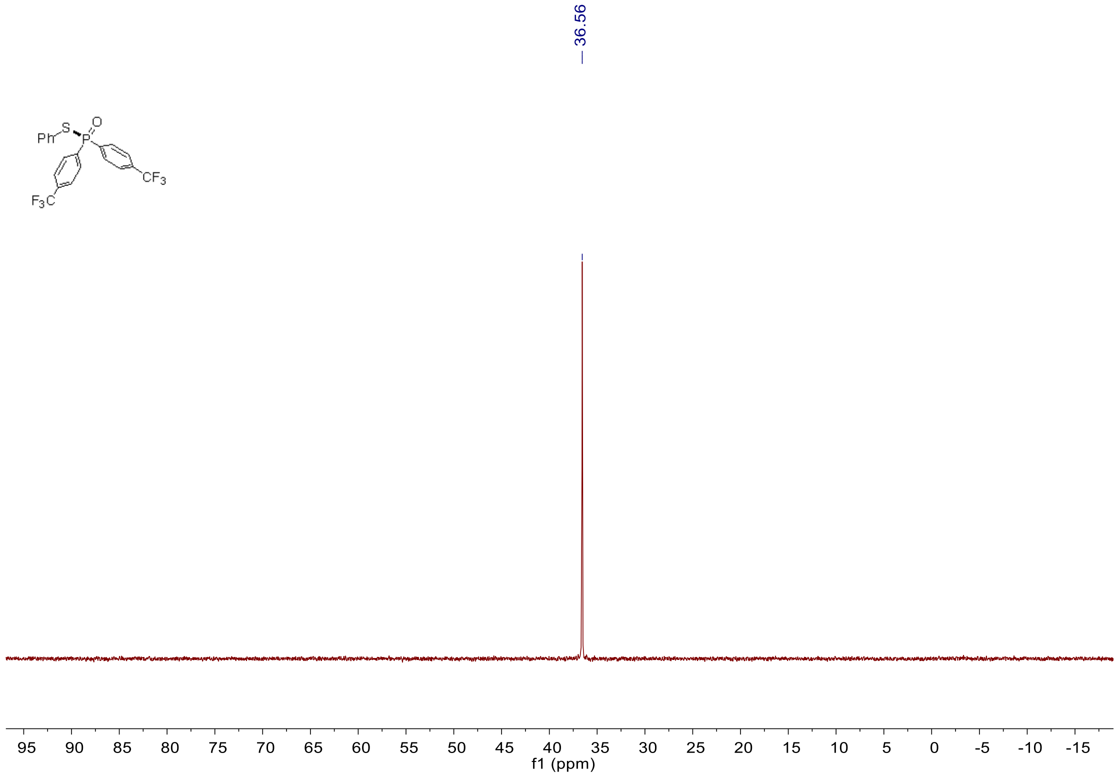

Figure S34. ^{31}P NMR spectra in $CDCl_3$ for Compound **3k**

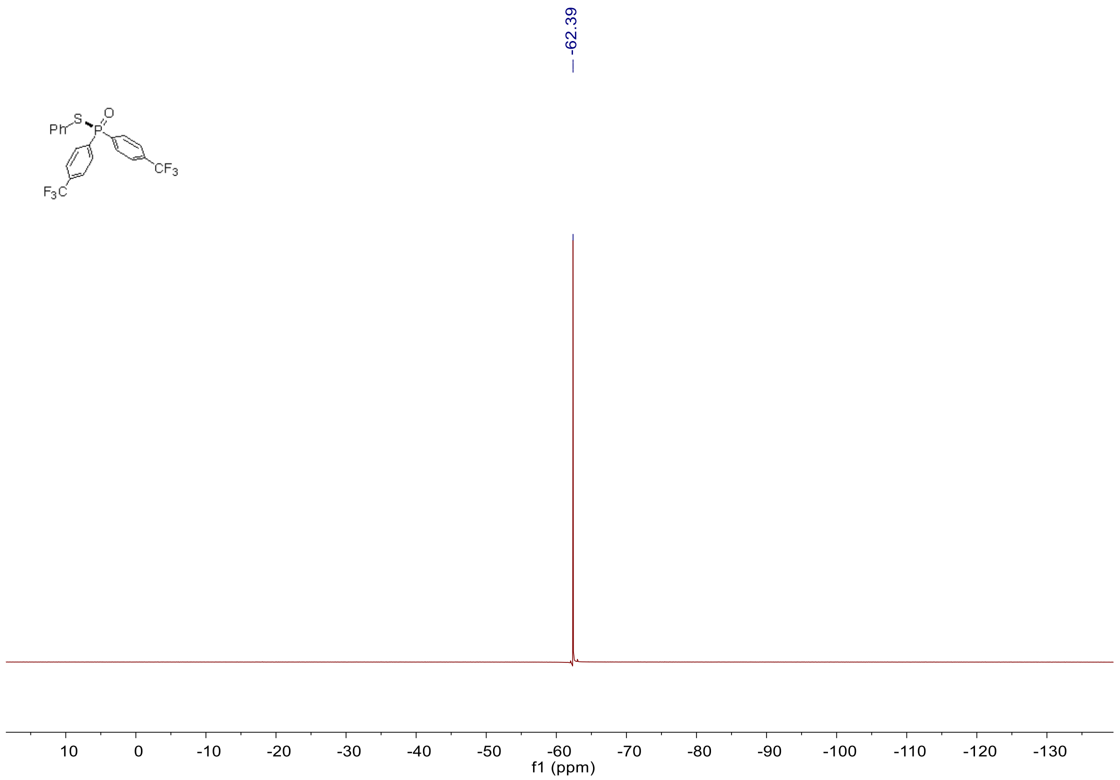

Figure S35. ^{19}F NMR spectra in $CDCl_3$ for Compound **3k**

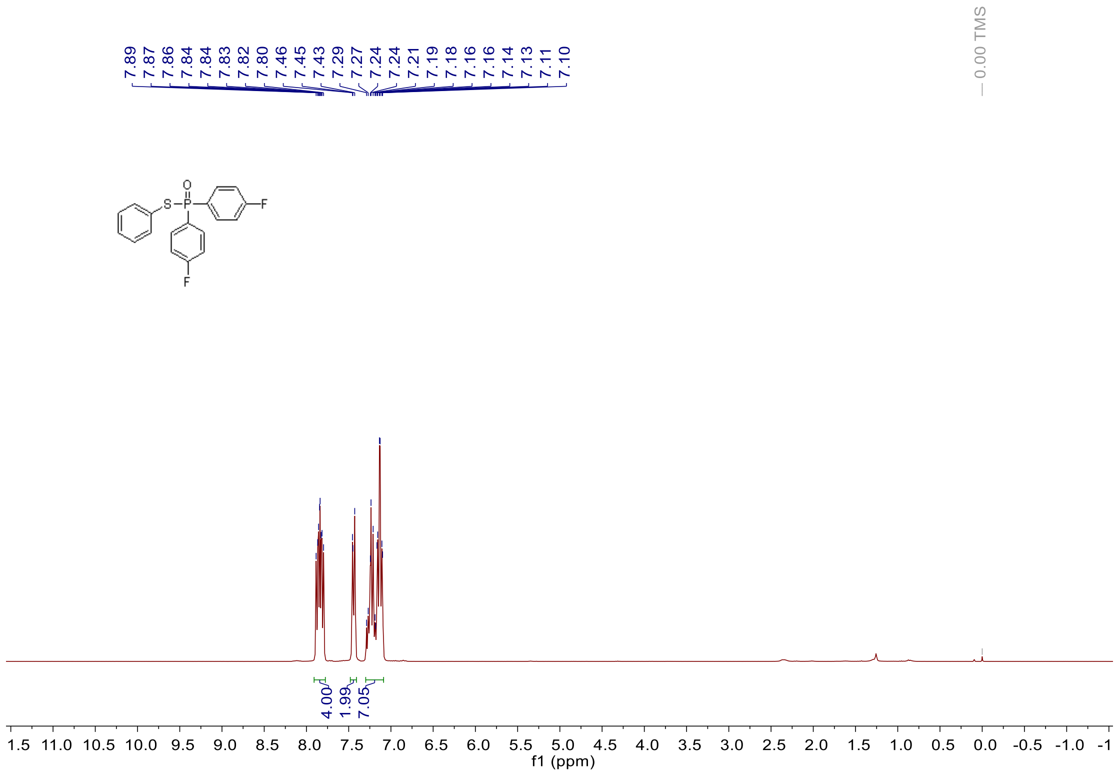

Figure S36. ^{1}H NMR spectra in $CDCl_3$ for Compound **3l**

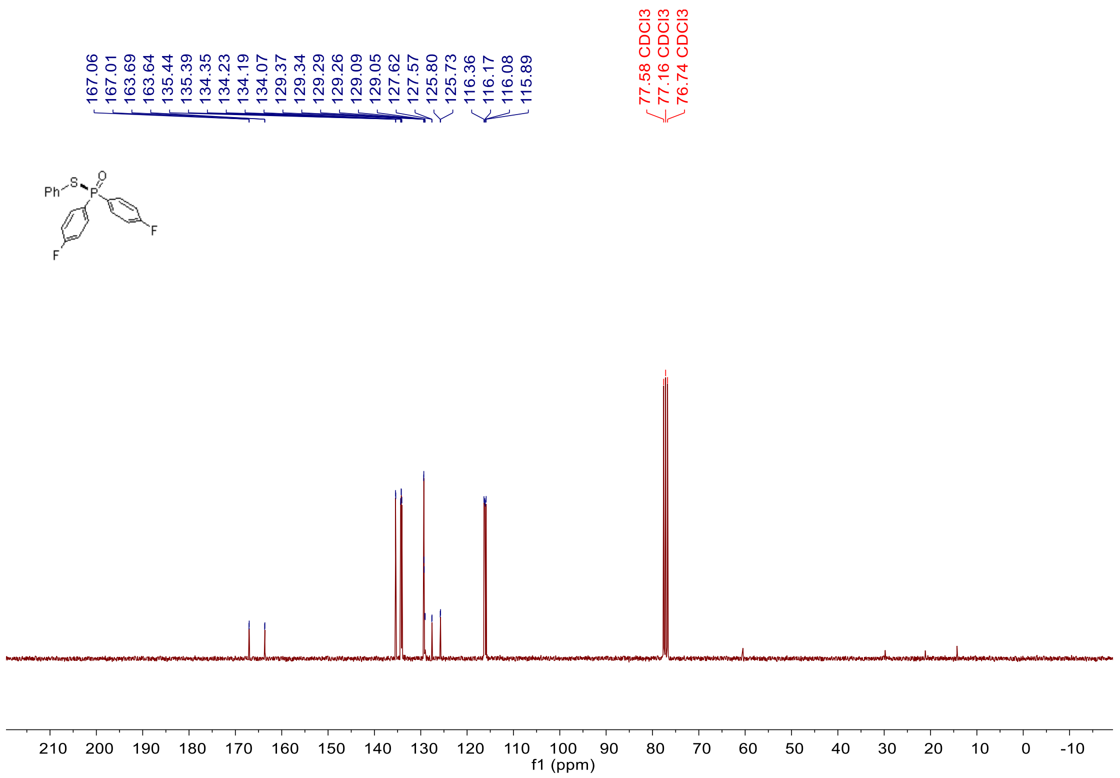

Figure S37. ^{13}C NMR spectra in $CDCl_3$ for Compound **3l**

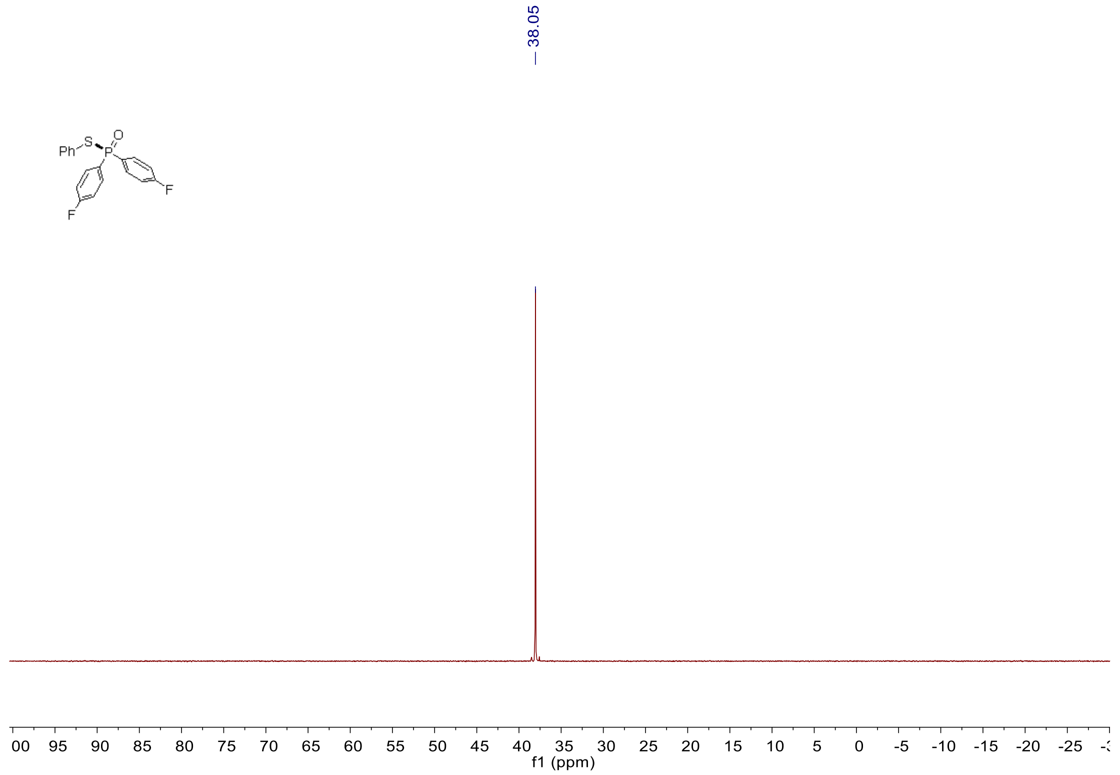

Figure S38. ^{31}P NMR spectra in $CDCl_3$ for compound **3l**

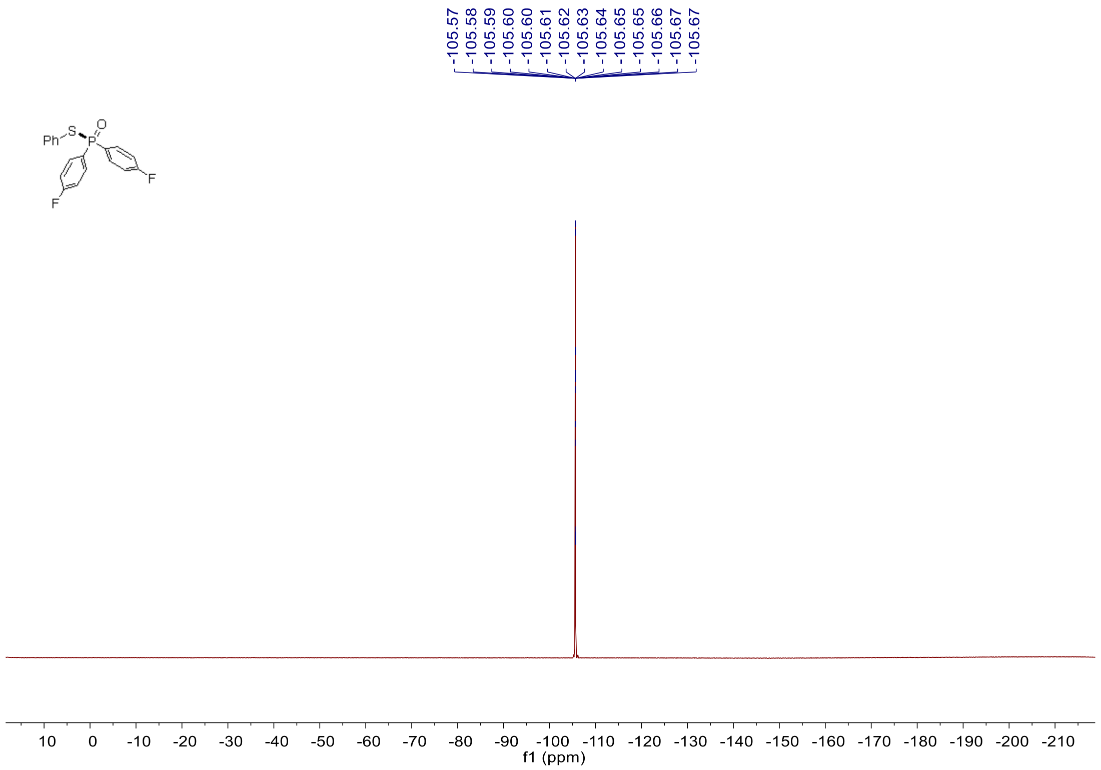

Figure S39. ^{19}F NMR spectra in $CDCl_3$ for compound **3l**

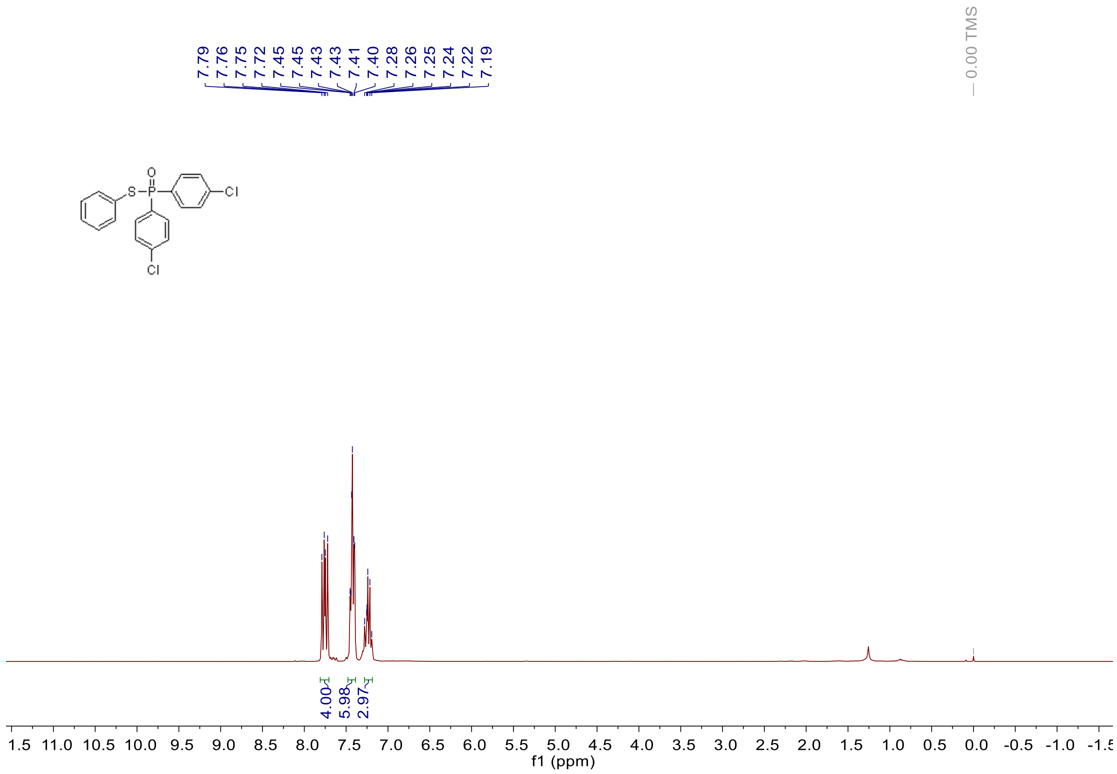

Figure S40. ^{1}H NMR spectra in $CDCl_3$ for Compound **3m**

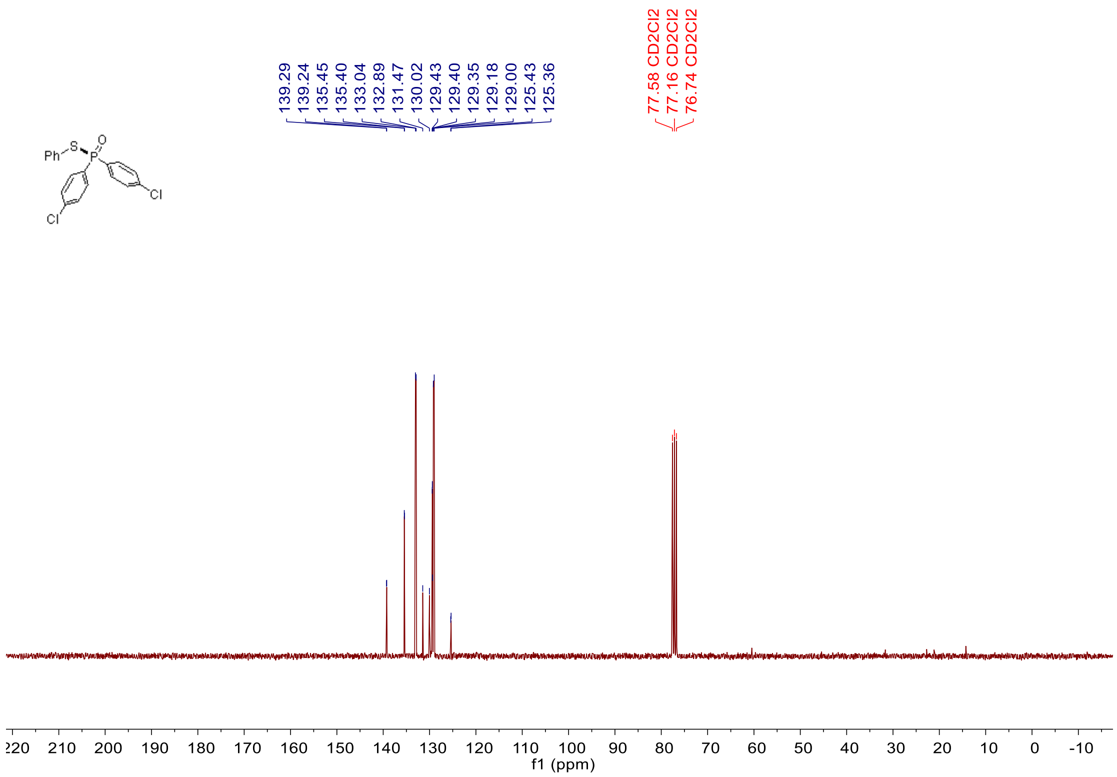

Figure S41. ^{13}C NMR spectra in $CDCl_3$ for Compound **3m**

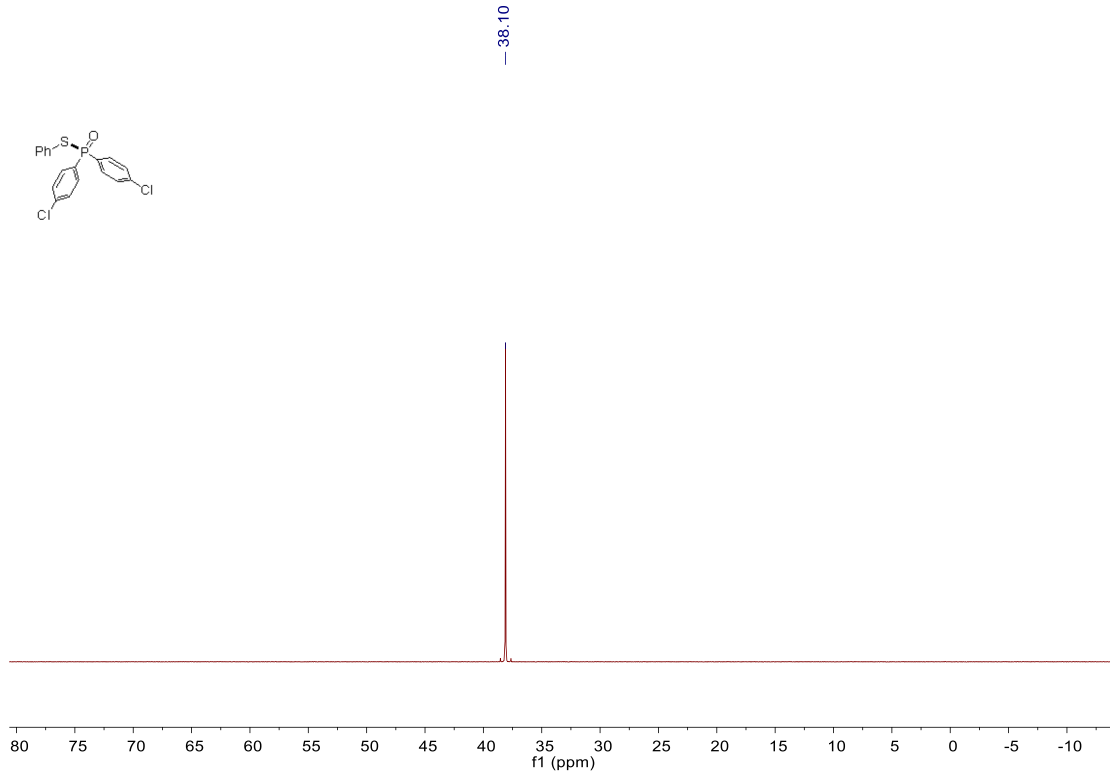

Figure S42. ^{31}P NMR spectra in $CDCl_3$ for Compound **3m**

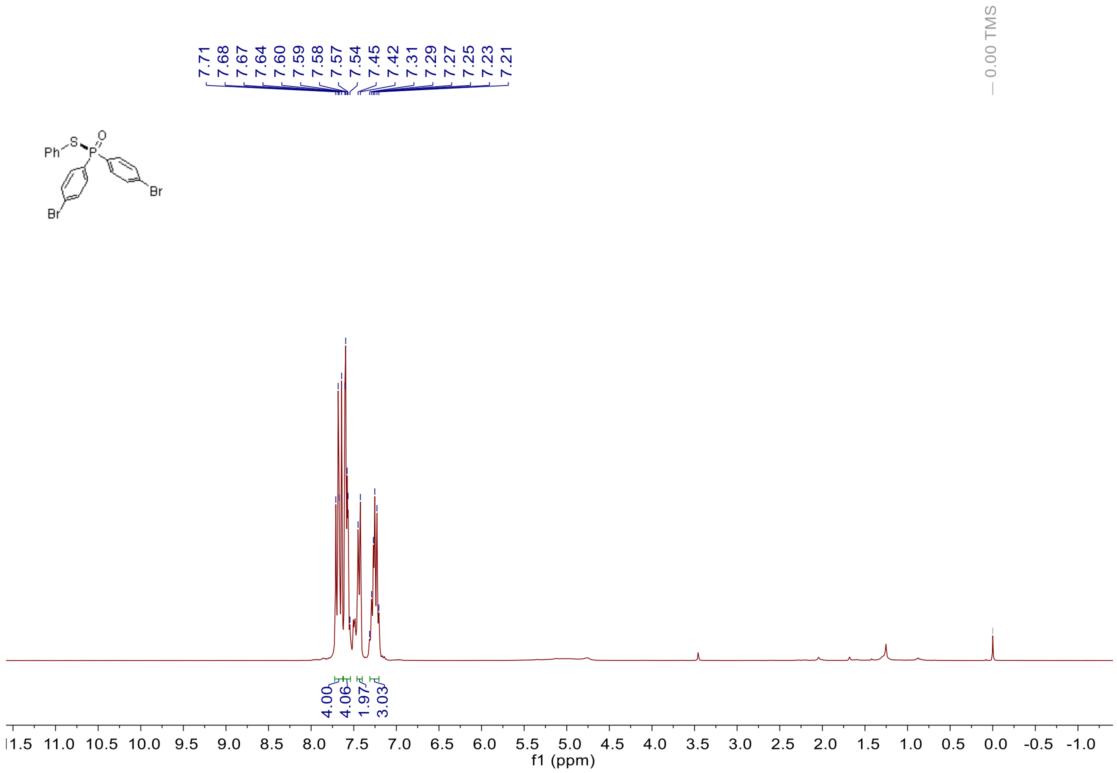

Figure S43. ^{1}H NMR spectra in $CDCl_3$ for Compound **3n**

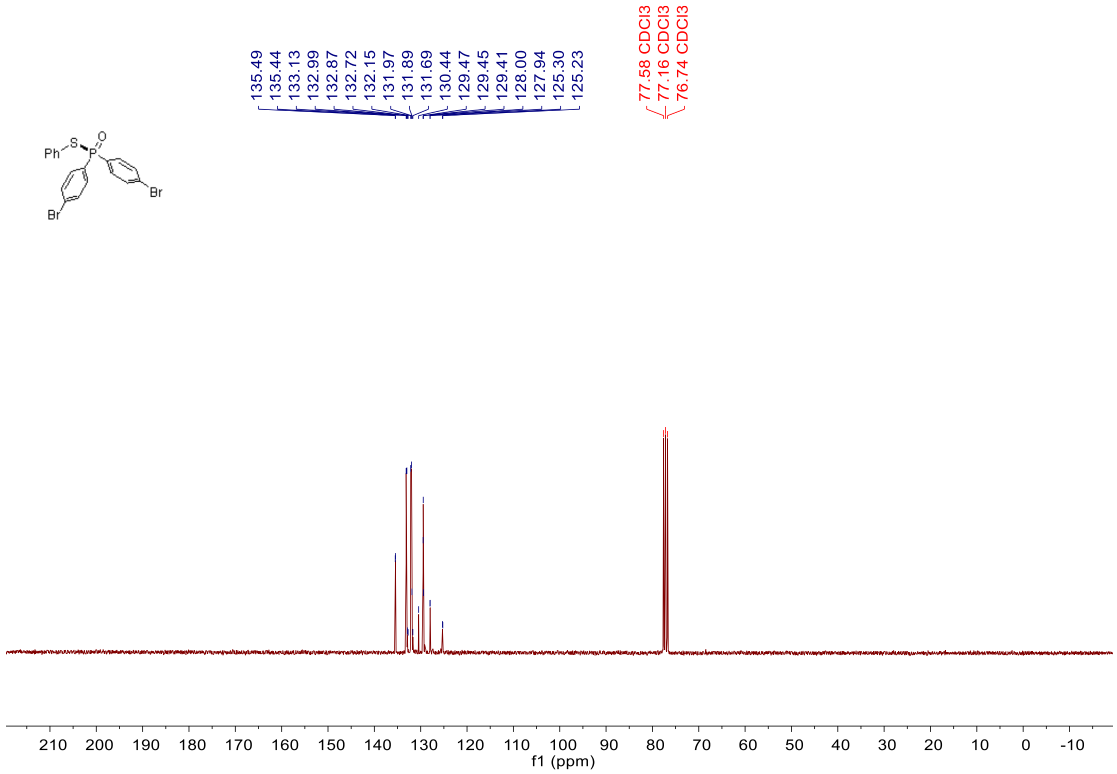

Figure S44. ^{13}C NMR spectra in $CDCl_3$ for Compound **3n**

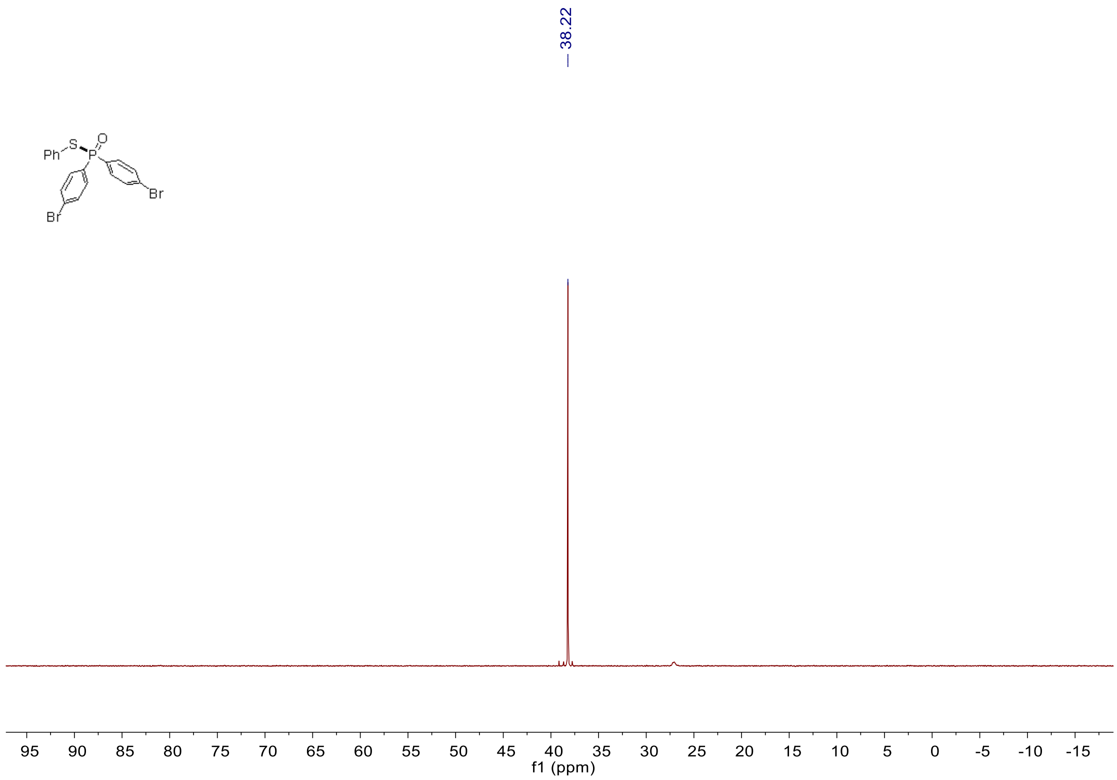

Figure S45. ^{31}P NMR spectra in $CDCl_3$ for Compound **3n**

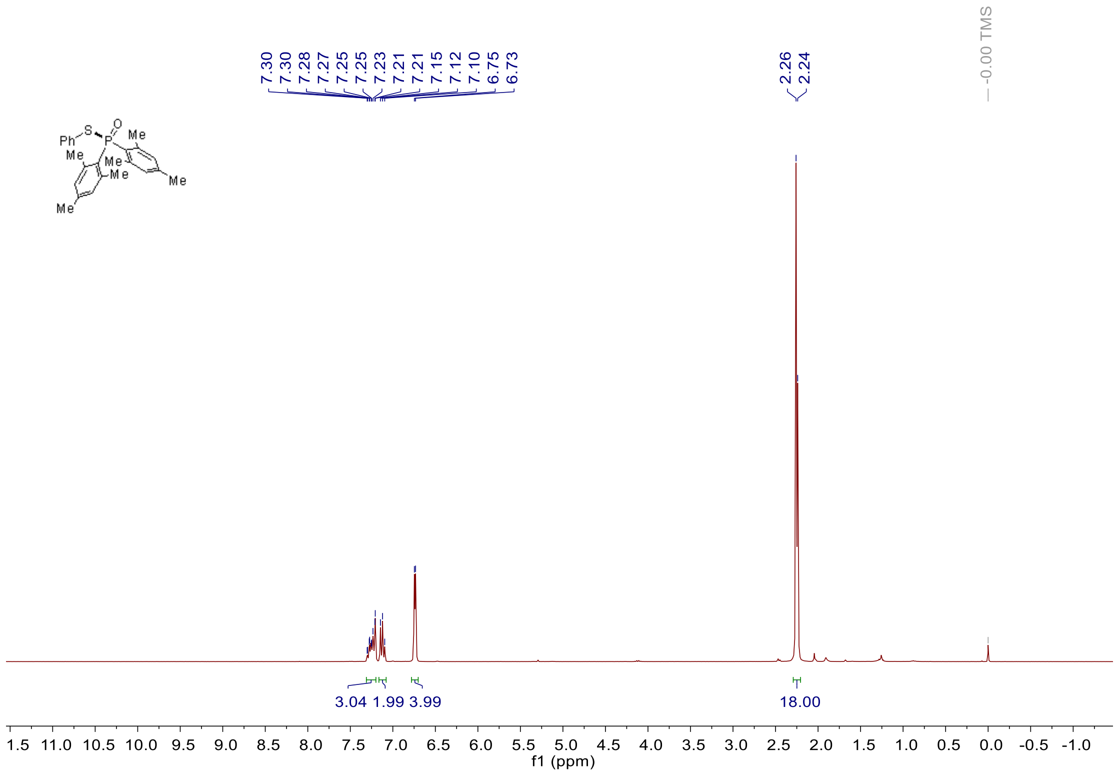

Figure S46. ^{1}H NMR spectra in $CDCl_3$ for Compound **3o**

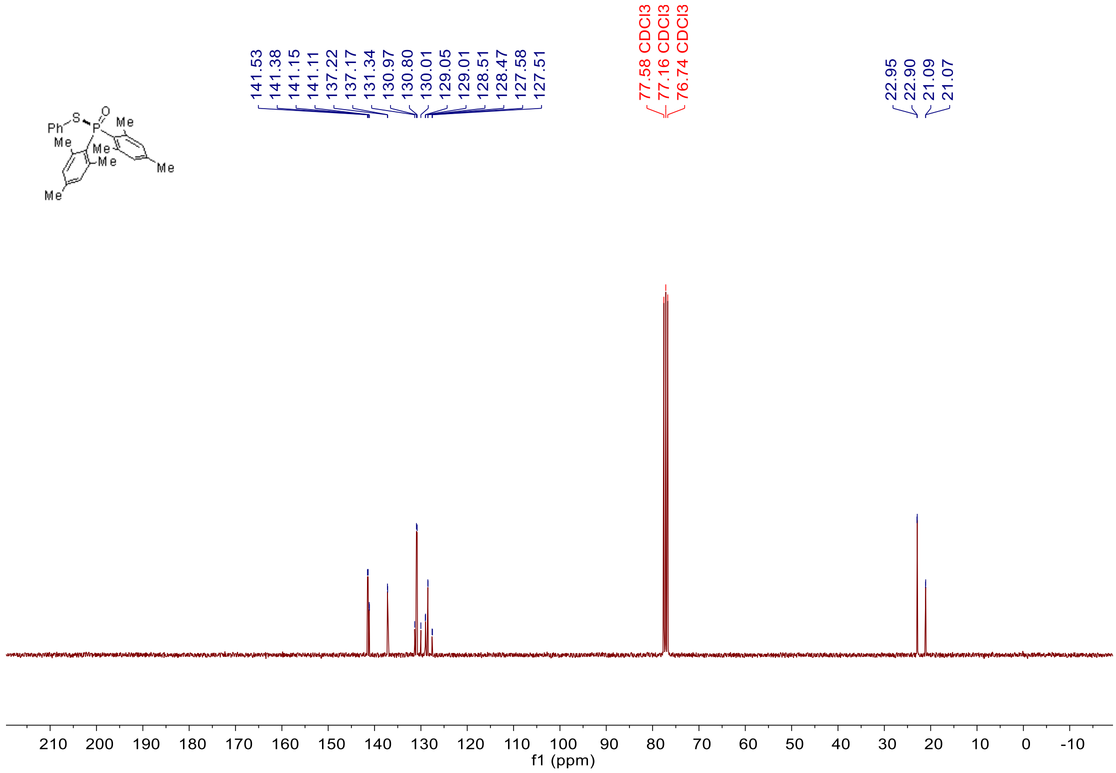

Figure S47. ^{13}C NMR spectra in $CDCl_3$ for Compound **3o**

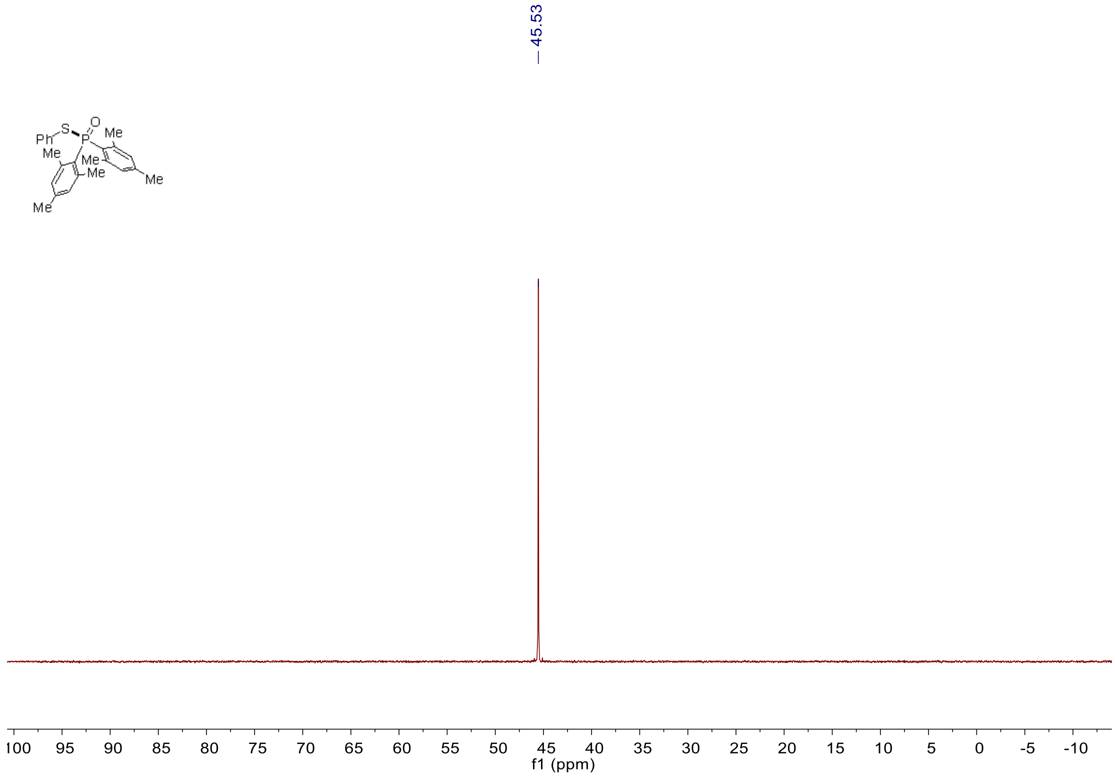

Figure S48. ^{31}P NMR spectra in $CDCl_3$ for Compound **3o**

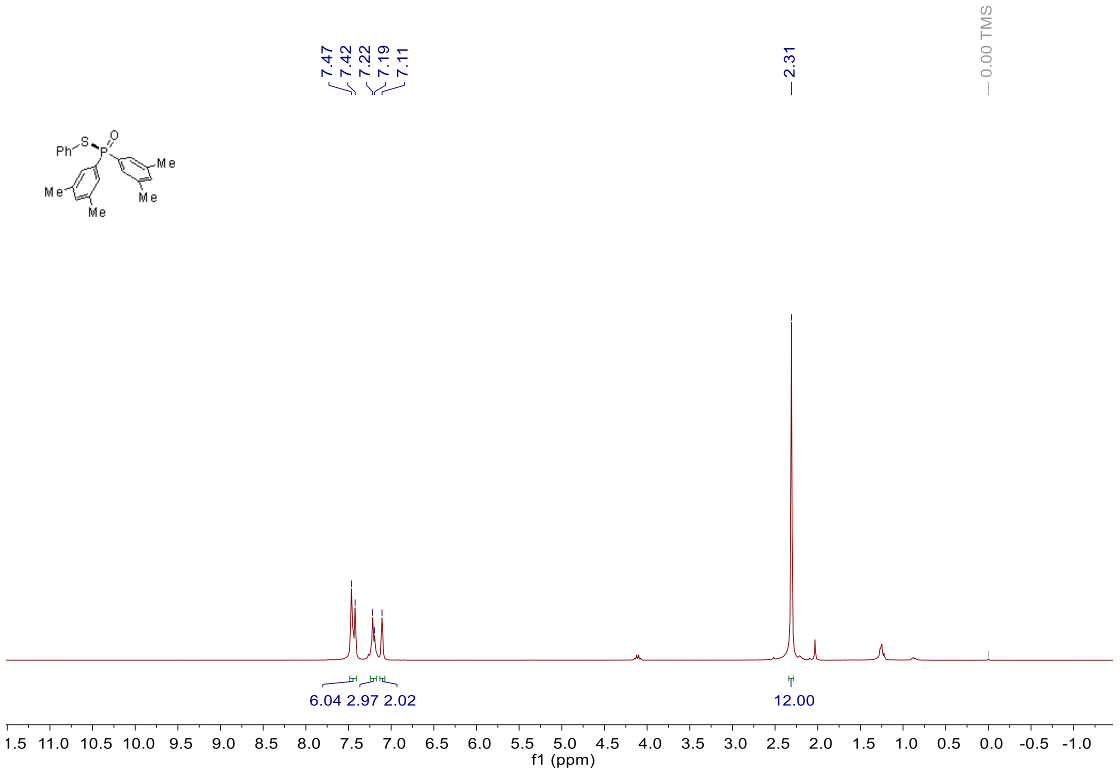

Figure S49. ^{1}H NMR spectra in $CDCl_3$ for Compound **3p**

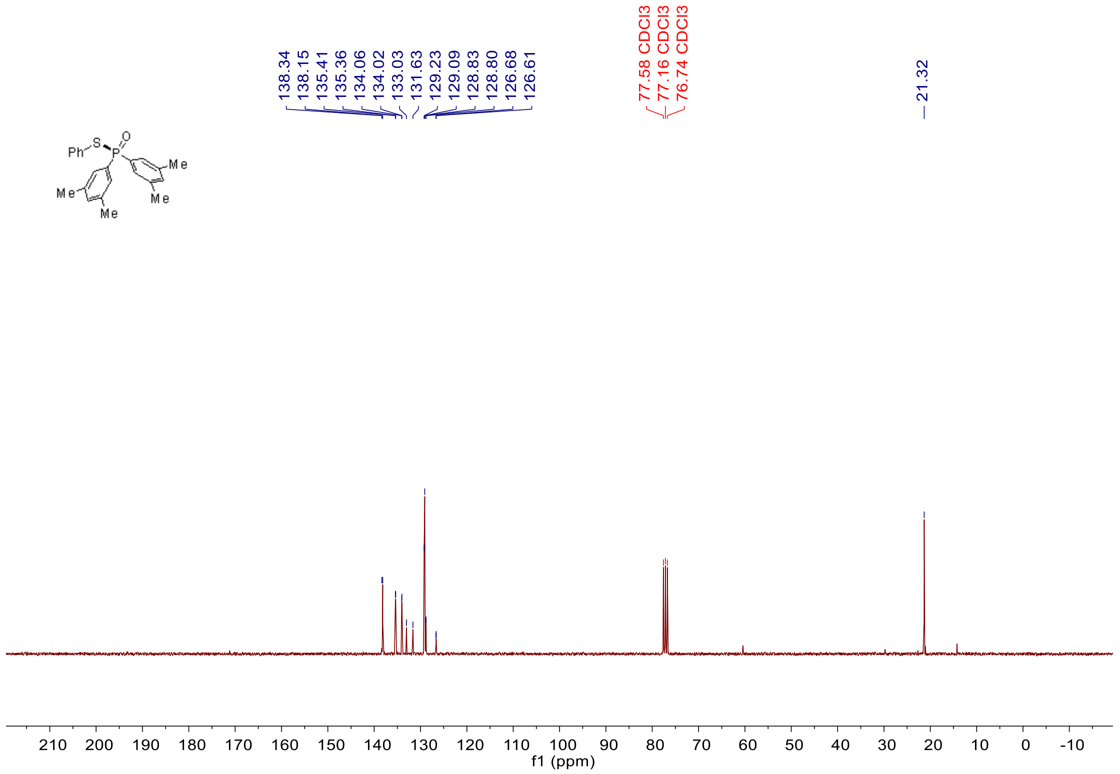

Figure S50. ^{13}C NMR spectra in $CDCl_3$ for Compound **3p**

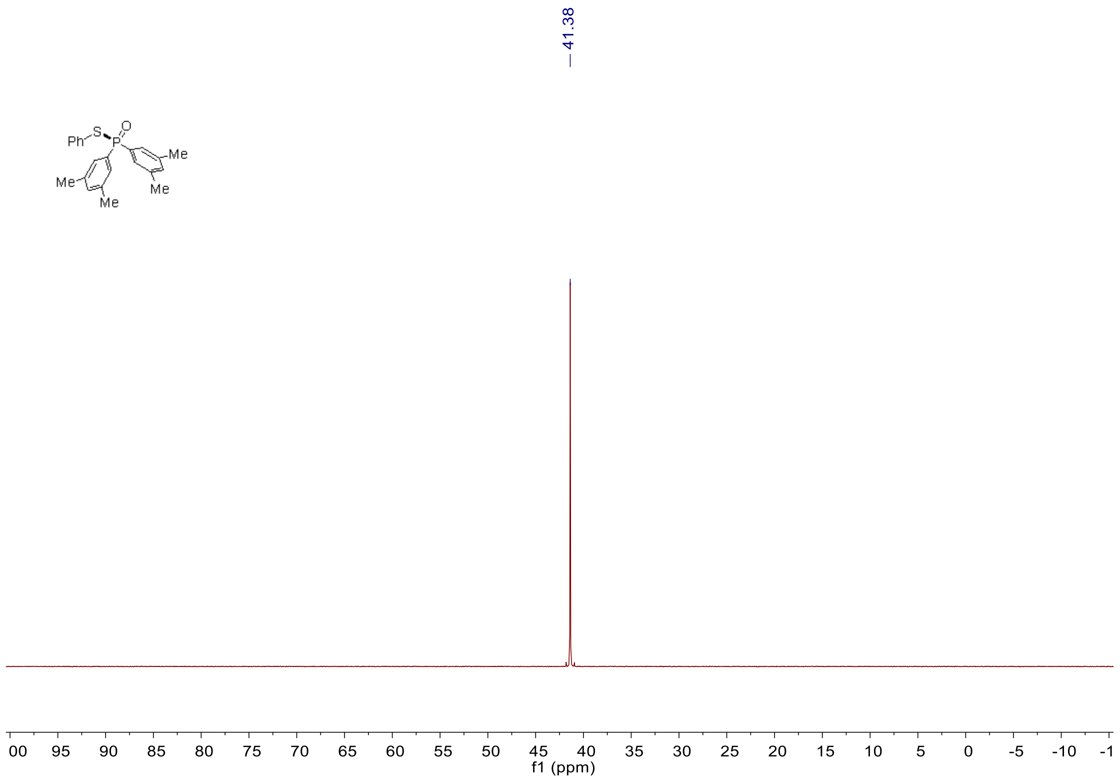

Figure S51. ^{31}P NMR spectra in $CDCl_3$ for Compound **3p**

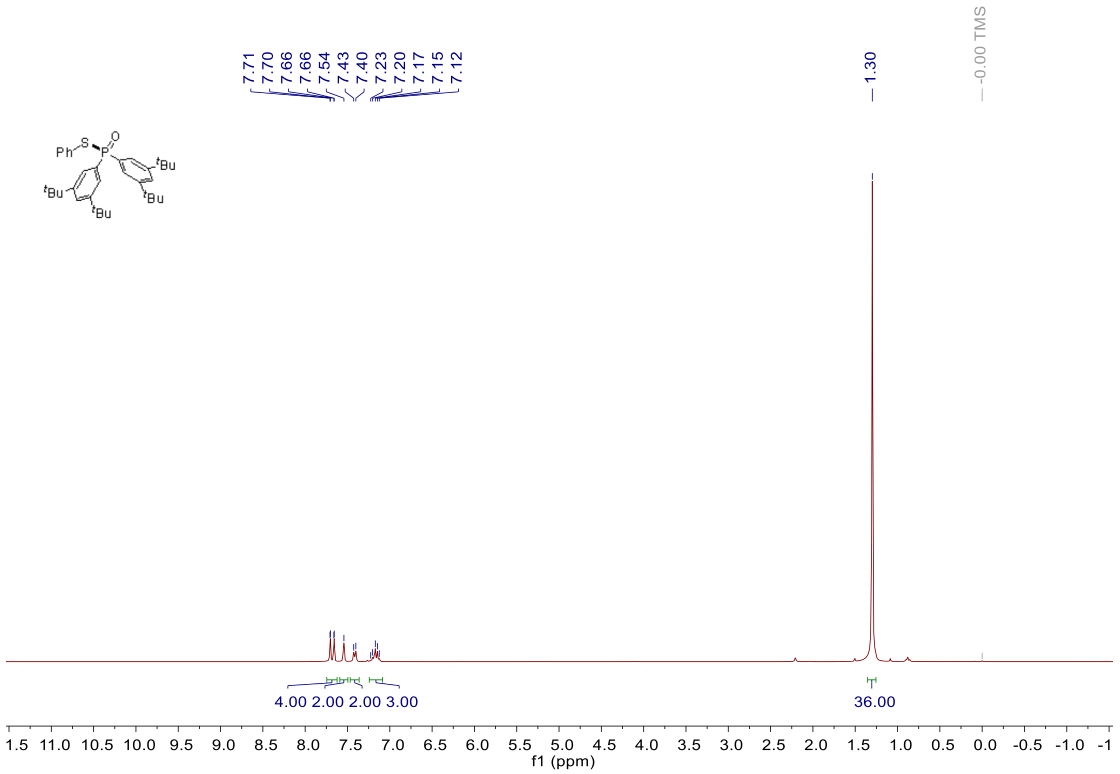

Figure S52. ^{1}H NMR spectra in $CDCl_3$ for Compound **3q**

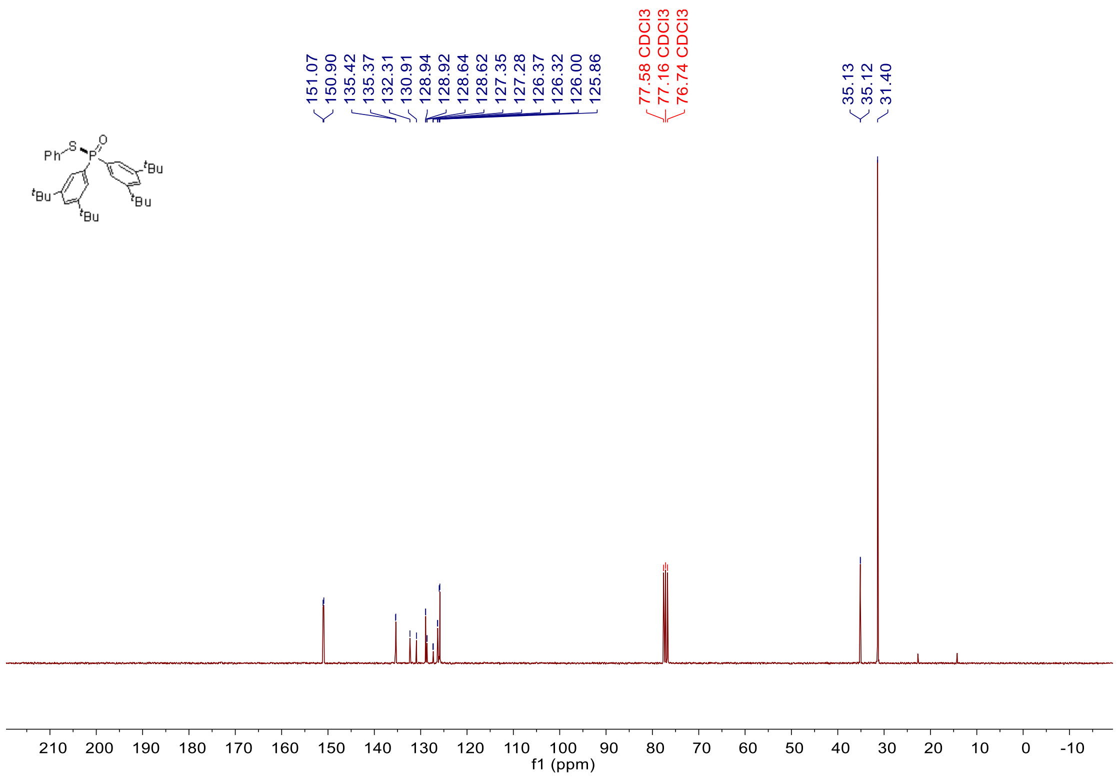

Figure S53. ^{13}C NMR spectra in $CDCl_3$ for Compound **3q**

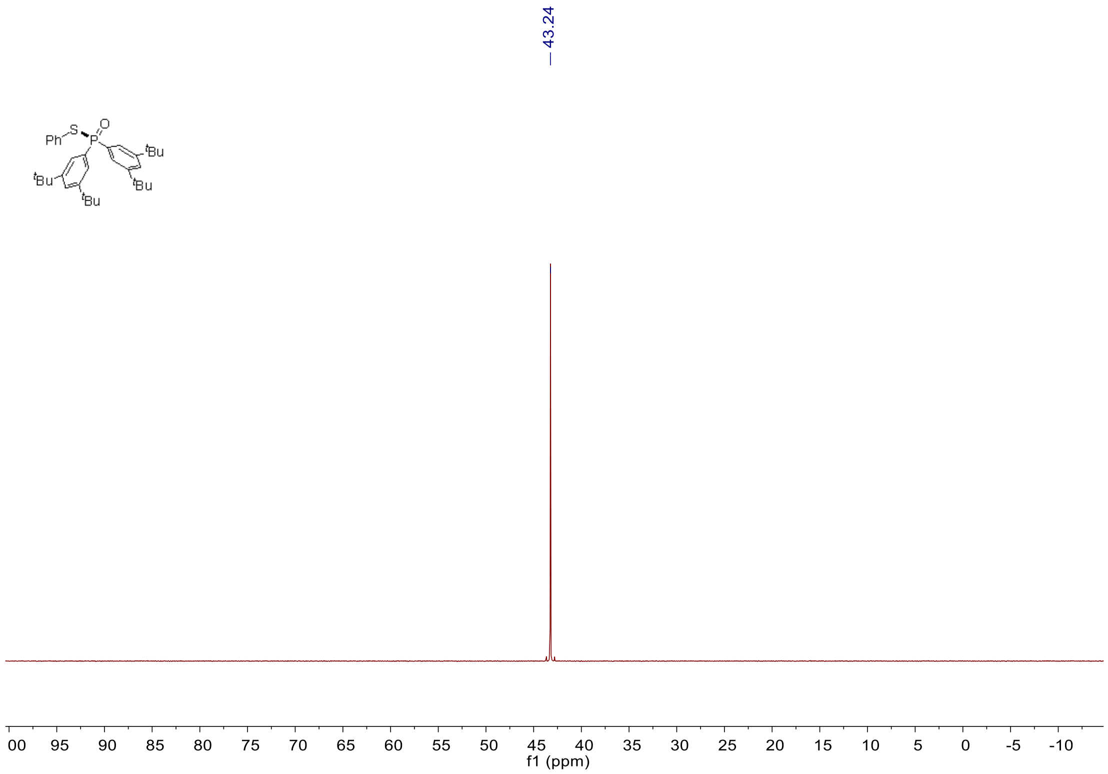

Figure S54. ^{31}P NMR spectra in $CDCl_3$ for Compound **3q**

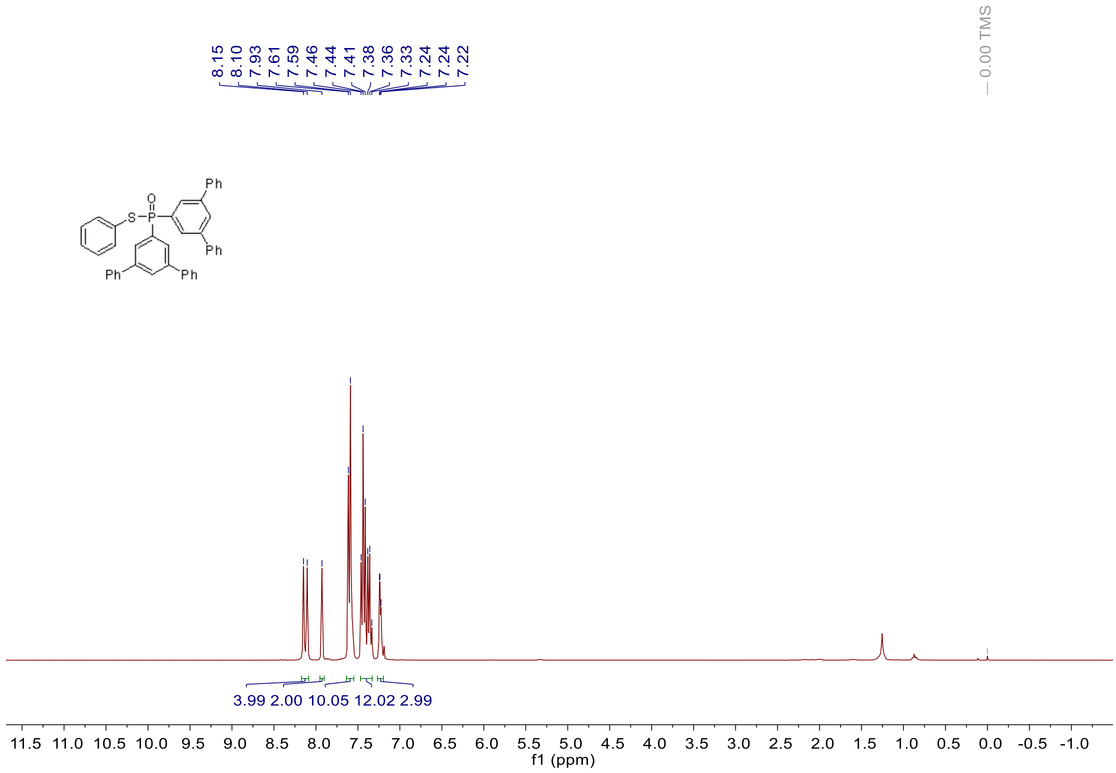

Figure S55. ^{1}H NMR spectra in $CDCl_3$ for Compound **3r**

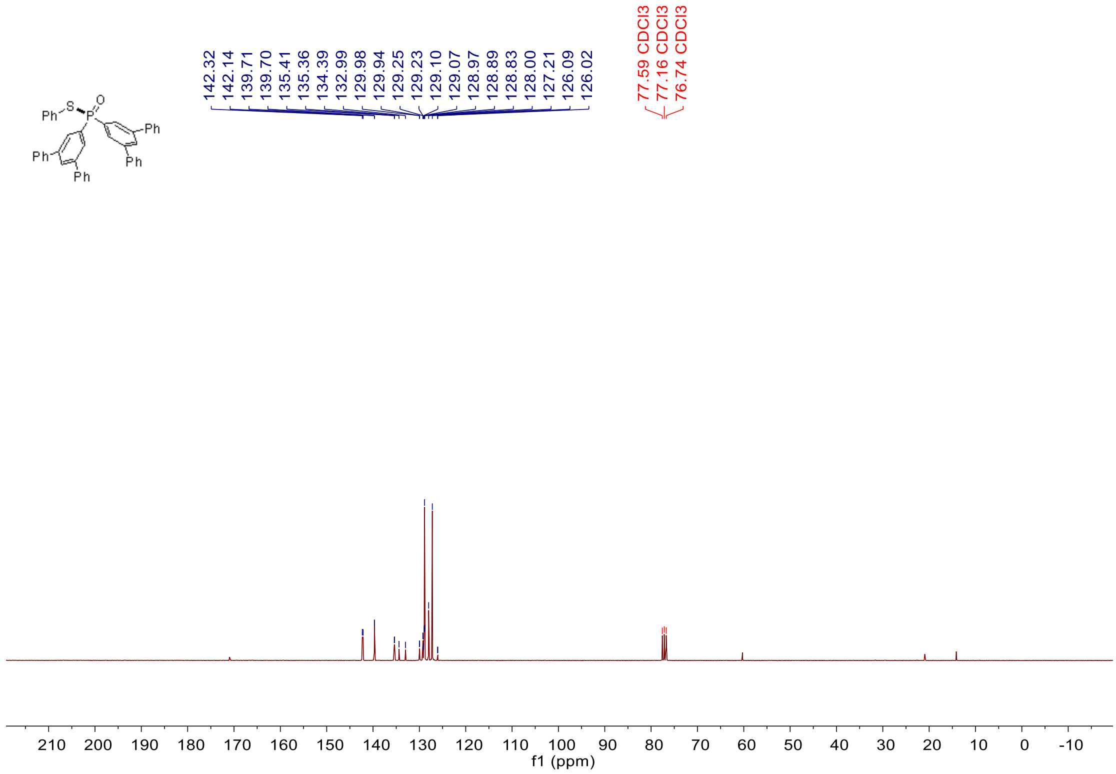

Figure S56. ^{13}C NMR spectra in $CDCl_3$ for Compound **3r**

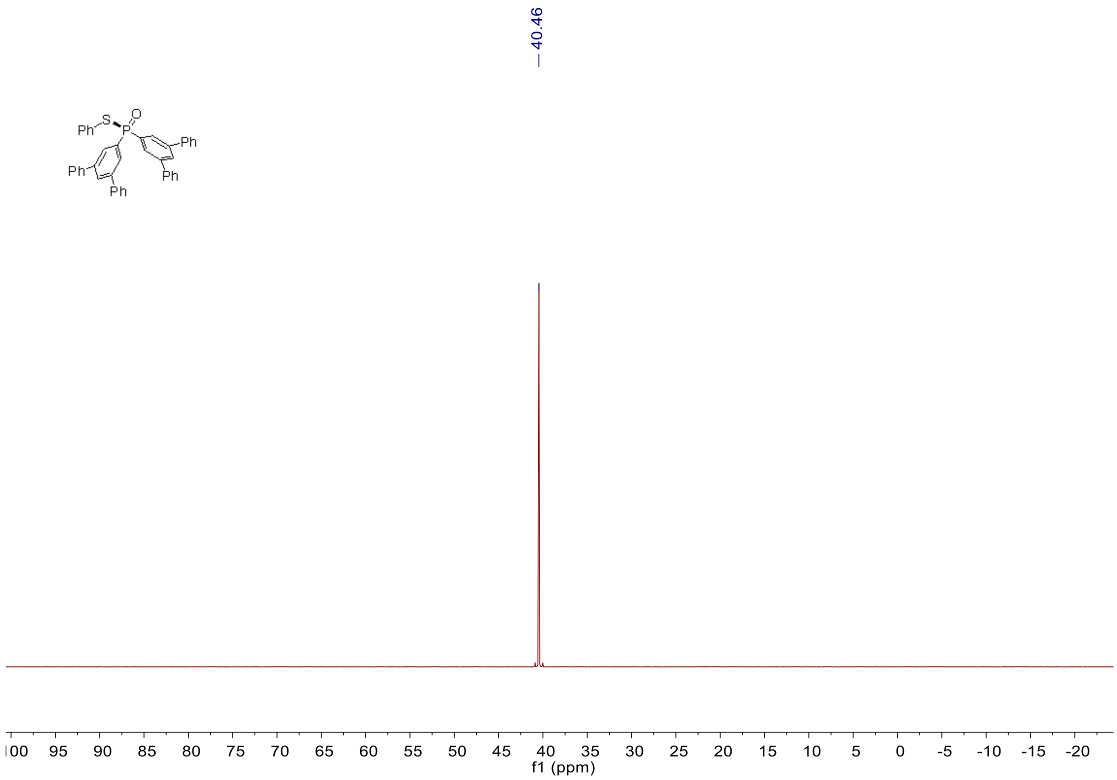

Figure S57. ^{31}P NMR spectra in $CDCl_3$ for Compound **3r**

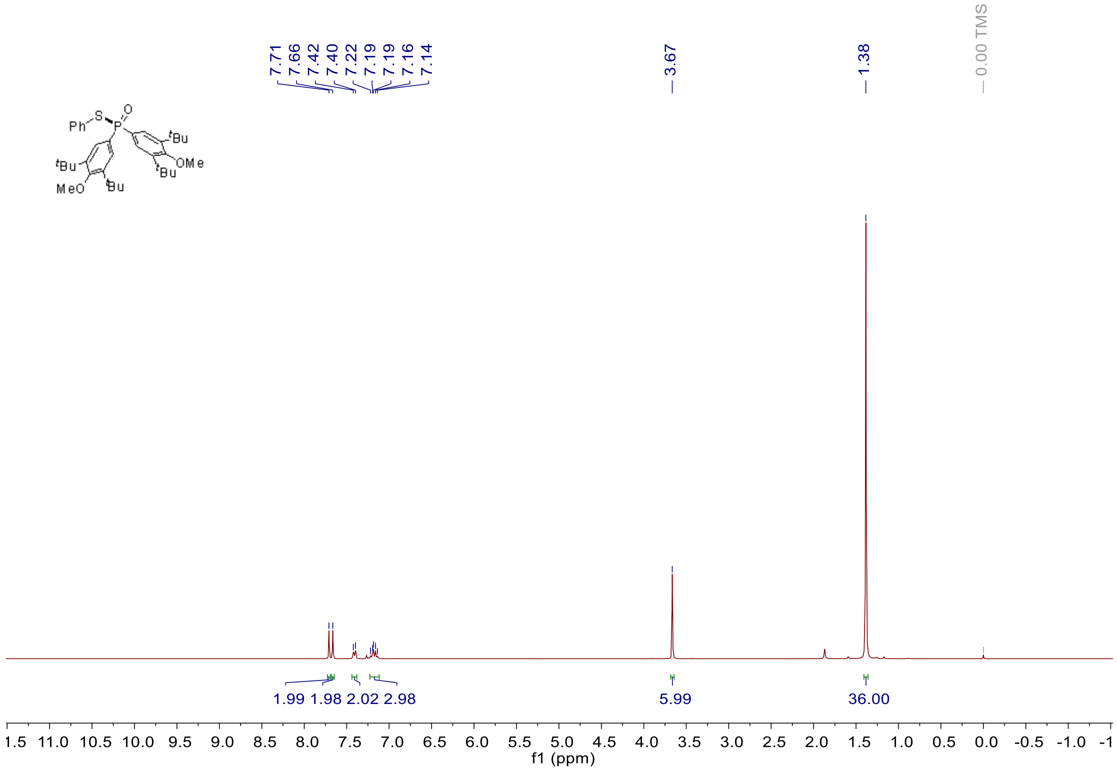

Figure S58. ^{1}H NMR spectra in $CDCl_3$ for Compound **3s**

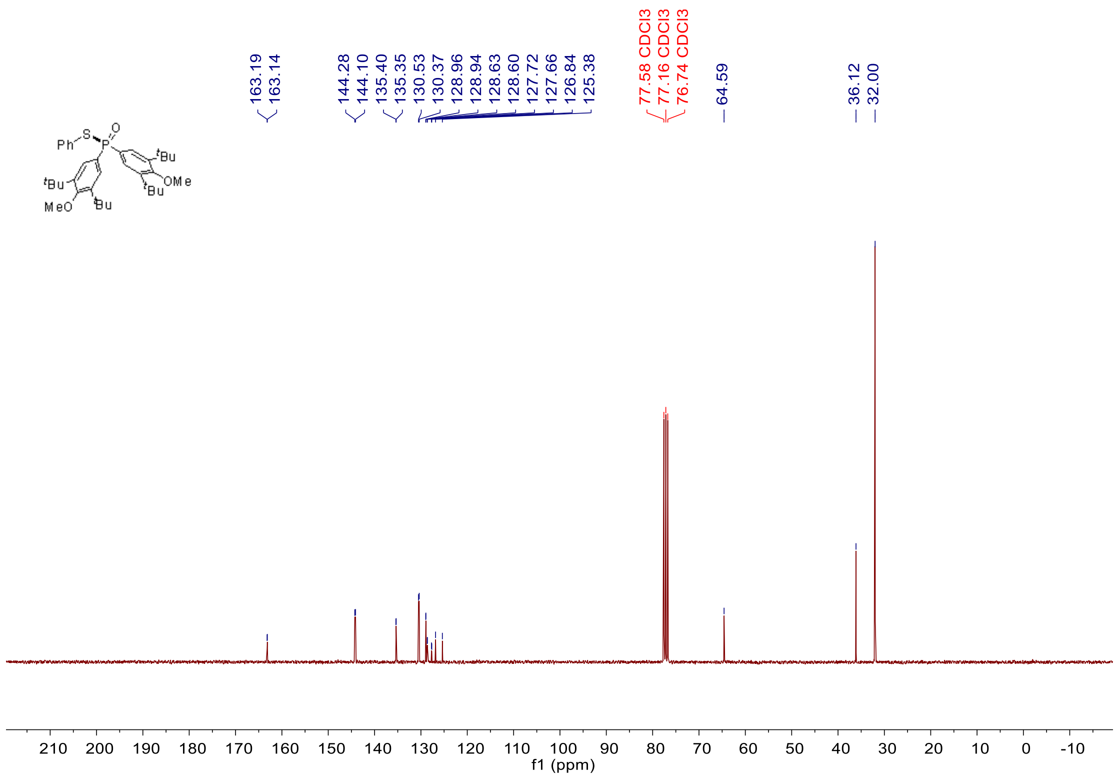

Figure S59. ^{13}C NMR spectra in $CDCl_3$ for Compound **3s**

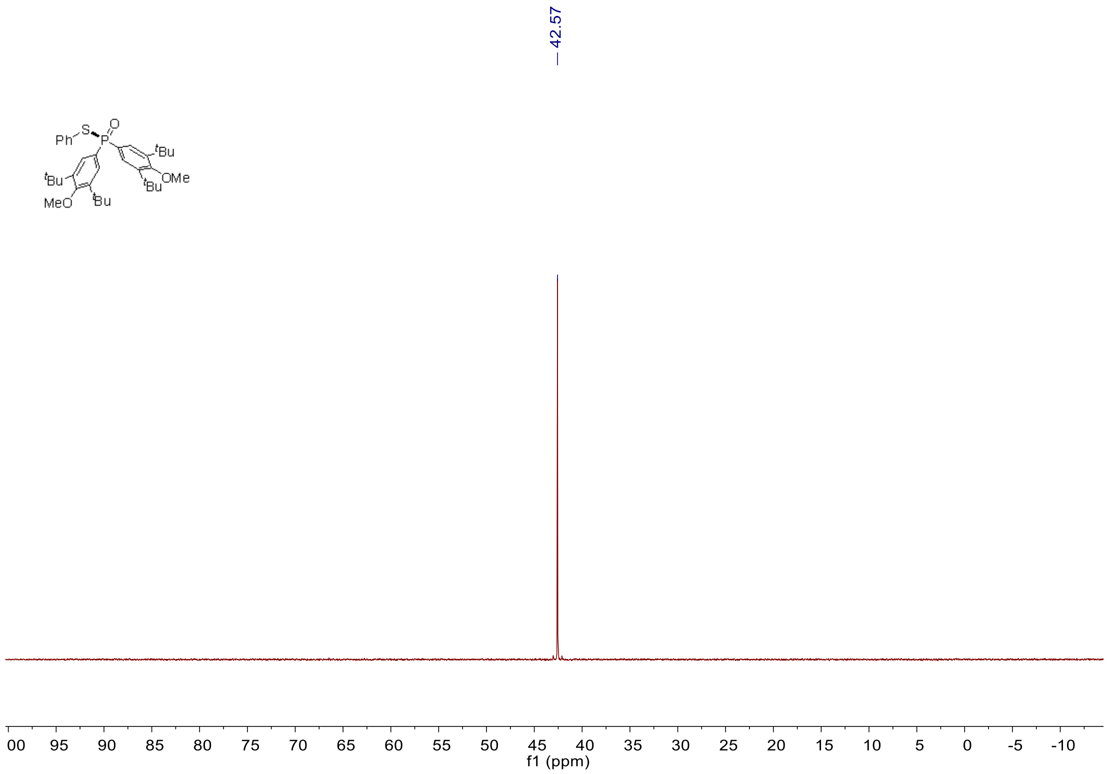

Figure S60. ^{31}P NMR spectra in $CDCl_3$ for Compound **3s**

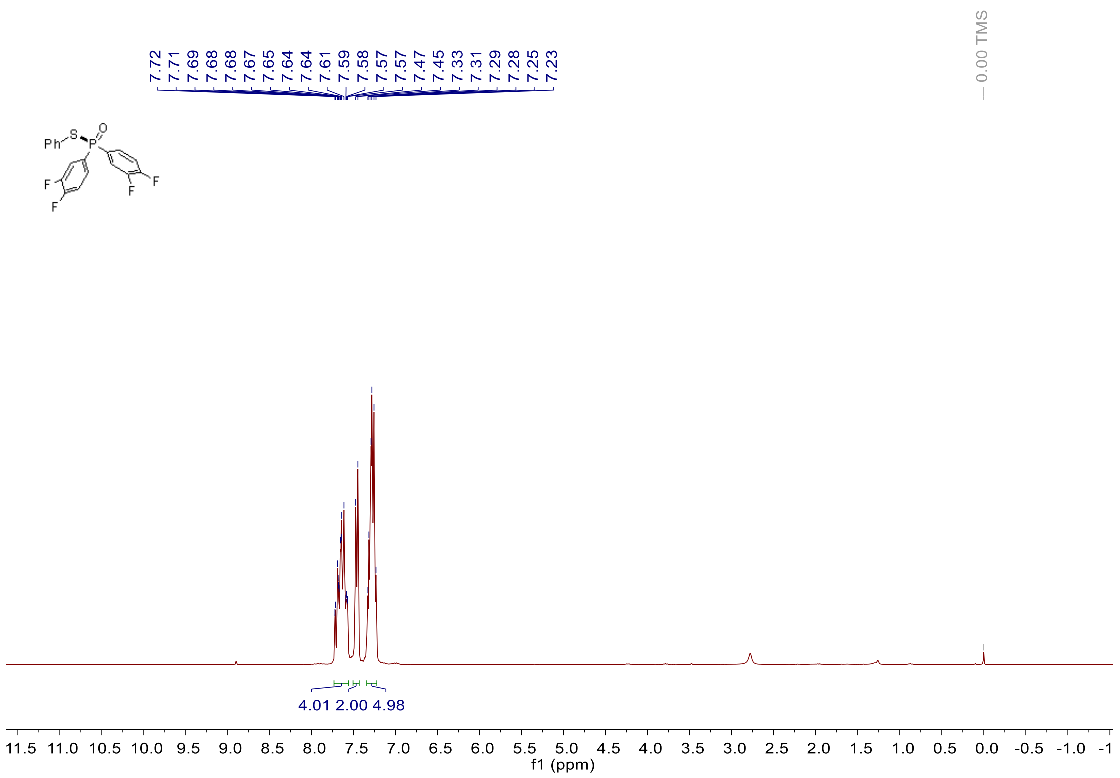

Figure S61. ^{1}H NMR spectra in $CDCl_3$ for Compound **3t**

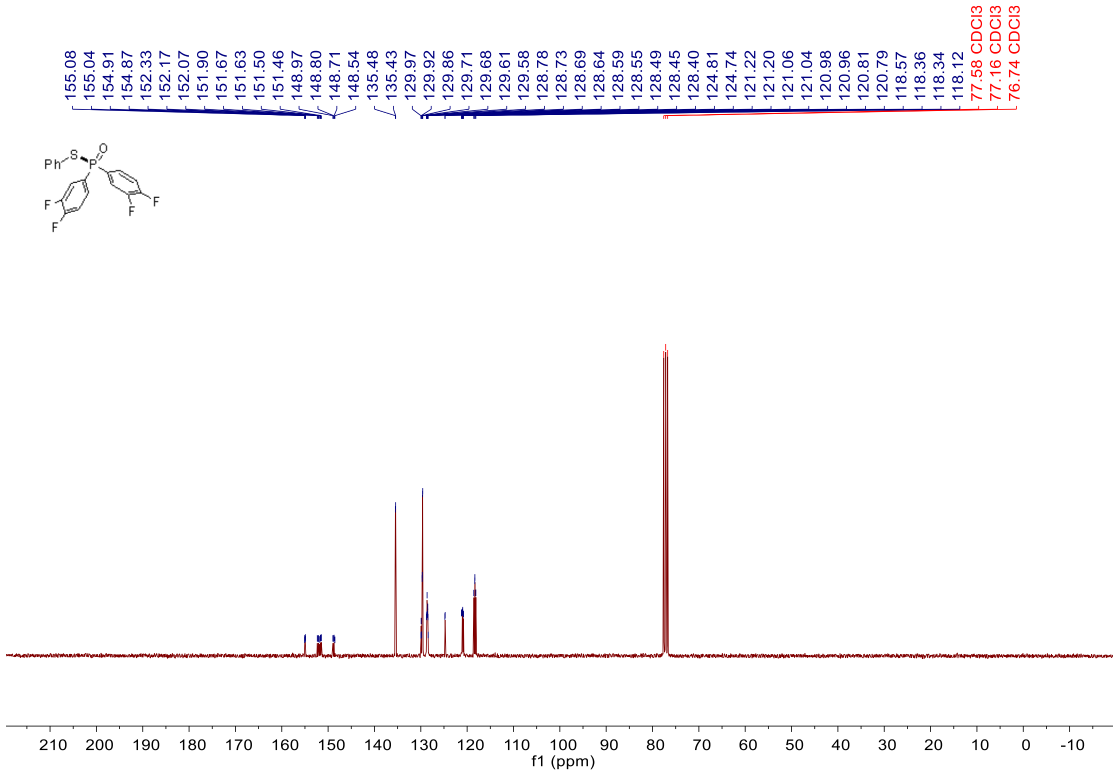

Figure S62. ^{13}C NMR spectra in $CDCl_3$ for Compound **3t**

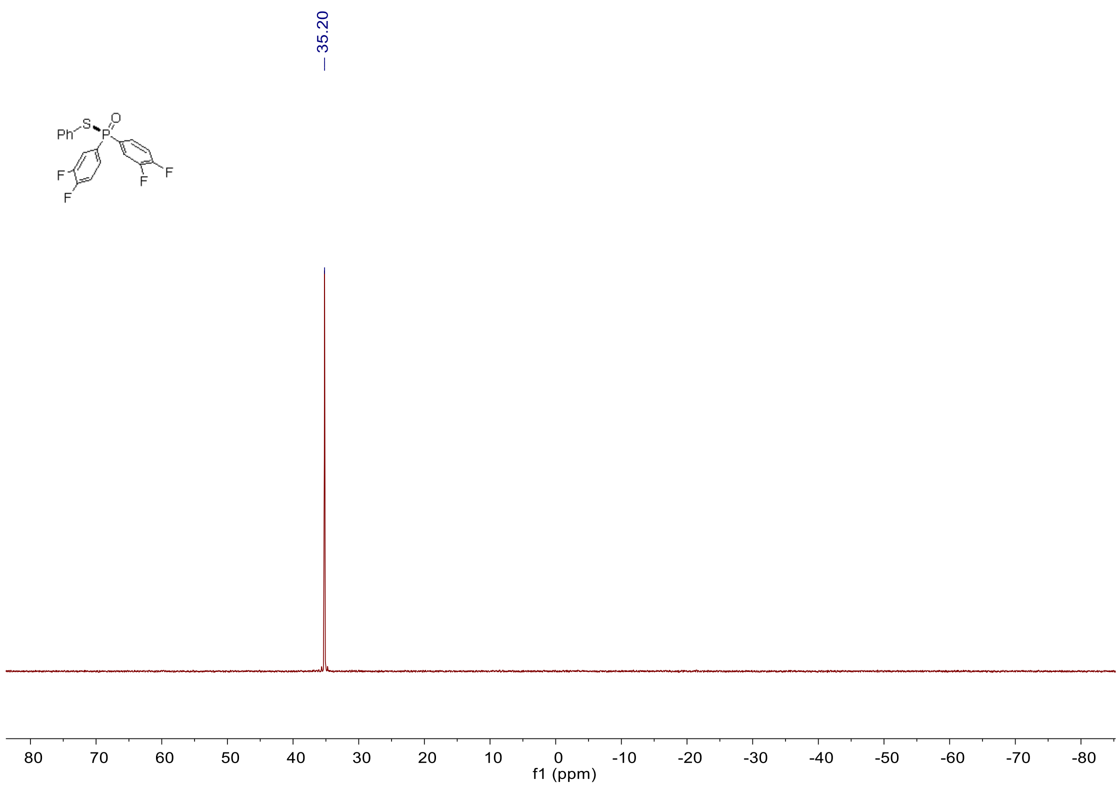

Figure S63. ^{31}P NMR spectra in $CDCl_3$ for Compound **3t**

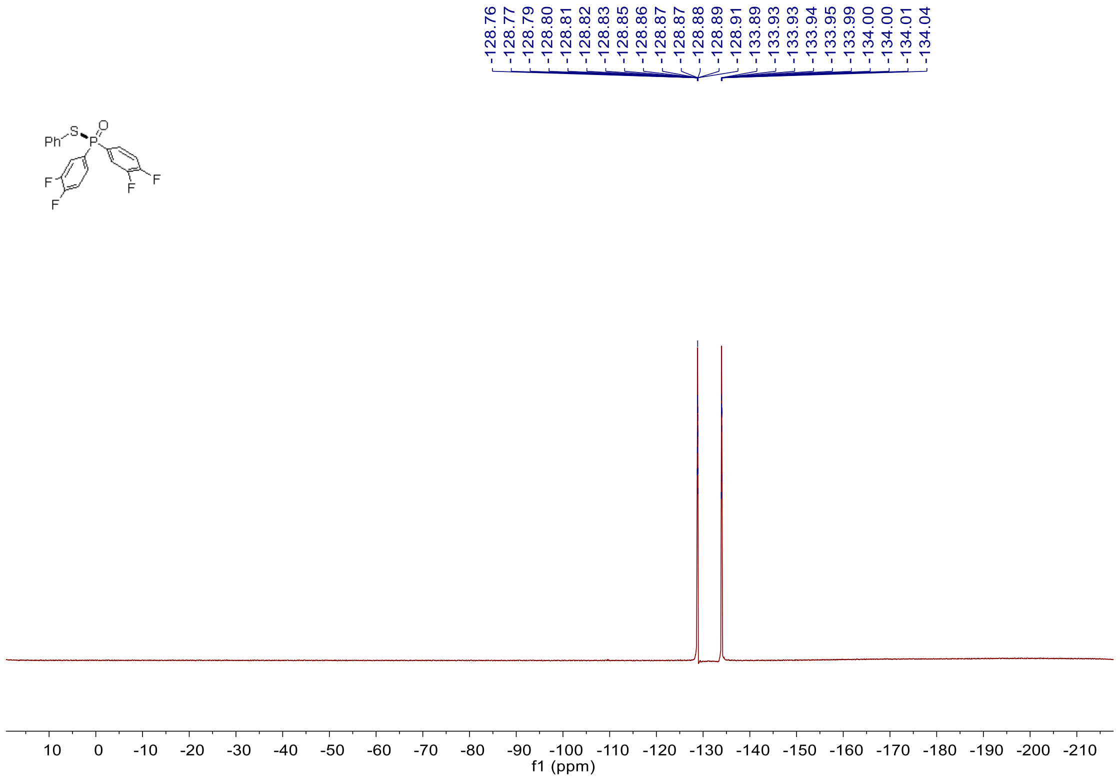

Figure S64. ^{19}F NMR spectra in $CDCl_3$ for Compound **3t**

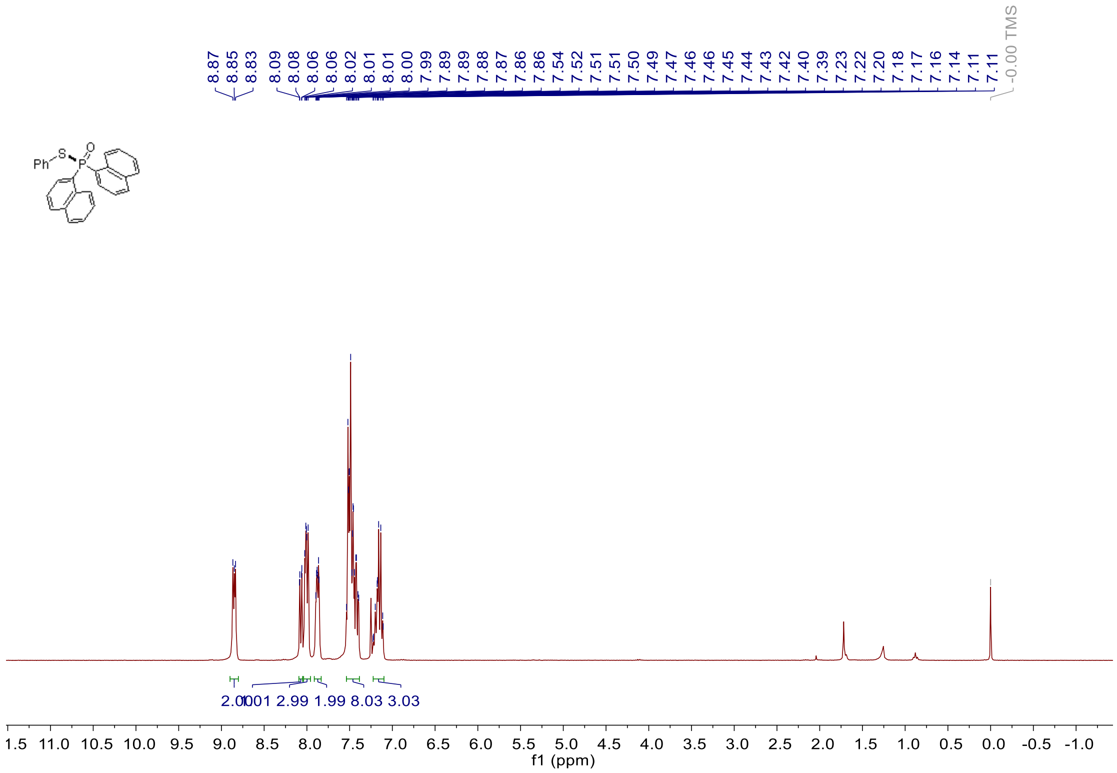

Figure S65. ^{1}H NMR spectra in $CDCl_3$ for Compound **3u**

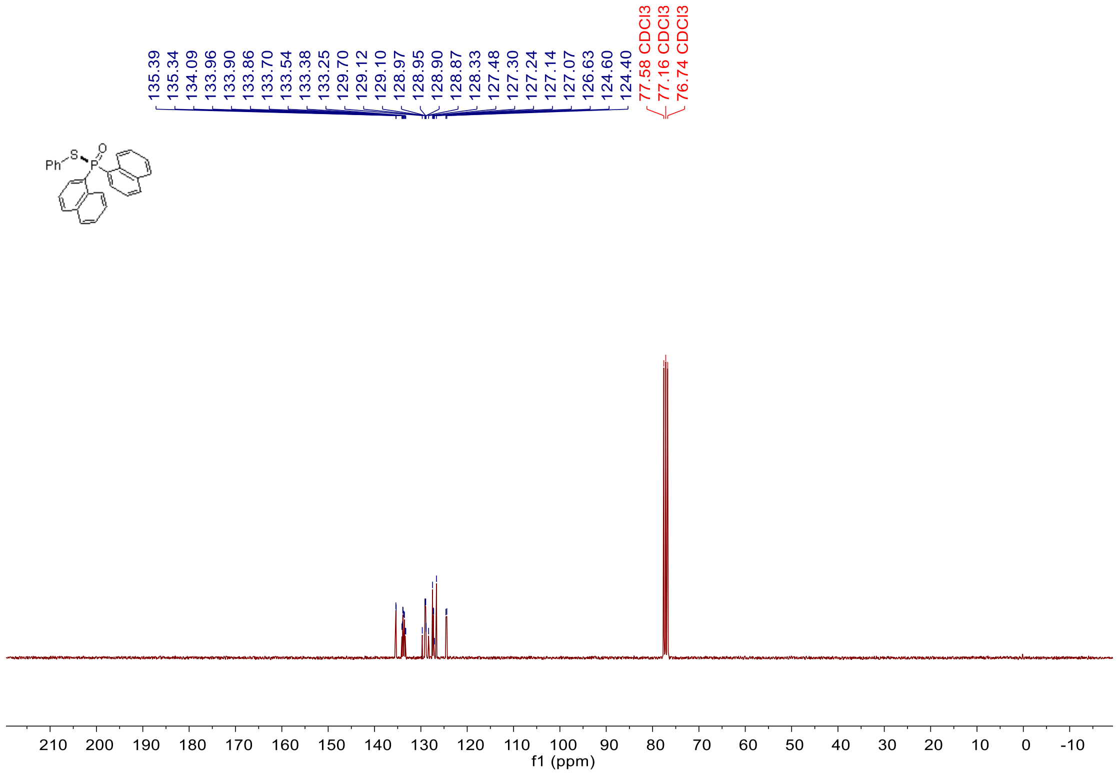

Figure S66. ^{13}C NMR spectra in $CDCl_3$ for Compound **3u**

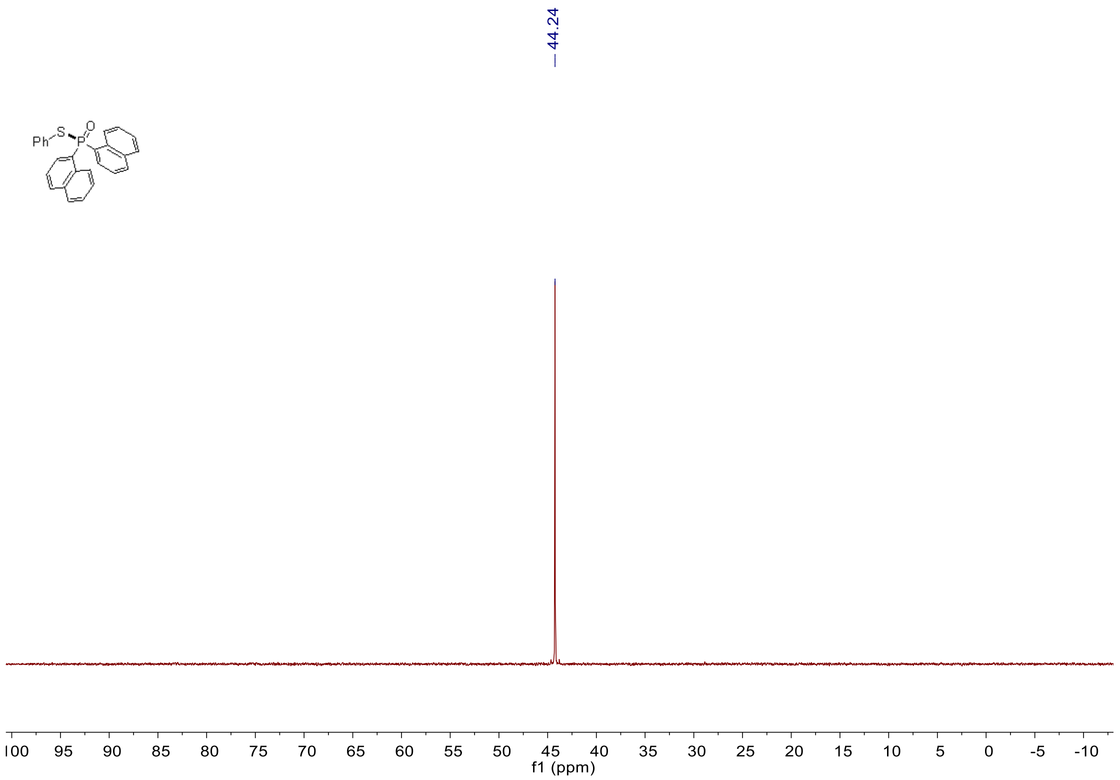

Figure S67. ^{31}P NMR spectra in $CDCl_3$ for Compound **3u**

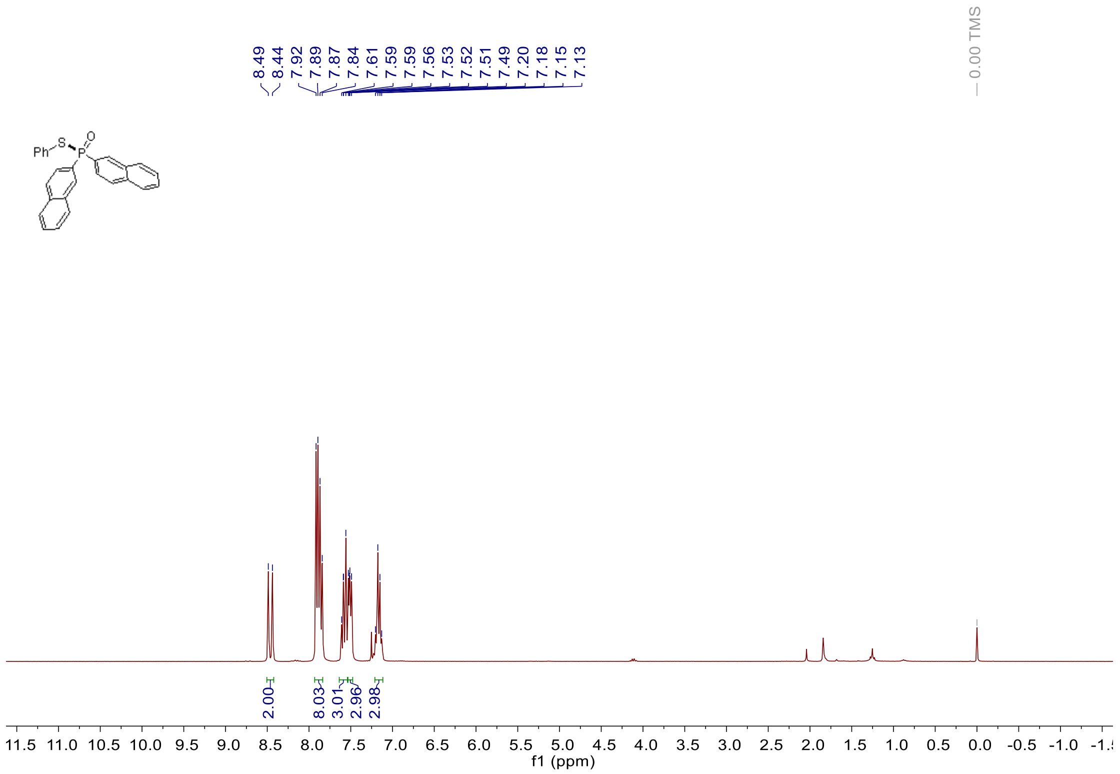

Figure S68. ^1H NMR spectra in $CDCl_3$ for Compound **3v**

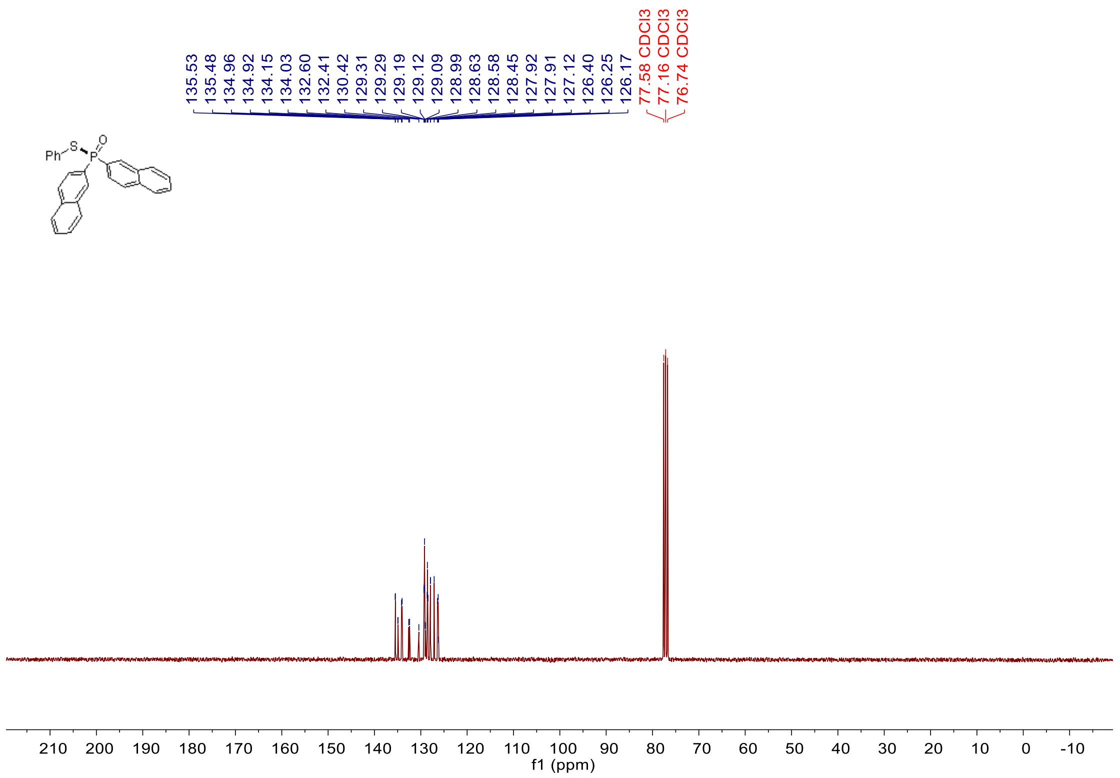

Figure S69. ^{13}C NMR spectra in $CDCl_3$ for Compound **3v**

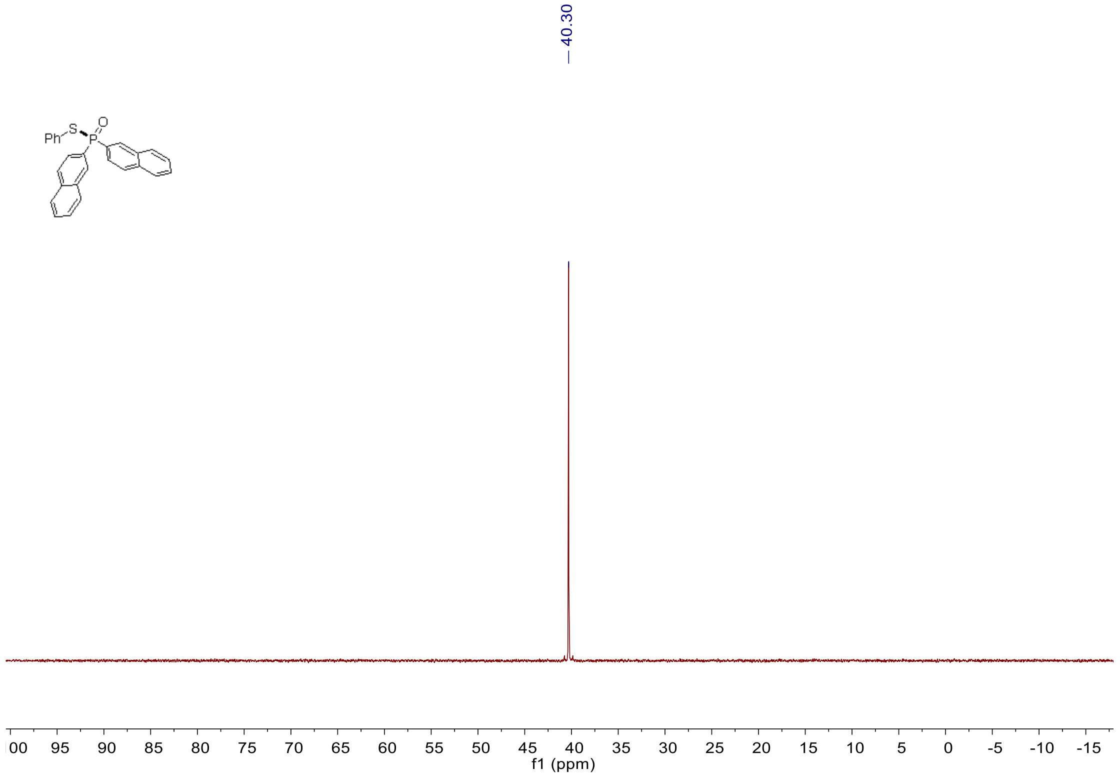

Figure S70. ^{31}P NMR spectra in $CDCl_3$ for Compound **3v**

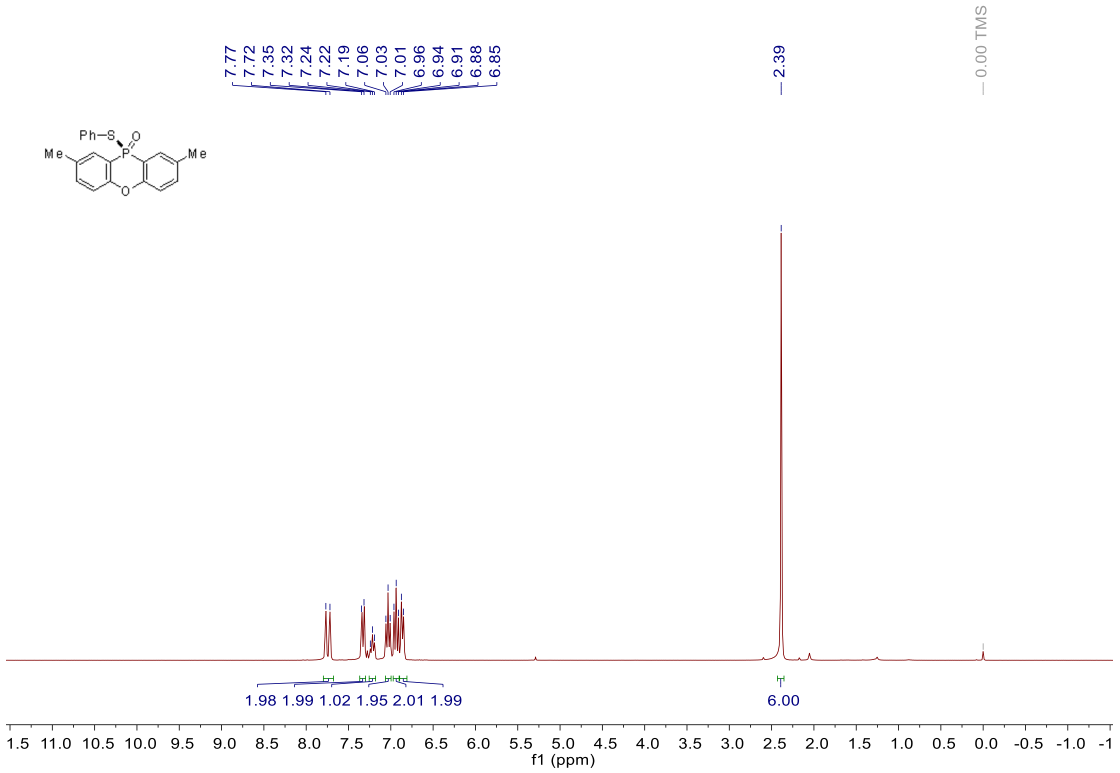

Figure S71. ^{1}H NMR spectra in $CDCl_3$ for Compound **3w**

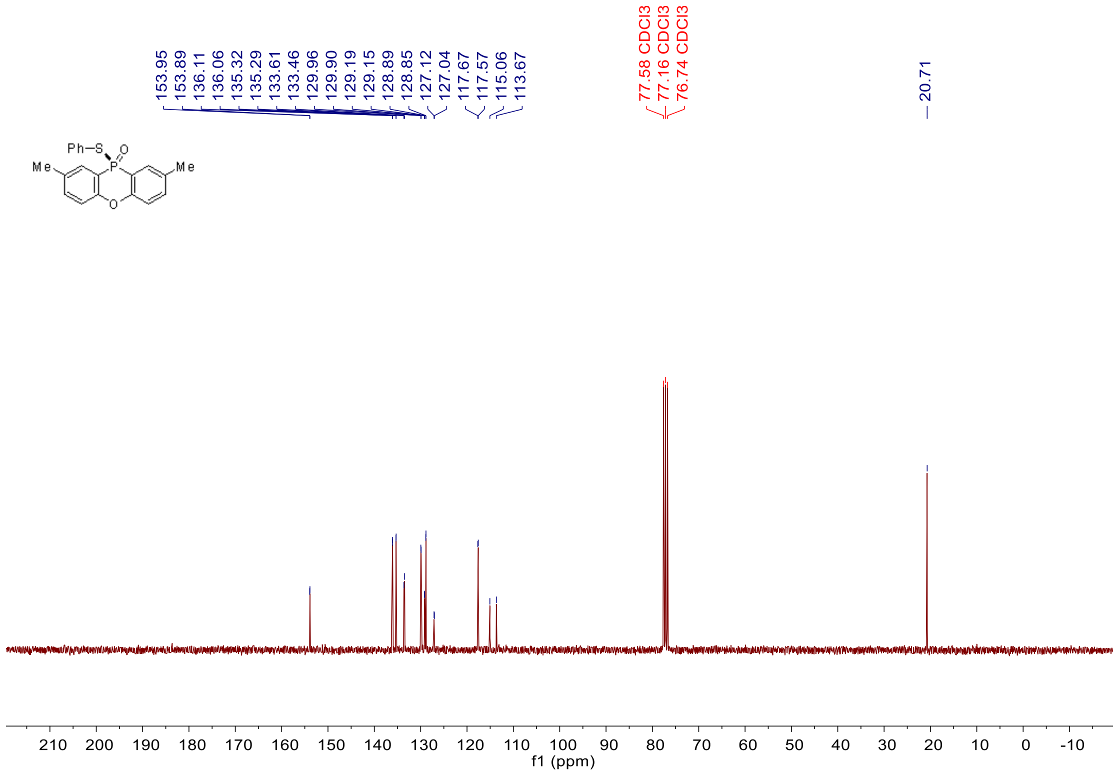

Figure S72. ^{13}C NMR spectra in $CDCl_3$ for Compound **3w**

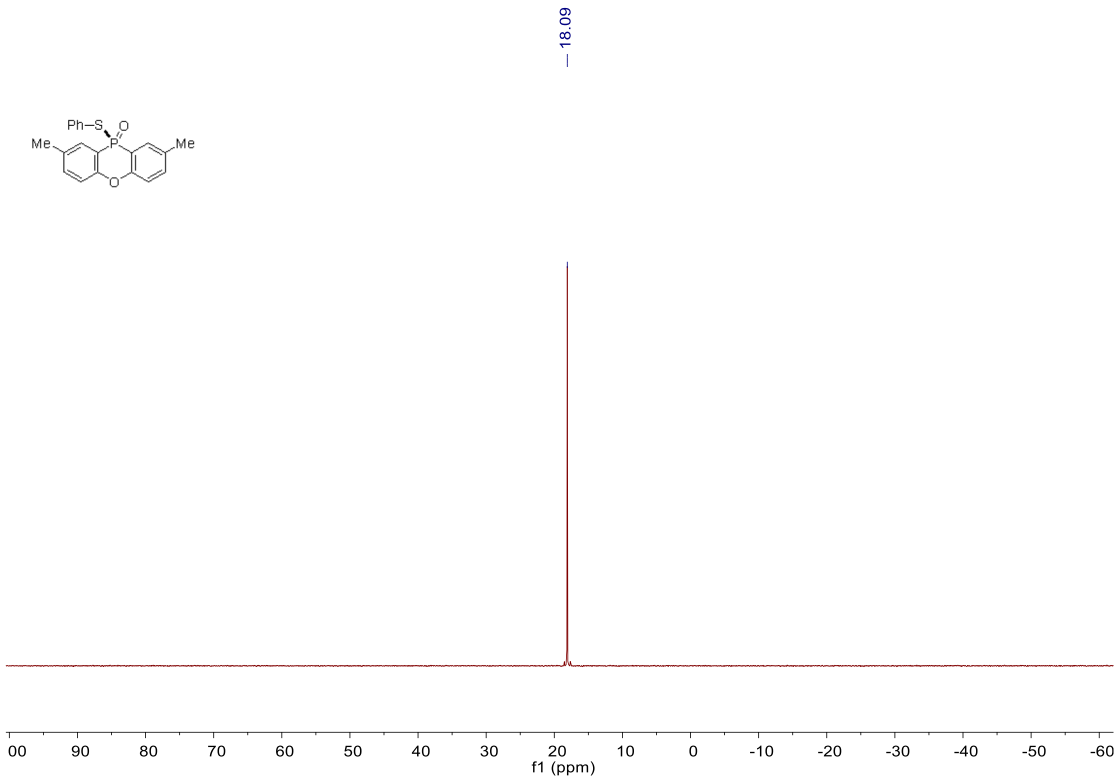

Figure S73. ^{31}P NMR spectra in $CDCl_3$ for Compound **3w**

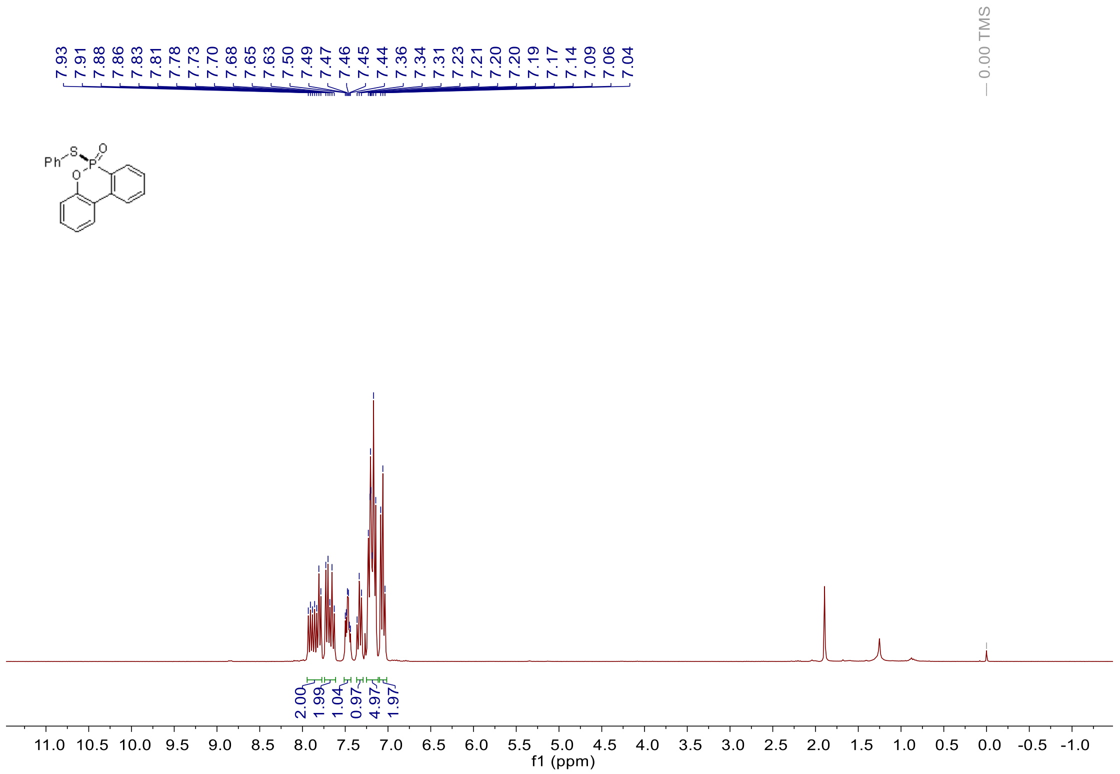

Figure S74. ^{1}H NMR spectra in $CDCl_3$ for Compound **4a**

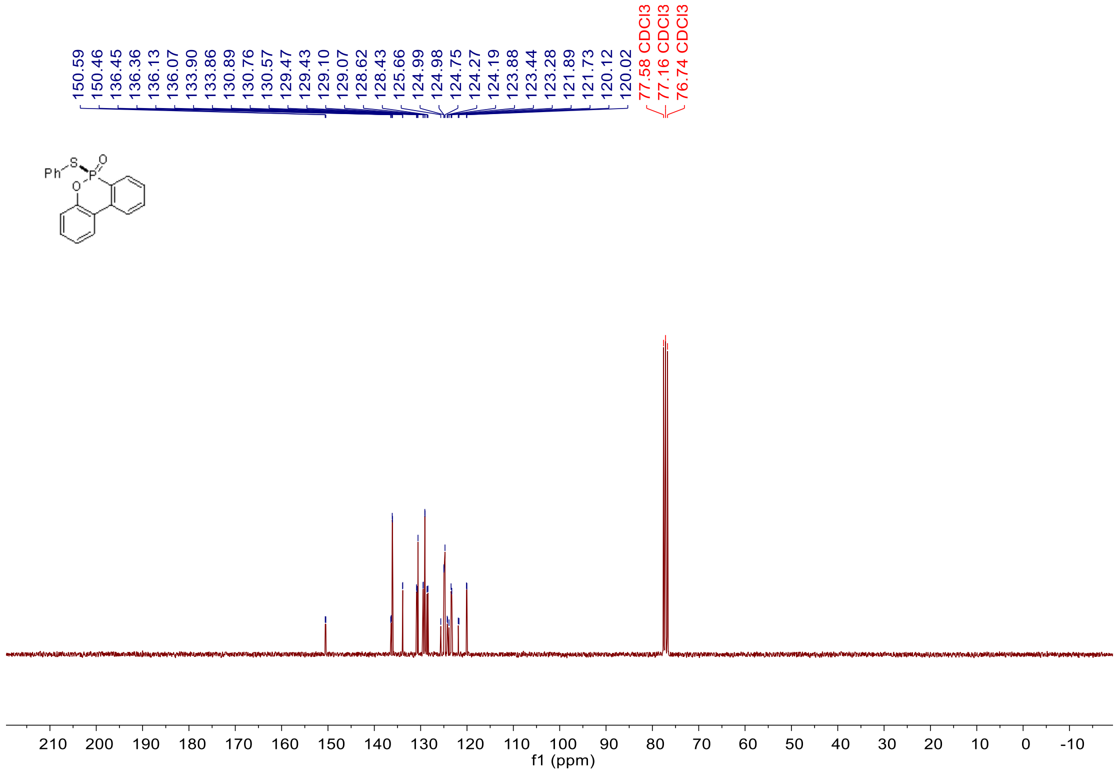

Figure S75. ^{13}C NMR spectra in $CDCl_3$ for Compound **4a**

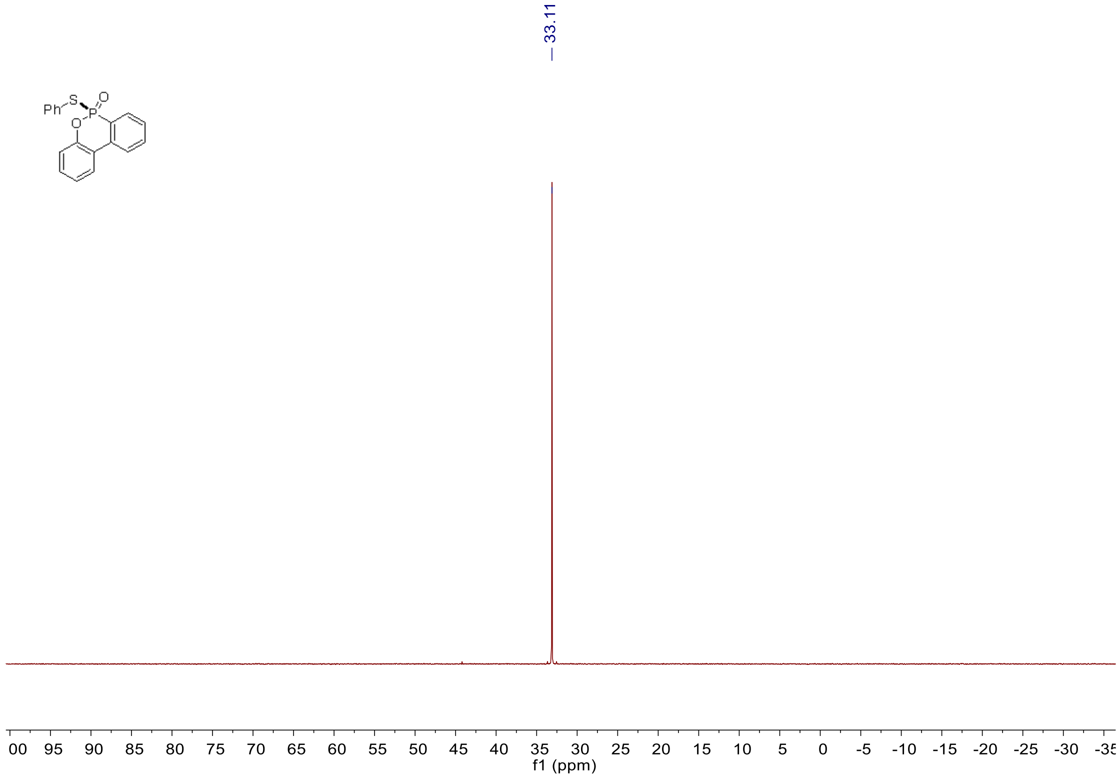

Figure S76. ^{31}P NMR spectra in $CDCl_3$ for Compound **4a**

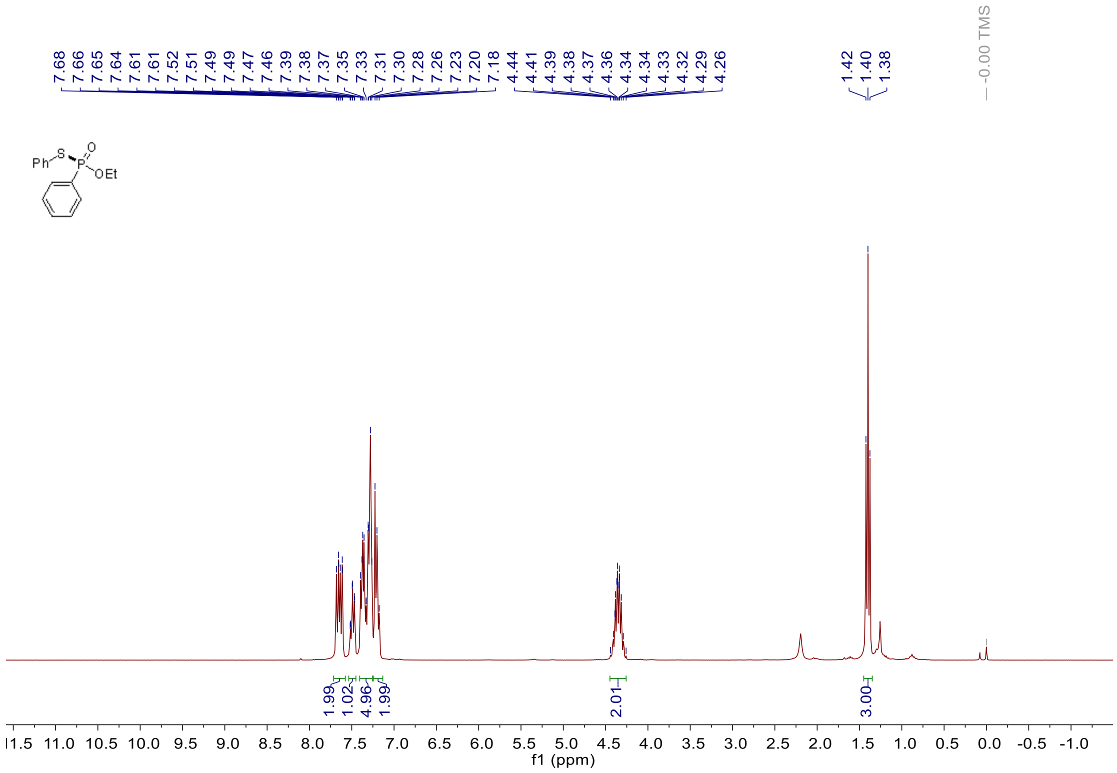

Figure S77. ^{1}H NMR spectra in $CDCl_3$ for Compound **4b**

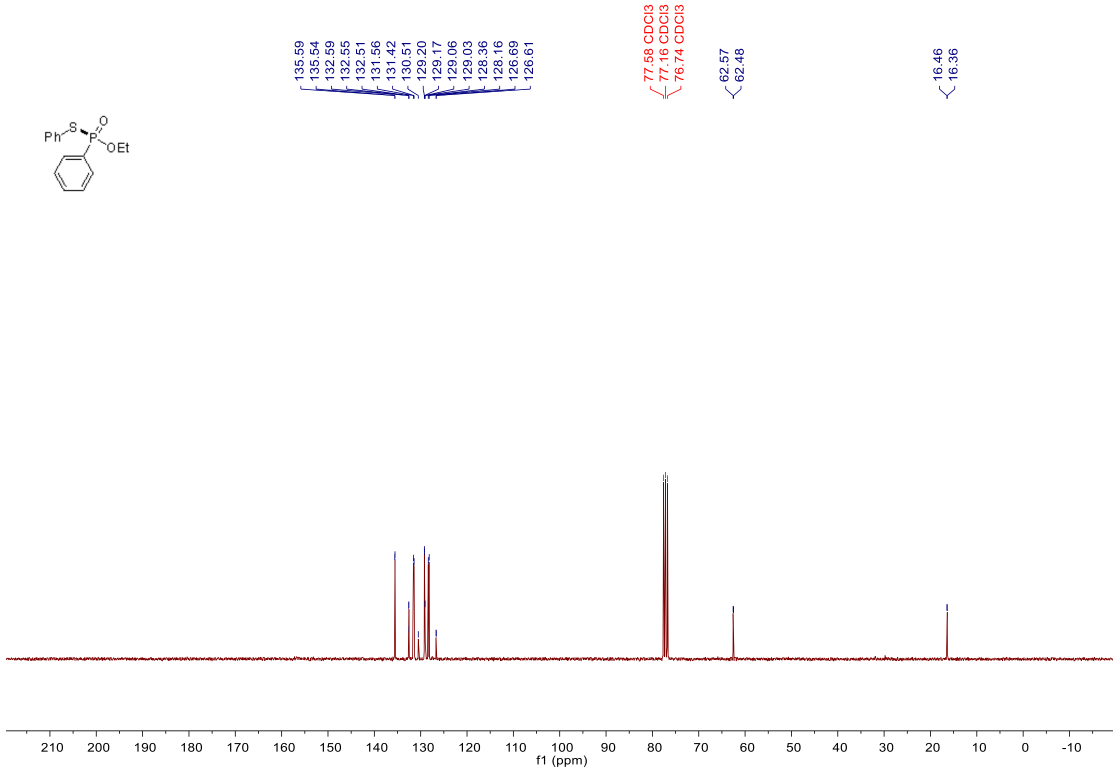

Figure S78. ^{13}C NMR spectra in $CDCl_3$ for Compound **4b**

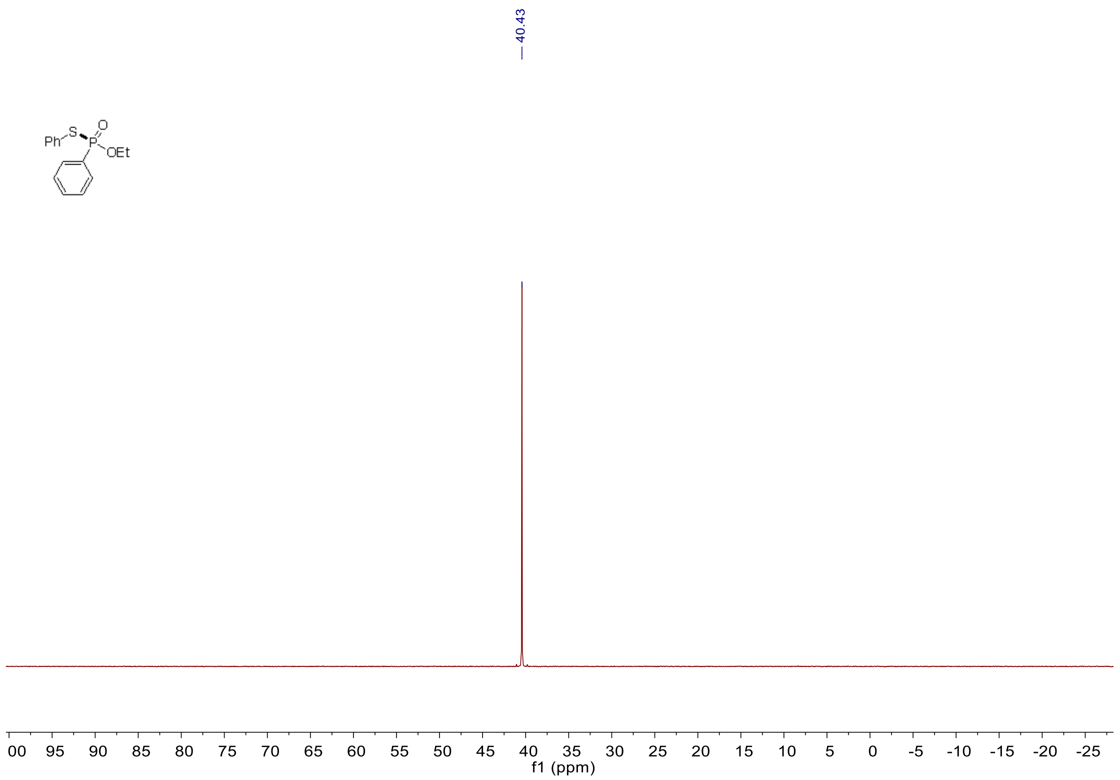

Figure S79. ^{31}P NMR spectra in $CDCl_3$ for Compound **4b**

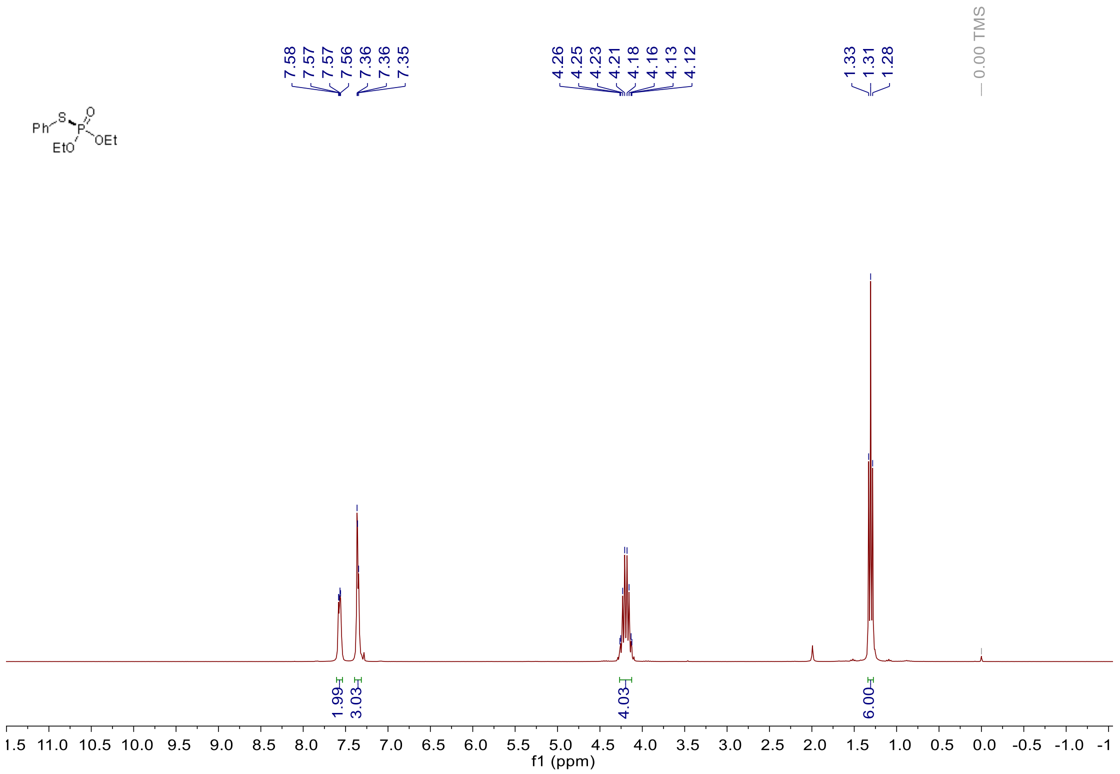

Figure S80. ^{1}H NMR spectra in $CDCl_3$ for Compound **4c**

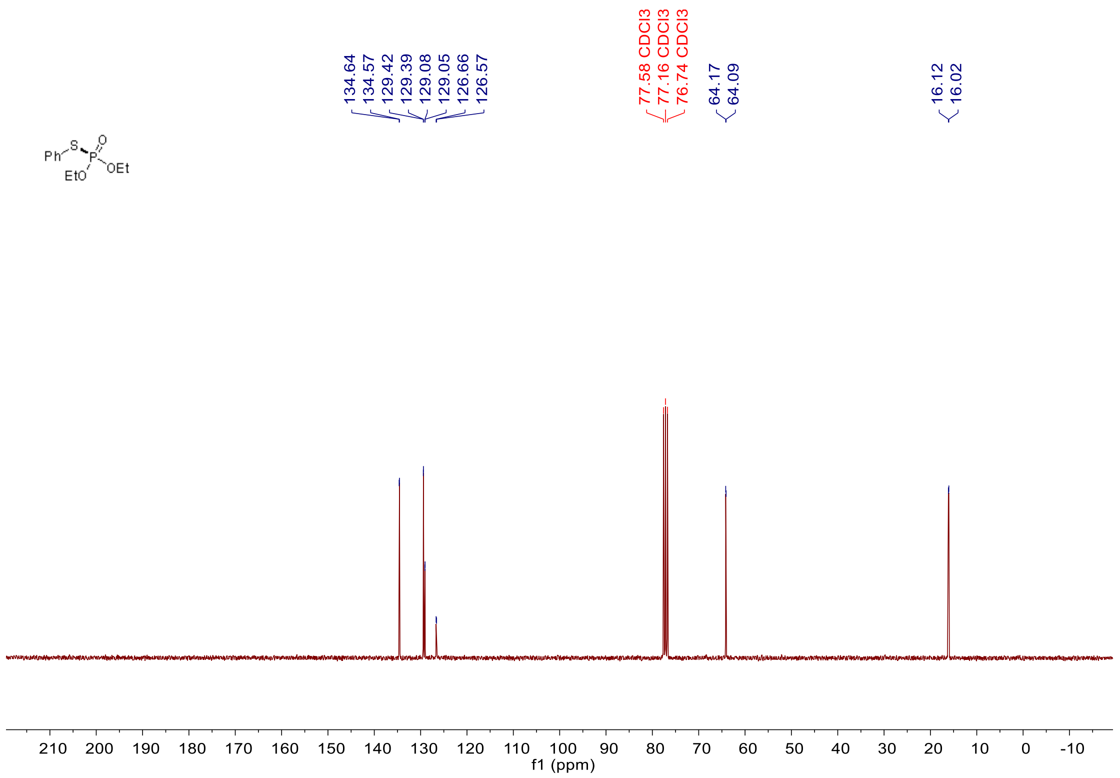

Figure S81. ^{13}C NMR spectra in $CDCl_3$ for Compound **4c**

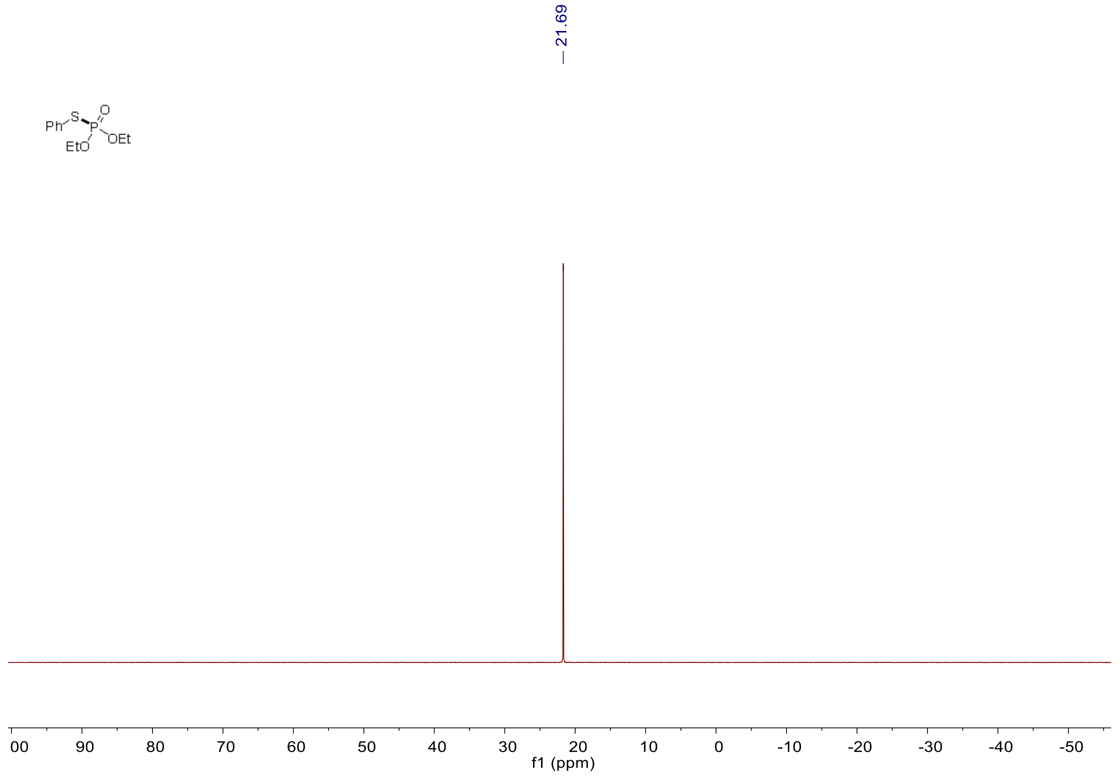

Figure S82. ^{31}P NMR spectra in $CDCl_3$ for Compound **4c**

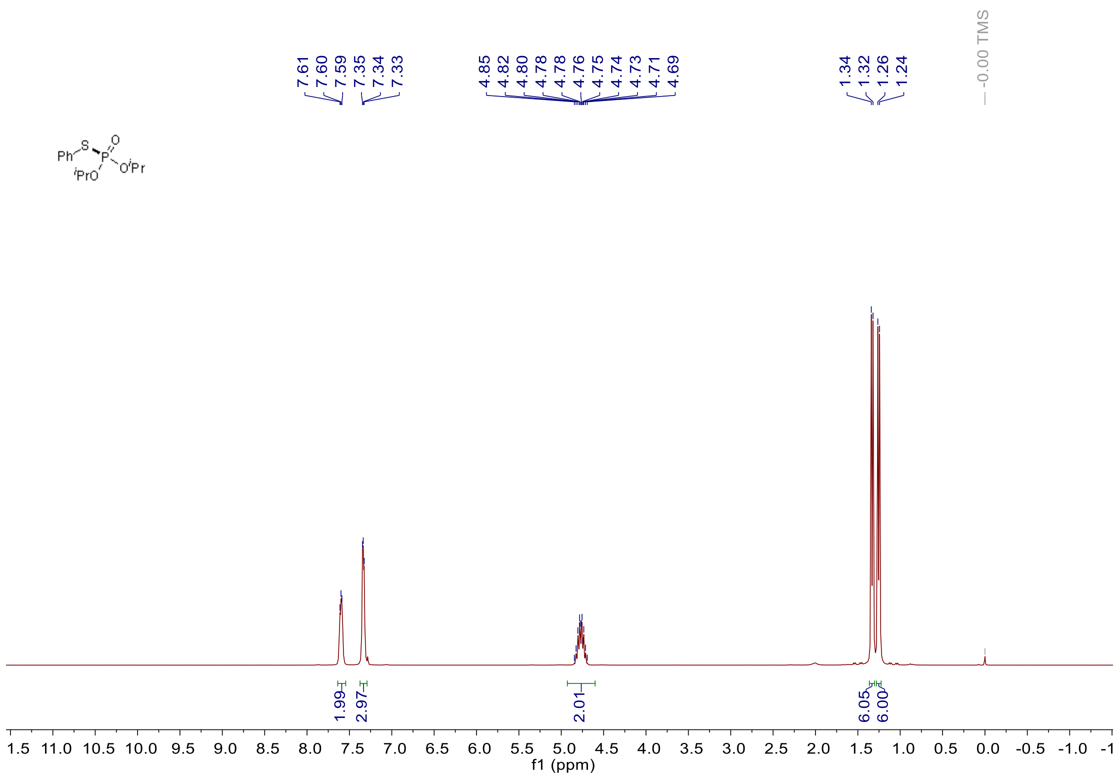

Figure S83. ^{1}H NMR spectra in $CDCl_3$ for Compound **4d**

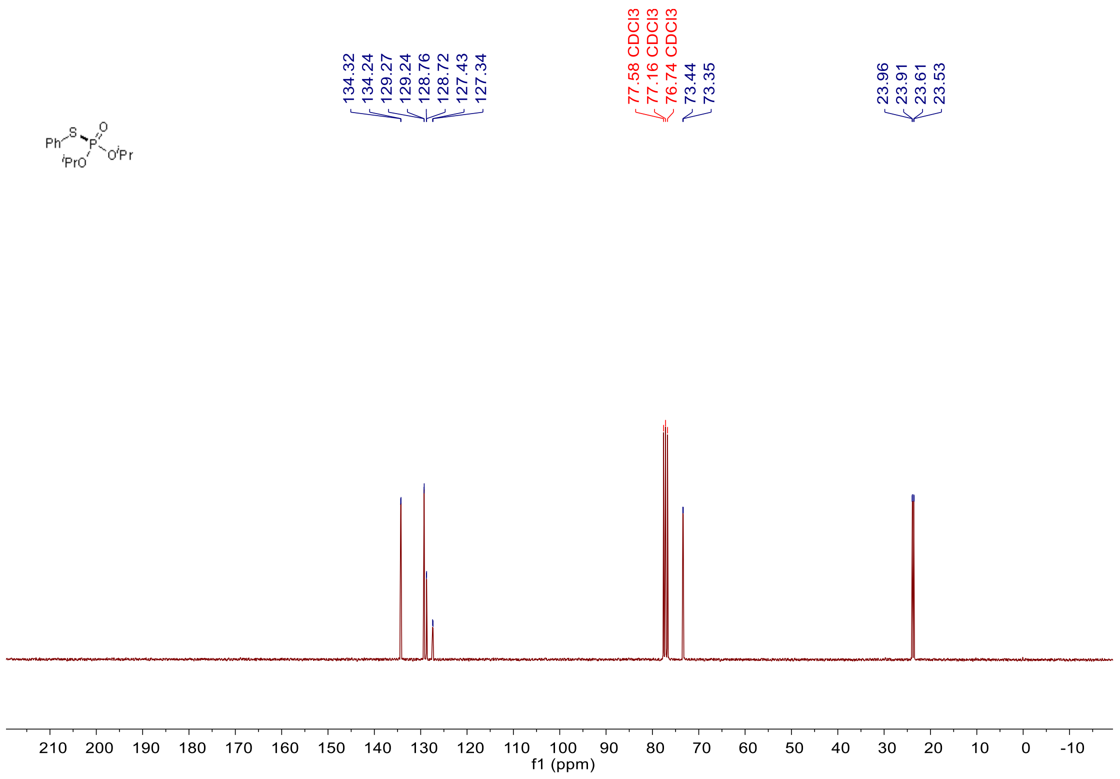

Figure S84. ^{13}C NMR spectra in $CDCl_3$ for Compound **4d**

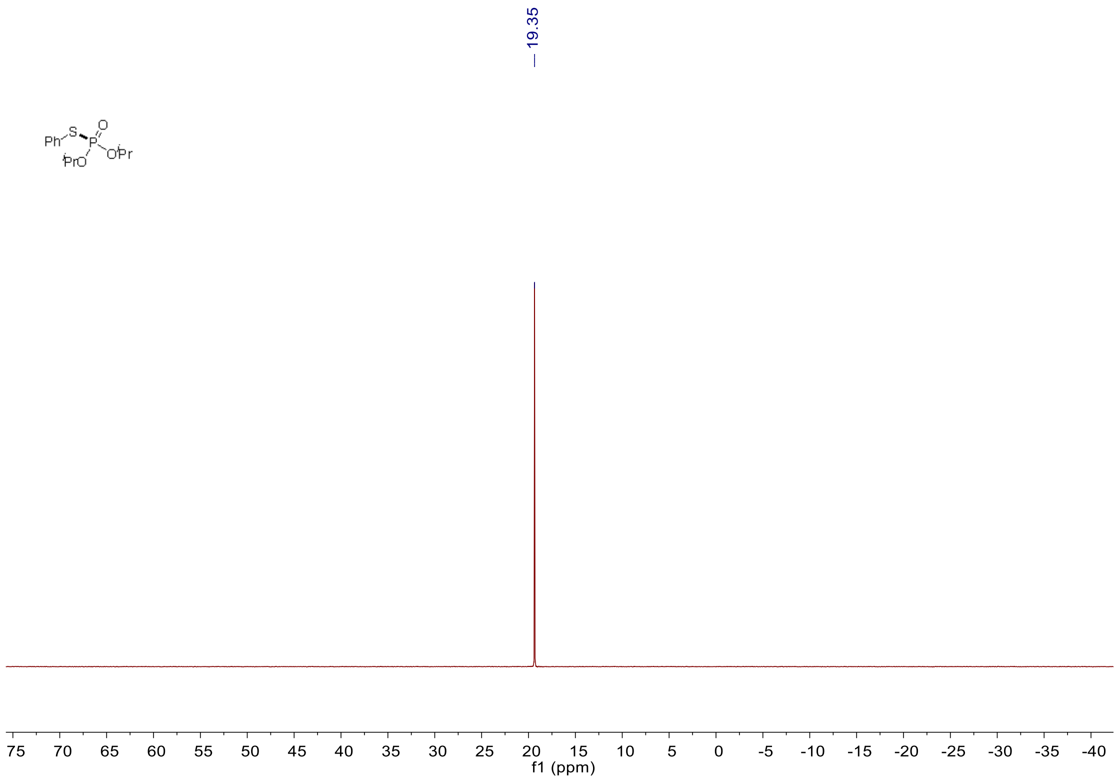

Figure S85. ^{31}P NMR spectra in $CDCl_3$ for Compound **4d**

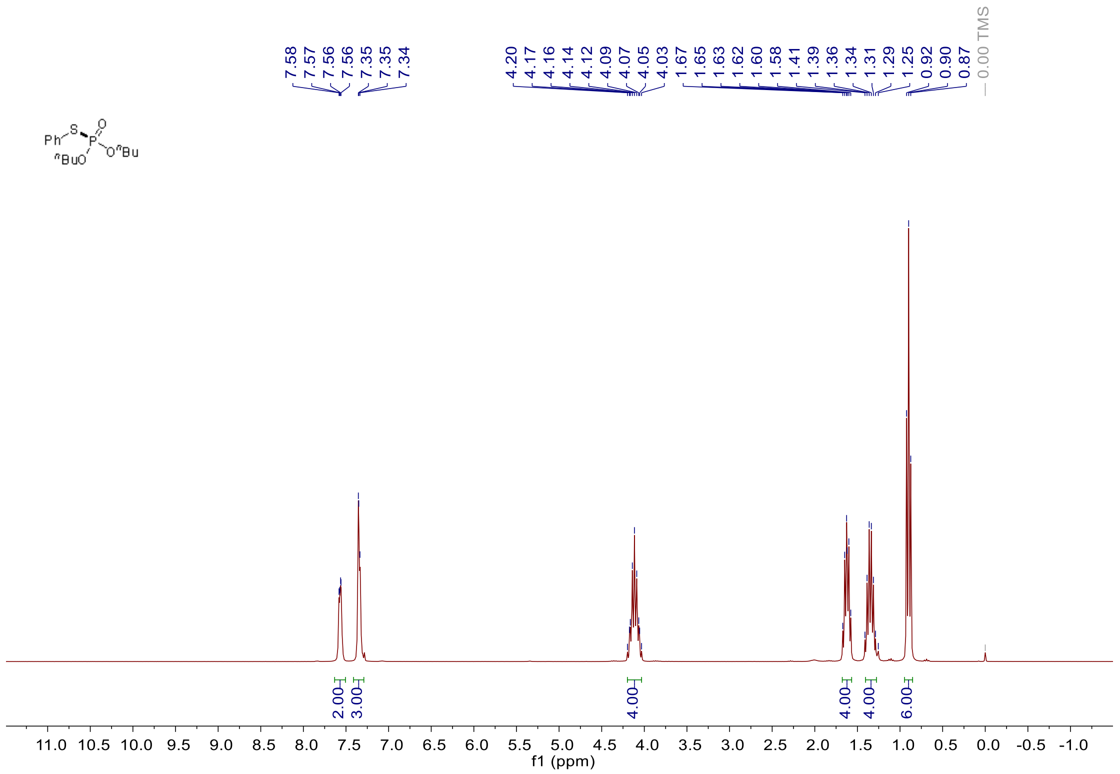

Figure S86. ^{1}H NMR spectra in $CDCl_3$ for Compound **4e**

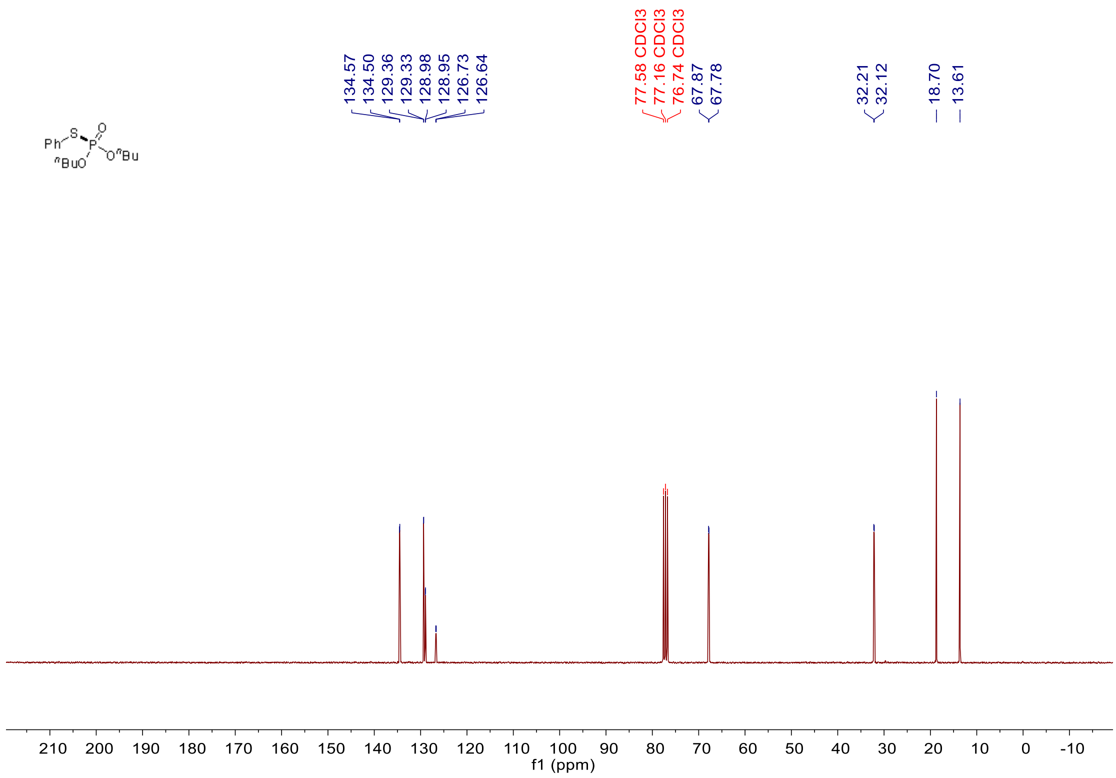

Figure S87. ^{13}C NMR spectra in $CDCl_3$ for Compound **4e**

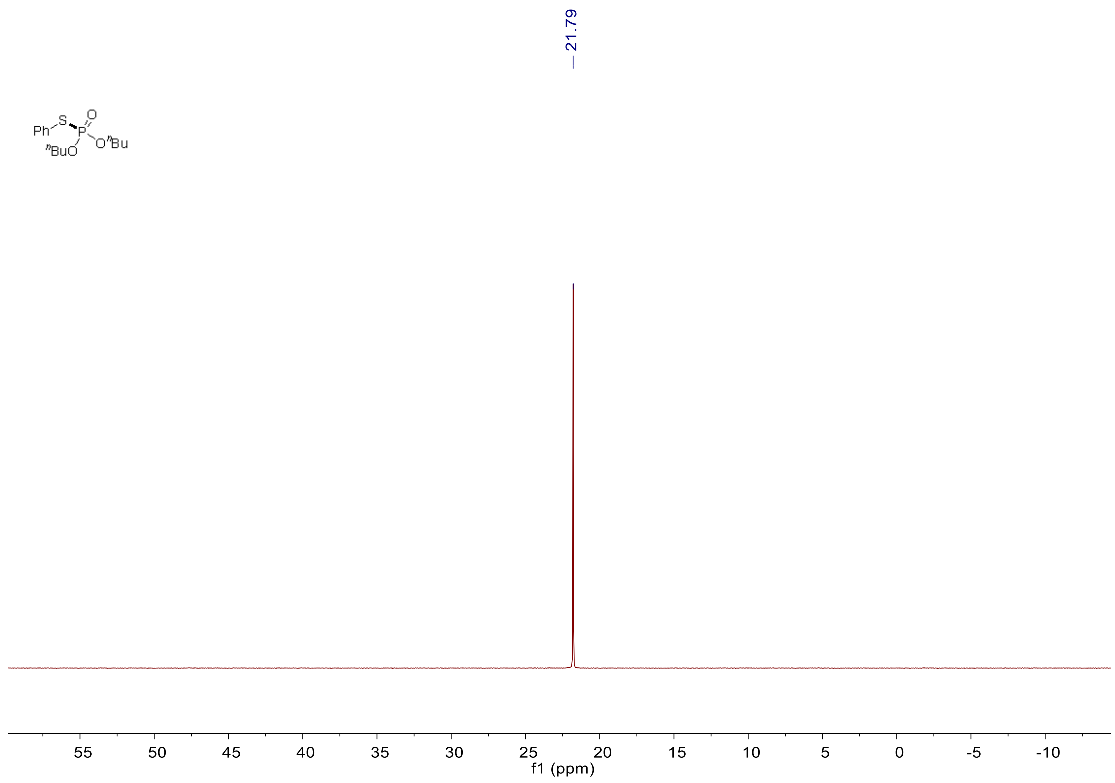

Figure S88. ^{31}P NMR spectra in $CDCl_3$ for Compound **4e**

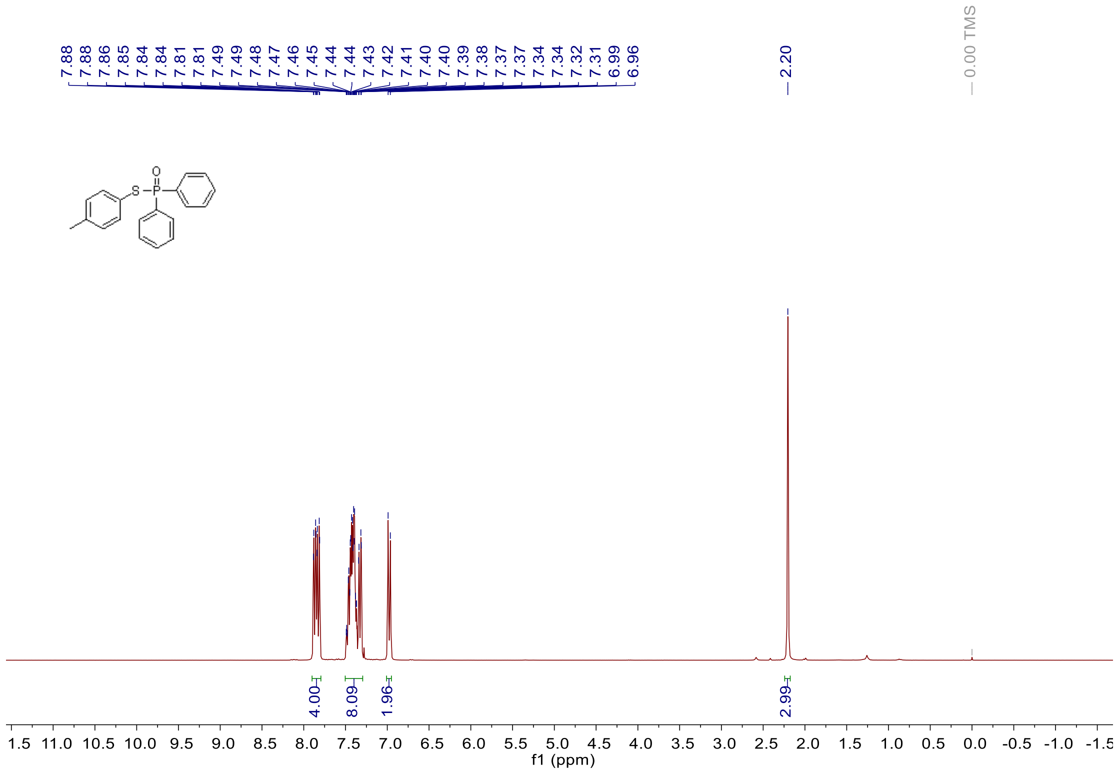

Figure S89. ^{1}H NMR spectra in $CDCl_3$ for Compound **5a**

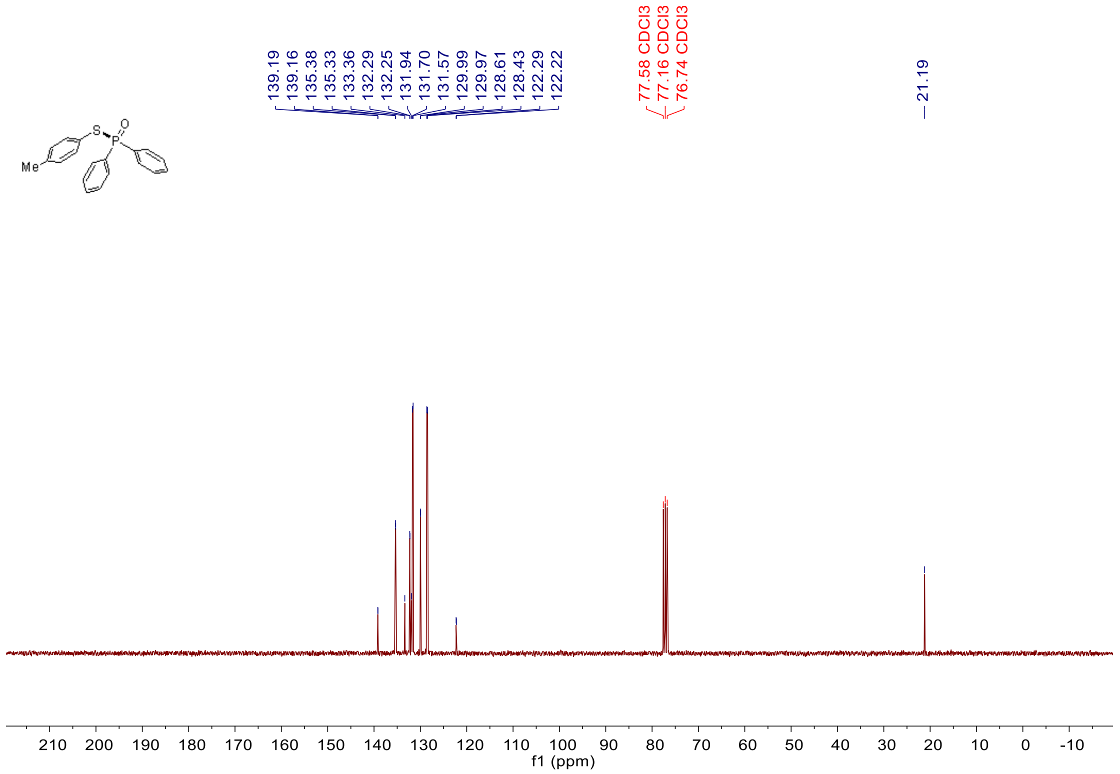

Figure S90. ^{13}C NMR spectra in $CDCl_3$ for Compound **5a**

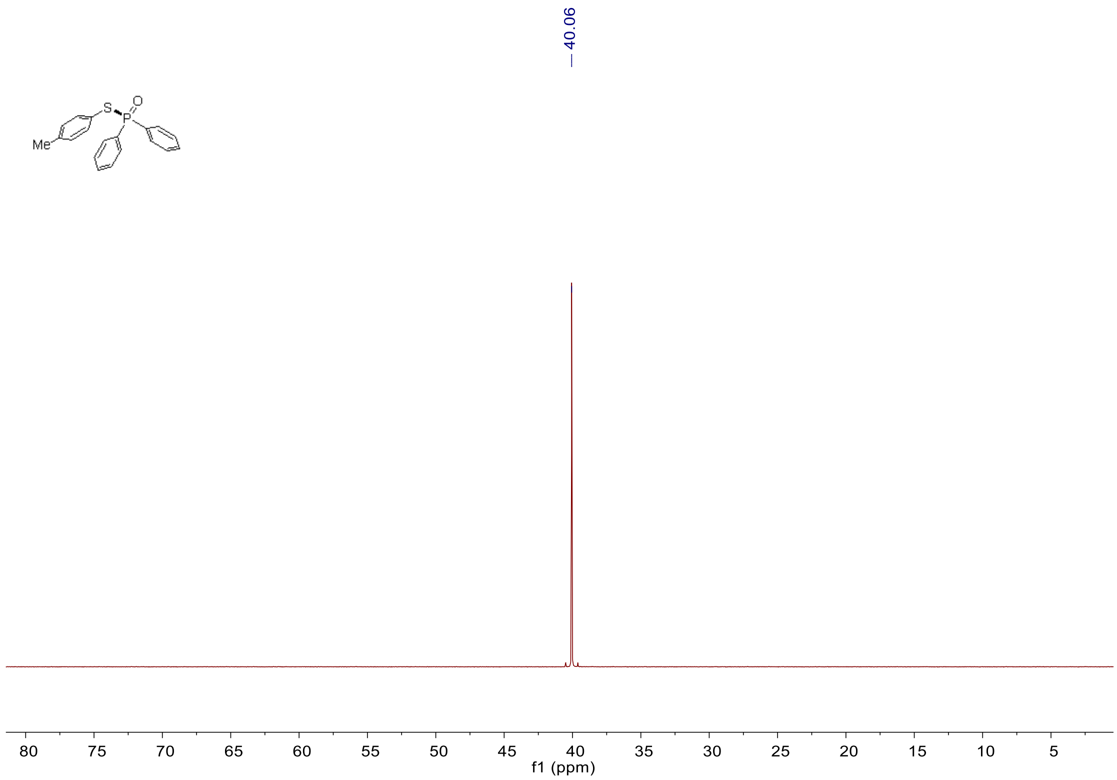

Figure S91. ^{31}P NMR spectra in $CDCl_3$ for Compound **5a**

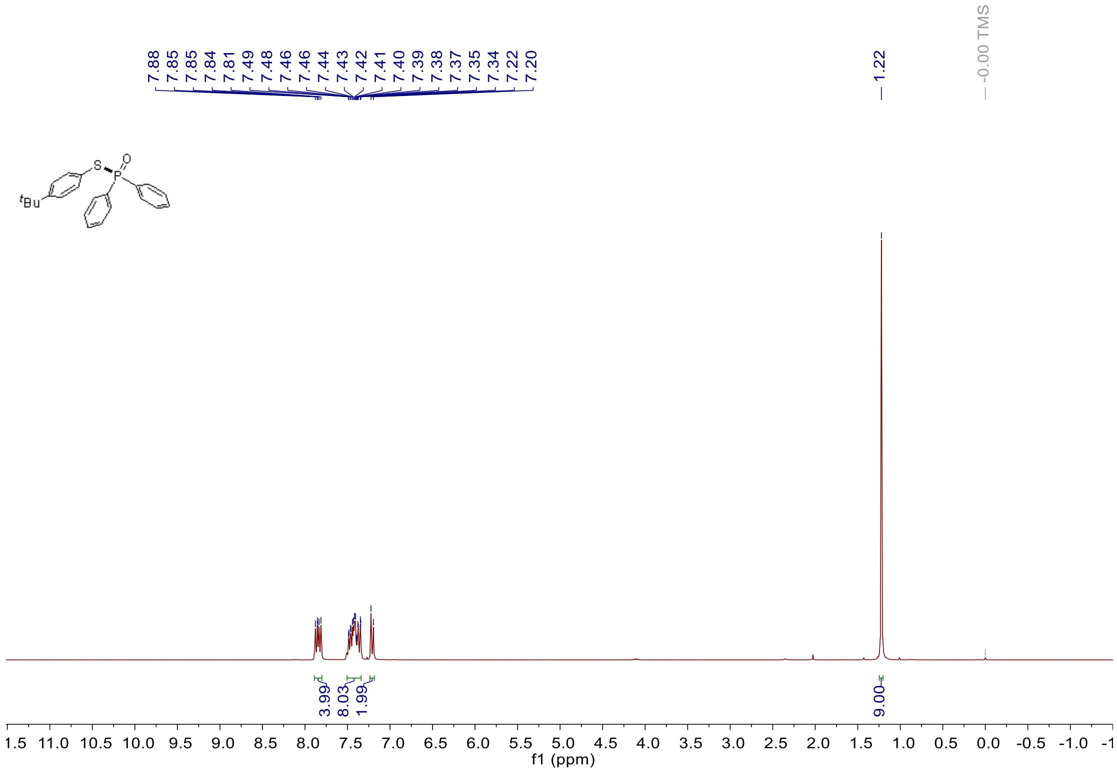

Figure S92. ^{1}H NMR spectra in $CDCl_3$ for Compound **5b**

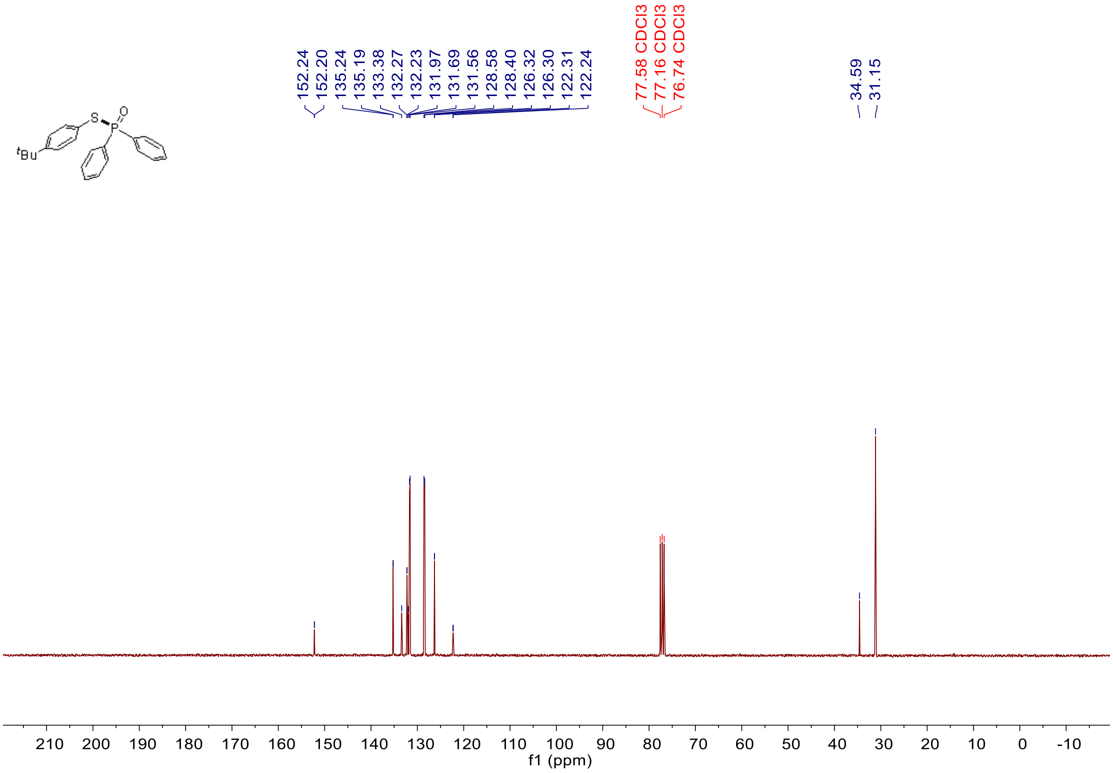

Figure S93. ^{13}C NMR spectra in $CDCl_3$ for Compound **5b**

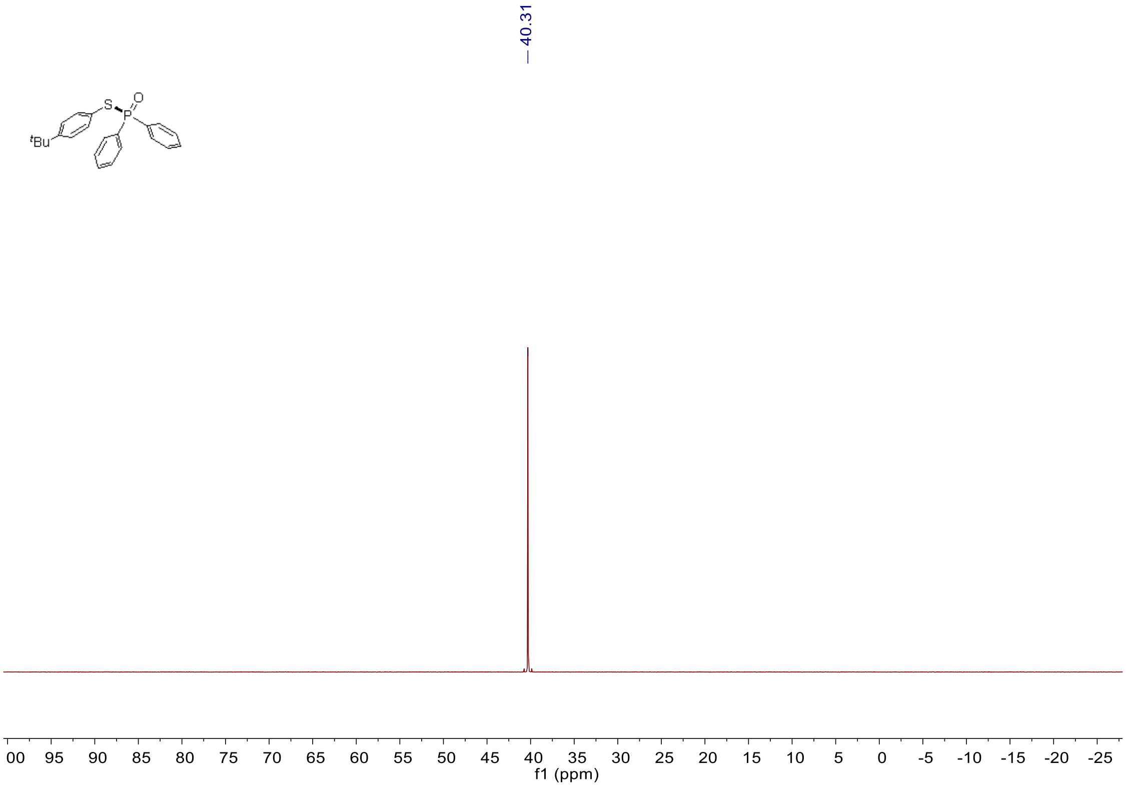

Figure S94. ^{31}P NMR spectra in $CDCl_3$ for Compound **5b**

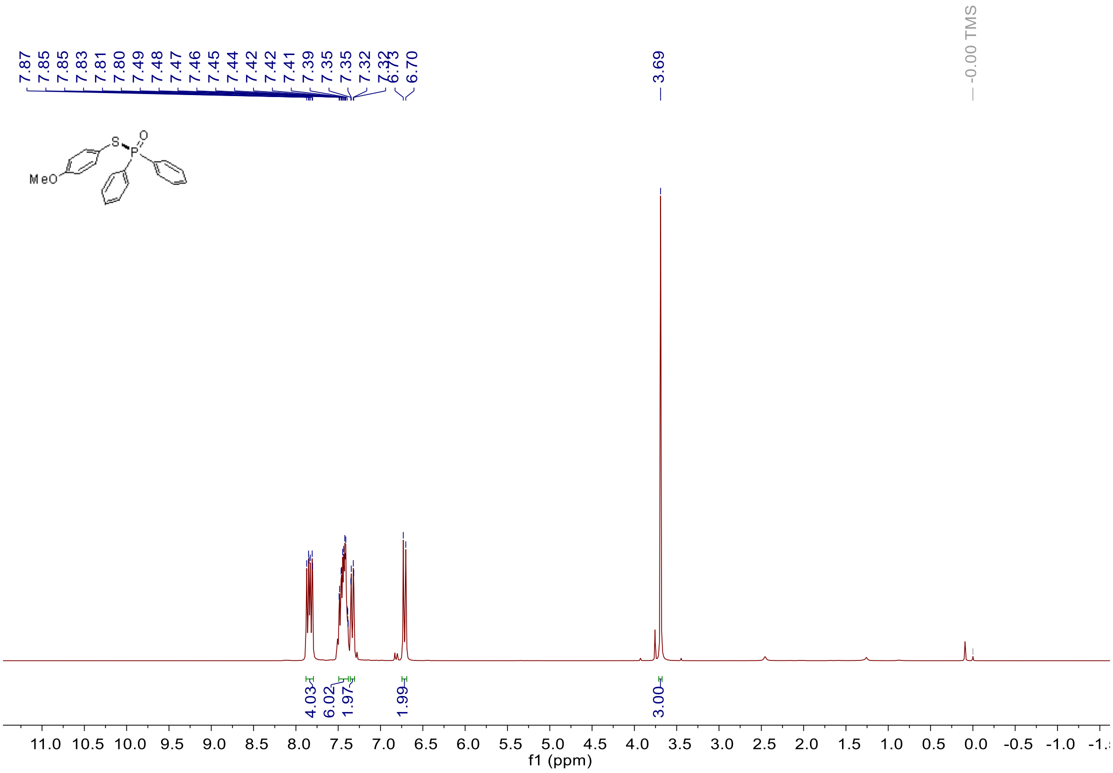

Figure S95. ^{1}H NMR spectra in $CDCl_3$ for Compound **5c**

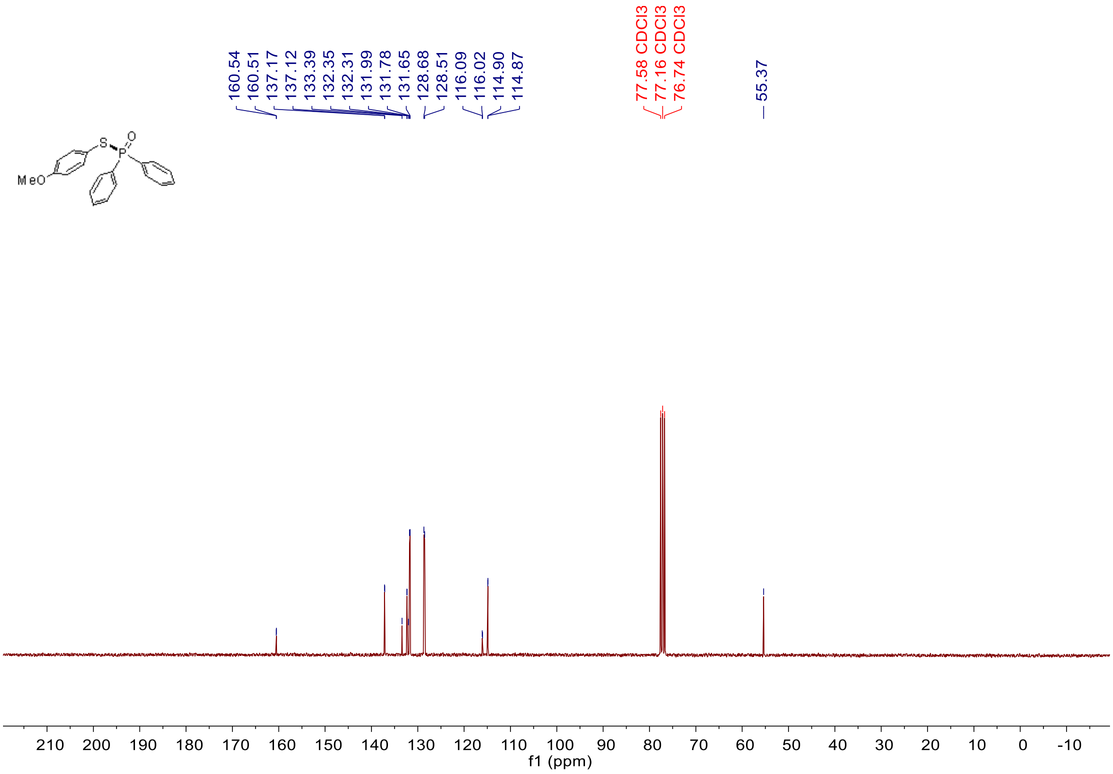

Figure S96. ^{13}C NMR spectra in $CDCl_3$ for Compound **5c**

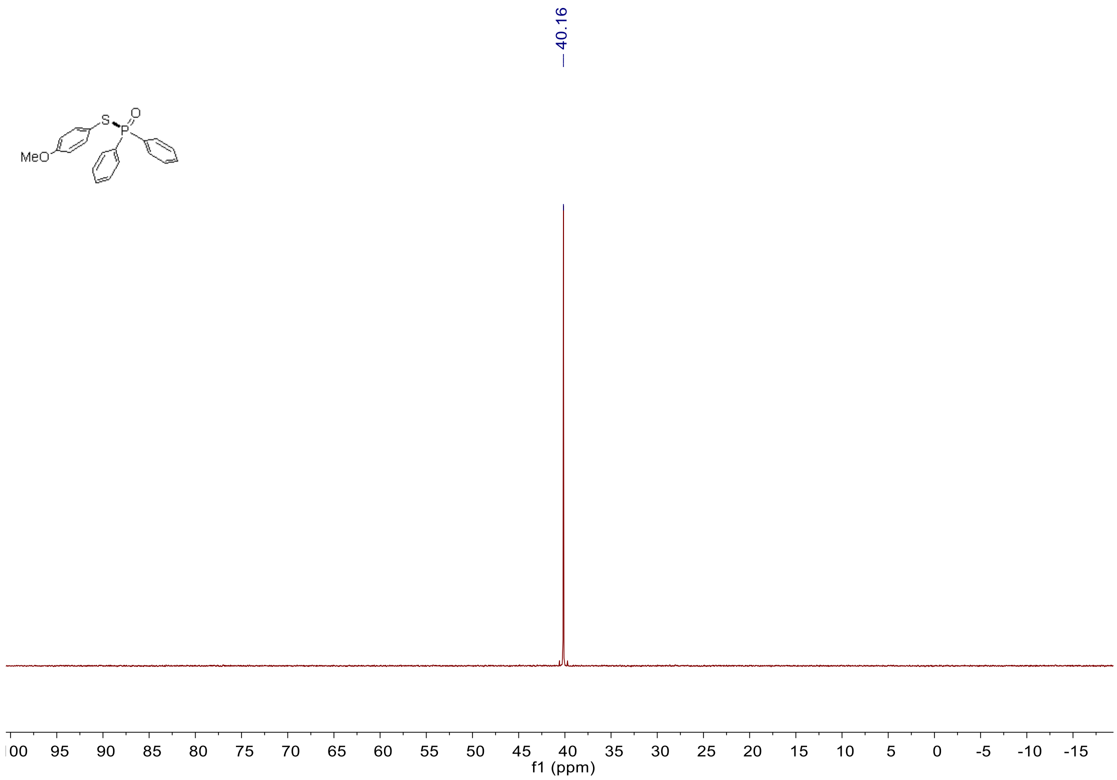

Figure S97. ^{31}P NMR spectra in $CDCl_3$ for Compound **5c**

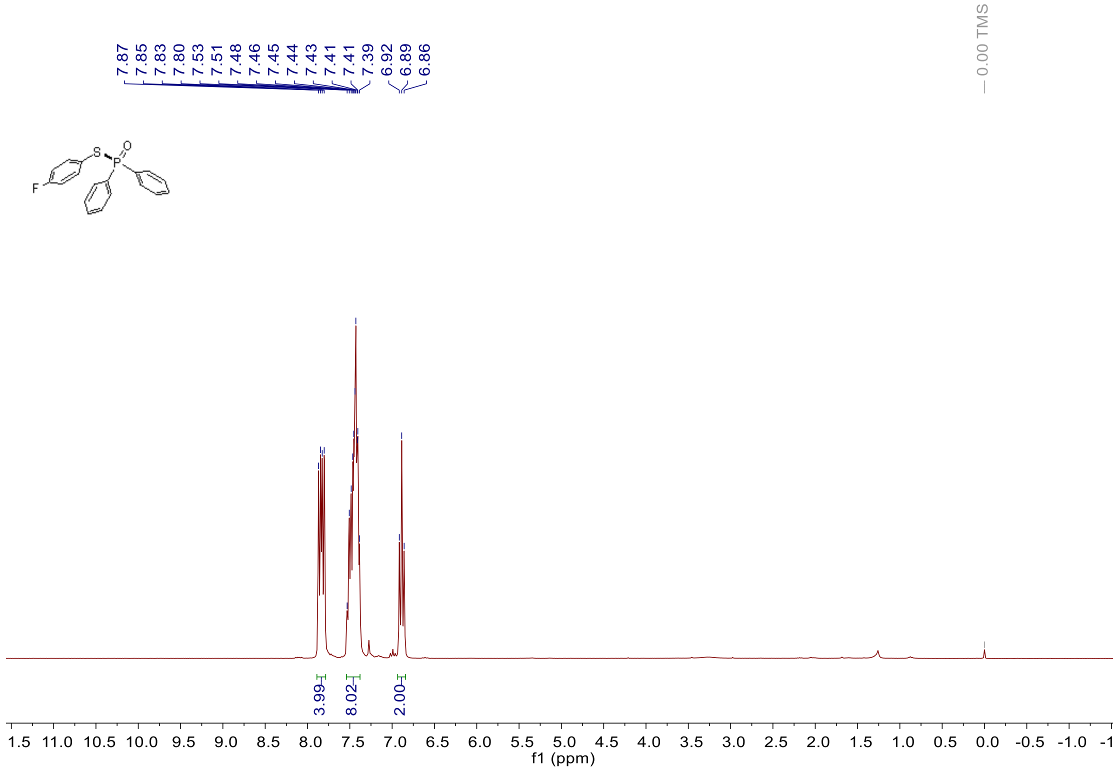

Figure S98. ^{1}H NMR spectra in $CDCl_3$ for Compound **5d**

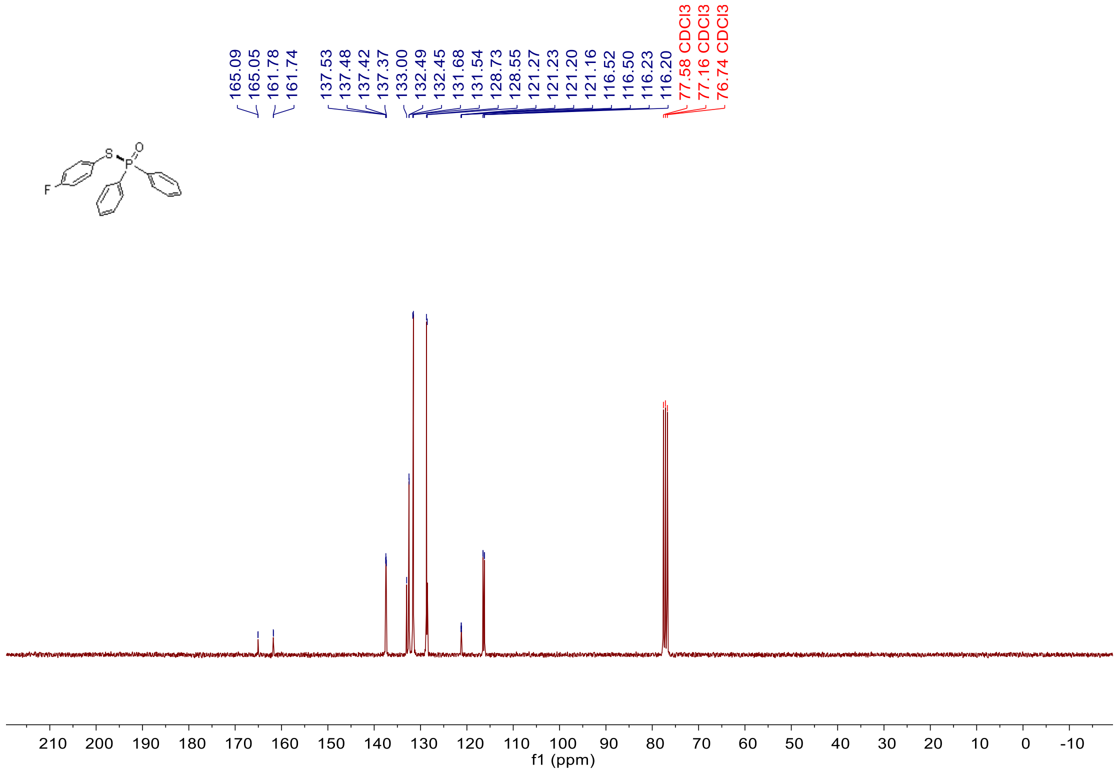

Figure S99. ^{13}C NMR spectra in $CDCl_3$ for Compound **5d**

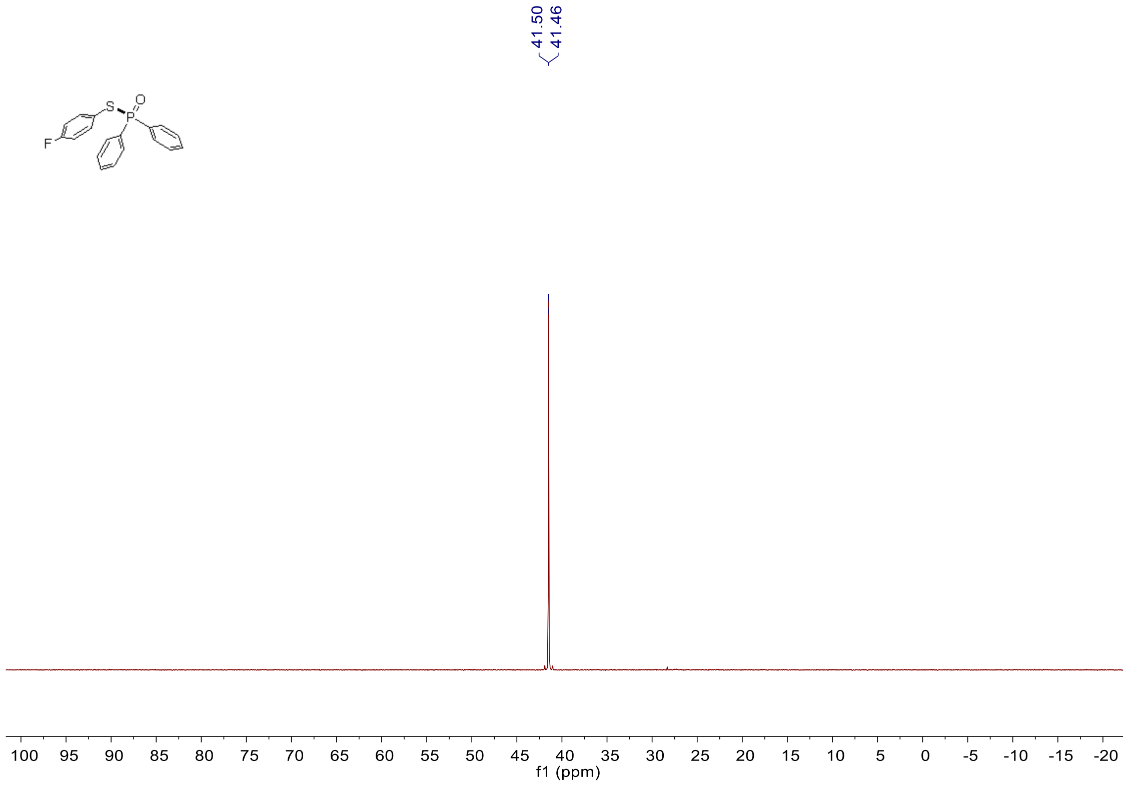

Figure S100. ^{31}P NMR spectra in $CDCl_3$ for Compound **5d**

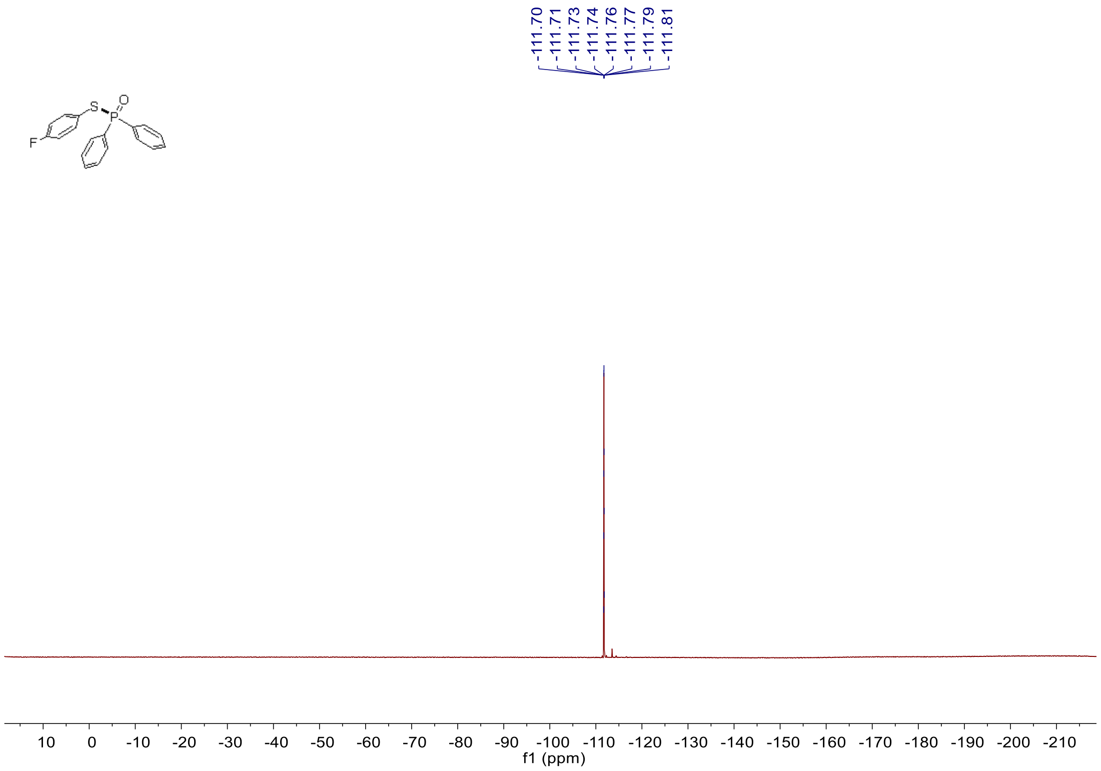

Figure S101. ^{19}F NMR spectra in $CDCl_3$ for compound **5d**

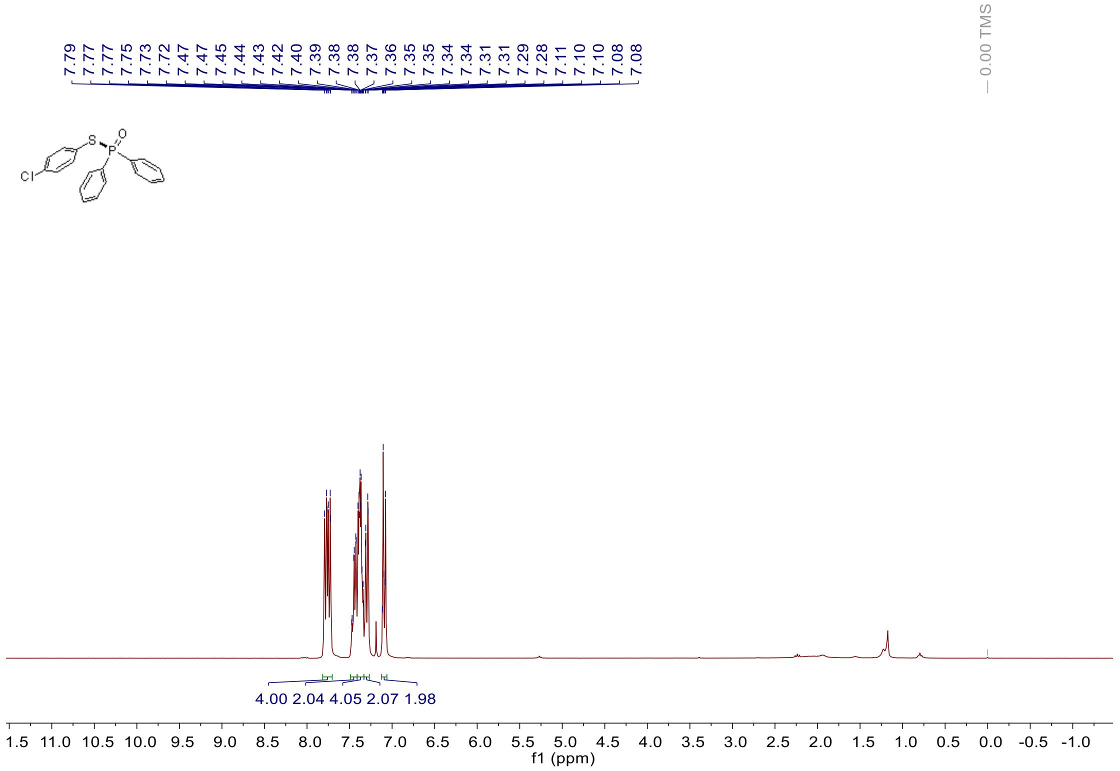

Figure S102. ^{1}H NMR spectra in $CDCl_3$ for Compound **5e**

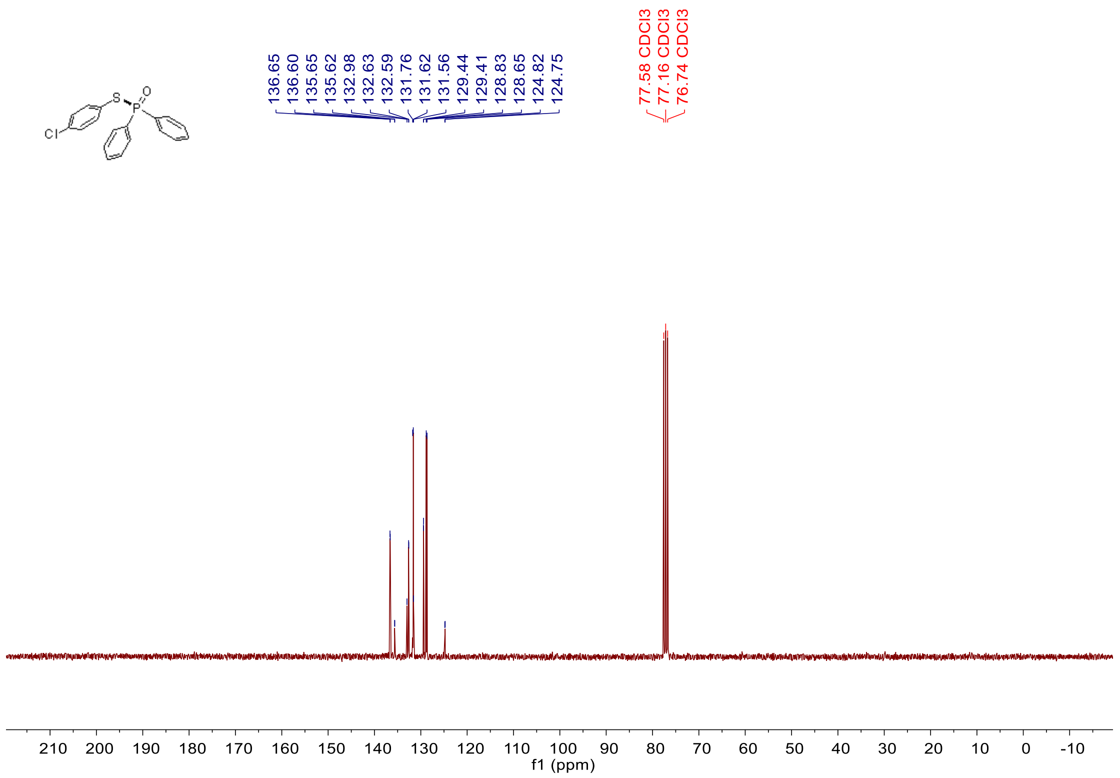

Figure S103. ^{13}C NMR spectra in $CDCl_3$ for Compound **5e**

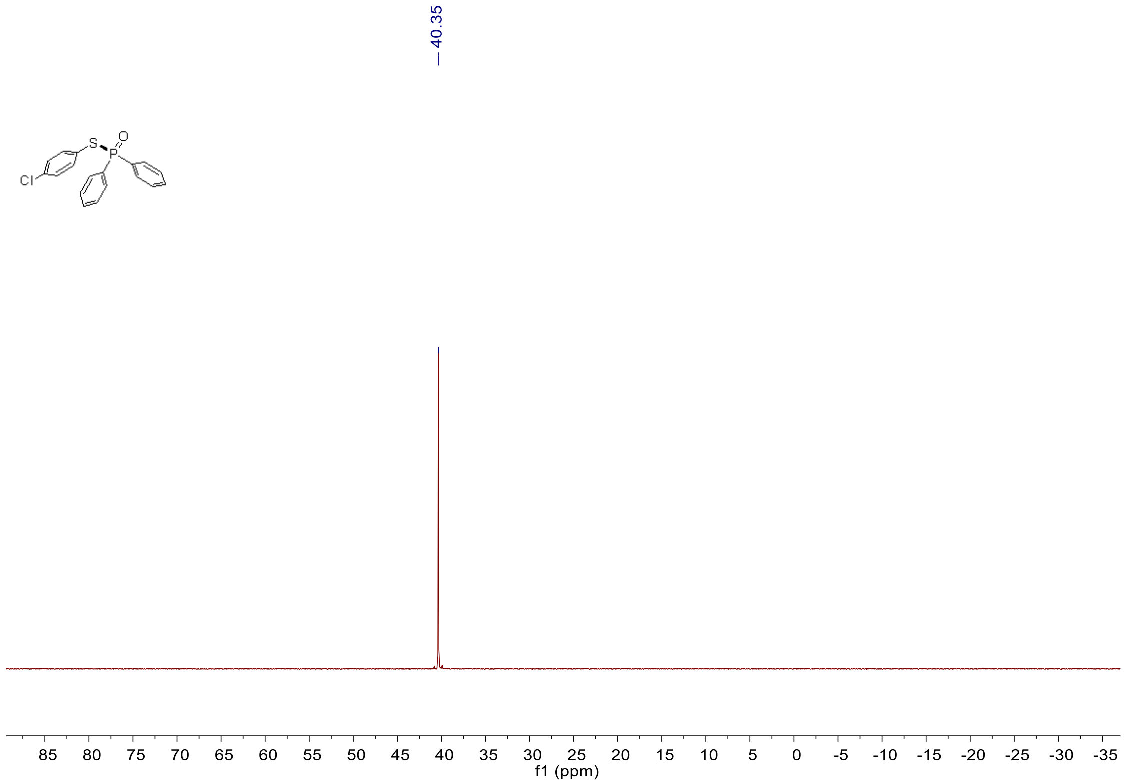

Figure S104. ^{31}P NMR spectra in $CDCl_3$ for Compound **5e**

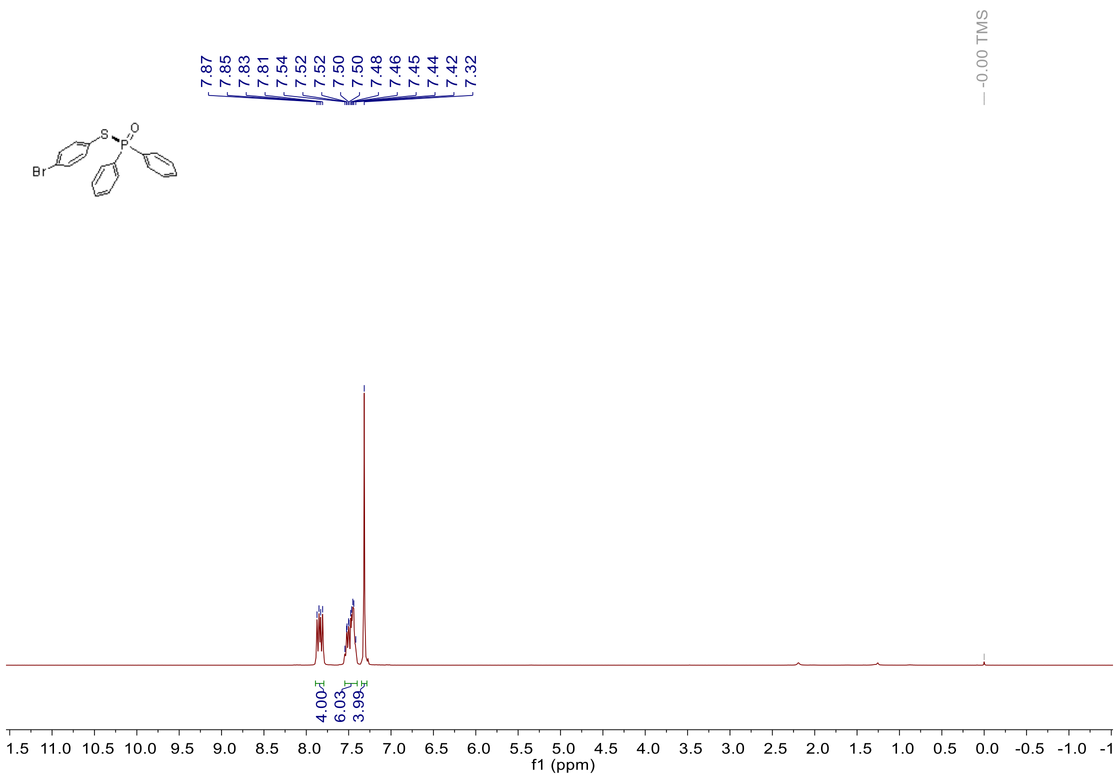

Figure S105. ^{1}H NMR spectra in $CDCl_3$ for Compound **5f**

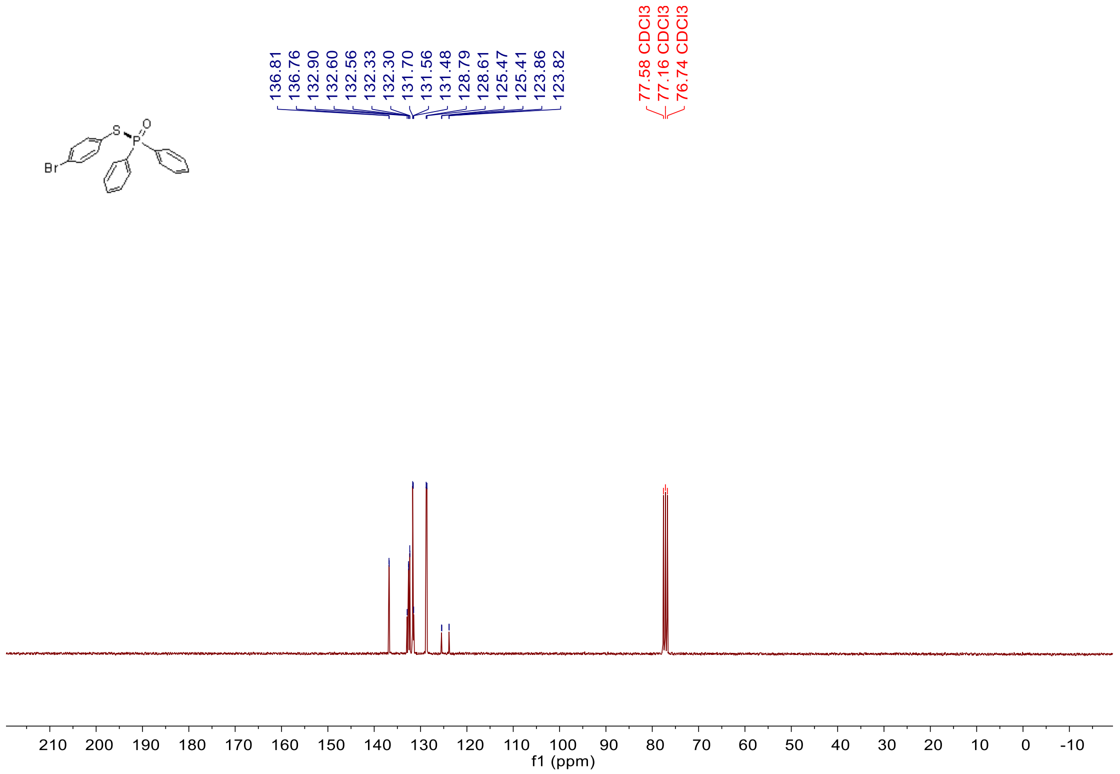

Figure S106. ^{13}C NMR spectra in $CDCl_3$ for Compound **5f**

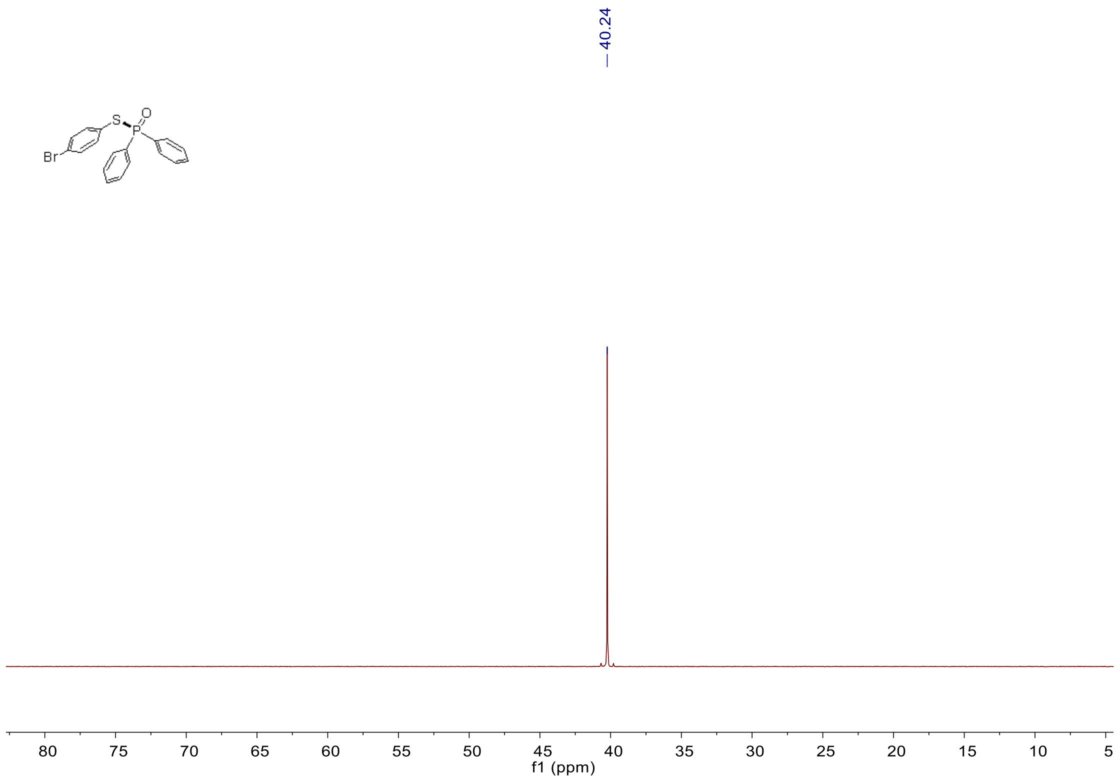

Figure S107. ^{31}P NMR spectra in $CDCl_3$ for Compound **5f**

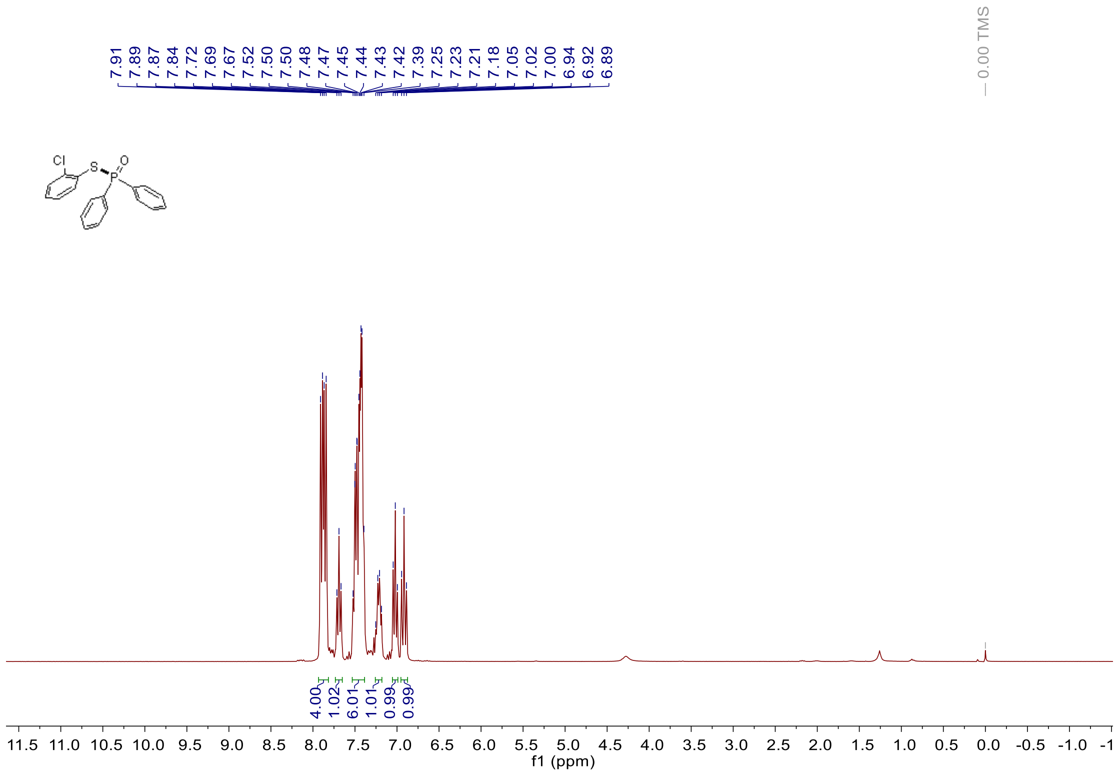

Figure S108. ^{1}H NMR spectra in $CDCl_3$ for Compound **5g**

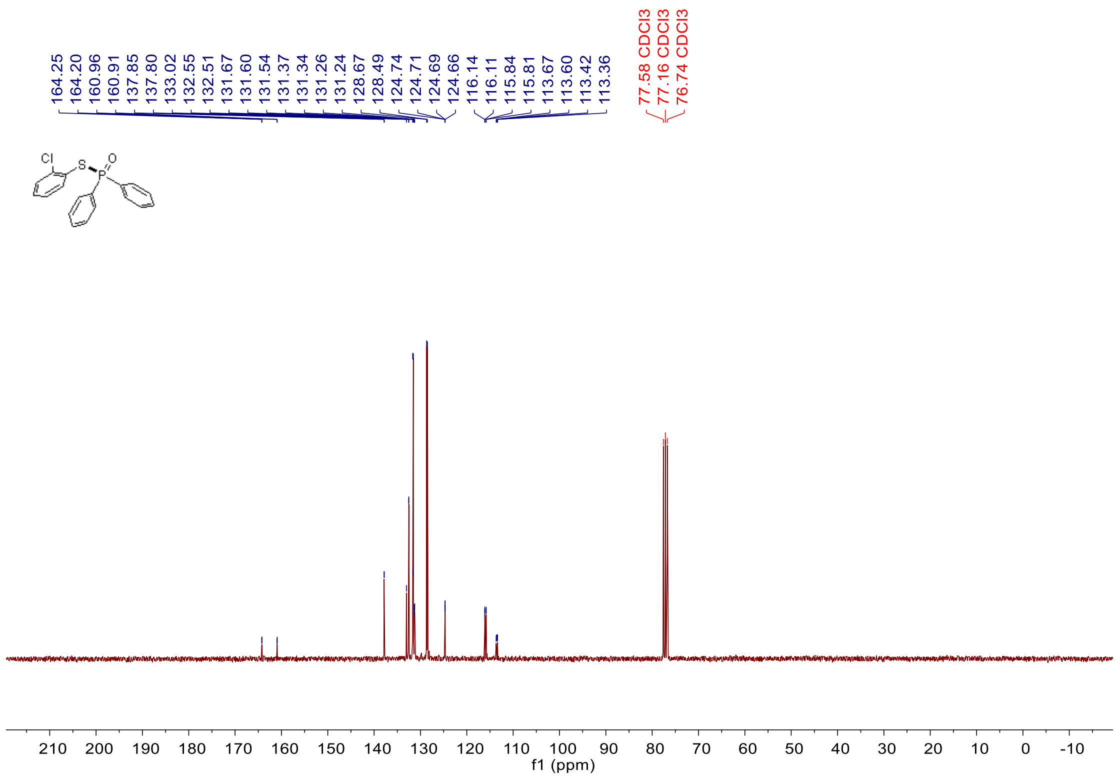

Figure S109. ^{13}C NMR spectra in $CDCl_3$ for Compound **5g**

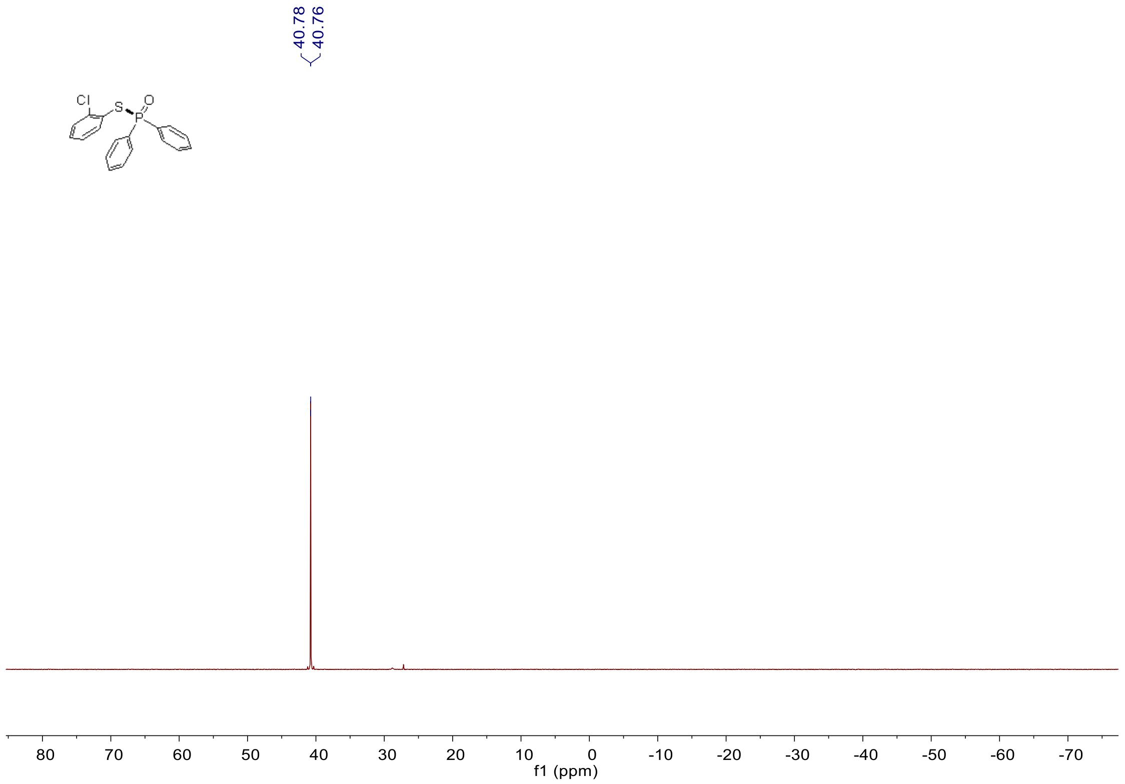

Figure S110. ^{31}P NMR spectra in $CDCl_3$ for Compound **5g**

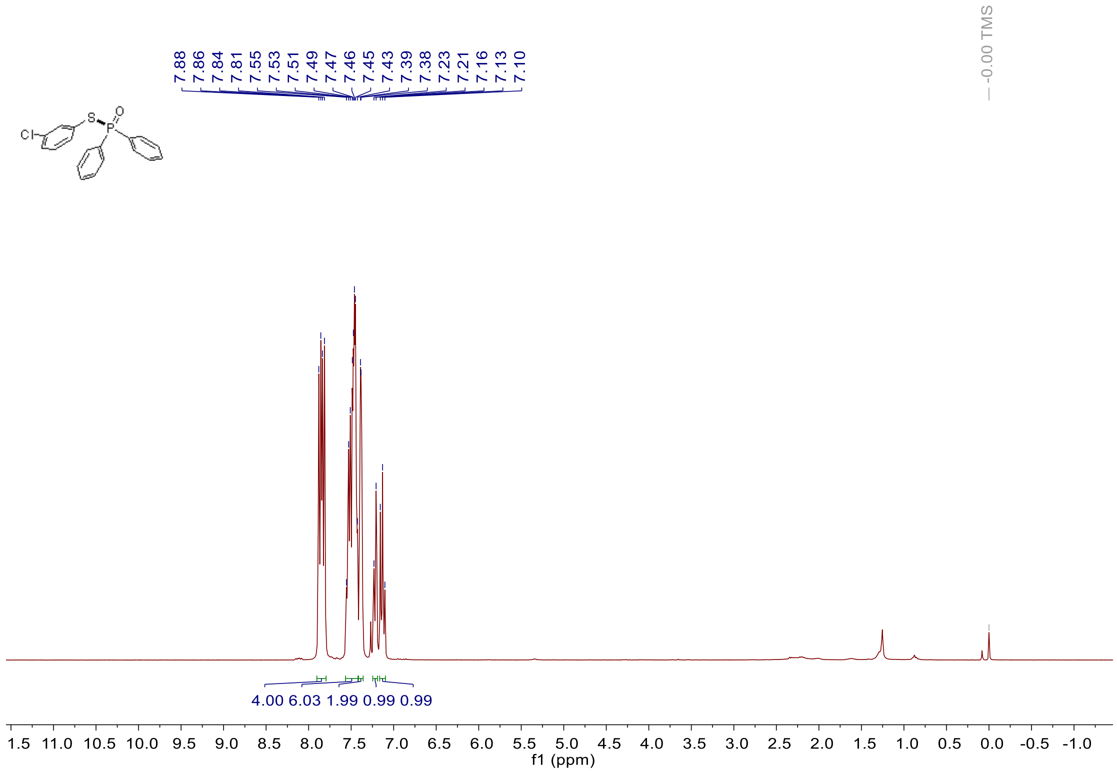

Figure S111. ^{1}H NMR spectra in $CDCl_3$ for Compound **5h**

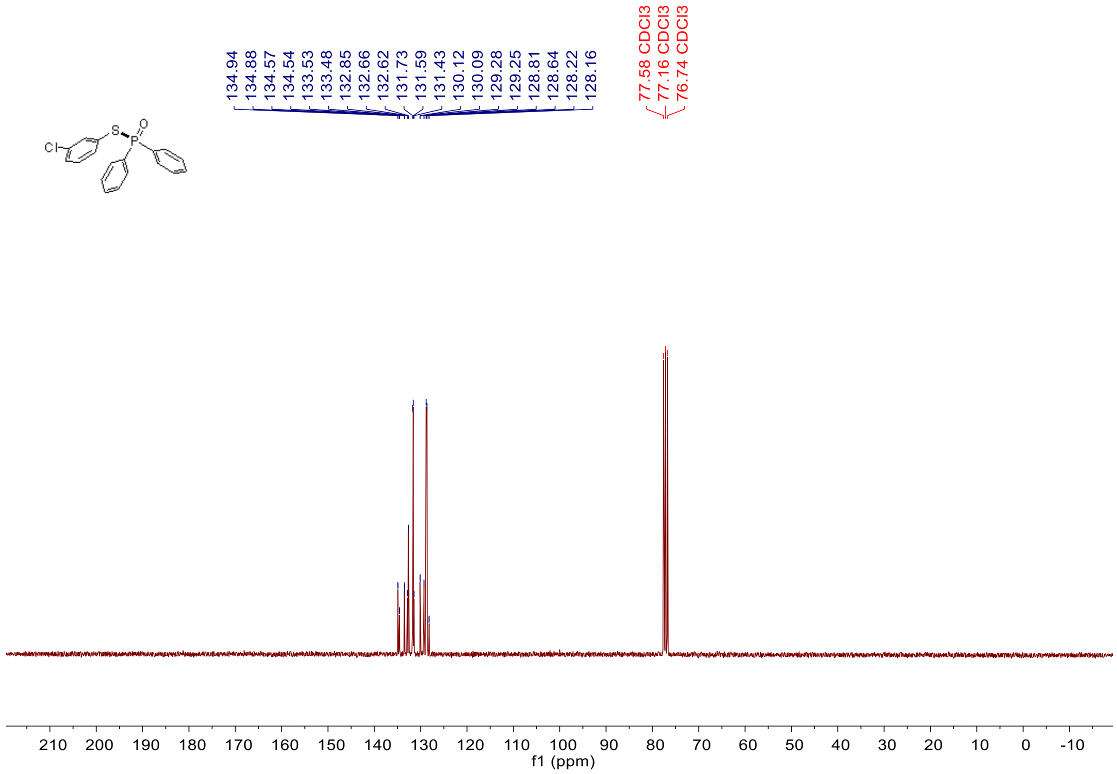

Figure S112. ^{13}C NMR spectra in $CDCl_3$ for Compound **5h**

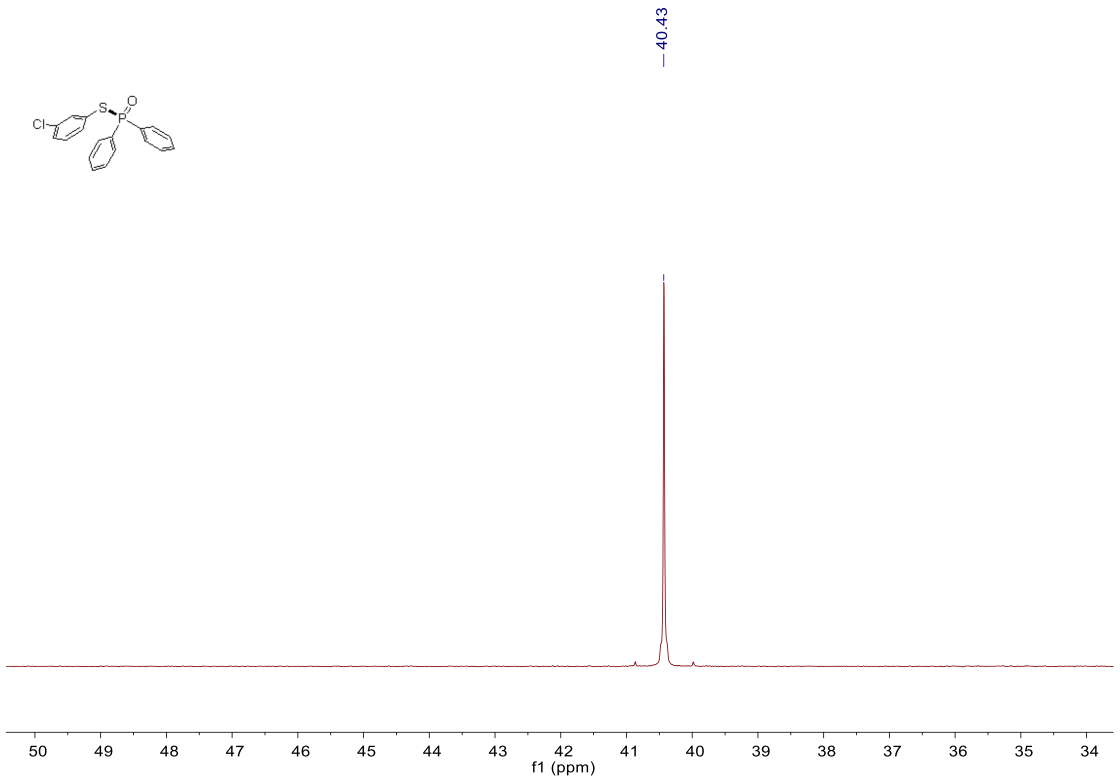

Figure S113. ^{31}P NMR spectra in $CDCl_3$ for Compound **5h**

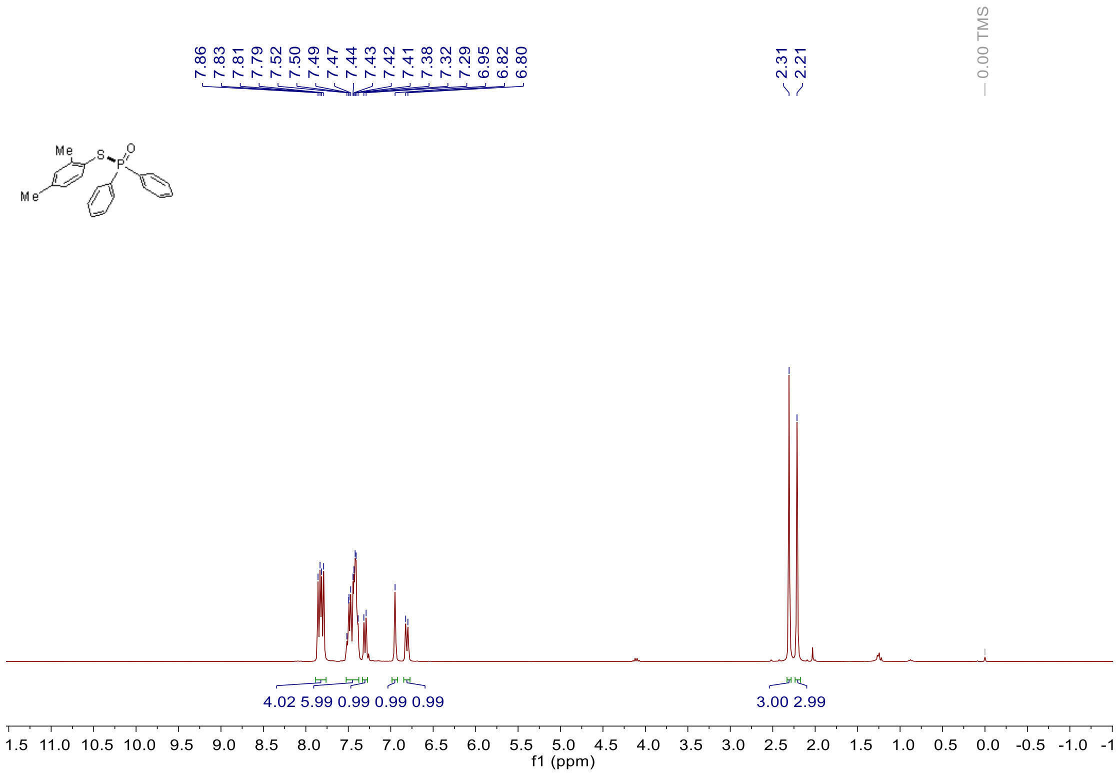

Figure S114. ^{1}H NMR spectra in $CDCl_3$ for Compound **5i**

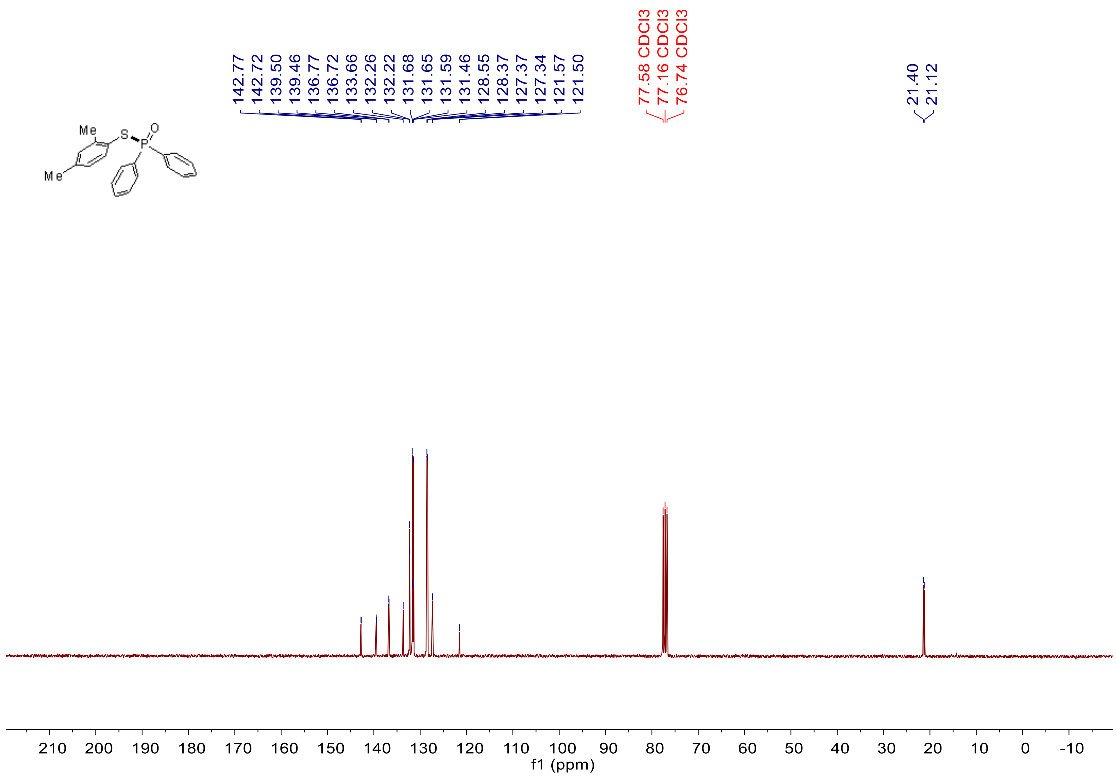

Figure S115. ^{13}C NMR spectra in $CDCl_3$ for Compound **5i**

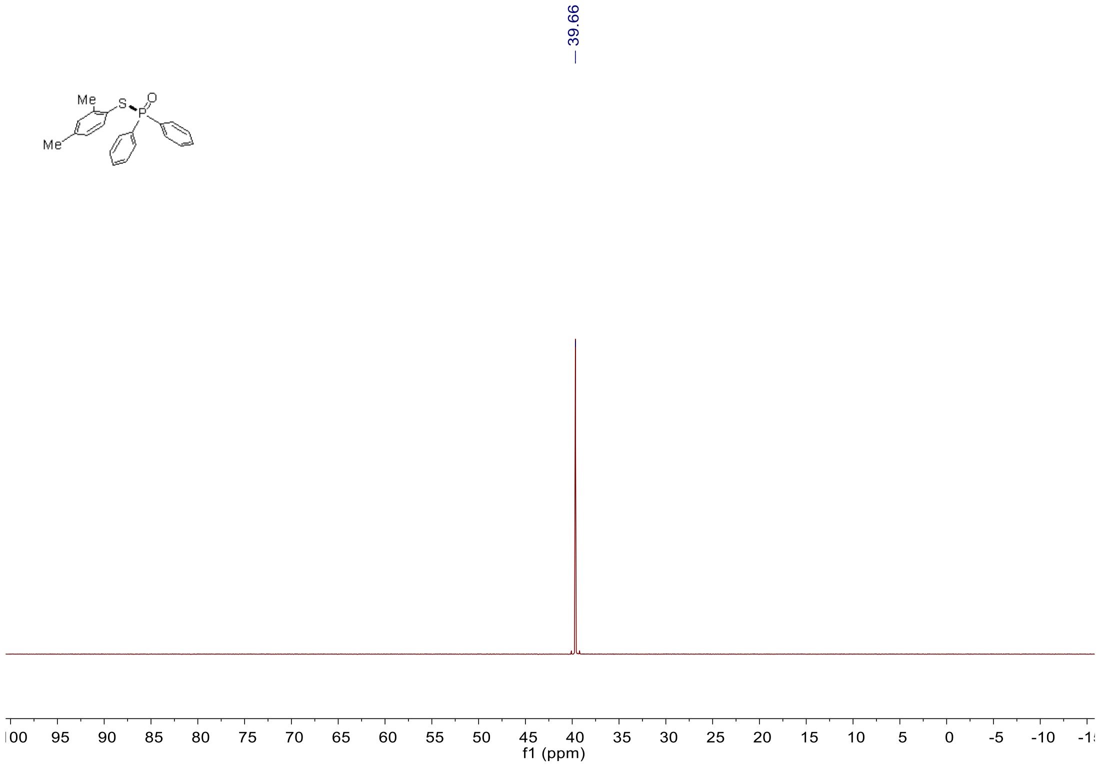

Figure S116. ^{31}P NMR spectra in $CDCl_3$ for Compound **5i**

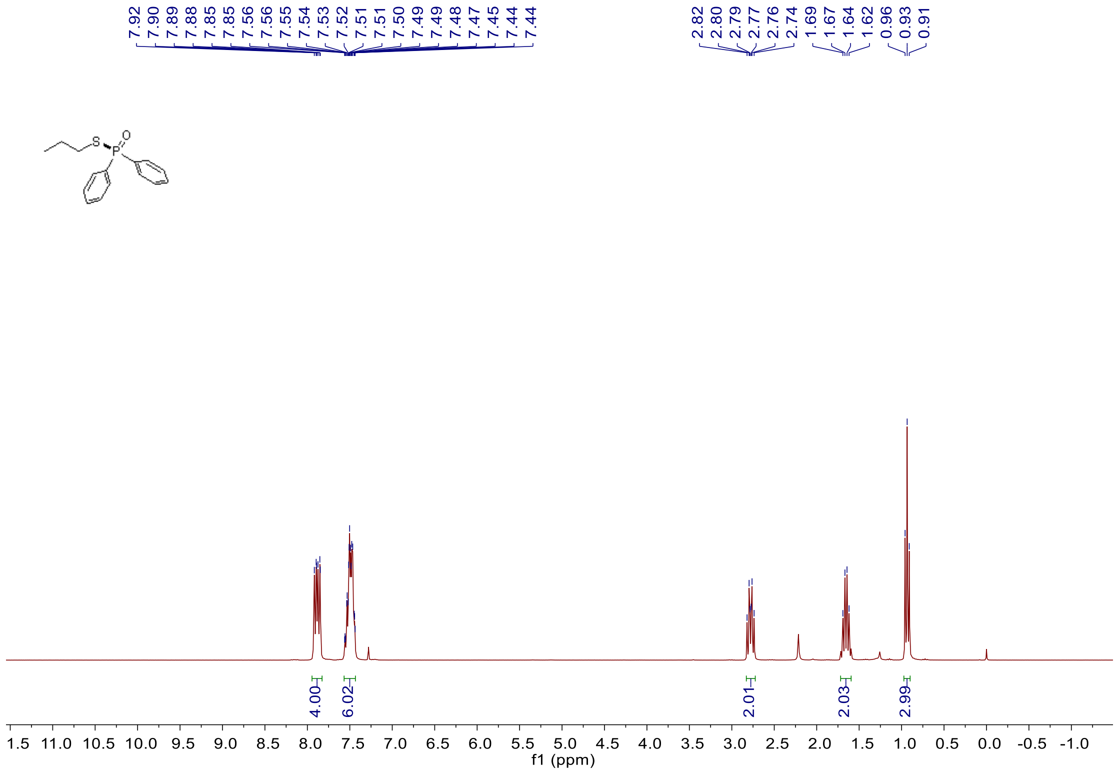

Figure S117. ^{1}H NMR spectra in $CDCl_3$ for Compound **5j**

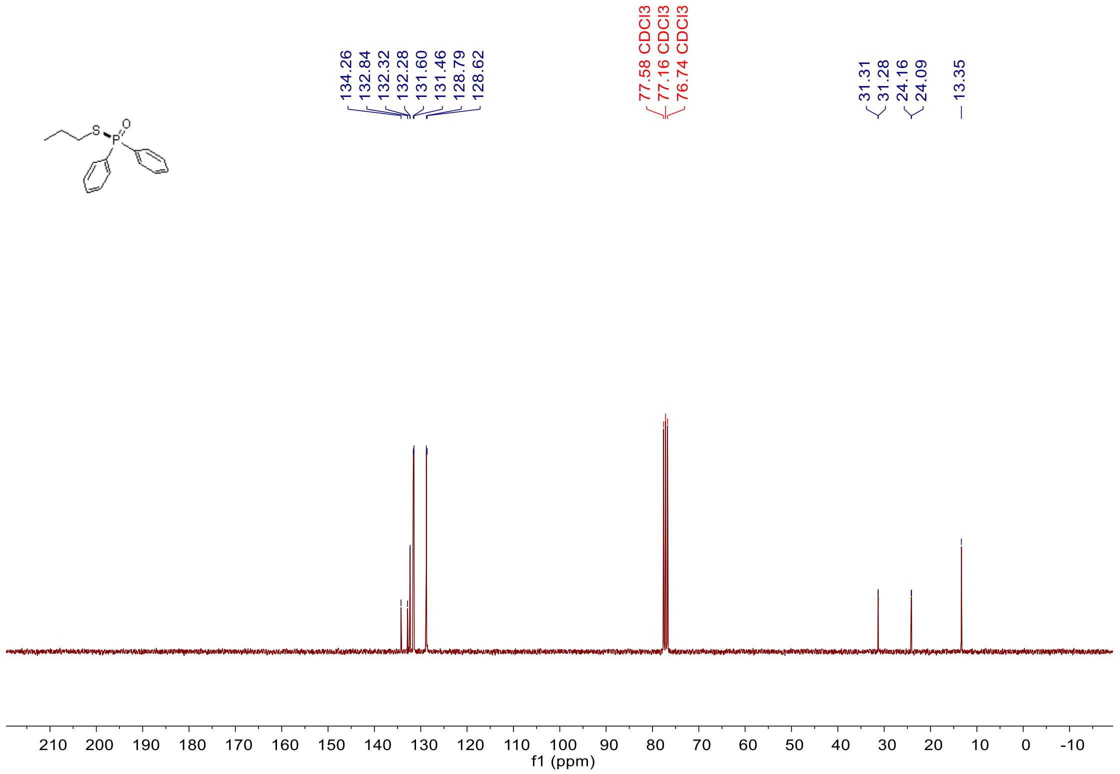

Figure S118. ^{13}C NMR spectra in $CDCl_3$ for Compound **5j**

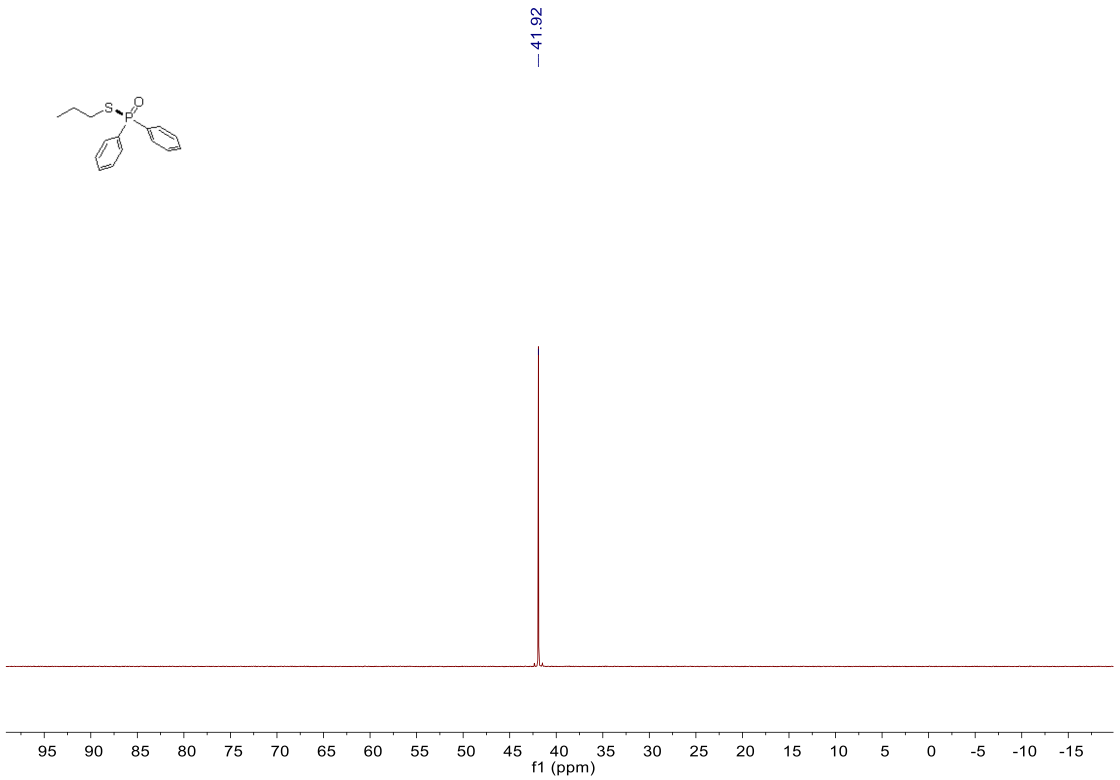

Figure S119. ^{31}P NMR spectra in $CDCl_3$ for Compound **5j**

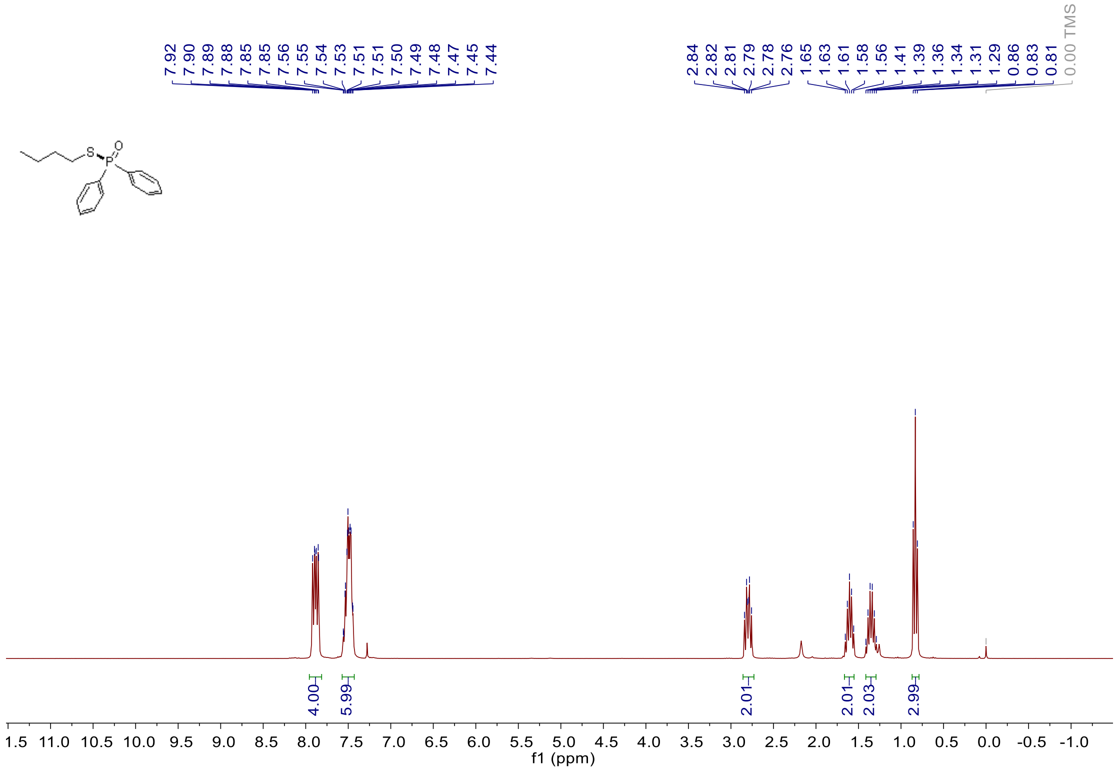

Figure S120. ^{1}H NMR spectra in $CDCl_3$ for Compound **5k**

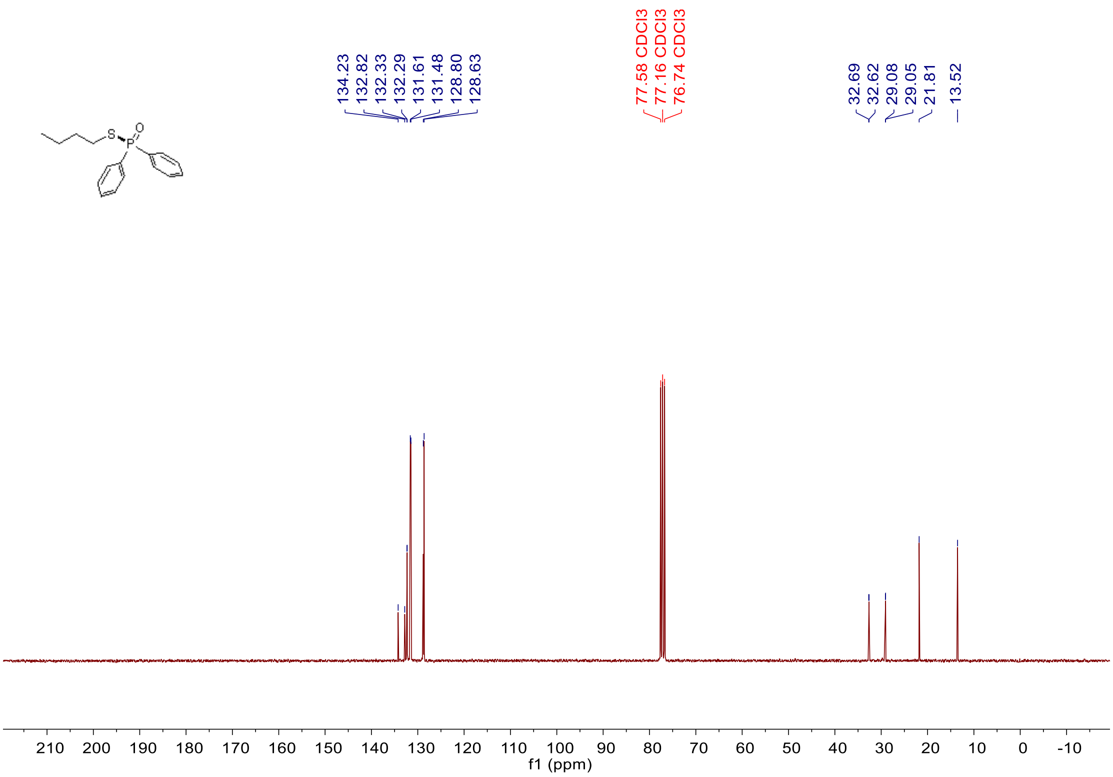

Figure S121. ^{13}C NMR spectra in $CDCl_3$ for Compound **5k**

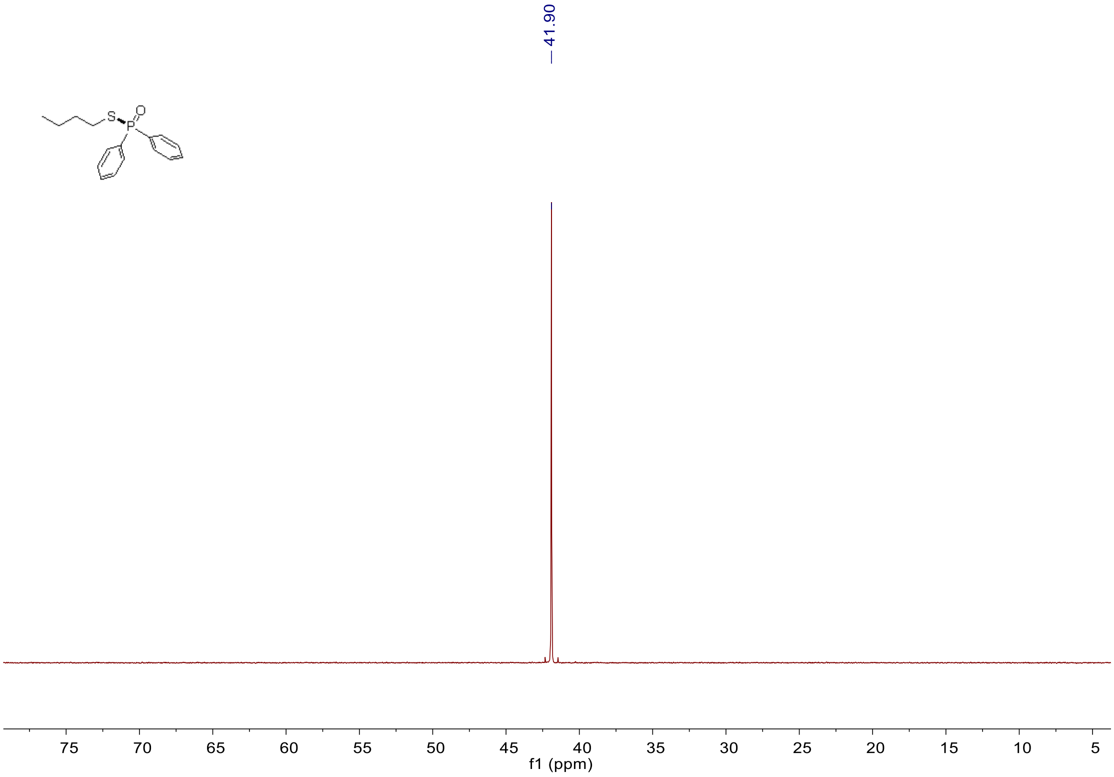

Figure S122. ^{31}P NMR spectra in $CDCl_3$ for Compound **5k**

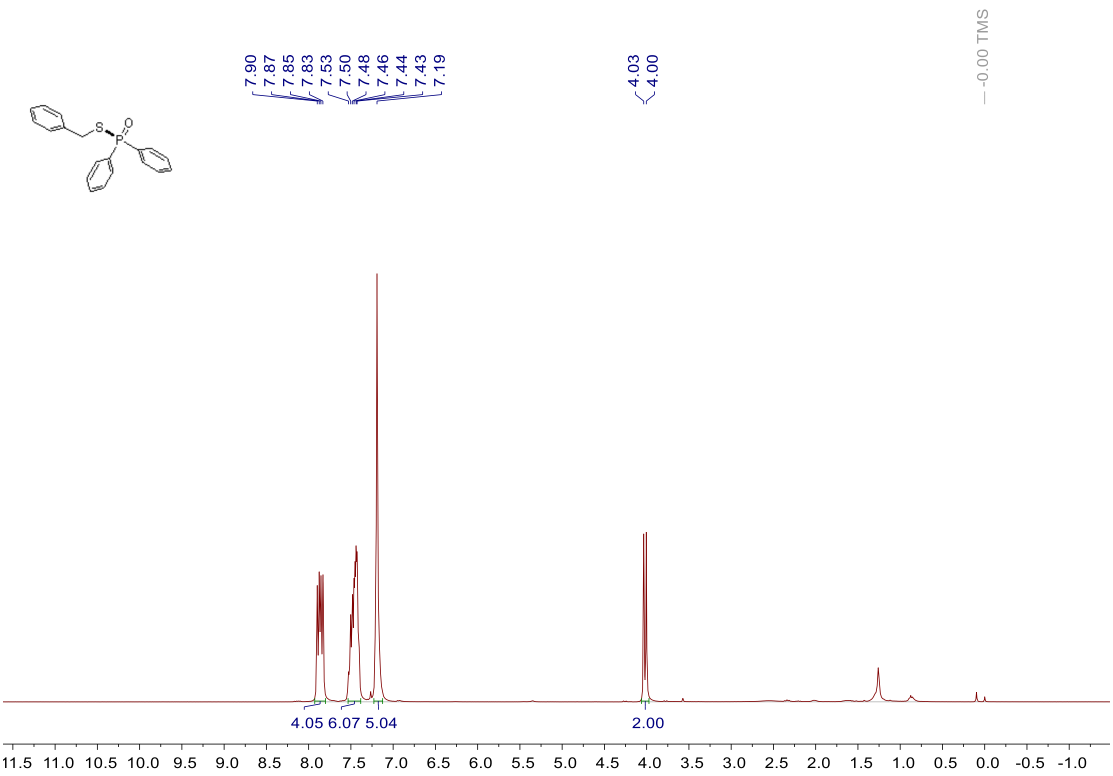

Figure S123. ^{1}H NMR spectra in $CDCl_3$ for Compound **5l**

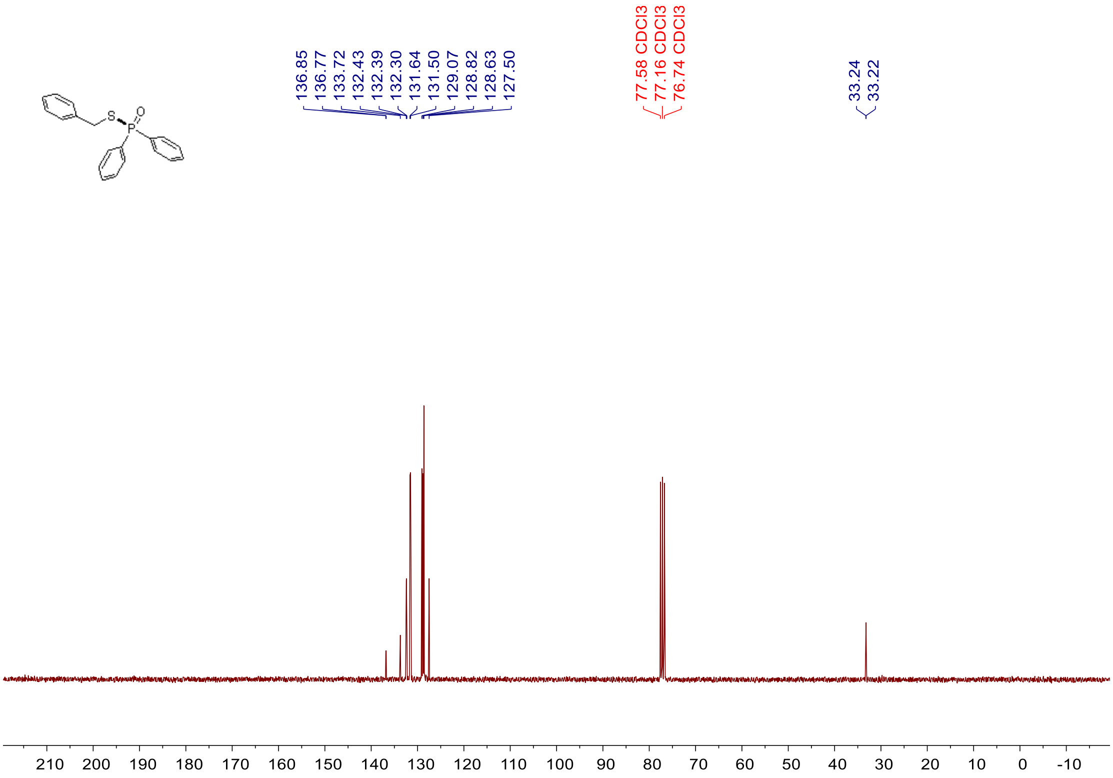

Figure S124. ^{13}C NMR spectra in $CDCl_3$ for Compound **5l**

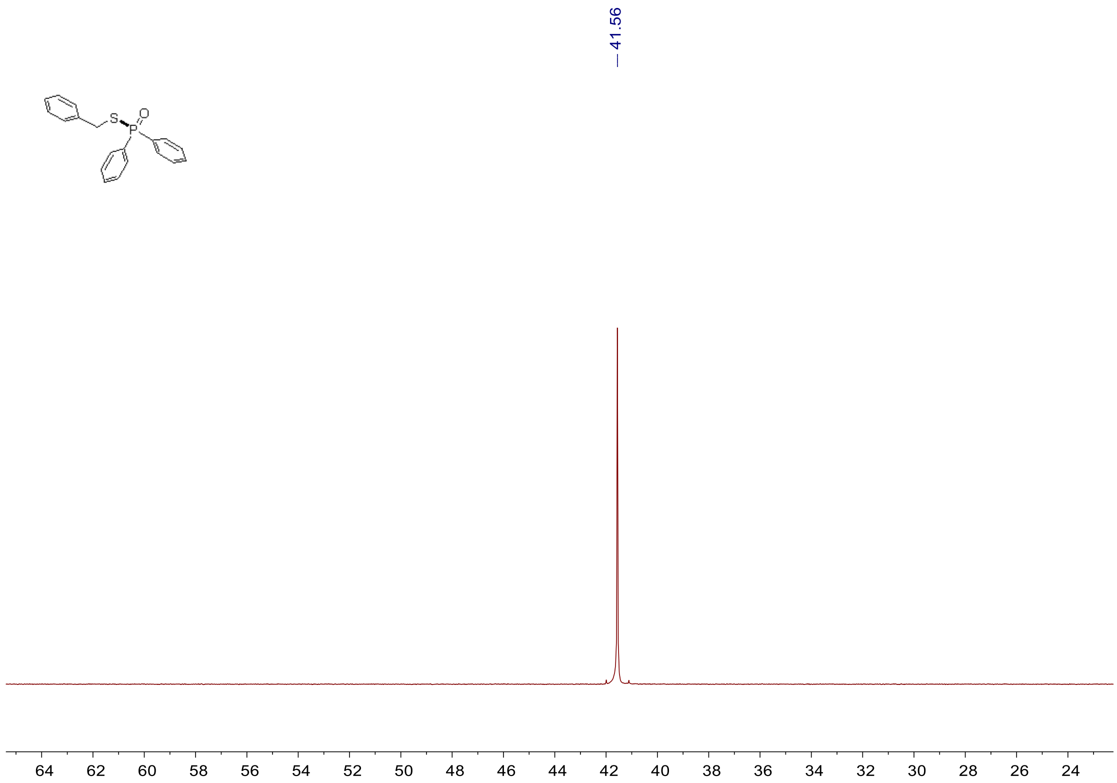

Figure S125. ^{31}P NMR spectra in $CDCl_3$ for Compound **5l**

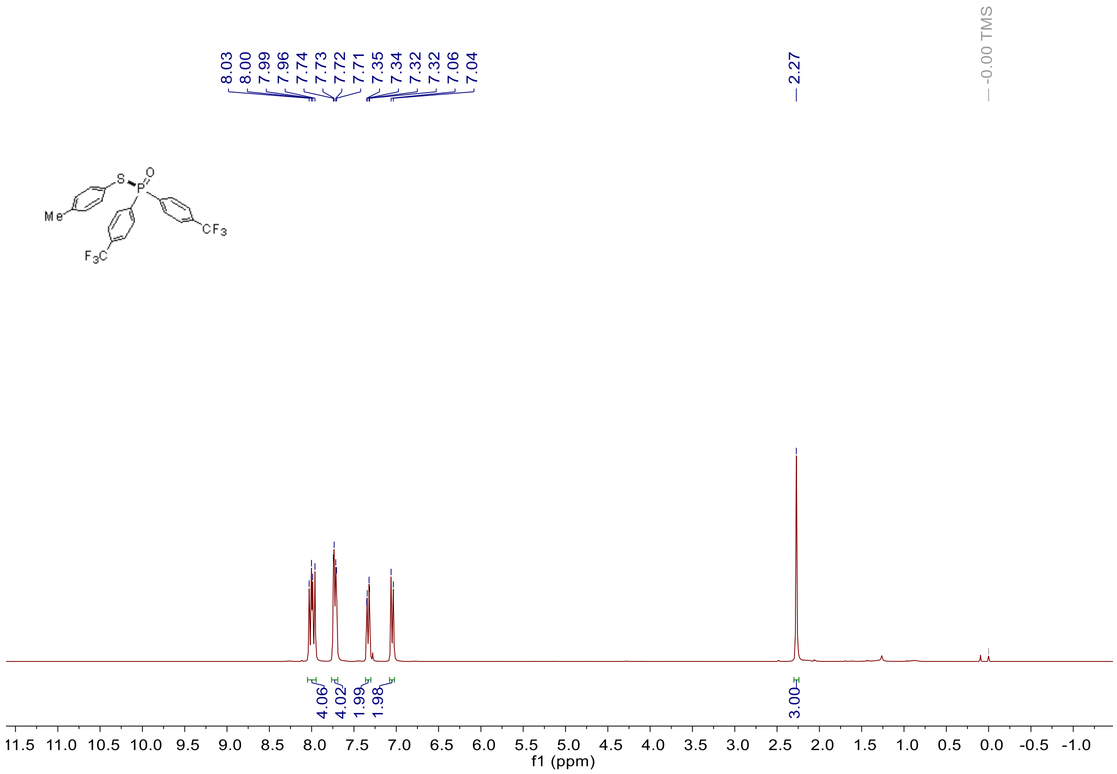

Figure S126. ^{1}H NMR spectra in $CDCl_3$ for Compound **5m**

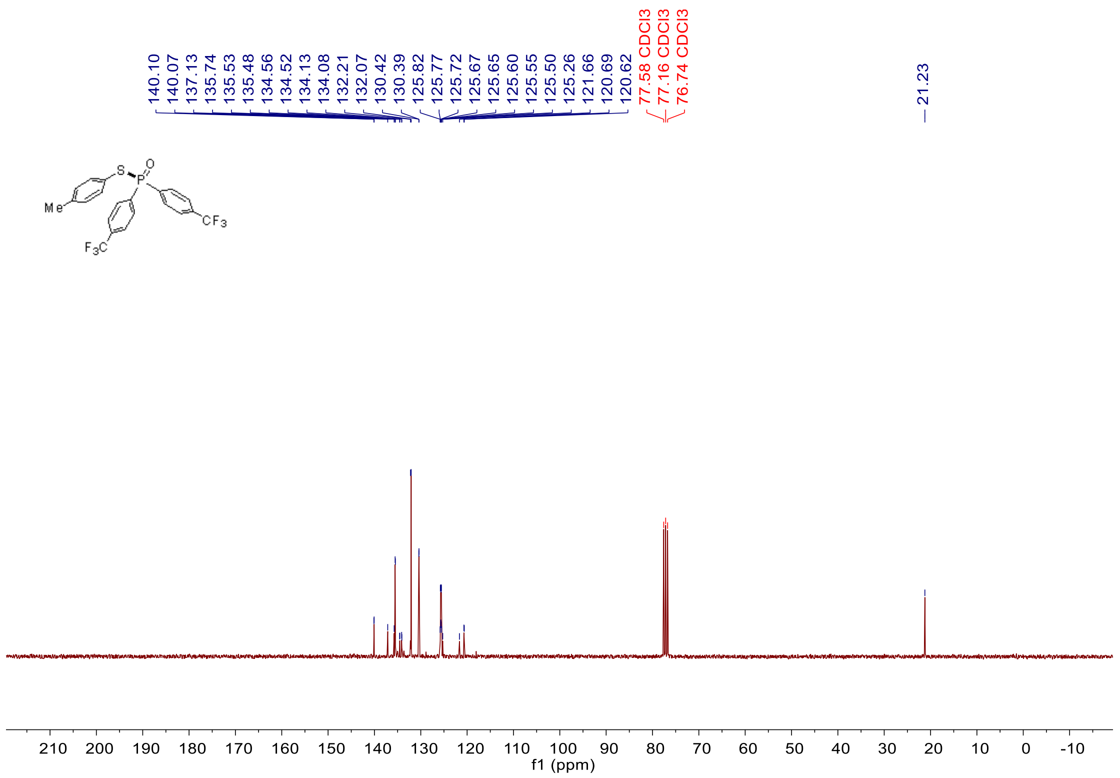

Figure S127. ^{13}C NMR spectra in $CDCl_3$ for Compound **5m**

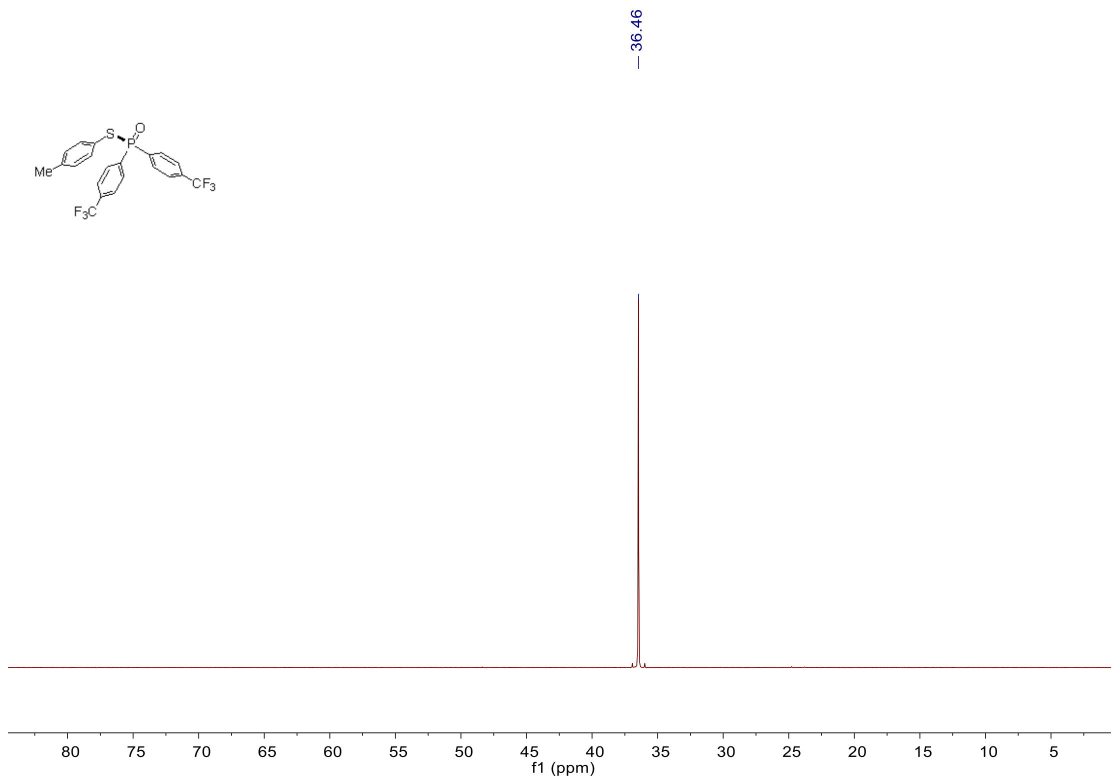

Figure S128. ^{31}P NMR spectra in $CDCl_3$ for Compound **5m**

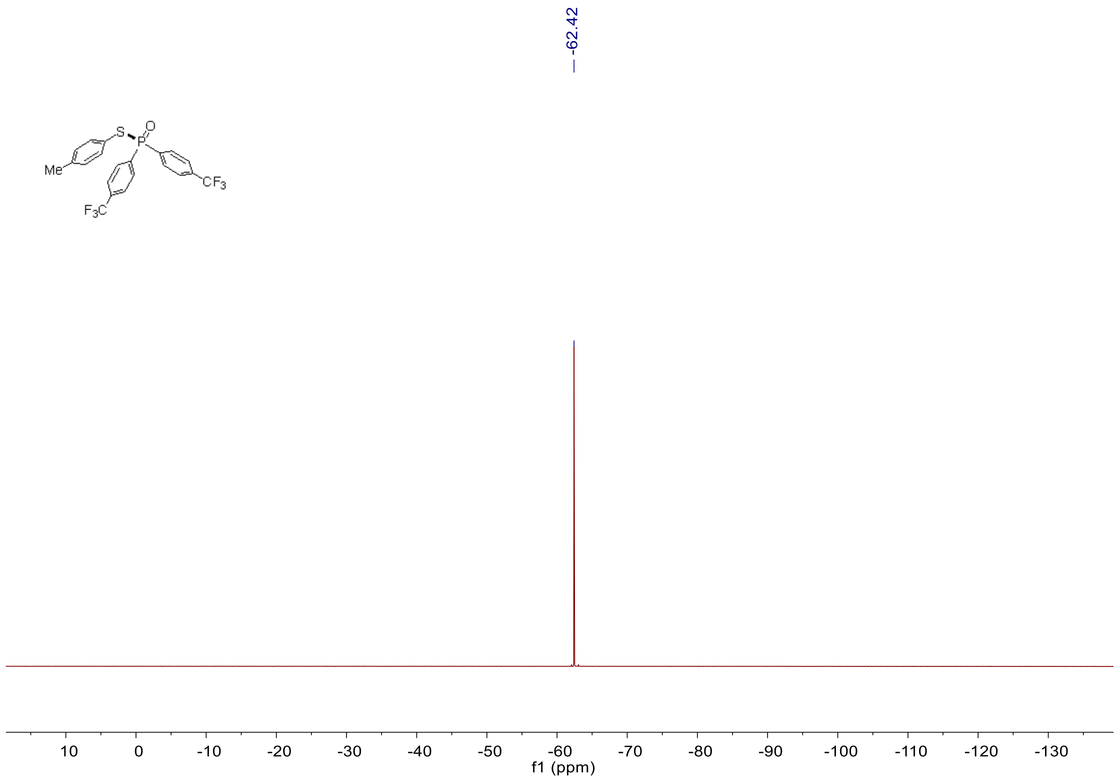

Figure S129. ^{19}F NMR spectra in $CDCl_3$ for Compound **5m**

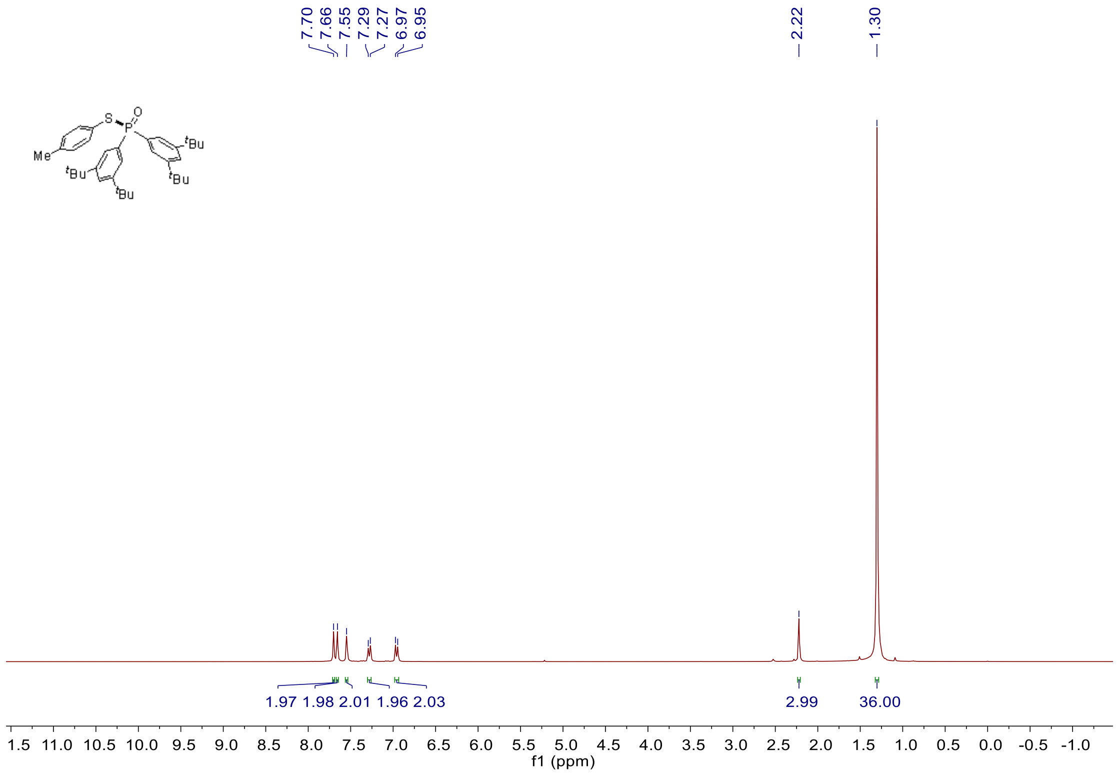

Figure S130. ^{1}H NMR spectra in $CDCl_3$ for Compound **5n**

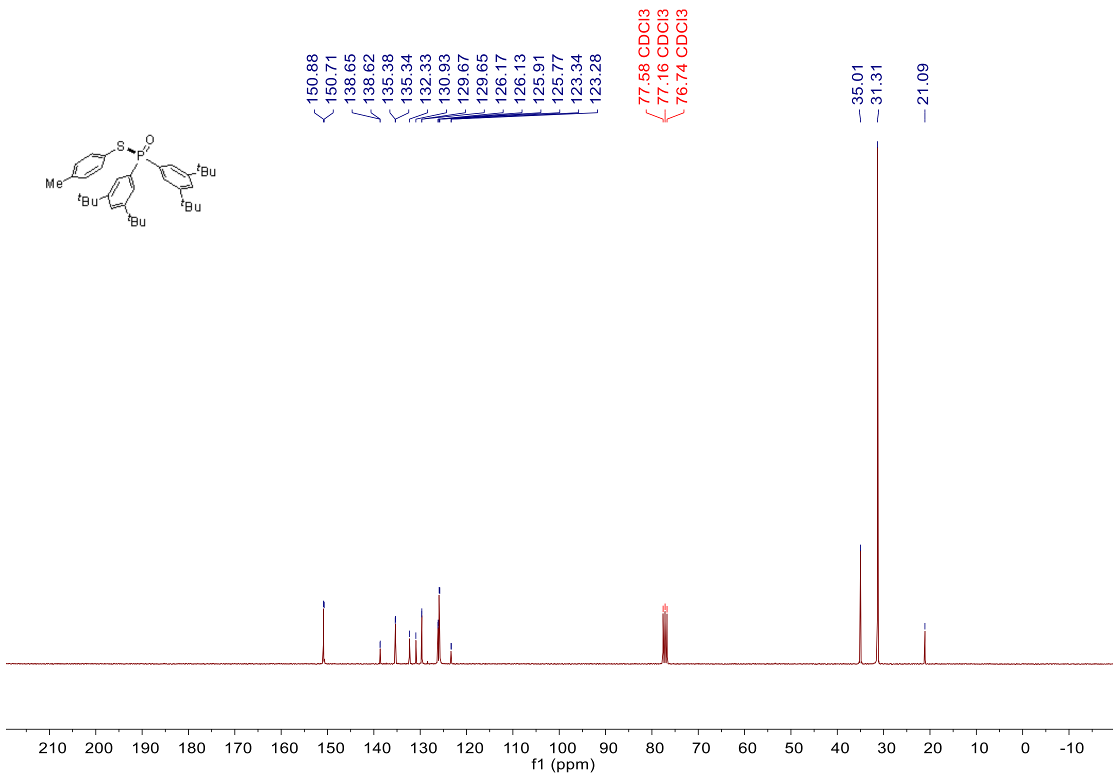

Figure S131. ^{13}C NMR spectra in $CDCl_3$ for Compound **5n**

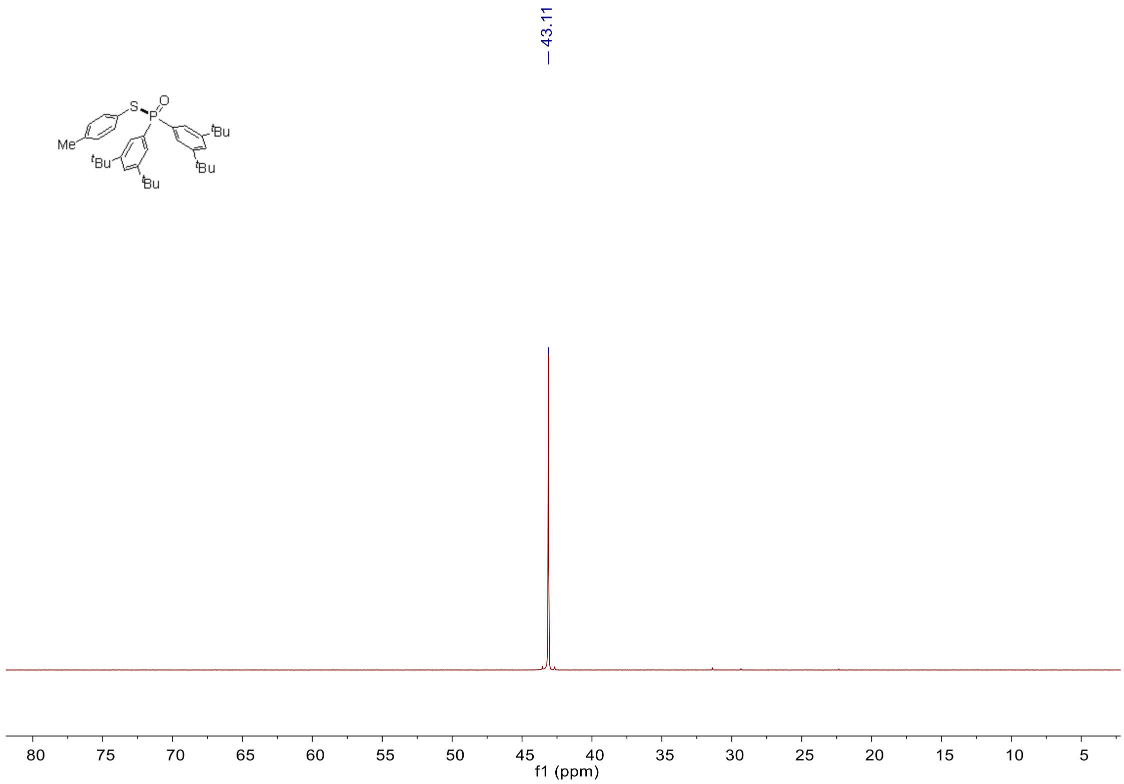

Figure S132. ^{31}P NMR spectra in $CDCl_3$ for Compound **5n**

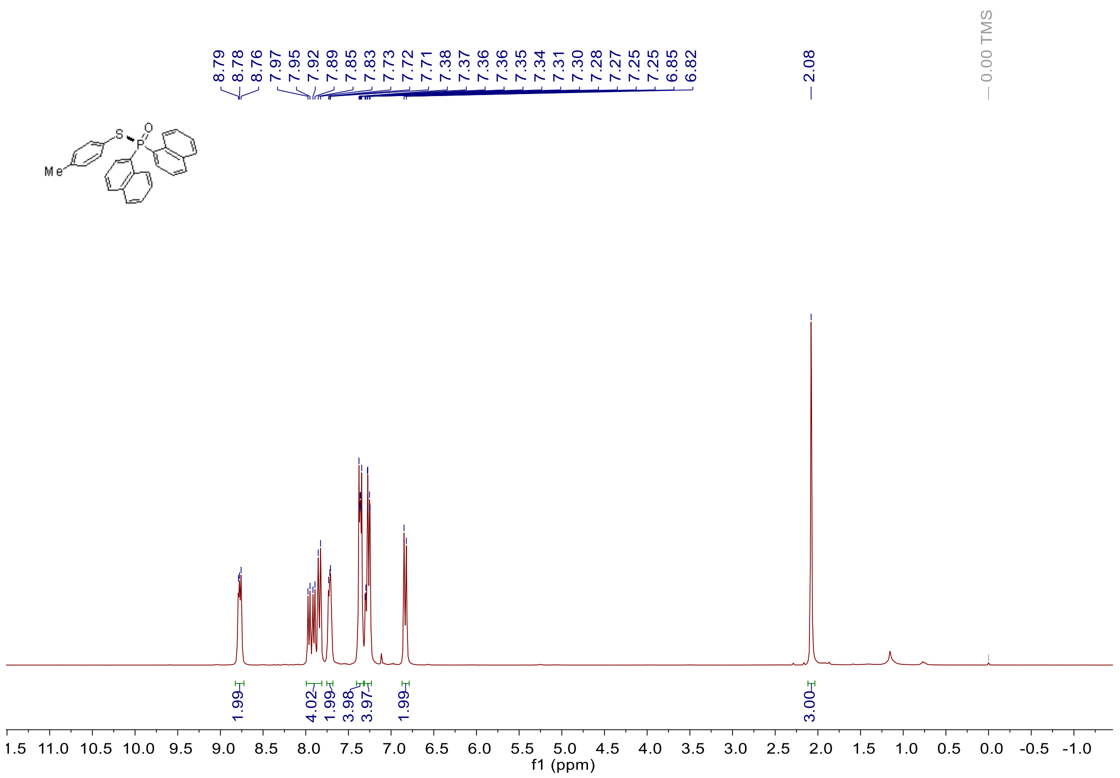

Figure S133. ^{1}H NMR spectra in $CDCl_3$ for Compound **5o**

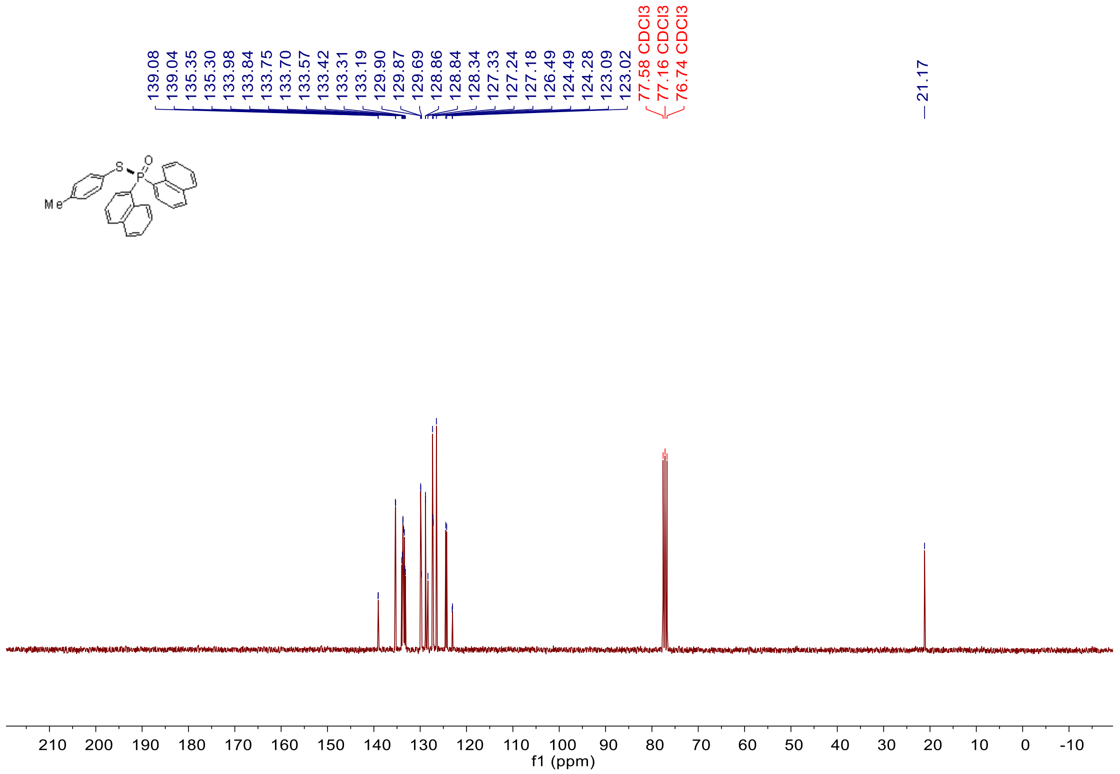

Figure S134. ^{13}C NMR spectra in $CDCl_3$ for Compound **5o**

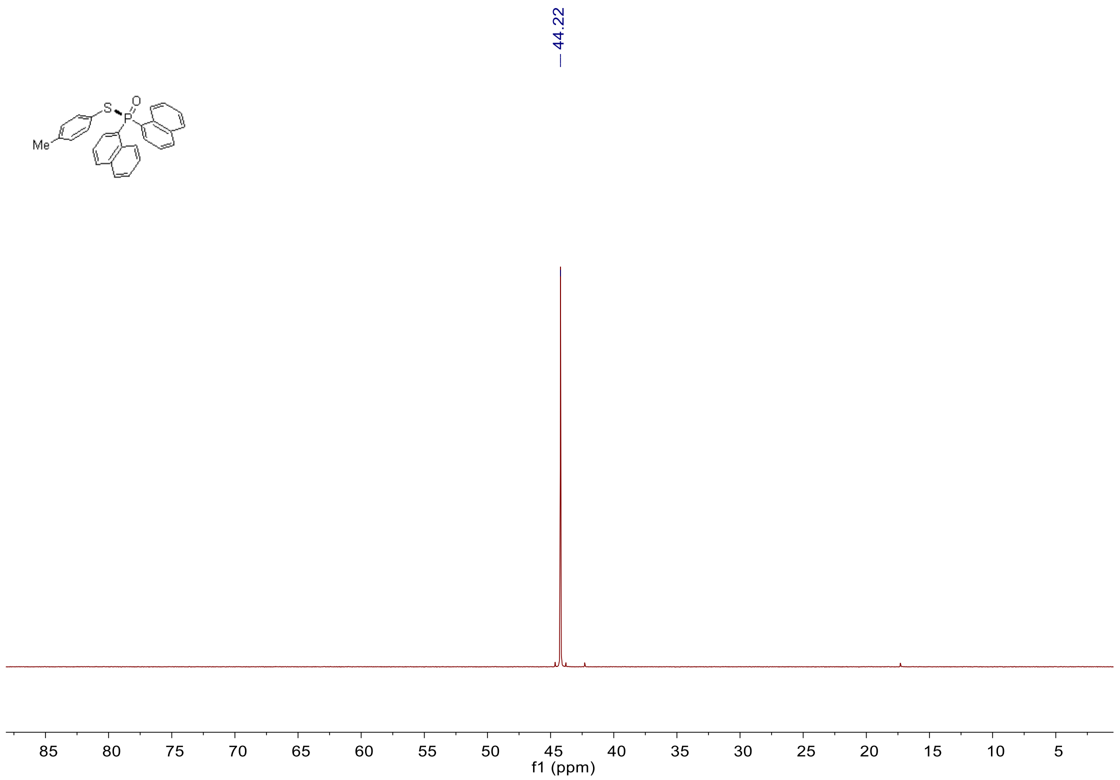

Figure S135. ^{31}P NMR spectra in $CDCl_3$ for Compound **5o**

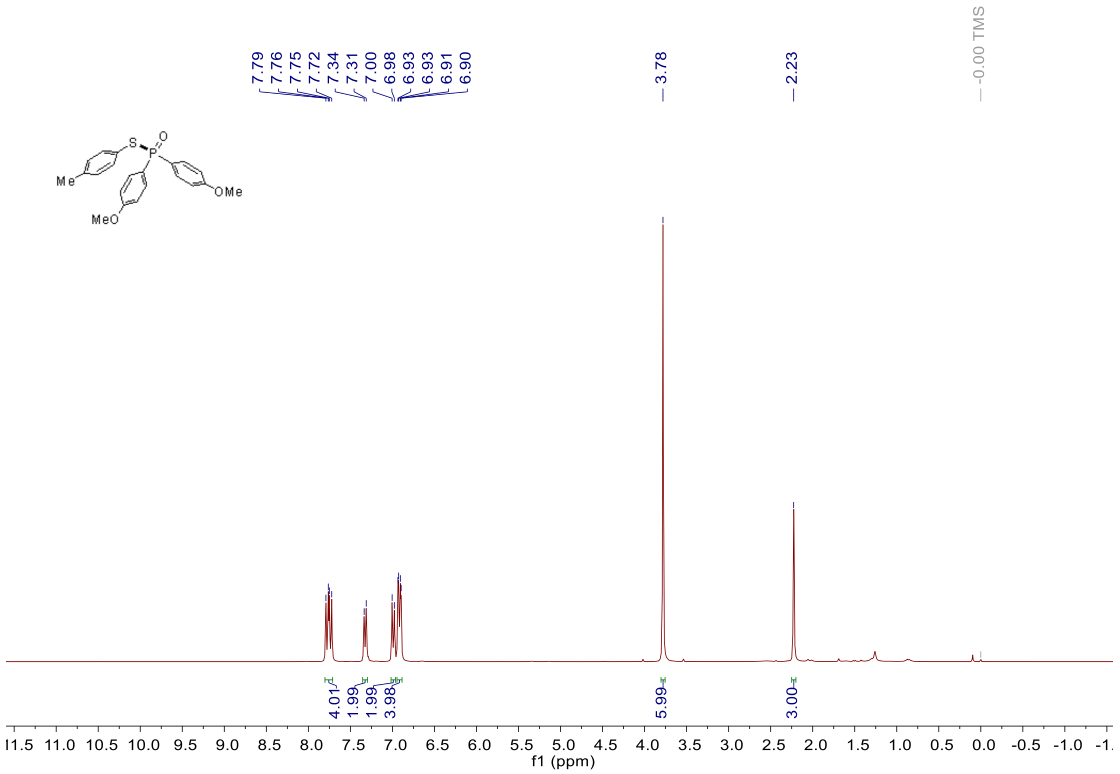

Figure S136. ^{1}H NMR spectra in $CDCl_3$ for Compound **5p**

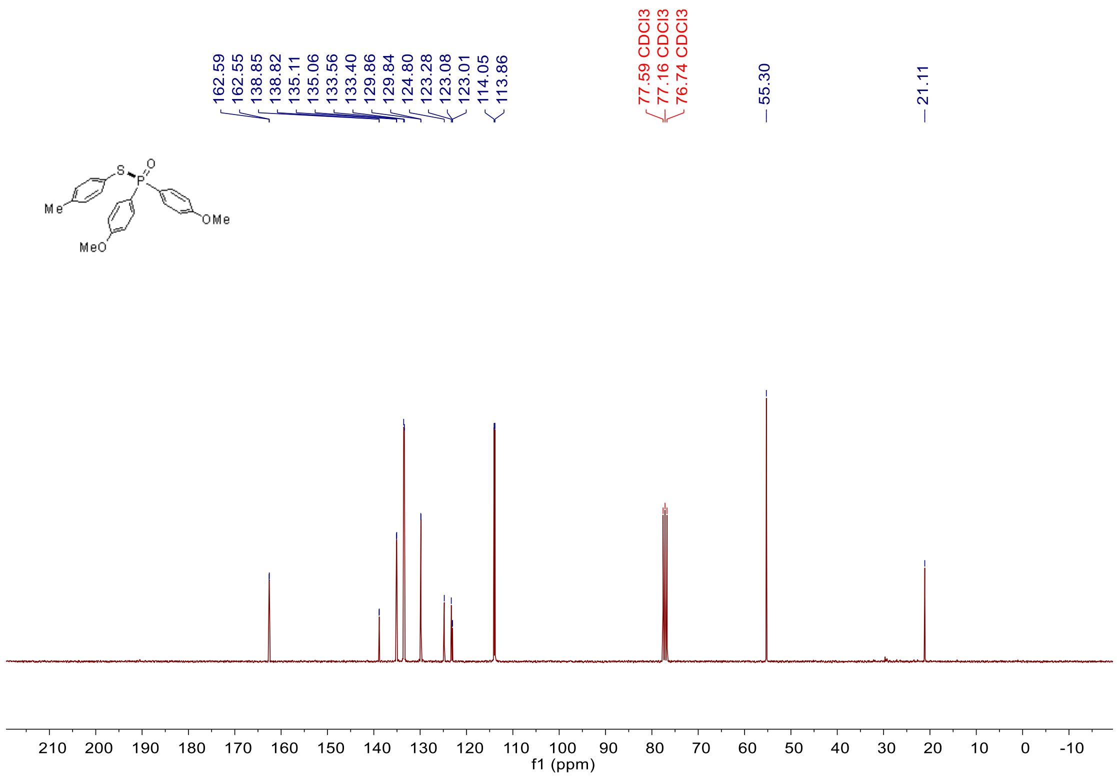

Figure S137. ^{13}C NMR spectra in $CDCl_3$ for Compound **5p**

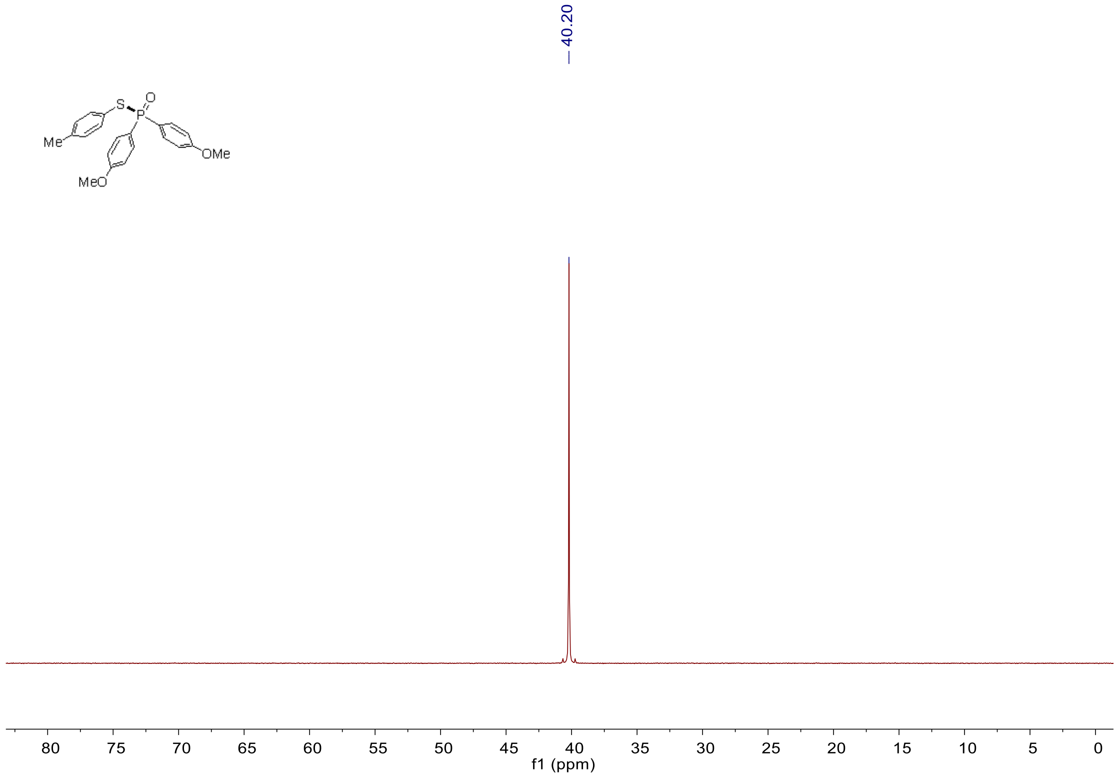

Figure S138. ^{31}P NMR spectra in $CDCl_3$ for Compound **5p**

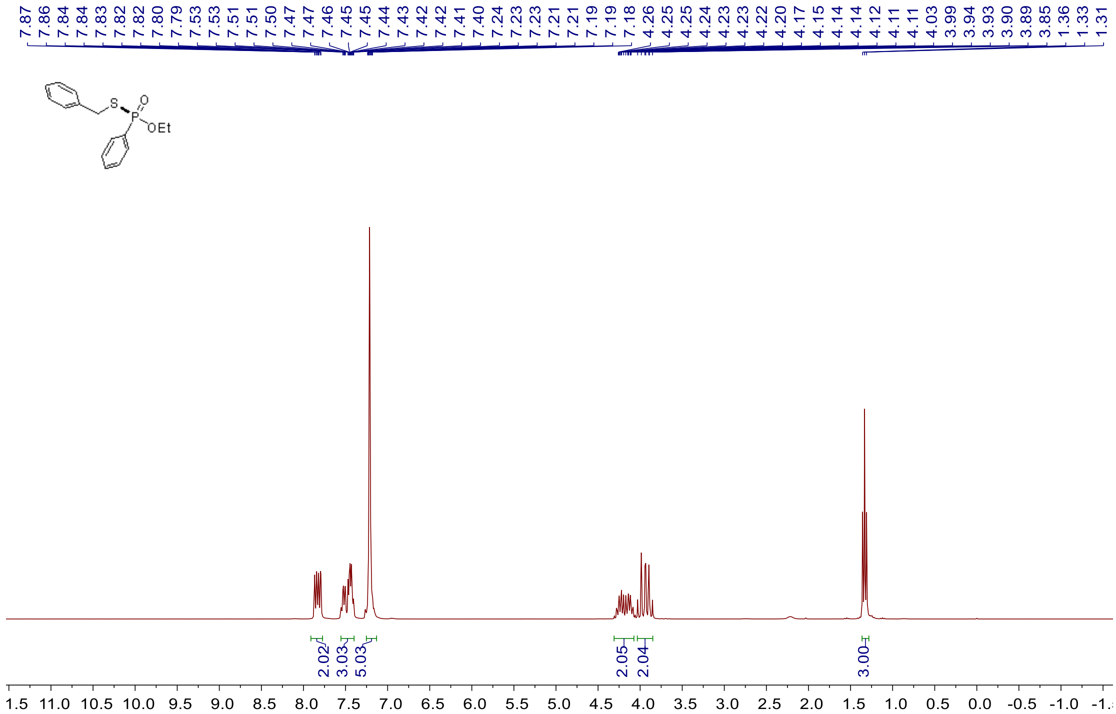

Figure S139. ^{1}H NMR spectra in $CDCl_3$ for Compound **5q**

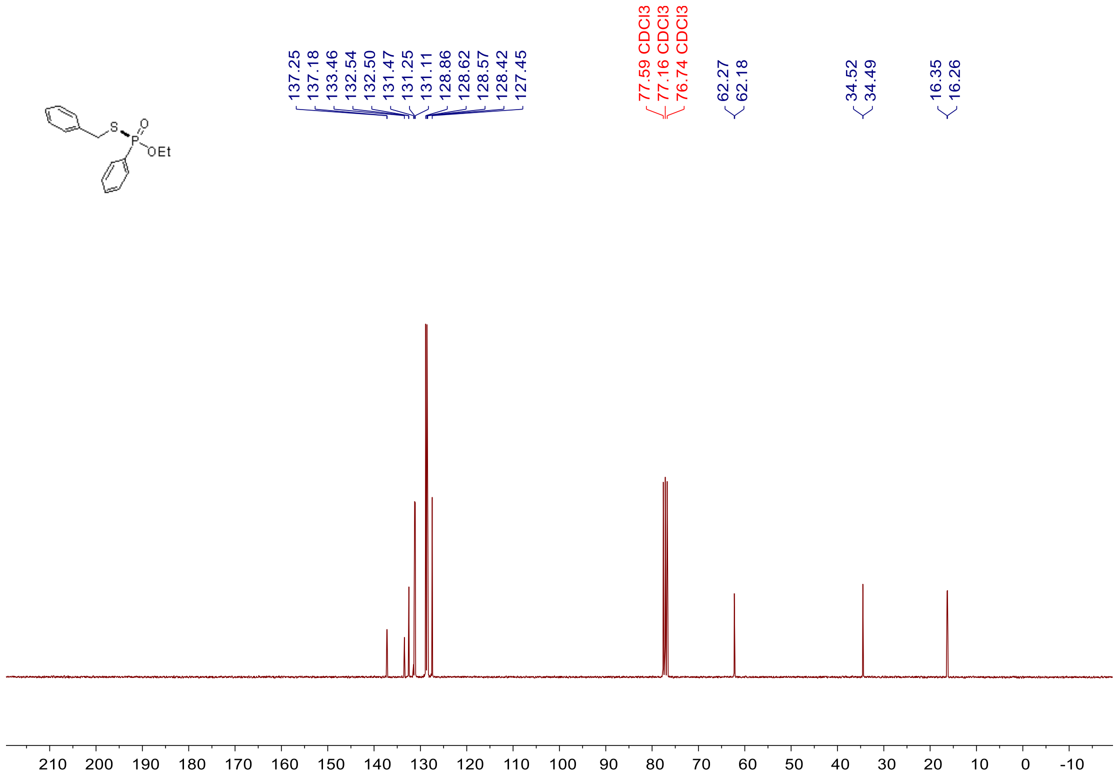

Figure S140. ^{13}C NMR spectra in $CDCl_3$ for Compound **5q**

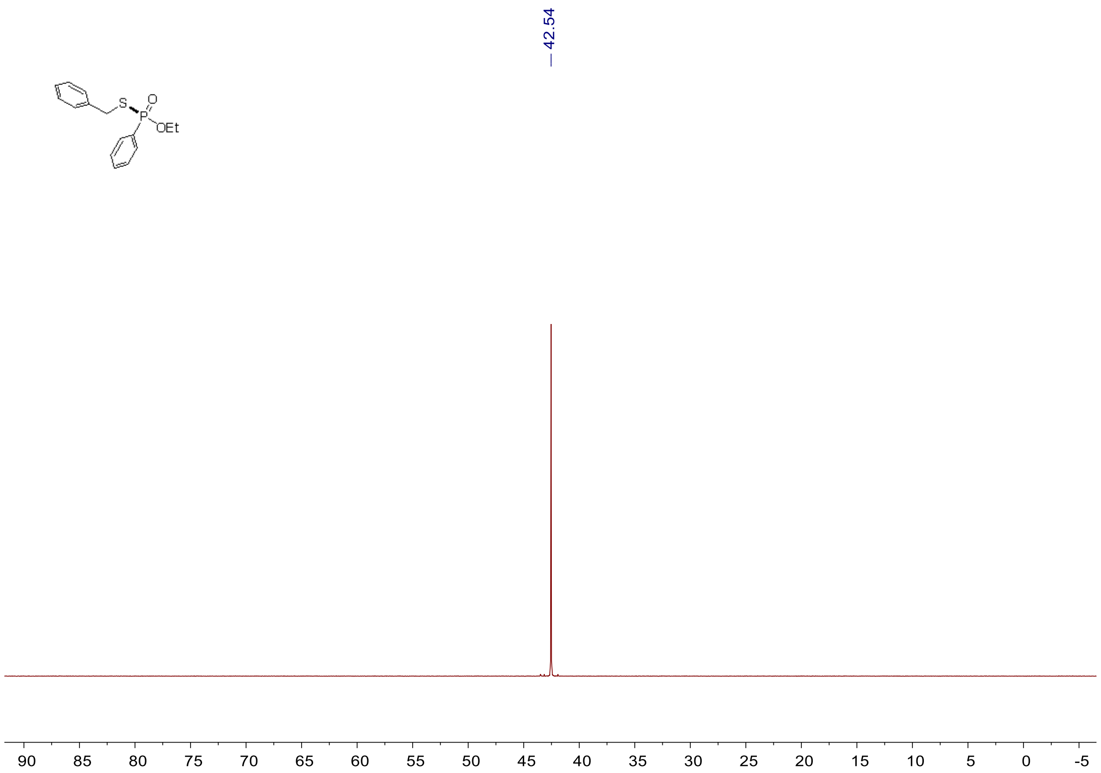

Figure S141. ^{31}P NMR spectra in $CDCl_3$ for Compound **5q**

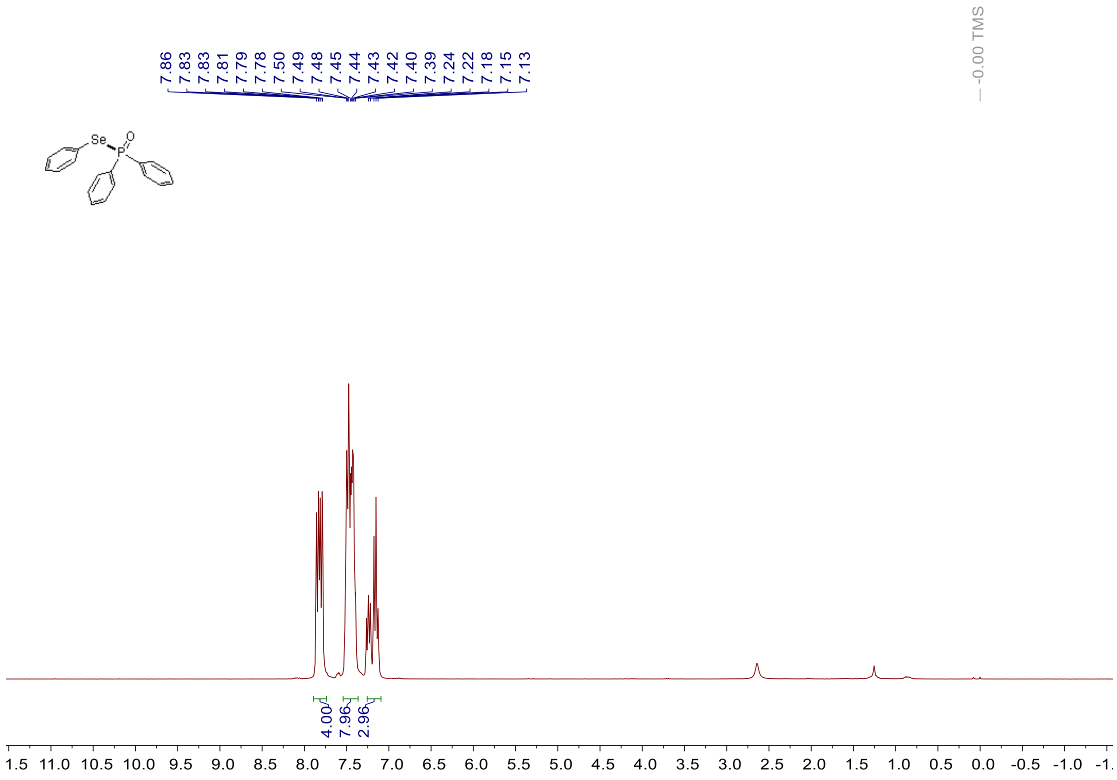

Figure S142. ^{1}H NMR spectra in $CDCl_3$ for Compound **5r**

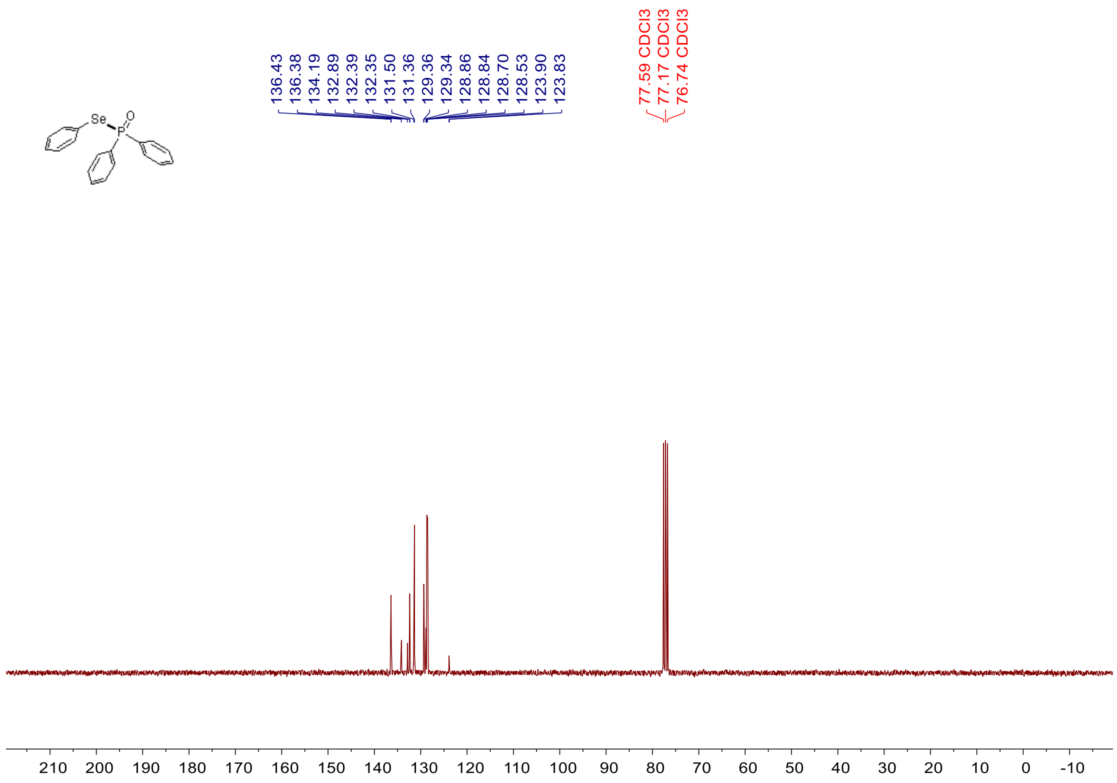

Figure S143. ^{13}C NMR spectra in $CDCl_3$ for Compound **5r**

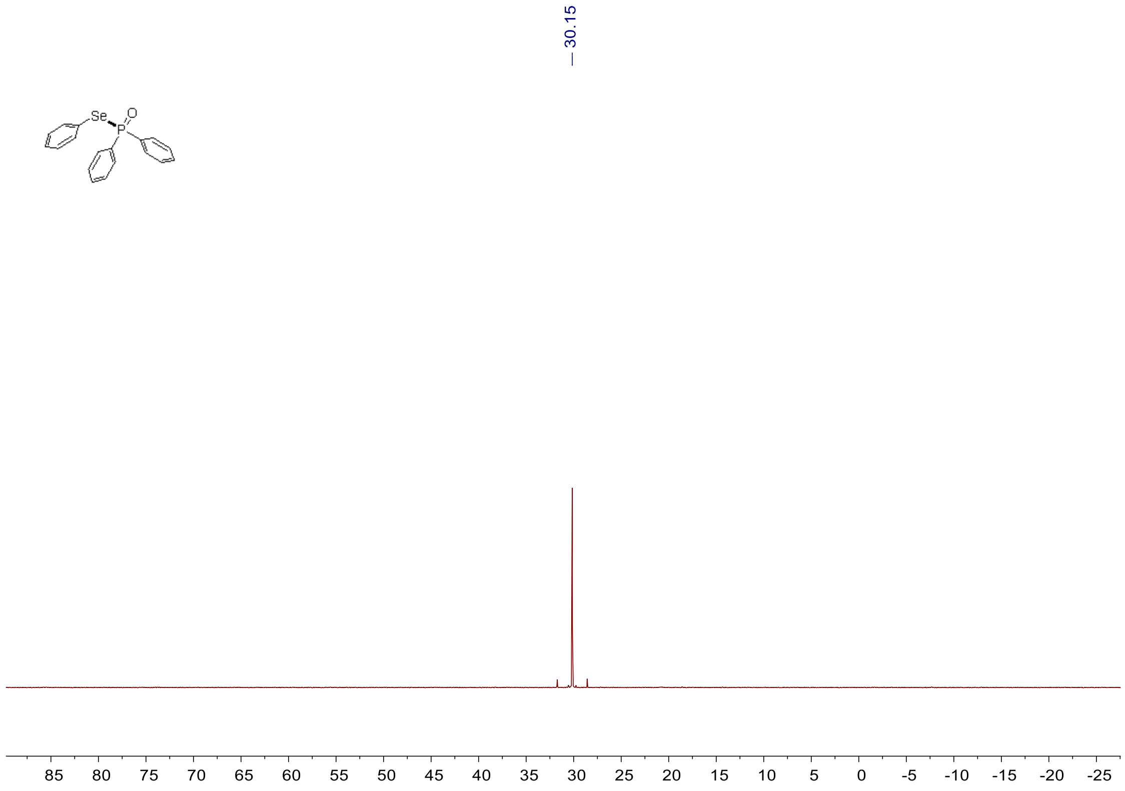

Figure S144. ^{31}P NMR spectra in $CDCl_3$ for Compound **5r**

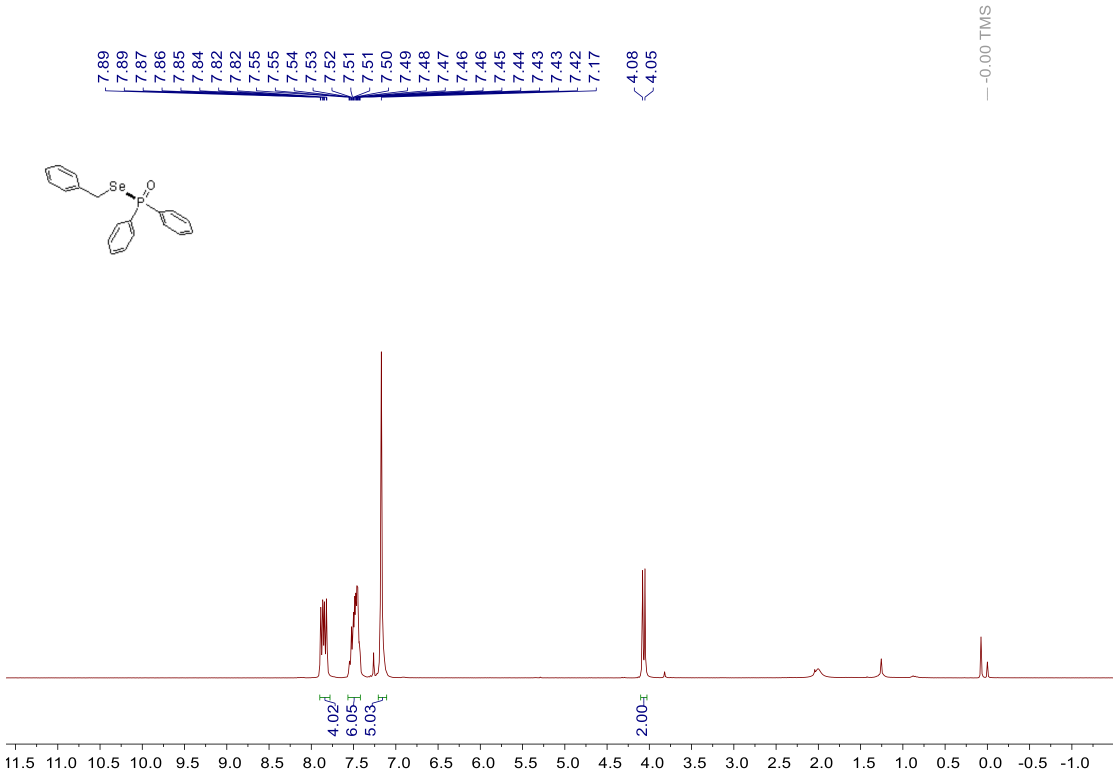

Figure S145. ^{1}H NMR spectra in $CDCl_3$ for Compound **5s**

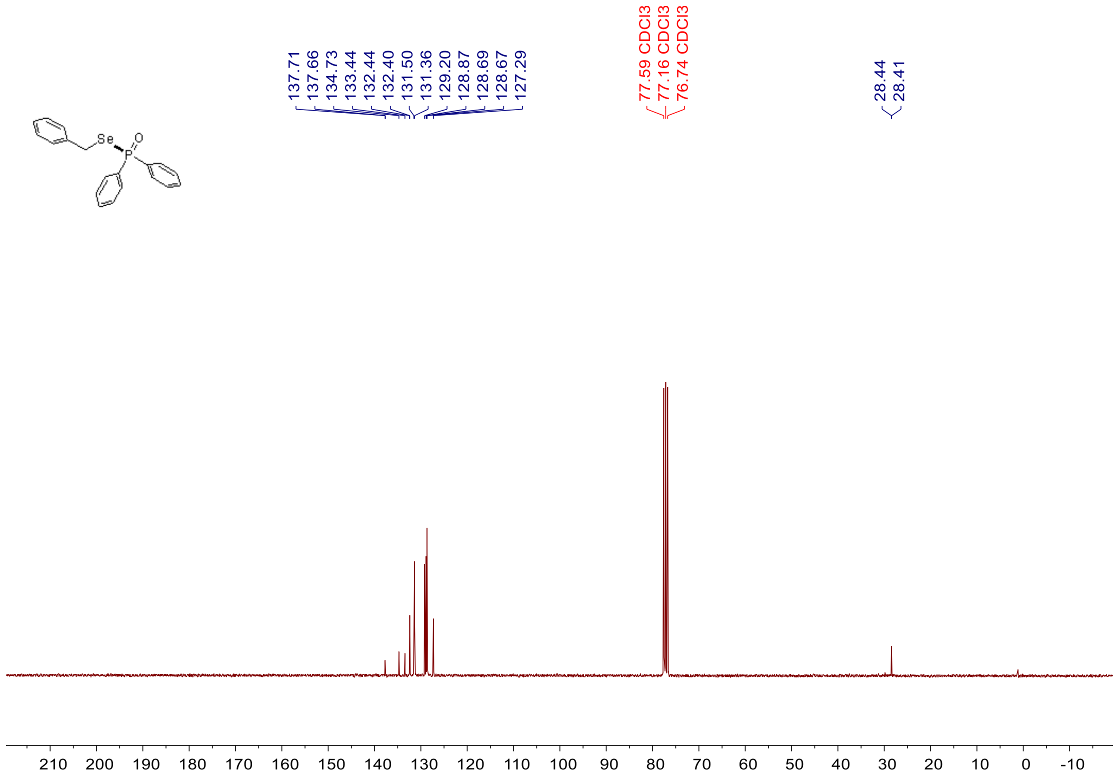

Figure S146. ^{13}C NMR spectra in $CDCl_3$ for Compound **5s**

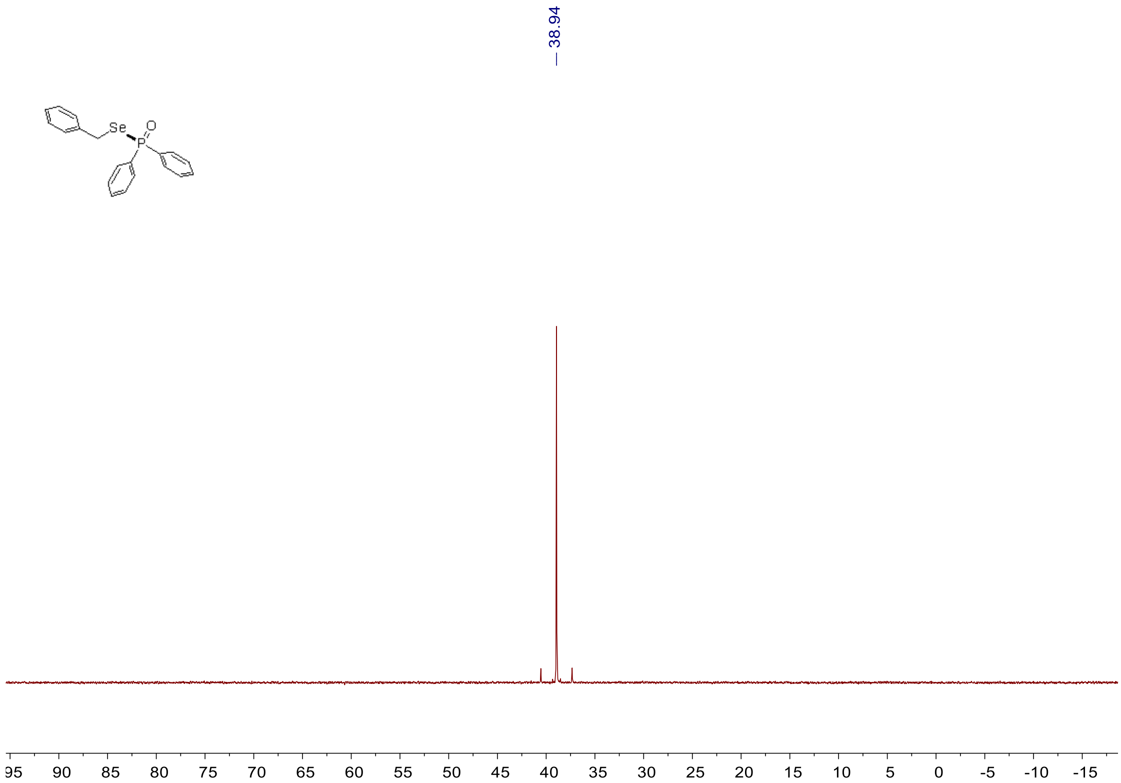

Figure S147. ^{31}P NMR spectra in $CDCl_3$ for Compound **5s**

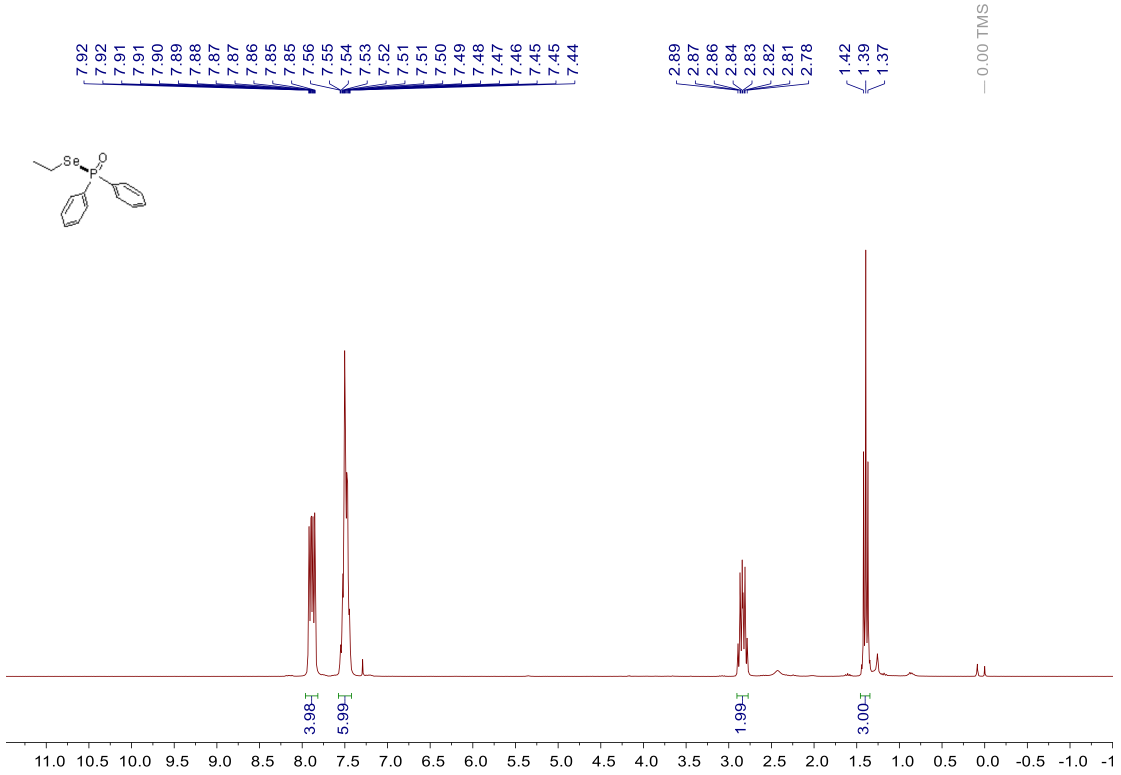

Figure S148. ^{1}H NMR spectra in $CDCl_3$ for Compound **5t**

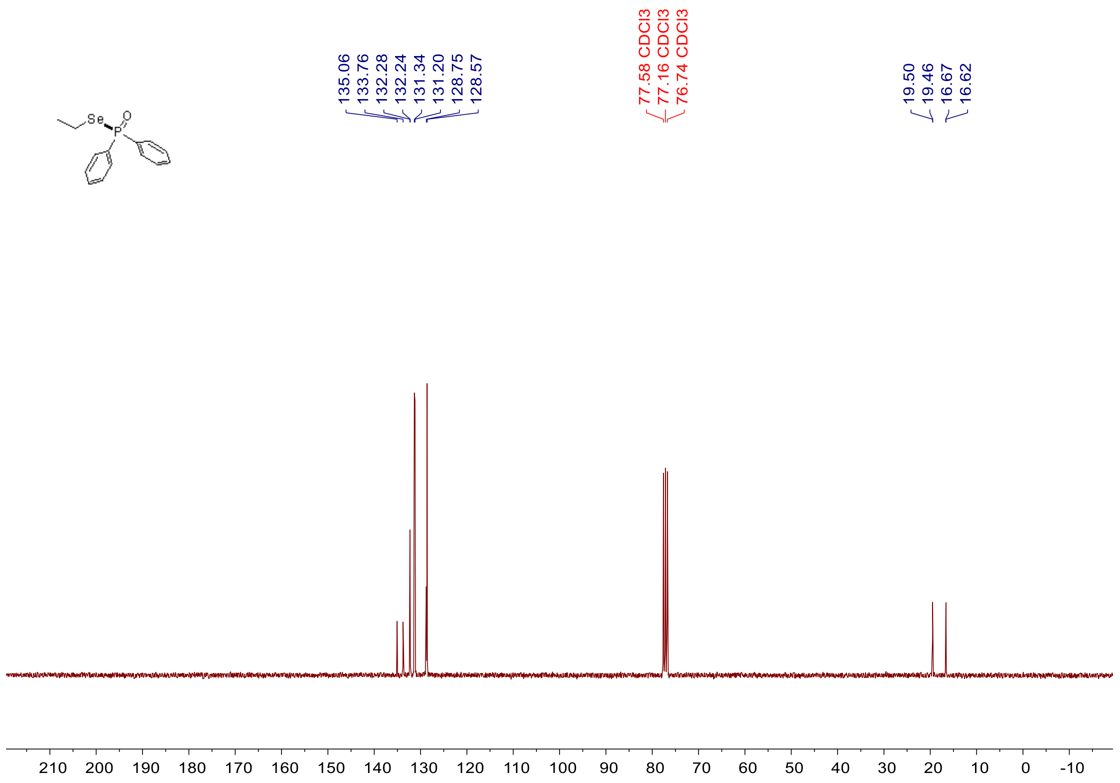

Figure S149. ^{13}C NMR spectra in $CDCl_3$ for Compound **5t**

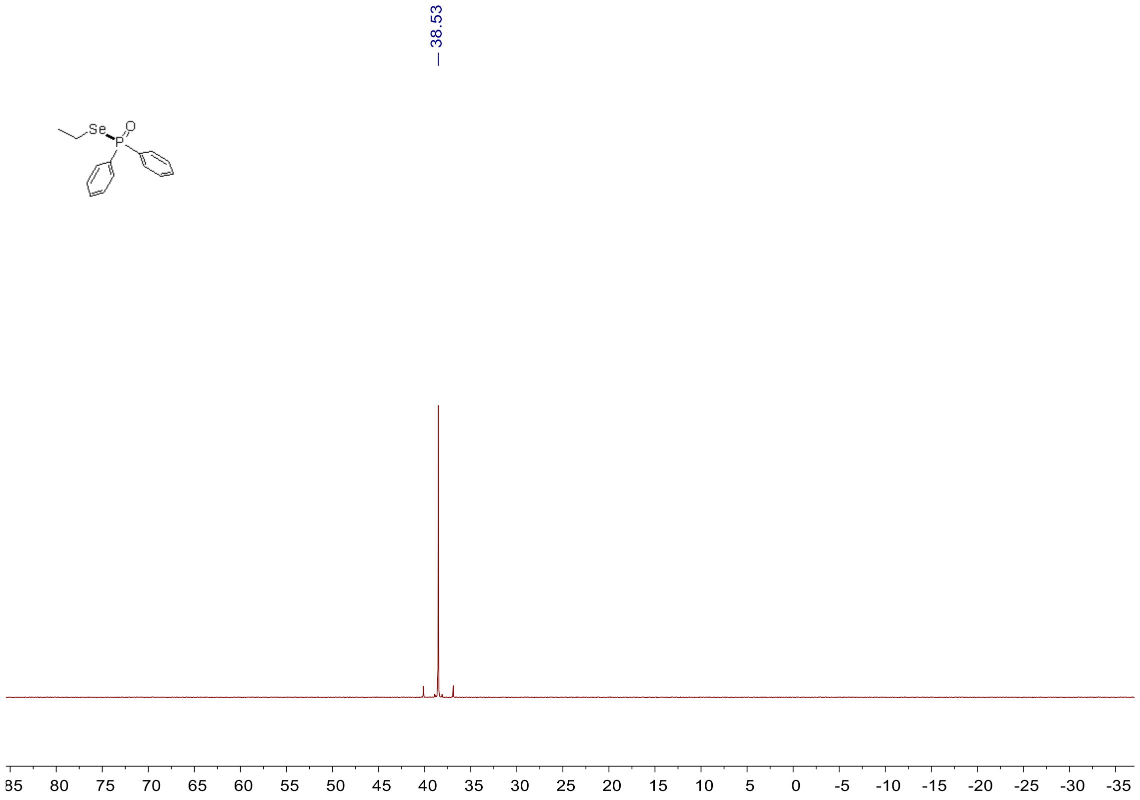

Figure S150. ^{31}P NMR spectra in $CDCl_3$ for Compound **5t**
